# Developmental disabilities among children younger than 5 years in 195 countries and territories, 1990–2016: a systematic analysis for the Global Burden of Disease Study 2016

**DOI:** 10.1016/S2214-109X(18)30309-7

**Published:** 2018-08-29

**Authors:** Global Research on Developmental Disabilities Collaborators, Bolajoko O. Olusanya, Adrian C. Davis, Donald Wertlieb, Nem-Yun Boo, M.K.C. Nair, Ricardo Halpern, Hannah Kuper, Cecilia Breinbauer, Petrus J de Vries, Melissa Gladstone, Neal Halfon, Vijaya Kancherla, Mphelekedzeni C. Mulaudzi, Angelina Kakooza-Mwesige, Felix A. Ogbo, Jacob O. Olusanya, Andrew N. Williams, Scott M. Wright, Helena Manguerra, Alison Smith, Michelle Echko, Chad Ikeda, Angela Liu, Anoushka Millear, Katherine Ballesteros, Emma Nichols, Holly E. Erskine, Damian Santomauro, Zane Rankin, Mari Smith, Harvey A. Whiteford, Helen E. Olsen, Nicholas J. Kassebaum

## Abstract

**Background:**

The Sustainable Development Goals (SDGs) mandate systematic monitoring of the health and wellbeing of all children to achieve optimal early childhood development. However, global epidemiological data on children with developmental disabilities are scarce. The Global Burden of Diseases, Injuries, and Risk Factors Study 2016 provides a comprehensive assessment of prevalence and years lived with disability (YLDs) for development disabilities among children younger than 5 years in 195 countries and territories from 1990 to 2016.

**Methods:**

We estimated prevalence and YLDs for epilepsy, intellectual disability, hearing loss, vision loss, autism spectrum disorder, and attention deficit hyperactivity disorder. YLDs were estimated as the product of the prevalence estimate and the disability weight for each mutually exclusive disorder, corrected for comorbidity. We used DisMod-MR 2.1, a Bayesian meta-regression tool, on a pool of primary data derived from systematic reviews of the literature, health surveys, hospital and claims databases, cohort studies, and disease-specific registries.

**Findings:**

Globally, 52·9 million (95% uncertainty interval [UI] 48·7–57·3; or 8·4% [7·7–9·1]) children younger than 5 years (54% males) had developmental disabilities in 2016 compared with 53·0 million (49·0–57·1; or 8·9% [8·2–9·5]) in 1990. About 95% of these children lived in low-income and middle-income countries. YLDs among these children increased from 3·8 million (95% UI 2·8–4·9) in 1990 to 3·9 million (2·9–5·2) in 2016. These disabilities accounted for 13·3% of the 29·3 million YLDs for all health conditions among children younger than 5 years in 2016. Vision loss was the most prevalent disability, followed by hearing loss, intellectual disability, and autism spectrum disorder. However, intellectual disability was the largest contributor to YLDs in both 1990 and 2016. Although the prevalence of developmental disabilities among children younger than 5 years decreased in all countries (except for North America) between 1990 and 2016, the number of children with developmental disabilities increased significantly in sub-Saharan Africa (71·3%) and in North Africa and the Middle East (7·6%). South Asia had the highest prevalence of children with developmental disabilities in 2016 and North America had the lowest.

**Interpretation:**

The global burden of developmental disabilities has not significantly improved since 1990, suggesting inadequate global attention on the developmental potential of children who survived childhood as a result of child survival programmes, particularly in sub-Saharan Africa and south Asia. The SDGs provide a framework for policy and action to address the needs of children with or at risk of developmental disabilities, particularly in resource-poor countries.

**Funding:**

The Bill & Melinda Gates Foundation.

## Introduction

Early childhood, commonly defined as the first 5 years of life, is the fastest period of growth and the period in which the developing brain is most sensitive to stimulation and nurturing.^1^ This period of development is regarded as the foundation for subsequent educational and vocational attainment at the individual level, and for overall human capital and economic development at the population level.^2^ The UN's Millennium Development Goals (MDGs) focused largely on reducing under-5 mortality, especially in low-income and middle-income countries (LMICs).^3^ By contrast, the UN's Sustainable Development Goals (SDGs) from 2015 to 2030 envision improvements in the broader health status of children beyond survival.^4^, ^5^ Alongside the general recognition of people with disabilities in several of the SDGs, SDG 4 specifically requires actions to monitor the proportion of children younger than 5 years who are achieving their developmental potential in health, education, and psychosocial wellbeing, disaggregated by disability, age, sex, geographic location, and other characteristics.

Developmental disabilities are a group of conditions resulting from impairments that affect a child's physical, learning, or behavioural functioning.^6^ Affected children typically have sensory impairments (hearing and vision loss), epilepsy or seizures, cerebral palsy, attention deficit hyperactivity disorder (ADHD), autism spectrum disorder (ASD), intellectual disability, or other learning disorders.^7^ Children with developmental delays and disabilities are at greater risk of suboptimal health, educational attainment, and wellbeing than are children without such disabilities.^8^ However, epidemiological data on developmental disabilities to guide comprehensive health policy engagements at global, regional, and national levels are scarce.^9^ The most widely reported data to date suggest that roughly 250 million children are at risk of suboptimal development in LMICs.^10^, ^11^ However, this number is based solely on children thought to be at risk of poor development because of stunting or extreme poverty and does not fully capture children with developmental disabilities.^10^ Another study^12^ based on UNICEF's Early Childhood Development Index estimated that 80·8 million children aged 3 or 4 years in LMICs had low cognitive or socioemotional development in 2010. That study was limited by the age group considered and the scope of developmental disabilities addressed. The Global Burden of Diseases, Injuries, and Risk Factors Study (GBD) offers an independent source of robust age-specific data on non-fatal health outcomes.^13^, ^14^ We aimed to estimate the prevalence of disability and years lived with disability (YLDs) among children younger than 5 years with developmental disabilities based on findings from GBD 2016. This analysis provides baseline data for monitoring the trends in these metrics over time among children younger than 5 years during the SDG era. Leading causes of specific developmental disabilities are also examined.

**Research in context**

**Evidence before this study**

Since 2007, *The Lancet* Series on early childhood development has provided estimates of children at risk of suboptimal development in low-income and middle-income countries (LMICs). However, similar to previous estimates, the most recent estimate of 250 million at-risk children, which was derived from 141 countries, is restricted to those who are stunted or exposed to extreme poverty in LMICs and excludes the vast majority of children with developmental delays and disabilities. More comprehensive baseline data that directly describe dimensions of health related to neurocognitive development are required for monitoring the proportion of children younger than 5 years who are at risk of suboptimal development in health, learning, and wellbeing globally as mandated by the UN's Sustainable Development Goals (SDGs). The Global Burden of Diseases, Injuries, and Risk Factors Study 2016 (GBD 2016) produced comprehensive and comparable estimates of age-specific health disorders for 195 countries and territories from 1990 to 2016.

**Added value of this study**

This study reports GBD 2016 estimates of the prevalence and years lived with disability for developmental disabilities among children younger than 5 years, including epilepsy, intellectual disability, vision loss, hearing loss, autism spectrum disorder (ASD), and attention deficit hyperactivity disorder (ADHD). To our knowledge, this study is the first to investigate trends in the burden of these developmental disabilities between 1990 and 2016 and to provide baseline data for monitoring progress at the global, regional, and country levels under the SDGs.

**Implications of all the available evidence**

Although under-5 mortality halved between 1990 and 2016, there has not been a corresponding improvement in non-fatal health outcomes among childhood survivors globally. The number of children younger than 5 years at risk of suboptimal development in LMICs is likely to exceed 350 million (roughly three in every five children), even without inclusion of all known disabilities in GBD 2016. The absence of any systematic attention to developmental disabilities has had greatest effect in sub-Saharan Africa, where the number of affected children increased by more than 70% between 1990 and 2016, despite an overall decrease in prevalence worldwide during this period. The SDGs now present a comprehensive framework for addressing the burden of developmental delays and disabilities among survivors of the leading causes of child mortality in the early years of life, especially in LMICs. More crucially, local health and educational systems should be appropriately equipped to support affected children and their families optimally. Although the prevalence of conditions such as ASD and ADHD typically peak at school age or later, some children will require timely intervention from early childhood. Global investment is needed to improve primary data sources for developmental disabilities to minimise uncertainty around the estimates of non-fatal health outcomes in most countries.

## Methods

### Overview

This study complies with Guidelines for Accurate and Transparent Health Estimates Reporting (GATHER) recommendations.^15^ An overview of the general modelling strategies for the non-fatal health outcomes of 328 diseases and conditions, as defined in International Classification of Diseases (ICD) codes 9 and 10, is provided in the appendix. Detailed descriptions of the modelling approaches for each of the four impairments (epilepsy, intellectual disability, hearing loss, and vision loss) and two GBD causes (ASD and ADHD) included in this report, along with specific data, diagnostic, and modelling considerations for each, are also provided in the appendix.

The causes included in estimation of each of the impairments are shown in the appendix. Although cerebral palsy was not estimated as a separate impairment, estimates of its prevalence and YLDs are included in those of intellectual disability because of overlapping, cause-specific perinatal complications, including neonatal preterm birth complications, neonatal encephalopathy due to asphyxia and birth trauma, neonatal sepsis and other neonatal infections, and kernicterus after neonatal jaundice. We considered all developmental disorders or impairments as proxies for developmental disabilities in this study.

The two metrics, prevalence and YLDs, associated with developmental disorders in the GBD study are collectively referred to as the burden of disability in this study, without prejudice towards any ethical, sociocultural, or public health constructs of disability in the literature. Prevalence estimates are stated in both absolute numbers and per 100 000 children, in keeping with the practice for all conditions in the GBD study. The term disability is used to describe the perceived short-term or long-term loss of health (and not welfare loss) associated with a condition, which is reflected through an estimated disability weight.^16^ Thus, YLDs do not strictly measure disability from a public health perspective based on WHO's International Classification of Functioning, Disability and Health (ICF).^17^ Rather, the metric seeks to provide a comparable measure of disease burden across diverse health conditions and impairments.^13^

Case definitions and diagnostic criteria were based on ICD-9 and ICD-10 codes complemented with relevant guidelines, such as the Diagnostic and Statistical Manual of Mental Disorders (DSM)-IV-TR and the Guidelines for Epidemiologic Studies on Epilepsy.^18^, ^19^ Developmental intellectual disability was separated into five bands based on intelligence quotient scores: borderline, mild, moderate, severe, and profound. Hearing and visual impairments were similarly separated into bands of severity corresponding to frequency response and visual acuity cutoffs, respectively. The age at which conditions such as ASD and ADHD could be diagnosed in early childhood was also considered in input data evaluation and model development.

### Data sources and modelling strategy

The GBD estimation strategy is designed to provide a standardised analytical approach for estimating prevalence and YLDs by age, sex, cause, year, and location. The first step in the estimation of each condition was compilation of all available data inputs from systematic reviews of the literature, hospital and claims databases, health surveys, case notification systems, cohort studies, and multinational survey data. All input data for GBD 2016 are available at the Global Health Data Exchange. Effort was made to optimise the comparability of data derived from different sources using different methods, to find a consistent set of estimates across prevalence data, and to generate estimates for locations with sparse or no data by use of available information from other locations combined with covariates.^13^ We obtained prevalence estimates by age group from 1990 to 2016 using DisMod-MR 2.1, a statistical modelling, Bayesian meta-regression tool developed for the GBD project.^20^ DisMod-MR 2.1 synthesises epidemiological data for non-fatal health outcomes from disparate settings and sources, adjusting for different case definitions or diagnostic criteria and sampling methods to generate internally consistent estimates of prevalence, incidence, remission, and mortality by location, year, age group, and sex. Estimation in DisMod-MR 2.1 occurs sequentially at five levels: global, super-region, region, country, and subnational. Results from higher levels guide analyses at lower geographical levels. Model parameters, input data values, and fit statistics for each component model are viewable in the publicly available Epi Visualisation tool.

After generating internally consistent estimates of incidence, prevalence, remission, and mortality for each condition, prevalent cases were distributed among a set of mutually exclusive and collectively exhaustive sequelae (ie, severity of the disease state). The complete sequela list for each cause and impairment are provided in their corresponding methods description in the appendix. After initial calculation of sequela-level prevalence, GBD ensured that the sum of all causes leading to each impairment was scaled to equal the total number of cases of the impairment. Because of the commonality of co-occurrence of epilepsy, blindness, and intellectual disability arising from many causes (eg, neonatal complications leading to cerebral palsy, long-term complications of cerebral malaria and meningitis), scaling the prevalence sums of each impairment was done sequentially. After scaling, each sequela was paired with a unique health state describing the associated disability. Each health state had a corresponding disability weight that was estimated with pairwise comparison methods that presented pairs of lay health-state descriptions to more than 60 000 respondents in open web-based surveys done among the general population in nine countries (Bangladesh, Hungary, Indonesia, Italy, Peru, Sweden, Tanzania, the USA, and the Netherlands). Disability weights ranged between 0 (perfect health) and 1 (death),^16^ and were assumed to be similar in different locations. By contrast, the distribution of sequelae varied by location, year, sex, and age. Sequela-level prevalence estimates were multiplied by the disability weights to generate YLDs. YLDs for each sequela were then adjusted for comorbidity with a microsimulation framework that assumed that comorbidity within each age group, sex, location, and year was independent.

All computations in GBD were done 1000 times, each time drawing from the distribution of the sampling error of data inputs, the uncertainty of data corrections for measurement errors, the uncertainty in coefficients from model fit, and the uncertainty of severity distributions and disability weights. Uncertainty intervals (UIs) were defined by the 25th and 975th values of the ordered 1000 estimate values. Changes in estimates between locations or over time that were in the same direction in more than 950 of the 1000 samples were considered as significant. Final estimates for children younger than 5 years were compiled by summing the prevalence and YLD estimates in the first four GBD age groups: early neonatal (0–6 days), late neonatal (7–27 days), post-neonatal (28–364 days), and 1–4 years. The aggregate figures for each of the impairments are independent from one another so the total burden from all developmental impairments is somewhat less than the sum of each impairment because of comorbidities.

### Role of the funding source

The funder of the study had no role in study design, data collection, data analysis, data interpretation, or the writing of the report. All authors had full access to the data in the study and had final responsibility for the decision to submit for publication.

## Results

### Global trend in prevalence and YLDs of developmental disabilities

The estimated global number of children younger than 5 years in GBD 2016 was 598·5 million in 1990, 600·2 million in 2005, 626·9 million in 2010, and 632·0 million in 2016. The total number of children with any of the six developmental disabilities after adjusting for comorbidity between intellectual disability and ASD was 53·0 million (95% UI 49·0–57·1 [8·9%, 8·2–9·5]) in 1990 compared with 52·9 million (48·7–57·3 [8·4%, 7·7–9·1]) in 2016. Of the children with developmental disabilities in 2016, 2·7 million (5·1%) lived in high-income countries and 50·2 million (94·9%) in LMICs (table 1). About 54% of children with any developmental disability were male, although the proportions of male and female children varied by type of impairment.

**Table 1:** Number and prevalence per 100 000 population of cases of developmental disability and of YLDs in children younger than 5 years globally and by SDI group, GBD region and super-region, country, and territory in 1990 and 2016

			**Number of cases**		**Cases per 100 000 population**		**Number of YLDs**		**YLDs per 100 000 population**	
			1990	2016	1990	2016	1990	2016	1990	2016
**Global**			**53 003 423 (48 994 705 to 57 127 440)**	**52 856 396 (48 706 753 to 57 252 926)**	**8856·5 (8186·7 to 9545·6)**	**8363·7 (7707·1 to 9059·4)**	**3 755 596 (2 816 709 to 4 880 859)**	**3 941 530 (2 905 872 to 5 226 909)**	**627·5 (470·6 to 815·6)**	**623·7 (459·8 to 827·1)**
		High SDI	2 967 871 (2 704 588 to 3 255 250)	2 540 450 (2 313 657 to 2 793 877)	5019·0 (4573·8 to 5505·0)	4723·3 (4301·6 to 5194·4)	226 493 (163 992 to 299 929)	197 289 (143 533 to 261 063)	383·0 (277·3 to 507·2)	366·8 (266·9 to 485·4)
		High-middle SDI	6 365 691 (5 860 792 to 6 907 595)	4 935 445 (4 524 536 to 5 356 919)	7718·6 (7106·4 to 8375·7)	6776·7 (6212·5 to 7355·4)	457 376 (334 911 to 611 639)	355 258 (264 684 to 470 853)	554·6 (406·1 to 741·6)	487·8 (363·4 to 646·5)
		Middle SDI	17 332 181 (16 058 042 to 18 647 376)	12 465 029 (11 485 979 to 13 470 308)	8449·2 (7828·0 to 9090·3)	7839·3 (7223·5 to 8471·5)	1 180 392 (880 452 to 1 537 019)	879 242 (652 657 to 1 156 149)	575·4 (429·2 to 749·3)	553·0 (410·5 to 727·1)
		Low-middle SDI	19 191 346 (17 654 271 to 20 819 431)	20 535 682 (18 751 245 to 22 351 779)	10 199·9 (9382·9 to 11 065·2)	9205·5 (8405·6 to 10 019·6)	1 404 664 (1 057 073 to 1 805 141)	1 574 971 (1 169 784 to 2 084 686)	746·5 (561·8 to 959·4)	706·0 (524·4 to 934·5)
		Low SDI	7 070 383 (6 475 315 to 7 664 120)	12 433 791 (11 397 088 to 13 517 025)	11 179·2 (10 238·4 to 12 118·0)	10 115·2 (9271·8 to 10 996·4)	481 760 (361 732 to 630 102)	942 926 (685 216 to 1 261 145)	761·7 (572·0 to 996·3)	767·1 (557·4 to 1026·0)
	High income		2 905 208 (2 637 546 to 3 205 994)	2 694 377 (2 449 641 to 2 967 846)	4843·6 (4397·3 to 5345·1)	4668·7 (4244·6 to 5142·6)	226 072 (164 411 to 298 305)	210 347 (152 883 to 276 346)	376·9 (274·1 to 497·3)	364·5 (264·9 to 478·8)
	High-income North America		835 136 (764 021 to 912 681)	884 082 (807 346 to 965 387)	3913·6 (3580·3 to 4276·9)	4090·4 (3735·4 to 4466·6)	72 660 (53 004 to 96 191)	74 837 (54 966 to 97 397)	340·5 (248·4 to 450·8)	346·2 (254·3 to 450·6)
		Canada	67 891 (61 657 to 74 437)	73 995 (66 866 to 81 405)	3590·1 (3260·5 to 3936·2)	3814·7 (3447·2 to 4196·8)	5437 (3900 to 7258)	5821 (4169 to 8128)	287·5 (206·2 to 383·8)	300·1 (214·9 to 419·0)
		Greenland	205 (184 to 225)	137 (124 to 151)	3908·7 (3518·0 to 4298·3)	4020·7 (3643·1 to 4429·0)	18 (13 to 25)	12 (8 to 16)	346·5 (239·2 to 481·7)	338·0 (239·3 to 463·3)
		USA	766 633 (699 536 to 838 170)	809 661 (740 178 to 883 771)	3945·0 (3599·8 to 4313·1)	4117·6 (3764·3 to 4494·5)	67 169 (48 845 to 89 286)	68 980 (50 734 to 89 782)	345·6 (251·3 to 459·5)	350·8 (258·0 to 456·6)
	Australasia		75 024 (68 481 to 82 443)	85 028 (77 631 to 93 120)	4994·4 (4558·9 to 5488·3)	4787·0 (4370·6 to 5242·6)	5539 (3952 to 7497)	6144 (4407 to 8370)	368·7 (263·1 to 499·1)	345·9 (248·1 to 471·2)
		Australia	61 054 (55 427 to 67 201)	71 005 (64 778 to 77 641)	4988·5 (4528·7 to 5490·7)	4763·8 (4346·0 to 5208·9)	4478 (3172 to 6116)	5064 (3578 to 6918)	365·9 (259·2 to 499·8)	339·8 (240·1 to 464·1)
		New Zealand	13 969 (12 785 to 15 346)	14 023 (12 726 to 15 422)	5020·1 (4594·6 to 5514·8)	4908·4 (4454·3 to 5397·9)	1061 (763 to 1441)	1080 (774 to 1451)	381·4 (274·2 to 517·8)	377·9 (271·1 to 507·8)
	High-income Asia Pacific		539 783 (488 294 to 597 587)	366 621 (330 750 to 406 893)	5393·1 (4878·7 to 5970·6)	4986·3 (4498·4 to 5534·0)	38 940 (28 299 to 51 710)	25 990 (18 853 to 34 820)	389·1 (282·8 to 516·6)	353·5 (256·4 to 473·6)
		Brunei	1783 (1606 to 1982)	1641 (1478 to 1830)	5385·1 (4852·4 to 5987·6)	4976·9 (4483·1 to 5550·0)	139 (96 to 196)	130 (91 to 190)	420·0 (288·6 to 590·9)	394·1 (275·6 to 575·3)
		Japan	346 604 (313 191 to 383 210)	253 381 (228 552 to 280 813)	5298·2 (4787·5 to 5857·8)	5051·7 (4556·6 to 5598·6)	24 440 (17 699 to 32 810)	17 776 (12 812 to 23 743)	373·6 (270·5 to 501·5)	354·4 (255·4 to 473·4)
		Singapore	13 264 (11 933 to 14 717)	8462 (7587 to 9508)	5461·4 (4913·5 to 6059·8)	4784·5 (4289·7 to 5376·0)	940 (659 to 1287)	595 (410 to 846)	387·1 (271·3 to 530·1)	336·5 (232·0 to 478·2)
		South Korea	178 133 (159 930 to 198 212)	103 136 (92 616 to 115 398)	5582·6 (5012·1 to 6211·9)	4849·0 (4354·4 to 5425·5)	13 421 (9416 to 18 424)	7489 (5248 to 10 275)	420·6 (295·1 to 577·4)	352·1 (246·7 to 483·1)
	Western Europe		1 173 283 (1 059 486 to 1 311 058)	1 082 083 (973 879 to 1 209 214)	5191·8 (4688·3 to 5801·5)	4930·8 (4437·7 to 5510·1)	89 500 (64 632 to 118 938)	84 095 (59 931 to 111 706)	396·0 (286·0 to 526·3)	383·2 (273·1 to 509·0)
		Andorra	109 (92 to 134)	145 (124 to 177)	4866·2 (4120·6 to 5982·5)	4750·2 (4061·0 to 5793·9)	8 (6 to 11)	11 (8 to 15)	354·1 (248·2 to 493·1)	349·4 (248·1 to 474·9)
		Austria	21 955 (18 758 to 26 826)	19 741 (16 980 to 24 074)	5087·4 (4346·6 to 6215·9)	4911·2 (4224·4 to 5989·2)	1624 (1167 to 2265)	1481 (1027 to 2026)	376·2 (270·4 to 524·7)	368·6 (255·5 to 504·0)
		Belgium	29 948 (25 583 to 35 957)	29 674 (25 389 to 36 087)	5076·5 (4336·6 to 6095·1)	4695·5 (4017·5 to 5710·3)	2151 (1503 to 3022)	2121 (1454 to 2996)	364·7 (254·7 to 512·3)	335·6 (230·1 to 474·1)
		Cyprus	3154 (2682 to 3877)	2370 (2014 to 2878)	5316·1 (4520·2 to 6533·4)	4776·2 (4059·1 to 5799·7)	244 (171 to 337)	184 (130 to 250)	411·2 (289·0 to 568·0)	370·8 (262·6 to 504·5)
		Denmark	10 307 (9351 to 11 382)	9913 (8935 to 11 008)	3534·5 (3206·9 to 3903·2)	3437·4 (3098·3 to 3817·1)	931 (667 to 1266)	902 (640 to 1214)	319·2 (228·7 to 434·1)	312·7 (222·0 to 421·0)
		Finland	14 959 (13 040 to 17 948)	13 513 (11 815 to 16 024)	4799·0 (4183·4 to 5758·2)	4652·4 (4067·6 to 5516·9)	1117 (789 to 1497)	1040 (739 to 1414)	358·2 (253·1 to 480·2)	358·1 (254·2 to 487·0)
		France	177 256 (159 525 to 195 623)	174 389 (156 592 to 194 448)	4665·0 (4198·4 to 5148·4)	4449·8 (3995·7 to 4961·6)	14 223 (9599 to 19 901)	14 036 (9440 to 20 255)	374·3 (252·6 to 523·8)	358·1 (240·9 to 516·8)
		Germany	253 341 (223 086 to 295 280)	197 394 (172 539 to 231 256)	5948·8 (5238·4 to 6933·6)	5755·6 (5030·9 to 6743·0)	18 992 (13 381 to 25 921)	15 773 (10 669 to 22 650)	446·0 (314·2 to 608·7)	459·9 (311·1 to 660·4)
		Greece	24 323 (21 272 to 27 872)	20 458 (17 821 to 23 515)	4622·7 (4042·8 to 5297·3)	4329·3 (3771·3 to 4976·2)	2062 (1427 to 2851)	1675 (1176 to 2285)	391·9 (271·3 to 541·8)	354·5 (248·9 to 483·6)
		Iceland	976 (863 to 1116)	929 (817 to 1060)	4501·1 (3982·1 to 5145·8)	4329·6 (3807·2 to 4941·9)	77 (55 to 106)	74 (51 to 101)	355·3 (253·7 to 489·6)	342·8 (237·7 to 472·1)
		Ireland	15 128 (12 989 to 18 321)	16 515 (14 042 to 19 950)	5419·6 (4653·4 to 6563·6)	4909·7 (4174·6 to 5930·8)	1139 (785 to 1561)	1249 (869 to 1727)	408·1 (281·3 to 559·1)	371·3 (258·3 to 513·3)
		Israel	33 959 (30 389 to 38 116)	52 288 (46 756 to 58 947)	6709·8 (6004·5 to 7531·1)	6241·4 (5581·1 to 7036·3)	2328 (1652 to 3095)	3563 (2500 to 4891)	460·0 (326·4 to 611·5)	425·3 (298·4 to 583·9)
		Italy	151 859 (135 089 to 171 954)	137 195 (122 240 to 154 091)	5400·7 (4804·3 to 6115·4)	5399·3 (4810·7 to 6064·2)	11 120 (7779 to 15 483)	10 121 (7130 to 13 894)	395·5 (276·7 to 550·6)	398·3 (280·6 to 546·8)
		Luxembourg	1204 (1044 to 1458)	1523 (1317 to 1825)	5369·4 (4656·5 to 6505·1)	5040·8 (4360·6 to 6040·1)	87 (62 to 122)	109 (76 to 150)	389·6 (276·5 to 543·9)	359·6 (252·3 to 497·7)
		Malta	1574 (1347 to 1919)	945 (813 to 1141)	5389·1 (4612·1 to 6572·5)	4954·4 (4264·3 to 5981·5)	120 (86 to 161)	73 (51 to 100)	411·2 (295·5 to 552·0)	382·5 (267·1 to 523·0)
		Netherlands	40 853 (36 356 to 46 079)	36 719 (32 416 to 41 479)	4397·7 (3913·6 to 4960·3)	4221·4 (3726·7 to 4768·6)	3212 (2257 to 4454)	3014 (2104 to 4158)	345·7 (243·0 to 479·4)	346·5 (241·8 to 478·1)
		Norway	11 786 (10 643 to 12 943)	11 986 (10 828 to 13 188)	4202·9 (3795·3 to 4615·5)	4033·5 (3643·8 to 4438·0)	965 (686 to 1323)	990 (698 to 1347)	344·2 (244·8 to 471·9)	333·0 (234·8 to 453·4)
		Portugal	32 308 (27 466 to 39 467)	20 624 (17 371 to 25 237)	5412·3 (4601·1 to 6611·5)	4867·5 (4099·8 to 5956·2)	2272 (1531 to 3173)	1425 (987 to 1969)	380·6 (256·5 to 531·6)	336·4 (232·9 to 464·8)
		Spain	117 273 (104 677 to 131 719)	111 722 (99 356 to 126 240)	5708·0 (5094·9 to 6411·2)	5158·9 (4587·9 to 5829·3)	8172 (5653 to 11 028)	7906 (5420 to 10 923)	397·8 (275·1 to 536·8)	365·1 (250·3 to 504·4)
		Sweden	20 042 (18 295 to 22 066)	21 546 (19 678 to 23 635)	3620·9 (3305·2 to 3986·5)	3767·3 (3440·7 to 4132·4)	1812 (1310 to 2424)	1957 (1399 to 2590)	327·4 (236·8 to 437·8)	342·1 (244·5 to 452·9)
		Switzerland	18 184 (15 484 to 22 460)	18 488 (15 699 to 22 724)	4651·2 (3960·5 to 5745·1)	4436·5 (3767·1 to 5452·8)	1421 (995 to 1945)	1505 (1070 to 2019)	363·4 (254·5 to 497·5)	361·2 (256·7 to 484·5)
		UK	191 620 (174 747 to 212 402)	182 856 (166 130 to 201 906)	5003·8 (4563·2 to 5546·5)	4683·0 (4254·6 to 5170·8)	15 333 (11 172 to 20 260)	14 799 (10 818 to 19 495)	400·4 (291·8 to 529·0)	379·0 (277·1 to 499·3)
	Southern Latin America		281 982 (257 534 to 307 105)	276 563 (252 233 to 302 846)	6222·8 (5683·3 to 6777·2)	5505·4 (5021·1 to 6028·6)	19 434 (13 724 to 25 976)	19 281 (13 756 to 26 016)	428·9 (302·9 to 573·2)	383·8 (273·8 to 517·9)
		Argentina	179 819 (164 295 to 196 731)	194 364 (177 338 to 212 052)	6099·7 (5573·1 to 6673·3)	5426·7 (4951·3 to 5920·5)	12 288 (8594 to 16 518)	13 533 (9443 to 18 633)	416·8 (291·5 to 560·3)	377·8 (263·6 to 520·2)
		Chile	86 933 (78 862 to 95 723)	68 590 (61 951 to 76 189)	6462·2 (5862·3 to 7115·6)	5696·4 (5145·0 to 6327·5)	6057 (4085 to 8318)	4817 (3311 to 6841)	450·2 (303·6 to 618·4)	400·1 (275·0 to 568·2)
		Uruguay	15 222 (13 734 to 16 837)	13 600 (12 319 to 14 968)	6394·3 (5769·4 to 7073·1)	5724·1 (5185·1 to 6300·2)	1088 (735 to 1576)	930 (640 to 1335)	456·9 (308·7 to 662·1)	391·4 (269·5 to 562·0)
Central Europe, eastern Europe, and central Asia			2 687 620 (2 464 817 to 2 925 101)	2 011 623 (1 837 692 to 2 186 726)	7682·3 (7045·4 to 8361·1)	7135·2 (6518·2 to 7756·2)	180 653 (132 858 to 238 834)	140 511 (104 528 to 185 395)	516·4 (379·8 to 682·7)	498·4 (370·8 to 657·6)
	Eastern Europe		1 194 573 (1 094 032 to 1 302 962)	884 720 (805 652 to 961 355)	7206·9 (6600·4 to 7860·9)	6827·2 (6217·1 to 7418·6)	84 134 (61 742 to 113 932)	63 846 (45 981 to 85 258)	507·6 (372·5 to 687·4)	492·7 (354·8 to 657·9)
		Belarus	52 235 (46 911 to 57 845)	35 765 (31 945 to 39 494)	6757·4 (6068·6 to 7483·1)	6210·4 (5547·0 to 6857·7)	3925 (2865 to 5242)	2697 (1938 to 3588)	507·7 (370·6 to 678·2)	468·2 (336·6 to 623·1)
		Estonia	6511 (5933 to 7134)	3447 (3127 to 3769)	5496·4 (5009·0 to 6023·1)	5029·4 (4562·9 to 5498·6)	584 (421 to 792)	285 (206 to 384)	493·1 (355·1 to 668·3)	415·8 (300·9 to 560·9)
		Latvia	14 195 (13 006 to 15 435)	7089 (6487 to 7767)	7171·1 (6570·3 to 7797·5)	6700·4 (6131·0 to 7341·1)	968 (698 to 1280)	492 (357 to 657)	489·0 (352·4 to 646·8)	465·4 (337·8 to 621·2)
		Lithuania	18 247 (16 425 to 20 130)	8959 (8019 to 9907)	6399·0 (5760·1 to 7059·2)	5960·8 (5335·2 to 6591·5)	1372 (981 to 1888)	712 (520 to 945)	481·1 (344·1 to 662·0)	473·9 (345·7 to 629·0)
		Moldova	31 188 (27 896 to 34 408)	15 705 (14 143 to 17 419)	7381·1 (6602·0 to 8143·2)	6983·4 (6288·5 to 7745·5)	2492 (1783 to 3412)	1255 (902 to 1708)	589·8 (422·0 to 807·5)	557·9 (400·9 to 759·6)
		Russia	839 843 (771 258 to 913 707)	658 551 (600 607 to 713 930)	7475·2 (6864·7 to 8132·6)	7022·8 (6404·9 to 7613·4)	57 189 (41 624 to 77 337)	46 494 (33 036 to 62 324)	509·0 (370·5 to 688·4)	495·8 (352·3 to 664·6)
		Ukraine	232 355 (211 587 to 255 186)	155 202 (141 023 to 170 435)	6557·9 (5971·8 to 7202·3)	6319·4 (5742·1 to 6939·6)	17 604 (12 580 to 24 031)	11 912 (8587 to 15 985)	496·9 (355·0 to 678·2)	485·0 (349·6 to 650·9)
	Central Europe		707 126 (646 747 to 774 641)	385 463 (350 258 to 419 432)	7834·4 (7165·5 to 8582·4)	6898·9 (6268·9 to 7506·9)	44 850 (33 058 to 59 520)	24 733 (18 236 to 32 588)	496·9 (366·2 to 659·4)	442·7 (326·4 to 583·3)
		Albania	32 804 (28 470 to 37 890)	13 095 (11 370 to 15 025)	8125·6 (7052·2 to 9385·5)	7066·8 (6136·0 to 8108·6)	2214 (1502 to 3051)	909 (632 to 1268)	548·4 (372·1 to 755·8)	490·7 (341·2 to 684·4)
		Bosnia and Herzegovina	34 545 (31 866 to 37 407)	12 961 (11 791 to 14 095)	9259·4 (8541·2 to 10 026·4)	7584·3 (6899·7 to 8247·9)	2193 (1576 to 2973)	843 (603 to 1140)	587·8 (422·5 to 796·9)	493·4 (352·8 to 667·1)
		Bulgaria	39 074 (34 297 to 45 083)	21 397 (18 735 to 24 238)	6921·4 (6075·3 to 7985·8)	6432·8 (5632·5 to 7286·8)	2574 (1814 to 3570)	1473 (1018 to 2026)	456·0 (321·4 to 632·3)	442·8 (305·9 to 609·0)
		Croatia	18 572 (16 806 to 20 509)	12 189 (11 039 to 13 435)	6335·4 (5732·9 to 6996·2)	6195·4 (5610·9 to 6828·6)	1347 (930 to 1857)	944 (682 to 1264)	459·5 (317·2 to 633·5)	479·9 (346·5 to 642·6)
		Czech Republic	37 910 (34 006 to 42 239)	30 270 (26 929 to 33 661)	5808·1 (5210·1 to 6471·4)	5556·6 (4943·2 to 6178·9)	2671 (1854 to 3733)	2187 (1505 to 3035)	409·3 (284·1 to 571·9)	401·4 (276·2 to 557·1)
		Hungary	38 486 (34 855 to 42 489)	26 418 (23 875 to 29 113)	6241·1 (5652·4 to 6890·4)	5836·6 (5274·7 to 6431·9)	2781 (1928 to 3785)	1904 (1343 to 2613)	450·9 (312·6 to 613·8)	420·6 (296·7 to 577·4)
		Macedonia	12 355 (10 791 to 14 104)	7497 (6610 to 8472)	7212·6 (6299·7 to 8233·8)	6680·1 (5889·9 to 7548·6)	805 (562 to 1112)	517 (365 to 710)	469·7 (327·9 to 649·4)	460·9 (325·3 to 632·5)
		Montenegro	3557 (3118 to 4034)	2402 (2104 to 2717)	7021·9 (6156·1 to 7964·4)	6593·9 (5775·6 to 7460·3)	244 (173 to 334)	165 (118 to 227)	481·2 (341·2 to 660·3)	453·4 (324·2 to 624·5)
		Poland	278 364 (257 877 to 297 823)	151 235 (139 438 to 163 911)	9639·5 (8930·1 to 10 313·3)	8090·1 (7459·1 to 8768·2)	15 028 (10 817 to 20 408)	7944 (5704 to 10 904)	520·4 (374·6 to 706·7)	424·9 (305·1 to 583·3)
		Romania	126 967 (111 424 to 145 093)	56 716 (49 641 to 63 981)	7156·0 (6280·0 to 8177·6)	6456·9 (5651·5 to 7284·0)	8665 (6065 to 11 896)	3991 (2808 to 5422)	488·4 (341·8 to 670·5)	454·4 (319·7 to 617·2)
		Serbia	52 480 (46 083 to 60 358)	28 830 (25 212 to 32 744)	7430·3 (6524·5 to 8545·8)	6968·6 (6094·0 to 7914·6)	3904 (2813 to 5433)	2111 (1476 to 2957)	552·8 (398·3 to 769·2)	510·3 (356·7 to 714·7)
		Slovakia	24 732 (22 274 to 27 395)	16 215 (14 602 to 17 958)	5997·2 (5401·1 to 6642·7)	5624·3 (5064·5 to 6228·9)	1875 (1299 to 2547)	1266 (894 to 1748)	454·7 (314·9 to 617·7)	439·2 (310·1 to 606·1)
		Slovenia	7282 (6584 to 7981)	6238 (5610 to 6913)	6097·5 (5512·9 to 6683·0)	5891·5 (5298·3 to 6529·7)	548 (388 to 755)	478 (335 to 656)	458·9 (324·9 to 632·3)	451·2 (316·1 to 619·2)
	Central Asia		785 920 (726 282 to 854 179)	741 440 (679 574 to 806 244)	8375·6 (7740·1 to 9103·1)	7685·6 (7044·3 to 8357·4)	51 669 (37 726 to 68 079)	51 932 (38 573 to 68 672)	550·6 (402·1 to 725·5)	538·3 (399·8 to 711·8)
		Armenia	33 654 (30 876 to 36 726)	16 569 (15 055 to 18 233)	8272·8 (7589·9 to 9028·1)	7484·9 (6800·8 to 8236·5)	2424 (1720 to 3243)	1224 (870 to 1676)	595·9 (422·9 to 797·2)	552·9 (392·8 to 757·3)
		Azerbaijan	69 516 (63 325 to 75 721)	69 980 (63 098 to 76 830)	7919·5 (7214·2 to 8626·4)	7314·6 (6595·2 to 8030·5)	4634 (3146 to 6489)	4840 (3422 to 7101)	527·9 (358·4 to 739·3)	505·9 (357·7 to 742·2)
		Georgia	35 727 (32 456 to 39 154)	24 963 (22 724 to 27 408)	7788·4 (7075·2 to 8535·3)	7491·8 (6820·0 to 8225·7)	2513 (1743 to 3521)	1784 (1259 to 2454)	547·8 (380·0 to 767·6)	535·4 (377·8 to 736·4)
		Kazakhstan	152 581 (139 324 to 167 458)	142 514 (129 368 to 156 165)	7802·1 (7124·2 to 8562·8)	7274·3 (6603·3 to 7971·1)	10 433 (7399 to 14 231)	10 201 (7098 to 13 965)	533·5 (378·3 to 727·7)	520·7 (362·3 to 712·8)
		Kyrgyzstan	51 009 (46 794 to 55 652)	60 985 (55 974 to 66 579)	8334·9 (7646·1 to 9093·5)	8156·6 (7486·4 to 8904·9)	3328 (2305 to 4549)	4149 (2956 to 5551)	543·8 (376·7 to 743·2)	554·9 (395·3 to 742·4)
		Mongolia	28 311 (25 911 to 30 728)	28 790 (26 282 to 31 543)	8665·2 (7930·6 to 9405·1)	7732·6 (7059·1 to 8472·1)	1804 (1284 to 2461)	2037 (1433 to 2754)	552·1 (393·1 to 753·1)	547·1 (385·0 to 739·7)
		Tajikistan	78 566 (71 907 to 85 153)	97 076 (88 722 to 105 534)	8923·9 (8167·6 to 9672·1)	8526·6 (7792·9 to 9269·5)	5202 (3604 to 7120)	6614 (4732 to 8950)	590·9 (409·4 to 808·8)	580·9 (415·7 to 786·2)
		Turkmenistan	46 283 (42 491 to 50 360)	42 036 (38 241 to 46 072)	8380·4 (7693·9 to 9118·7)	7278·5 (6621·4 to 7977·4)	2956 (2038 to 4060)	2992 (2081 to 4132)	535·2 (369·0 to 735·2)	518·1 (360·4 to 715·4)
		Uzbekistan	290 274 (266 883 to 315 185)	258 528 (236 454 to 281 738)	8761·4 (8055·4 to 9513·3)	7738·8 (7078·0 to 8433·5)	18 376 (13 188 to 24 661)	18 091 (12 852 to 25 026)	554·6 (398·1 to 744·4)	541·5 (384·7 to 749·1)
Latin America and Caribbean			4 426 619 (4 093 548 to 4 772 219)	3 829 678 (3 531 041 to 4 141 135)	8474·1 (7836·5 to 9135·7)	7720·4 (7118·3 to 8348·2)	286 888 (210 794 to 375 808)	258 663 (189 348 to 341 342)	549·2 (403·5 to 719·4)	521·5 (381·7 to 688·1)
	Central Latin America		1 949 847 (1 795 021 to 2 105 618)	1 680 405 (1 541 412 to 1 827 754)	8196·6 (7545·8 to 8851·4)	7356·1 (6747·6 to 8001·1)	133 143 (97 830 to 175 882)	116 653 (83 529 to 154 377)	559·7 (411·2 to 739·4)	510·7 (365·6 to 675·8)
		Colombia	389 470 (358 680 to 422 348)	276 089 (250 661 to 301 863)	9109·8 (8389·6 to 9878·8)	7726·4 (7014·8 to 8447·7)	24 339 (16 420 to 34 936)	18 069 (12 251 to 26 037)	569·3 (384·1 to 817·1)	505·7 (342·8 to 728·6)
		Costa Rica	32 172 (28 996 to 35 408)	21 871 (19 805 to 23 887)	8086·5 (7288·1 to 8899·8)	7189·6 (6510·6 to 7852·4)	2065 (1429 to 2866)	1430 (986 to 2026)	519·0 (359·1 to 720·3)	470·2 (324·2 to 665·9)
		El Salvador	66 584 (60 262 to 72 894)	42 046 (38 229 to 46 043)	9208·2 (8333·9 to 10 080·9)	7874·9 (7159·9 to 8623·5)	4306 (2983 to 6083)	2804 (1926 to 3896)	595·5 (412·5 to 841·3)	525·2 (360·7 to 729·8)
		Guatemala	143 257 (132 086 to 154 923)	149 140 (135 999 to 162 702)	9219·1 (8500·2 to 9969·8)	7564·0 (6897·5 to 8251·8)	8888 (6086 to 12 126)	10 171 (7130 to 14 023)	572·0 (391·7 to 780·3)	515·9 (361·6 to 711·2)
		Honduras	80 019 (72 798 to 87 302)	76 709 (69 837 to 83 521)	9545·6 (8684·1 to 10 414·4)	8099·2 (7373·6 to 8818·4)	4952 (3461 to 6822)	5146 (3531 to 7055)	590·8 (412·9 to 813·8)	543·4 (372·8 to 744·9)
		Mexico	963 139 (883 996 to 1 042 522)	838 518 (770 385 to 909 551)	7662·1 (7032·5 to 8293·7)	7184·4 (6600·6 to 7793·0)	70 248 (50 700 to 92 613)	59 982 (43 124 to 80 105)	558·9 (403·3 to 736·8)	513·9 (369·5 to 686·3)
		Nicaragua	50 769 (46 140 to 55 551)	49 095 (44 927 to 53 579)	9105·9 (8275·7 to 9963·6)	8059·8 (7375·5 to 8795·7)	3267 (2243 to 4566)	3248 (2206 to 4462)	586·0 (402·4 to 819·0)	533·2 (362·2 to 732·5)
		Panama	22 704 (20 518 to 25 047)	24 542 (22 266 to 26 910)	7932·5 (7168·6 to 8751·0)	7078·1 (6421·6 to 7761·0)	1470 (1020 to 2018)	1621 (1127 to 2292)	513·7 (356·3 to 705·1)	467·5 (324·9 to 661·0)
		Venezuela	201 733 (183 072 to 221 415)	202 395 (184 229 to 222 580)	7800·5 (7079·0 to 8561·6)	7012·3 (6383·0 to 7711·7)	13 606 (9393 to 19 115)	14 181 (9546 to 20 012)	526·1 (363·2 to 739·1)	491·3 (330·7 to 693·4)
	Andean Latin America		532 549 (492 891 to 573 086)	551 364 (507 353 to 597 484)	9675·5 (8955·0 to 10 412·0)	8275·3 (7614·8 to 8967·5)	32 088 (22 912 to 43 283)	37 231 (26 702 to 50 922)	583·0 (416·3 to 786·4)	558·8 (400·8 to 764·3)
		Bolivia	115 065 (106 475 to 123 844)	116 804 (107 554 to 126 915)	10 513·3 (9728·5 to 11 315·5)	8534·6 (7858·7 to 9273·3)	6457 (4489 to 8782)	7368 (5152 to 10 168)	590·0 (410·2 to 802·4)	538·4 (376·5 to 743·0)
		Ecuador	143 491 (132 895 to 154 725)	142 712 (131 295 to 155 221)	9413·8 (8718·7 to 10 150·9)	7979·4 (7341·1 to 8678·9)	8481 (5976 to 11 729)	9015 (6197 to 12 444)	556·4 (392·0 to 769·5)	504·0 (346·5 to 695·8)
		Peru	273 993 (252 646 to 298 031)	291 847 (266 744 to 318 506)	9495·9 (8756·1 to 10 329·0)	8325·1 (7609·0 to 9085·5)	17 149 (11 571 to 23 634)	20 848 (14 493 to 29 571)	594·4 (401·0 to 819·1)	594·7 (413·4 to 843·5)
	Caribbean		350 473 (323 853 to 377 937)	309 142 (284 012 to 335 926)	8398·0 (7760·1 to 9056·1)	7748·8 (7118·9 to 8420·2)	24 874 (17 770 to 34 244)	24 965 (17 514 to 37 326)	596·0 (425·8 to 820·6)	625·8 (439·0 to 935·6)
		Antigua and Barbuda	391 (359 to 423)	391 (357 to 425)	6988·5 (6419·4 to 7573·4)	6401·8 (5843·0 to 6971·8)	27 (19 to 37)	29 (20 to 41)	487·5 (339·7 to 657·9)	481·5 (335·7 to 672·3)
		The Bahamas	1924 (1764 to 2101)	2121 (1931 to 2313)	6865·6 (6292·2 to 7494·6)	6426·9 (5850·7 to 7008·5)	157 (109 to 227)	172 (117 to 248)	560·7 (387·8 to 811·6)	520·6 (355·1 to 752·8)
		Barbados	1315 (1206 to 1436)	920 (836 to 1021)	6471·1 (5939·0 to 7068·4)	6218·9 (5650·8 to 6907·1)	108 (74 to 158)	87 (58 to 135)	529·9 (366·6 to 778·1)	591·5 (392·2 to 910·1)
		Belize	3217 (2977 to 3469)	3480 (3200 to 3792)	8737·6 (8085·6 to 9422·6)	7325·1 (6736·4 to 7982·0)	232 (162 to 332)	287 (193 to 419)	631·0 (439·2 to 901·8)	604·2 (405·5 to 883·0)
		Bermuda	256 (236 to 278)	264 (241 to 286)	6560·1 (6034·8 to 7104·5)	6019·4 (5487·7 to 6526·0)	17 (12 to 24)	17 (12 to 23)	431·7 (306·3 to 605·7)	380·3 (263·2 to 524·8)
		Cuba	65 833 (60 125 to 71 640)	40 223 (36 540 to 43 686)	7363·9 (6725·4 to 8013·4)	6709·3 (6094·9 to 7286·9)	4789 (3335 to 6689)	2754 (1934 to 3784)	535·7 (373·0 to 748·3)	459·4 (322·5 to 631·2)
		Dominica	659 (607 to 714)	372 (336 to 414)	7679·2 (7073·7 to 8312·1)	7000·6 (6308·2 to 7791·4)	50 (35 to 73)	36 (23 to 58)	582·4 (402·7 to 844·9)	676·1 (441·6 to 1093·3)
		Dominican Republic	76 881 (70 495 to 83 979)	72 107 (65 143 to 80 761)	8567·6 (7855·9 to 9358·6)	7661·3 (6921·3 to 8580·7)	6066 (3924 to 9076)	7130 (4614 to 11 452)	676·0 (437·3 to 1011·5)	757·5 (490·3 to 1216·8)
		Grenada	1226 (1132 to 1319)	609 (557 to 664)	8391·9 (7746·9 to 9031·2)	7001·1 (6406·5 to 7633·9)	88 (61 to 122)	53 (36 to 76)	602·3 (419·8 to 834·1)	604·7 (418·3 to 875·6)
		Guyana	8831 (8111 to 9594)	4887 (4455 to 5386)	8400·0 (7715·6 to 9125·6)	7478·8 (6817·9 to 8242·8)	703 (483 to 1014)	458 (306 to 702)	668·7 (459·0 to 964·8)	700·3 (468·8 to 1074·3)
		Haiti	115 309 (107 283 to 123 280)	132 719 (122 172 to 144 055)	10 011·0 (9314·2 to 10 703·0)	8735·1 (8041·0 to 9481·2)	6910 (4794 to 9375)	9625 (6558 to 14 386)	599·9 (416·2 to 814·0)	633·5 (431·6 to 946·8)
		Jamaica	25 892 (23 833 to 28 092)	19 567 (17 919 to 21 514)	7681·6 (7070·5 to 8334·2)	7072·2 (6476·9 to 7776·0)	2030 (1428 to 2873)	1722 (1179 to 2550)	602·2 (423·8 to 852·2)	622·5 (426·2 to 921·7)
		Puerto Rico	20 761 (19 038 to 22 597)	12 758 (11 620 to 14 065)	6634·8 (6084·1 to 7221·5)	6087·5 (5544·4 to 6711·0)	1573 (1096 to 2210)	1023 (710 to 1453)	502·6 (350·1 to 706·1)	488·0 (338·6 to 693·5)
		Saint Lucia	1629 (1503 to 1758)	673 (614 to 741)	7914·3 (7301·8 to 8540·6)	6991·2 (6371·6 to 7693·7)	118 (82 to 165)	59 (40 to 86)	573·6 (400·5 to 799·9)	608·4 (420·2 to 895·6)
		Saint Vincent and the Grenadines	1252 (1159 to 1348)	662 (605 to 725)	8051·2 (7455·4 to 8665·8)	7243·1 (6621·3 to 7925·9)	78 (56 to 106)	58 (40 to 87)	504·1 (358·4 to 679·9)	635·3 (439·5 to 946·3)
		Suriname	4495 (4134 to 4890)	3348 (3030 to 3743)	7982·5 (7342·1 to 8684·0)	7206·9 (6522·6 to 8056·6)	376 (257 to 556)	322 (207 to 518)	668·5 (456·6 to 987·5)	692·7 (446·2 to 1115·0)
		Trinidad and Tobago	10 493 (9667 to 11 403)	4535 (4157 to 4944)	7401·2 (6818·8 to 8043·1)	6583·8 (6034·7 to 7176·2)	832 (583 to 1176)	367 (259 to 535)	586·6 (411·1 to 829·7)	532·5 (376·3 to 776·5)
		Virgin Islands	719 (658 to 785)	333 (304 to 365)	6513·6 (5958·4 to 7108·4)	6027·4 (5512·8 to 6603·7)	53 (38 to 74)	27 (18 to 38)	482·8 (341·9 to 670·8)	484·7 (333·9 to 686·9)
	Tropical Latin America		1 593 751 (1 471 390 to 1 721 800)	1 288 768 (1 190 481 to 1 396 313)	8490·4 (7838·6 to 9172·6)	8000·4 (7390·2 to 8668·0)	96 784 (70 793 to 129 045)	79 814 (59 666 to 105 314)	515·6 (377·1 to 687·5)	495·5 (370·4 to 653·8)
		Brazil	1 531 450 (1 412 900 to 1 655 138)	1 238 091 (1 144 498 to 1 341 133)	8442·9 (7789·3 to 9124·8)	7999·0 (7394·3 to 8664·7)	93 146 (68 058 to 124 025)	76 604 (57 163 to 101 031)	513·5 (375·2 to 683·8)	494·9 (369·3 to 652·7)
		Paraguay	62 301 (57 275 to 67 614)	50 677 (46 195 to 55 186)	9854·9 (9059·8 to 10 695·3)	8034·3 (7323·8 to 8749·1)	3638 (2551 to 4982)	3210 (2280 to 4321)	575·4 (403·4 to 788·1)	508·9 (361·5 to 685·0)
Southeast Asia, east Asia, and Oceania			13 126 413 (12 207 927 to 14 051 448)	8 601 788 (7 928 118 to 9 288 537)	7820·4 (7273·2 to 8371·5)	6989·2 (6441·9 to 7547·3)	873 879 (648 712 to 1 140 335)	586 929 (436 091 to 772 852)	520·6 (386·5 to 679·4)	476·9 (354·3 to 628·0)
	East Asia		8 343 699 (7 771 751 to 8 909 781)	4 337 471 (3 990 626 to 4 681 742)	7421·5 (6912·8 to 7925·0)	6711·1 (6174·5 to 7243·8)	533 524 (396 708 to 704 333)	270 007 (199 649 to 355 126)	474·6 (352·9 to 626·5)	417·8 (308·9 to 549·5)
		China	8 023 886 (7 466 111 to 8 569 138)	4 036 176 (3 706 050 to 4 356 704)	7439·3 (6922·1 to 7944·8)	6654·0 (6109·8 to 7182·4)	513 291 (382 292 to 677 473)	251 325 (186 080 to 331 226)	475·9 (354·4 to 628·1)	414·3 (306·8 to 546·0)
		North Korea	217 143 (200 110 to 234 426)	241 183 (223 774 to 258 478)	7288·4 (6716·7 to 7868·5)	8150·9 (7562·5 to 8735·3)	13 483 (9643 to 18 162)	14 798 (10 429 to 19 852)	452·6 (323·7 to 609·6)	500·1 (352·4 to 670·9)
		Taiwan	102 670 (94 323 to 110 674)	60 111 (54 879 to 65 016)	6464·9 (5939·3 to 6968·9)	5927·7 (5411·8 to 6411·4)	6750 (4621 to 9214)	3885 (2721 to 5375)	425·0 (291·0 to 580·2)	383·1 (268·3 to 530·0)
	Southeast Asia		4 689 433 (4 325 383 to 5 089 350)	4 145 551 (3 814 810 to 4 492 125)	8621·4 (7952·1 to 9356·6)	7269·4 (6689·4 to 7877·1)	334 448 (247 161 to 433 354)	309 202 (229 321 to 408 292)	614·9 (454·4 to 796·7)	542·2 (402·1 to 716·0)
		Cambodia	126 502 (110 132 to 145 378)	148 013 (128 331 to 169 316)	10 279·6 (8949·4 to 11 813·5)	7857·2 (6812·3 to 8988·0)	8847 (6112 to 12 120)	11 443 (7809 to 15 681)	718·9 (496·7 to 984·8)	607·5 (414·5 to 832·4)
		Indonesia	1 710 437 (1 579 352 to 1 853 498)	1 707 335 (1 574 318 to 1 843 556)	8325·0 (7687·0 to 9021·3)	7512·6 (6927·3 to 8111·9)	117 832 (86 814 to 154 745)	124 155 (92 159 to 163 904)	573·5 (422·5 to 753·2)	546·3 (405·5 to 721·2)
		Laos	49 214 (44 142 to 54 817)	65 585 (58 853 to 73 307)	7567·0 (6787·1 to 8428·5)	5802·4 (5206·8 to 6485·5)	3815 (2629 to 5276)	5665 (3948 to 7789)	586·5 (404·2 to 811·2)	501·2 (349·3 to 689·1)
		Malaysia	142 813 (127 428 to 159 047)	121 304 (107 620 to 136 955)	5842·1 (5212·8 to 6506·2)	4724·3 (4191·4 to 5333·9)	12 101 (8129 to 16 913)	10 809 (7356 to 15 785)	495·0 (332·5 to 691·9)	421·0 (286·5 to 614·8)
		Maldives	1705 (1478 to 1973)	2007 (1754 to 2274)	8696·8 (7537·2 to 10 061·5)	6404·1 (5596·9 to 7255·1)	131 (90 to 178)	150 (104 to 207)	666·1 (461·4 to 908·6)	477·0 (332·4 to 660·1)
		Mauritius	7554 (6781 to 8458)	4054 (3626 to 4521)	7441·8 (6680·5 to 8332·9)	6128·3 (5480·6 to 6833·4)	593 (410 to 798)	341 (226 to 483)	584·5 (403·7 to 786·4)	515·3 (341·8 to 730·7)
		Myanmar	489 593 (442 136 to 542 953)	351 772 (316 701 to 391 686)	10 075·7 (9099·0 to 11 173·8)	7552·7 (6799·7 to 8409·7)	41 365 (28 836 to 54 880)	31 317 (21 788 to 42 909)	851·3 (593·4 to 1129·4)	672·4 (467·8 to 921·3)
		Philippines	965 939 (892 461 to 1 041 933)	1 014 332 (935 653 to 1 100 860)	10 947·8 (10 115·0 to 11 809·1)	8769·0 (8088·8 to 9517·0)	53 972 (37 766 to 73 071)	63 694 (43 075 to 88 331)	611·7 (428·0 to 828·2)	550·6 (372·4 to 763·6)
		Sri Lanka	167 170 (150 999 to 185 487)	110 308 (99 796 to 122 614)	9380·8 (8473·4 to 10 408·7)	7298·0 (6602·5 to 8112·2)	12 536 (8886 to 16 936)	8919 (6056 to 12 084)	703·5 (498·6 to 950·4)	590·1 (400·7 to 799·5)
		Seychelles	596 (515 to 686)	515 (446 to 587)	7278·6 (6290·9 to 8379·4)	6163·8 (5335·9 to 7027·3)	46 (32 to 64)	42 (29 to 59)	566·0 (389·6 to 777·7)	505·5 (348·5 to 706·3)
		Thailand	454 321 (400 968 to 515 738)	188 135 (164 469 to 211 217)	7797·1 (6881·5 to 8851·2)	6081·0 (5316·1 to 6827·1)	38 022 (27 475 to 50 801)	16 331 (11 216 to 22 342)	652·5 (471·5 to 871·9)	527·9 (362·5 to 722·1)
		Timor-Leste	11 497 (10 022 to 13 149)	13 017 (11 405 to 14 749)	10 435·8 (9097·3 to 11 935·0)	7863·8 (6890·2 to 8909·9)	777 (546 to 1055)	957 (647 to 1305)	705·0 (495·6 to 957·9)	578·3 (391·1 to 788·4)
		Vietnam	556 292 (509 007 to 606 437)	414 499 (375 155 to 456 573)	7020·8 (6424·1 to 7653·7)	5487·4 (4966·6 to 6044·4)	43 998 (31 373 to 58 909)	35 030 (25 049 to 47 369)	555·3 (395·9 to 743·5)	463·8 (331·6 to 627·1)
	Oceania		93 280 (86 091 to 101 233)	118 767 (109 518 to 128 135)	9056·6 (8358·5 to 9828·8)	8405·8 (7751·2 to 9068·8)	5907 (4265 to 7850)	7719 (5560 to 10 296)	573·5 (414·1 to 762·1)	546·3 (393·5 to 728·7)
		American Samoa	526 (481 to 571)	471 (429 to 515)	6742·4 (6165·9 to 7321·0)	6311·0 (5747·8 to 6896·1)	35 (25 to 48)	33 (23 to 44)	450·5 (321·5 to 614·5)	436·7 (307·9 to 586·1)
		Federated States of Micronesia	1414 (1303 to 1532)	684 (627 to 743)	8099·4 (7463·3 to 8775·6)	7150·6 (6549·7 to 7765·9)	97 (70 to 130)	48 (35 to 65)	554·8 (402·4 to 746·6)	501·6 (360·5 to 674·3)
		Fiji	6539 (5939 to 7128)	3350 (3054 to 3674)	7201·4 (6540·4 to 7850·2)	6590·2 (6007·2 to 7227·2)	460 (330 to 621)	248 (178 to 334)	506·9 (363·5 to 683·5)	487·8 (349·3 to 657·8)
		Guam	1017 (921 to 1109)	982 (891 to 1081)	6057·5 (5481·2 to 6605·5)	5866·2 (5318·0 to 6452·3)	69 (48 to 93)	68 (49 to 93)	407·8 (286·7 to 554·0)	408·5 (289·6 to 556·6)
		Kiribati	1267 (1176 to 1368)	1111 (1021 to 1203)	9047·2 (8393·6 to 9768·8)	8220·9 (7553·9 to 8898·9)	82 (59 to 108)	74 (53 to 101)	582·9 (418·2 to 773·9)	551·1 (392·5 to 747·8)
		Marshall Islands	570 (524 to 617)	722 (663 to 784)	7850·8 (7219·3 to 8503·6)	7282·9 (6694·6 to 7911·5)	39 (28 to 52)	51 (36 to 69)	532·4 (379·5 to 712·6)	510·2 (361·6 to 693·1)
		Northern Mariana Islands	446 (407 to 490)	928 (838 to 1015)	6159·4 (5614·9 to 6765·7)	6081·7 (5493·5 to 6654·1)	30 (21 to 40)	64 (45 to 87)	409·2 (287·5 to 556·4)	418·0 (297·4 to 570·0)
		Papua New Guinea	64 742 (59 789 to 70 468)	93 611 (86 224 to 101 023)	9570·9 (8838·7 to 10 417·5)	8687·0 (8001·5 to 9374·8)	4005 (2845 to 5360)	5995 (4236 to 8075)	592·1 (420·5 to 792·4)	556·3 (393·1 to 749·3)
		Samoa	2228 (2046 to 2417)	2013 (1851 to 2180)	7944·5 (7297·1 to 8617·3)	7327·2 (6734·9 to 7933·6)	150 (107 to 204)	137 (98 to 184)	535·3 (382·4 to 728·0)	497·4 (354·9 to 668·1)
		Solomon Islands	4687 (4320 to 5086)	6869 (6319 to 7437)	9082·2 (8371·4 to 9856·8)	8243·0 (7583·4 to 8925·2)	306 (221 to 406)	467 (340 to 624)	592·7 (428·8 to 787·6)	560·1 (408·0 to 749·0)
		Tonga	1215 (1119 to 1308)	933 (857 to 1012)	7539·7 (6947·2 to 8117·9)	6930·5 (6369·4 to 7521·9)	84 (61 to 111)	65 (46 to 87)	521·2 (376·4 to 688·8)	486·1 (345·2 to 645·6)
		Vanuatu	2504 (2316 to 2705)	3149 (2912 to 3399)	8644·5 (7994·7 to 9335·3)	7761·4 (7178·2 to 8376·6)	163 (117 to 218)	213 (153 to 283)	563·1 (403·9 to 752·7)	526·1 (377·9 to 698·4)
North Africa and Middle East			5 546 439 (5 036 430 to 6 143 208)	5 967 288 (5 405 745 to 6 595 230)	11 096·8 (10 076·4 to 12 290·7)	9443·3 (8554·6 to 10 437·0)	425 298 (303 410 to 609 338)	478 842 (341 007 to 688 948)	850·9 (607·0 to 1219·1)	757·8 (539·6 to 1090·3)
	Afghanistan		307 202 (275 997 to 343 207)	590 050 (528 905 to 656 325)	14 008·9 (12 585·9 to 15 650·8)	11 902·7 (10 669·3 to 13 239·6)	20 208 (13 975 to 28 736)	42 063 (28 995 to 60 519)	921·5 (637·3 to 1310·4)	848·5 (584·9 to 1220·8)
	Algeria		420 937 (370 443 to 485 787)	404 974 (356 384 to 464 757)	10 790·5 (9496·1 to 12 452·9)	8901·3 (7833·3 to 10 215·3)	33 454 (22 851 to 50 114)	33 708 (22 761 to 49 151)	857·6 (585·8 to 1284·6)	740·9 (500·3 to 1080·3)
	Bahrain		6649 (5811 to 7797)	8214 (7215 to 9455)	9740·5 (8512·0 to 11 421·3)	8238·5 (7236·3 to 9483·6)	507 (344 to 730)	612 (417 to 857)	743·4 (503·8 to 1069·3)	613·4 (418·4 to 859·6)
	Egypt		1 003 443 (903 584 to 1 122 301)	1 116 287 (1 003 395 to 1 240 846)	12 674·5 (11 413·2 to 14 175·8)	10 235·1 (9200·0 to 11 377·1)	71 848 (49 544 to 106 070)	89 515 (60 751 to 132 237)	907·5 (625·8 to 1339·8)	820·8 (557·0 to 1212·5)
	Iran		882 136 (794 852 to 976 707)	600 999 (538 440 to 665 241)	9344·8 (8420·2 to 10 346·6)	7452·2 (6676·5 to 8248·8)	73 873 (50 383 to 109 417)	52 838 (36 217 to 76 262)	782·6 (533·7 to 1159·1)	655·2 (449·1 to 945·6)
	Iraq		356 856 (312 665 to 423 819)	797 308 (706 812 to 932 363)	11 660·3 (10 216·4 to 13 848·4)	10 347·9 (9173·4 to 12 100·8)	31 437 (20 964 to 54 253)	70 013 (46 960 to 123 252)	1027·2 (685·0 to 1772·7)	908·7 (609·5 to 1599·6)
	Jordan		54 356 (47 845 to 62 345)	82 497 (72 654 to 94 275)	10 495·9 (9238·7 to 12 038·5)	8591·1 (7566·1 to 9817·7)	3858 (2777 to 5258)	6218 (4347 to 8639)	745·0 (536·2 to 1015·2)	647·6 (452·7 to 899·6)
	Kuwait		22 004 (19 251 to 25 680)	21 641 (19 145 to 25 021)	9204·5 (8052·7 to 10 742·2)	7678·4 (6792·8 to 8877·9)	1538 (1065 to 2140)	1563 (1079 to 2150)	643·2 (445·3 to 895·4)	554·5 (382·9 to 763·0)
	Lebanon		32 597 (29 655 to 36 138)	24 181 (21 691 to 26 929)	9032·8 (8217·4 to 10 013·9)	7537·4 (6761·4 to 8393·9)	2726 (1893 to 4171)	1994 (1382 to 2867)	755·5 (524·5 to 1155·7)	621·4 (430·9 to 893·7)
	Libya		47 266 (42 662 to 52 423)	36 769 (33 156 to 40 727)	9236·9 (8337·1 to 10 244·7)	7941·8 (7161·5 to 8796·7)	3885 (2701 to 5663)	3146 (2199 to 4627)	759·2 (527·9 to 1106·6)	679·6 (474·9 to 999·5)
	Morocco		389 914 (355 200 to 427 334)	204 052 (185 612 to 224 460)	11 299·3 (10 293·3 to 12 383·7)	8905·2 (8100·4 to 9795·9)	30 035 (21 047 to 44 210)	16 853 (11 407 to 25 521)	870·4 (609·9 to 1281·2)	735·5 (497·8 to 1113·8)
	Palestine		40 687 (36 448 to 45 410)	108 607 (97 667 to 121 111)	10 701·2 (9586·3 to 11 943·4)	10 006·0 (8998·1 to 11 158·0)	3374 (2262 to 5303)	9031 (6114 to 14 141)	887·5 (594·8 to 1394·7)	832·1 (563·3 to 1302·8)
	Oman		40 313 (35 704 to 46 251)	34 938 (30 573 to 40 136)	12 025·1 (10 650·5 to 13 796·5)	7880·0 (6895·6 to 9052·3)	2281 (1560 to 3099)	2338 (1600 to 3330)	680·4 (465·2 to 924·4)	527·4 (360·8 to 751·0)
	Qatar		4459 (4015 to 4904)	9090 (8199 to 10 057)	8652·8 (7790·6 to 9517·1)	7342·1 (6622·3 to 8123·3)	352 (240 to 484)	669 (456 to 949)	682·6 (466·2 to 939·9)	540·6 (368·3 to 766·2)
	Saudi Arabia		333 444 (303 930 to 372 714)	200 029 (181 195 to 223 507)	11 224·1 (10 230·7 to 12 546·0)	8026·6 (7270·9 to 8968·7)	26 820 (18 150 to 42 504)	16 880 (11 928 to 25 010)	902·8 (611·0 to 1430·7)	677·3 (478·6 to 1003·6)
	Sudan		321 859 (286 550 to 371 817)	420 767 (370 723 to 480 367)	12 879·4 (11 466·5 to 14 878·5)	10 034·0 (8840·6 to 11 455·2)	22 244 (15 662 to 31 777)	32 075 (21 575 to 47 304)	890·1 (626·7 to 1271·6)	764·9 (514·5 to 1128·1)
	Syria		220 906 (194 425 to 260 245)	151 805 (133 967 to 175 890)	10 682·9 (9402·3 to 12 585·3)	8591·1 (7581·6 to 9954·2)	18 572 (12 812 to 28 116)	12 175 (8274 to 17 774)	898·1 (619·6 to 1359·7)	689·0 (468·2 to 1005·9)
	Tunisia		102 039 (92 458 to 114 135)	64 352 (58 266 to 71 565)	9444·7 (8557·9 to 10 564·3)	7712·1 (6982·7 to 8576·5)	8570 (5793 to 13 887)	5566 (3793 to 8473)	793·2 (536·2 to 1285·4)	667·1 (454·5 to 1015·4)
	Turkey		595 743 (547 707 to 644 596)	466 481 (425 080 to 508 587)	9201·4 (8459·5 to 9955·9)	7574·5 (6902·3 to 8258·2)	43 903 (29 627 to 60 860)	33 025 (22 695 to 47 573)	678·1 (457·6 to 940·0)	536·2 (368·5 to 772·5)
	United Arab Emirates		28 079 (24 917 to 32 195)	71 111 (63 533 to 81 618)	10 234·7 (9082·1 to 11 735·1)	8720·3 (7791·0 to 10 008·7)	1974 (1365 to 2820)	5046 (3423 to 7107)	719·4 (497·7 to 1028·0)	618·7 (419·8 to 871·5)
	Yemen		332 058 (301 679 to 362 938)	548 381 (495 306 to 600 159)	15 391·3 (13 983·2 to 16 822·7)	11 852·5 (10 705·3 to 12 971·6)	23 569 (16 460 to 31 745)	43 133 (31 011 to 58 699)	1092·5 (762·9 to 1471·4)	932·3 (670·2 to 1268·7)
South Asia			15 723 355 (14 388 260 to 17 095 151)	15 042 176 (13 732 348 to 16 346 221)	10 388·4 (9506·3 to 11 294·8)	9792·4 (8939·7 to 10 641·4)	1 158 310 (871 747 to 1 488 314)	1 111 008 (832 645 to 1 435 848)	765·3 (576·0 to 983·3)	723·3 (542·0 to 934·7)
	Bangladesh		1 573 129 (1 441 908 to 1 712 730)	1 315 169 (1 201 817 to 1 436 543)	10 770·5 (9872·1 to 11 726·3)	9178·1 (8387·1 to 10 025·1)	126 917 (91 041 to 167 567)	106 010 (77 499 to 139 377)	868·9 (623·3 to 1147·3)	739·8 (540·8 to 972·7)
	Bhutan		7593 (6858 to 8410)	5740 (5082 to 6471)	8855·2 (7998·0 to 9808·0)	7352·5 (6509·2 to 8289·2)	610 (438 to 810)	464 (339 to 627)	711·9 (510·4 to 944·5)	593·9 (434·5 to 803·6)
	India		12 051 742 (10 996 894 to 13 089 888)	11 560 118 (10 518 238 to 12 554 824)	10 524·7 (9603·5 to 11 431·3)	10 308·6 (9379·5 to 11 195·6)	870 624 (658 552 to 1 120 261)	828 693 (622 745 to 1 073 770)	760·3 (575·1 to 978·3)	739·0 (555·3 to 957·5)
	Nepal		285 403 (257 987 to 316 932)	312 510 (278 186 to 352 784)	9319·0 (8423·8 to 10 348·5)	7830·6 (6970·6 to 8839·8)	21 581 (15 935 to 28 502)	25 406 (18 342 to 34 268)	704·7 (520·3 to 930·6)	636·6 (459·6 to 858·6)
	Pakistan		1 805 489 (1 631 990 to 2 002 632)	1 848 638 (1 646 524 to 2 053 317)	9457·3 (8548·5 to 10 489·9)	8012·7 (7136·7 to 8899·9)	138 577 (100 072 to 186 838)	150 435 (109 051 to 202 581)	725·9 (524·2 to 978·7)	652·0 (472·7 to 878·1)
Sub-Saharan Africa			8 587 769 (7 857 220 to 9 325 853)	14 709 465 (13 425 142 to 16 030 223)	10 462·8 (9572·7 to 11 362·0)	9393·7 (8573·5 to 10 237·1)	604 496 (450 993 to 787 667)	1 155 230 (842 482 to 1 546 754)	736·5 (549·5 to 959·6)	737·8 (538·0 to 987·8)
	Southern sub-Saharan Africa		616 444 (572 289 to 660 577)	769 223 (712 345 to 828 232)	9410·3 (8736·3 to 10 084·0)	8935·9 (8275·1 to 9621·4)	46 467 (34 445 to 61 456)	59 316 (43 499 to 80 054)	709·3 (525·8 to 938·2)	689·1 (505·3 to 930·0)
		Botswana	17 818 (16 407 to 19 291)	21 217 (19 411 to 23 020)	9422·0 (8676·3 to 10 201·3)	8014·7 (7332·3 to 8695·8)	1259 (908 to 1661)	1603 (1143 to 2182)	665·6 (480·1 to 878·5)	605·7 (431·8 to 824·3)
		Lesotho	24 292 (22 251 to 26 383)	24 288 (22 166 to 26 554)	11 119·2 (10 185·0 to 12 076·4)	9405·9 (8584·0 to 10 283·1)	1584 (1144 to 2086)	1721 (1206 to 2297)	725·1 (523·5 to 954·9)	666·4 (467·1 to 889·5)
		Namibia	22 988 (21 235 to 24 742)	28 555 (26 178 to 30 983)	10 357·2 (9567·4 to 11 147·6)	8579·2 (7864·9 to 9308·6)	1548 (1100 to 2091)	2095 (1492 to 2857)	697·6 (495·7 to 942·2)	629·4 (448·1 to 858·4)
		South Africa	393 368 (366 445 to 420 789)	453 111 (421 280 to 484 391)	9406·6 (8762·8 to 10 062·3)	9042·0 (8406·8 to 9666·2)	30 863 (22 845 to 40 346)	35 607 (25 913 to 47 222)	738·0 (546·3 to 964·8)	710·6 (517·1 to 942·3)
		Swaziland	13 345 (12 357 to 14 353)	18 798 (17 310 to 20 351)	10 201·0 (9445·7 to 10 972·0)	9014·2 (8300·6 to 9758·8)	880 (628 to 1167)	1356 (948 to 1831)	673·0 (479·9 to 891·9)	650·1 (454·8 to 877·9)
		Zimbabwe	144 634 (132 669 to 156 845)	223 253 (202 949 to 244 627)	8991·8 (8247·9 to 9750·9)	8814·8 (8013·1 to 9658·7)	10 332 (7405 to 14 095)	16 933 (11 709 to 24 236)	642·3 (460·4 to 876·3)	668·6 (462·3 to 956·9)
	Western sub-Saharan Africa		3 238 338 (2 939 162 to 3 555 022)	5 610 365 (5 060 026 to 6 179 830)	9957·4 (9037·5 to 10 931·2)	8682·1 (7830·5 to 9563·4)	232 173 (171 915 to 304 141)	448 005 (325 011 to 601 220)	713·9 (528·6 to 935·2)	693·3 (503·0 to 930·4)
		Benin	90 652 (80 730 to 101 146)	177 242 (157 525 to 197 834)	10 660·9 (9494·0 to 11 894·9)	9276·1 (8244·2 to 10 353·8)	6746 (4703 to 9078)	14 937 (10 396 to 21 291)	793·4 (553·1 to 1067·6)	781·7 (544·1 to 1114·3)
		Burkina Faso	146 144 (132 148 to 160 264)	250 783 (225 669 to 276 009)	9037·1 (8171·6 to 9910·2)	7849·8 (7063·7 to 8639·4)	11 503 (8060 to 15 711)	21 608 (15 311 to 29 493)	711·3 (498·4 to 971·5)	676·4 (479·2 to 923·2)
		Cameroon	181 677 (162 175 to 204 786)	319 715 (281 862 to 361 089)	9061·1 (8088·4 to 10 213·7)	8246·0 (7269·7 to 9313·1)	13 991 (9786 to 19 506)	26 862 (18 680 to 39 088)	697·8 (488·1 to 972·8)	692·8 (481·8 to 1008·1)
		Cape Verde	5292 (4727 to 5916)	5501 (4882 to 6114)	9196·8 (8213·8 to 10 281·2)	7326·8 (6502·9 to 8142·9)	435 (304 to 596)	508 (352 to 727)	756·5 (527·8 to 1036·0)	676·7 (468·4 to 968·1)
		Chad	116 736 (107 648 to 125 883)	254 549 (232 903 to 276 285)	11 115·3 (10 250·0 to 11 986·3)	9636·5 (8817·0 to 10 459·3)	7222 (5168 to 9915)	17 747 (12 374 to 24 089)	687·7 (492·0 to 944·1)	671·9 (468·4 to 911·9)
		Côte d'Ivoire	207 725 (191 596 to 225 049)	325 273 (299 003 to 351 973)	10 442·8 (9632·0 to 11 313·7)	9140·2 (8402·0 to 9890·5)	13 898 (9974 to 18 709)	23 939 (16 865 to 33 333)	698·7 (501·4 to 940·5)	672·7 (473·9 to 936·7)
		The Gambia	18 056 (16 072 to 20 299)	33 238 (29 492 to 36 922)	9883·3 (8797·4 to 11 111·0)	9065·7 (8044·1 to 10 070·6)	1383 (974 to 1844)	2872 (2093 to 4023)	757·2 (533·1 to 1009·1)	783·5 (570·8 to 1097·2)
		Ghana	241 529 (222 998 to 263 580)	373 912 (340 586 to 411 048)	9923·7 (9162·4 to 10 829·7)	8602·7 (7836·0 to 9457·1)	18 653 (13 371 to 25 289)	33 385 (22 714 to 47 944)	766·4 (549·4 to 1039·1)	768·1 (522·6 to 1103·1)
		Guinea	94 700 (83 414 to 106 690)	190 362 (169 748 to 212 868)	10 266·5 (9043·0 to 11 566·4)	9354·9 (8341·9 to 10 461·0)	6334 (4384 to 8847)	14 483 (10 018 to 19 978)	686·7 (475·3 to 959·1)	711·7 (492·3 to 981·8)
		Guinea-Bissau	17 499 (15 560 to 19 675)	28 482 (25 335 to 31 810)	10 452·1 (9294·0 to 11 751·9)	9188·7 (8173·4 to 10 262·1)	1152 (808 to 1580)	2141 (1495 to 2983)	688·1 (482·9 to 943·6)	690·8 (482·4 to 962·4)
		Liberia	42 443 (37 509 to 47 576)	67 948 (60 384 to 76 189)	10 522·9 (9299·5 to 11 795·4)	9532·3 (8471·2 to 10 688·5)	3056 (2103 to 4244)	5344 (3722 to 7348)	757·6 (521·3 to 1052·1)	749·7 (522·2 to 1030·8)
		Mali	151 015 (136 599 to 165 815)	264 821 (238 858 to 289 075)	9739·0 (8809·3 to 10 693·4)	8407·0 (7582·8 to 9176·9)	10 657 (7626 to 14 114)	21 298 (14 954 to 29 028)	687·3 (491·8 to 910·2)	676·1 (474·7 to 921·5)
		Mauritania	37 028 (34 392 to 39 758)	54 156 (49 459 to 58 568)	12 612·9 (11 714·8 to 13 542·5)	10 528·7 (9615·4 to 11 386·3)	2414 (1701 to 3281)	4300 (2938 to 6067)	822·2 (579·4 to 1117·6)	836·0 (571·1 to 1179·5)
		Niger	164 510 (145 322 to 183 831)	388 768 (344 131 to 435 501)	11 325·9 (10 004·8 to 12 656·0)	10 554·0 (9342·2 to 11 822·7)	10 452 (7429 to 14 414)	26 490 (18 737 to 36 230)	719·6 (511·4 to 992·3)	719·1 (508·7 to 983·5)
		Nigeria	1 476 143 (1 317 058 to 1 639 394)	2 458 460 (2 190 812 to 2 749 552)	9796·5 (8740·7 to 10 880·0)	8324·2 (7418·0 to 9309·8)	105 830 (75 766 to 144 421)	196 858 (137 846 to 275 936)	702·4 (502·8 to 958·5)	666·5 (466·7 to 934·3)
		São Tomé and Príncipe	1913 (1694 to 2129)	2857 (2542 to 3169)	9705·0 (8594·7 to 10 803·0)	8580·1 (7634·8 to 9518·1)	152 (107 to 207)	261 (182 to 367)	770·1 (540·7 to 1049·8)	783·8 (545·8 to 1102·9)
		Senegal	121 632 (111 286 to 132 866)	226 916 (206 184 to 248 687)	9901·6 (9059·4 to 10 816·1)	8926·7 (8111·1 to 9783·1)	9671 (6824 to 13 063)	20 198 (14 292 to 27 945)	787·3 (555·5 to 1063·4)	794·6 (562·2 to 1099·3)
		Sierra Leone	59 363 (52 756 to 66 760)	89 956 (80 107 to 100 511)	9921·5 (8817·2 to 11 157·8)	8650·0 (7703·0 to 9665·0)	3775 (2632 to 5167)	6559 (4605 to 9276)	631·0 (439·9 to 863·6)	630·7 (442·8 to 892·0)
		Togo	64 227 (57 084 to 71 848)	97 409 (87 002 to 108 389)	10 178·3 (9046·4 to 11 386·1)	8906·2 (7954·7 to 9910·0)	4844 (3376 to 6774)	8214 (5758 to 11 523)	767·7 (535·0 to 1073·5)	751·0 (526·4 to 1053·5)
	Eastern sub-Saharan Africa		3 412 289 (3 091 032 to 3 749 540)	5 573 942 (5 032 121 to 6 110 163)	10 107·4 (9155·8 to 11 106·3)	8908·9 (8042·9 to 9765·9)	253 465 (190 111 to 330 218)	475 608 (344 603 to 647 554)	750·8 (563·1 to 978·1)	760·2 (550·8 to 1035·0)
		Burundi	105 740 (93 725 to 120 993)	202 093 (177 356 to 227 968)	9941·4 (8811·8 to 11 375·5)	9433·1 (8278·5 to 10 640·9)	7791 (5532 to 10 506)	15 641 (10 791 to 21 423)	732·5 (520·1 to 987·8)	730·1 (503·7 to 1000·0)
		Comoros	8051 (7422 to 8747)	9806 (8959 to 10 732)	12 562·5 (11 580·9 to 13 648·5)	10 569·1 (9655·9 to 11 566·9)	517 (354 to 688)	746 (528 to 1028)	806·5 (552·3 to 1073·1)	803·7 (568·9 to 1108·5)
		Djibouti	11 006 (9709 to 12 451)	14 608 (12 956 to 16 536)	9843·0 (8683·7 to 11 135·6)	8472·6 (7514·4 to 9590·6)	854 (591 to 1181)	1320 (888 to 1906)	763·5 (528·4 to 1056·4)	765·8 (515·2 to 1105·2)
		Eritrea	63 370 (56 211 to 72 094)	74 548 (66 074 to 84 199)	11 212·7 (9945·9 to 12 756·3)	9373·1 (8307·6 to 10 586·4)	4653 (3408 to 6218)	6688 (4866 to 9431)	823·4 (602·9 to 1100·3)	840·9 (611·8 to 1185·8)
		Ethiopia	785 925 (711 134 to 862 410)	1 320 045 (1 197 956 to 1 455 318)	10 210·8 (9239·1 to 11 204·4)	8636·5 (7837·7 to 9521·5)	59 032 (43 034 to 78 712)	117 697 (83 543 to 164 084)	766·9 (559·1 to 1022·6)	770·0 (546·6 to 1073·5)
		Kenya	434 918 (403 139 to 467 459)	655 871 (607 766 to 705 248)	10 066·5 (9331·0 to 10 819·7)	9977·5 (9245·7 to 10 728·6)	35 669 (26 381 to 47 161)	56 691 (41 725 to 75 573)	825·6 (610·6 to 1091·6)	862·4 (634·7 to 1149·7)
		Madagascar	228 772 (204 517 to 254 158)	371 418 (331 001 to 411 783)	10 519·0 (9403·7 to 11 686·2)	9551·0 (8511·7 to 10 589·0)	17 389 (12 316 to 23 192)	30 891 (22 112 to 42 269)	799·6 (566·3 to 1066·4)	794·4 (568·6 to 1087·0)
		Malawi	145 810 (131 904 to 160 385)	249 622 (221 779 to 277 295)	8595·4 (7775·6 to 9454·5)	7797·4 (6927·7 to 8661·9)	11 810 (8329 to 16 167)	23 408 (16 289 to 32 505)	696·2 (491·0 to 953·0)	731·2 (508·8 to 1015·4)
		Mozambique	259 337 (227 862 to 291 274)	479 221 (423 575 to 540 445)	11 404·5 (10 020·4 to 12 808·9)	9649·5 (8529·1 to 10 882·4)	17 546 (12 053 to 24 081)	39 092 (26 822 to 54 469)	771·6 (530·0 to 1059·0)	787·1 (540·1 to 1096·8)
		Rwanda	146 537 (128 423 to 165 157)	162 254 (143 807 to 181 097)	10 267·0 (8997·9 to 11 571·7)	8666·8 (7681·4 to 9673·4)	10 942 (7822 to 14 789)	14 269 (9965 to 20 247)	766·6 (548·0 to 1036·2)	762·2 (532·3 to 1081·5)
		Somalia	100 476 (87 943 to 113 534)	134 702 (118 979 to 151 229)	10 718·5 (9381·5 to 12 111·4)	10 214·0 (9021·8 to 11 467·2)	6816 (4852 to 9204)	10 305 (7362 to 14 011)	727·1 (517·6 to 981·9)	781·4 (558·2 to 1062·4)
		South Sudan	162 890 (144 861 to 182 314)	319 254 (282 816 to 356 600)	12 411·9 (11 038·1 to 13 892·0)	11 161·6 (9887·7 to 12 467·3)	10 089 (7064 to 13 938)	21 747 (15 295 to 29 920)	768·7 (538·2 to 1062·0)	760·3 (534·8 to 1046·1)
		Tanzania	469 787 (415 627 to 537 009)	739 263 (653 817 to 824 299)	9495·5 (8400·8 to 10 854·2)	8211·4 (7262·3 to 9156·0)	34 183 (24 290 to 45 863)	63 703 (43 725 to 88 463)	690·9 (491·0 to 927·0)	707·6 (485·7 to 982·6)
		Uganda	345 183 (301 837 to 393 407)	613 715 (537 984 to 687 875)	9651·1 (8439·2 to 10 999·5)	8163·7 (7156·3 to 9150·2)	25 231 (17 469 to 34 522)	53 439 (36 237 to 74 895)	705·5 (488·4 to 965·2)	710·9 (482·0 to 996·2)
		Zambia	142 646 (130 778 to 154 927)	224 384 (202 238 to 245 931)	9064·3 (8310·1 to 9844·6)	7894·6 (7115·5 to 8652·7)	10 805 (7649 to 14 841)	19 704 (13 534 to 27 774)	686·6 (486·0 to 943·1)	693·2 (476·2 to 977·2)
	Central sub-Saharan Africa		1 320 698 (1 237 965 to 1 399 075)	2 755 934 (2 571 283 to 2 945 481)	14 283·2 (13 388·5 to 15 130·9)	13 253·1 (12 365·1 to 14 164·6)	72 391 (52 597 to 94 366)	172 301 (124 475 to 231 839)	782·9 (568·8 to 1020·6)	828·6 (598·6 to 1114·9)
		Angola	311 174 (292 300 to 331 133)	575 427 (535 751 to 614 576)	14 586·6 (13 701·8 to 15 522·2)	11 728·3 (10 919·7 to 12 526·3)	16 692 (11 890 to 22 394)	37 564 (26 209 to 867)	782·5 (557·4 to 1049·8)	765·6 (534·2 to 1057·1)
		Central African Republic	66 308 (61 775 to 70 949)	103 693 (96 234 to 111 613)	15 030·9 (14 003·4 to 16 082·9)	13 974·0 (12 968·7 to 15 041·2)	3654 (2648 to 4877)	6175 (4444 to 8282)	828·3 (600·3 to 1105·6)	832·2 (598·9 to 1116·1)
		Congo (Brazzaville)	50 729 (47 417 to 54 276)	80 001 (74 038 to 86 182)	12 521·5 (11 703·8 to 13 396·8)	10 736·0 (9935·8 to 11 565·5)	3203 (2313 to 4351)	5757 (4033 to 8257)	790·6 (571·0 to 1073·9)	772·6 (541·2 to 1108·0)
		Democratic Republic of the Congo	867 649 (810 350 to 922 155)	1 964 218 (1 827 979 to 2 101 721)	14 307·3 (13 362·4 to 15 206·1)	13 973·0 (13 003·9 to 14 951·2)	47 274 (33 791 to 61 971)	120 278 (84 970 to 164 263)	779·5 (557·2 to 1021·9)	855·6 (604·5 to 1168·5)
		Equatorial Guinea	9378 (8762 to 10 013)	8770 (8081 to 9420)	14 259·0 (13 322·9 to 15 225·4)	8846·9 (8151·7 to 9503·0)	543 (383 to 721)	616 (428 to 881)	825·8 (582·6 to 1096·5)	621·6 (432·2 to 888·2)
		Gabon	15 459 (14 396 to 16 580)	23 825 (21 949 to 25 891)	11 303·2 (10 525·5 to 12 122·7)	9730·7 (8964·5 to 10 574·7)	1024 (727 to 1417)	1910 (1328 to 2839)	748·9 (531·8 to 1035·9)	780·2 (542·3 to 1159·3)

Data in parentheses are 95% uncertainty intervals. YLD=years lived with disability. SDI=Sociodemographic index. GBD=Global Burden of Disease.

**Figure 1:**
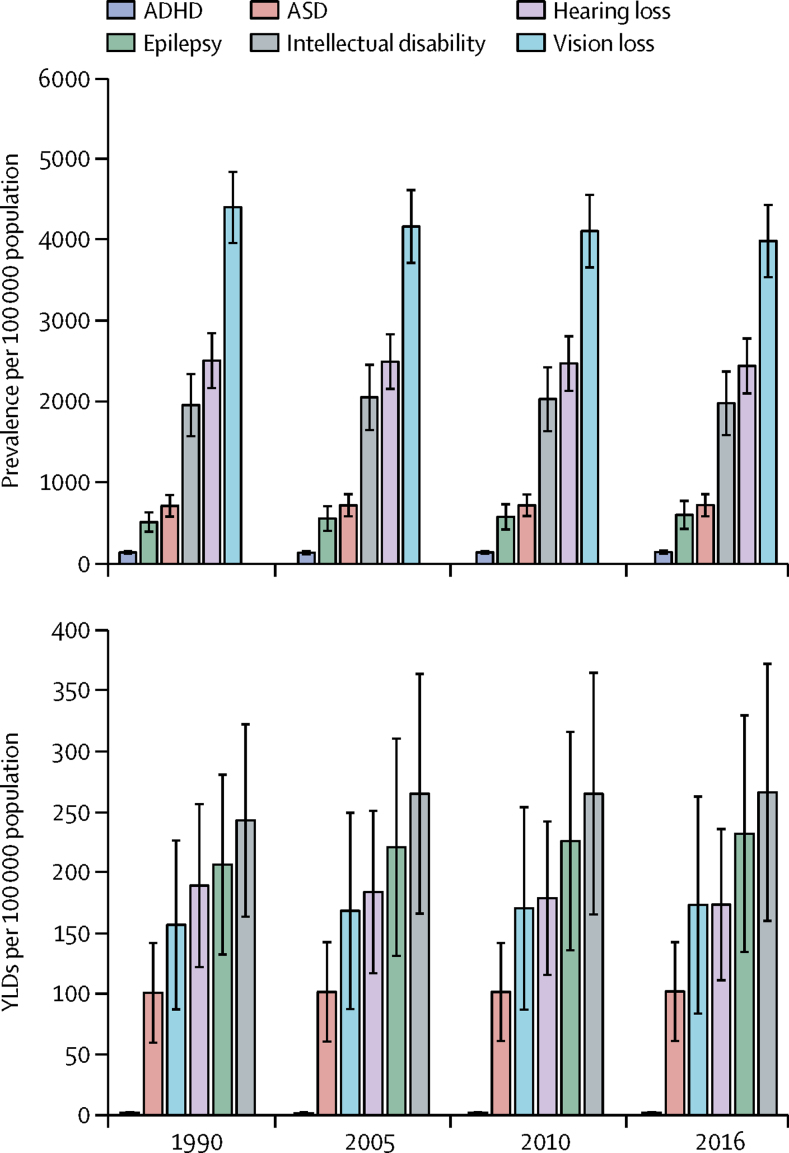
Global trends in developmental disabilities between 1990 and 2016 among children younger than 5 years ADHD=attention deficit hyperactivity disorder. ASD=autism spectrum disorder. YLD=years lived with disability.

Vision loss was the most prevalent developmental disability and decreased from 1990 to 2016 (figure 1). 26·4 million (95% UI 24·0–29·2) children had vision loss in 1990, corresponding to a prevalence of 4407 (95% UI 4006–4887) per 100 000 children. This figure declined to 25·2 million (22·7–28·4) in 2016, corresponding to a prevalence of 3991 (3595–4487) per 100 000 children. Hearing loss was the second most prevalent disability, affecting 15·0 million (13·0–17·1) children (prevalence 2511 [95% UI 2178–2859] per 100 000) in 1990. Although the prevalence of hearing loss decreased to 2445 (95% UI 2113–2793) per 100 000 children in 2016, the number of affected children increased slightly to 15·5 million (95% UI 13·4–17·7). ADHD was the least prevalent disability during the period, but increased from 835 171 (746 061–946 713) affected children in 1990 to 890 229 (794 104–1 022 157) in 2016. Intellectual disability was associated with the highest YLDs throughout the period, followed by epilepsy, hearing loss, vision loss, ASD, and ADHD (figure 1). Compared with the estimates in 1990, the YLDs for intellectual disability and epilepsy increased in 2016, whereas the YLDs for hearing loss showed a steady but modest decline up to 2016. Generally, the UIs for the YLDs were wider than those for prevalence (figure 1).

**Figure 2:**
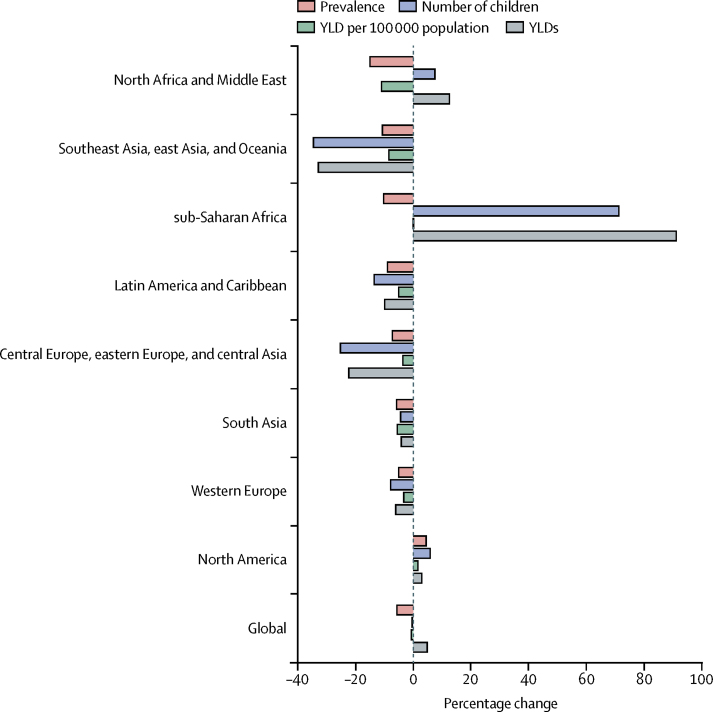
Percentage change in all developmental disabilities in children younger than 5 years between 1990 and 2016 YLD=years lived with disability.

Although the prevalence of developmental disabilities in children younger than 5 years declined from 1990 to 2006 in all regions except North America, the number of affected children increased by 71·3% in sub-Saharan Africa, 7·6% in north Africa and the Middle East, and 5·9% in North America (figure 2, table 1). These increases were offset globally by a decline in the number of children with disabilities in all other regions during this period, with southeast Asia, east Asia, and Oceania recording the largest decline of 34·5%. The six disabilities accounted for 3·8 million (95% UI 2·8–4·9) YLDs in 1990 compared with 3·9 million (2·9–5·2) in 2016 (table 1), representing 13·3% of the total 29·3 million (20·7–39·8) YLDs from all causes in children younger than 5 years in 2016. Sub-Saharan Africa recorded the highest increase in YLDs (91·1%), whereas southeast Asia, east Asia, and Oceania recorded the greatest decrease (−32·8%; figure 2).

### Regional and national prevalence of developmental disabilities in 2016

**Figure 3:**
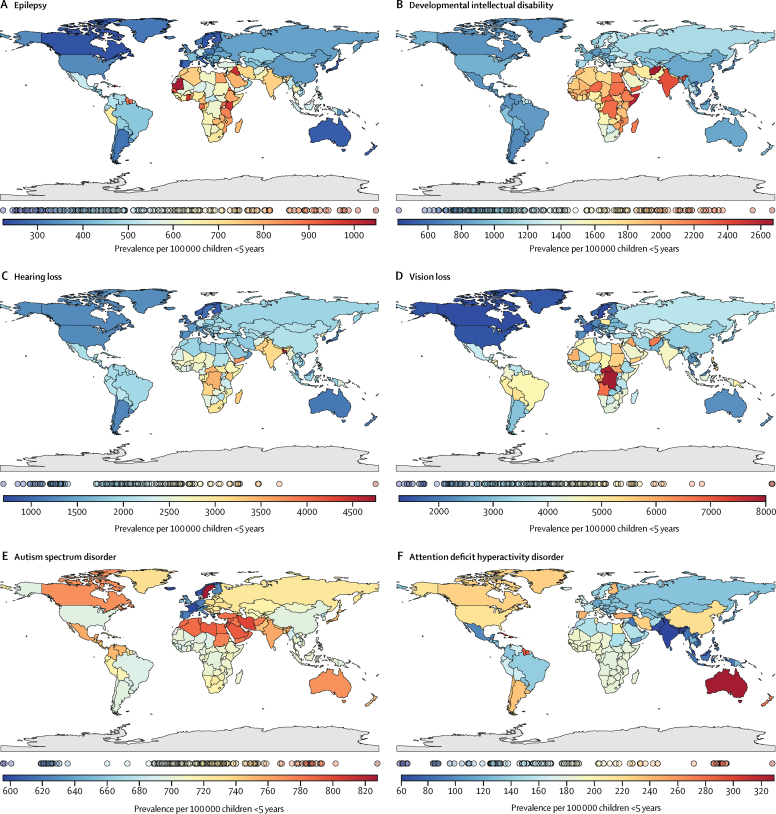
Global prevalence per 100 000 population of developmental disabilities among children younger than 5 years in 2016

The geographical distribution of developmental disabilities in 2016 based on prevalence estimates is shown in figure 3. Unlike most developmental disabilities, ASD was least prevalent in western Europe and ADHD in south Asia. The highest number of children with intellectual disability (3·9 million [95% UI 3·1–4·7]), hearing loss (5·1 million [4·4–5·8]), and ASD (1·2 million [1·0–1·4]) were recorded in south Asia (table 2), accounting for 31·1%, 32·9%, and 25·4% of the global prevalence, respectively. The highest number of children with epilepsy (1·2 million [0·8–1·7]), vision loss (6·9 million [6·2–7·8]), and ADHD (284 197 [251 986–325 635]) were recorded in sub-Saharan Africa, representing 30·4%, 27·5%, and 31·9% of the global prevalence, respectively. The prevalence per 100 000 children of intellectual disability (2567 [95% UI 2099–3189]), vision loss (4947 [4324–5803]), and ASD (785 [645–948]) were highest in north Africa and the Middle East. Epilepsy, hearing loss, and vision loss were least prevalent in North America, whereas intellectual disability was least prevalent in Latin America. The highest prevalence of children with all disabilities was recorded in south Asia (9792 [8940–10 641] per 100 000) and the lowest in North America (4090 [3735–4467] per 100 000; table 1).

**Table 2:** Global and regional prevalence of developmental disabilities and YLDs among children younger than 5 years in 2016

	**Epilepsy**		**Intellectual disability**		**Hearing loss**		**Vision loss**		**ASD**		**ADHD**	
	Cases	YLDs	Cases	YLDs	Cases	YLDs	Cases	YLDs	Cases	YLDs	Cases	YLDs
**High-income North America**												
Number in 2016	71 653 (57 749 to 87 401)	22 008 (15 396 to 30 350)	310 023 (282 715 to 341 273)	39 287 (28 434 to 52 255)	273 277 (222 909 to 334 653)	16 663 (11 253 to 23 156)	311 691 (275 984 to 358 104)	13 596 (9020 to 20 484)	151 645 (124 270 to 181 684)	24 096 (15 377 to 35 154)	46 710 (41 905 to 52 513)	573 (345 to 916)
Per 100 000 population	332 (267 to 404)	102 (71 to 140)	1434 (1308 to 1579)	182 (132 to 242)	1264 (1031 to 1548)	77 (52 to 107)	1442 (1277 to 1657)	63 (42 to 95)	702 (575 to 841)	111 (71 to 163)	216 (194 to 243)	3 (2 to 4)
**Western Europe**												
Number in 2016	79 002 (57 881 to 105 444)	23 991 (15 648 to 35 651)	327 359 (298 460 to 361 756)	40 140 (28 887 to 54 357)	307 575 (242 689 to 382 474)	20 075 (13 064 to 28 632)	460 788 (394 282 to 552 778)	20 037 (13 170 to 30 392)	137 281 (112 032 to 165 492)	21 961 (14 075 to 31 661)	36 490 (32 064 to 36 490)	448 (259 to 733)
Per 100 000 population	360 (264 to 480)	109 (71 to 162)	1492 (1360 to 1648)	183 (131 to 248)	1401 (1106 to 1743)	91 (60 to 130)	2100 (1797 to 2519)	91 (60 to 138)	626 (511 to 754)	100 (64 to 144)	166 (146 to 195)	2 (1 to 3)
**Central Europe, eastern Europe, and central Asia**												
Number in 2016	113 423 (84 345 to 154 252)	40 967 (26 254 to 61 946)	440 549 (379 781 to 505 012)	64 790 (46 564 to 88 756)	555 762 (469 274 to 648 697)	36 089 (24 021 to 50 056)	1 049 687 (936 194 to 1 190 142)	39 400 (25 350 to 60 912)	203 912 (166 105 to 249 993)	29 456 (19 059 to 43 240)	36 580 (31 794 to 42 765)	447 (249 to 734)
Per 100 000 population	402 (299 to 547)	145 (93 to 220)	1563 (1347 to 1791)	230 (165 to 315)	1971 (1665 to 2301)	128 (85 to 178)	3723 (3321 to 4221)	140 (90 to 216)	723 (589 to 887)	104 (68 to 153)	130 (113 to 152)	2 (1 to 3)
**Latin America and Caribbean**												
Number in 2016	252 539 (194 217 to 335 785)	88 684 (59 778 to 130 931)	559 143 (476 346 to 657 291)	100 006 (71 865 to 144 783)	1 004 037 (854 075 to 1 160 668)	66 136 (44 962 to 91 980)	2 187 447 (1 969 105 to 2 454 166)	78 686 (51 002 to 121 141)	360 585 (296 045 to 435 834)	52 508 (33 825 to 75 356)	70 952 (63 427 to 80 791)	866 (521 to 1349)
Per 100 000 population	509 (392 to 677)	179 (121 to 264)	1127 (960 to 1325)	202 (145 to 292)	2024 (1722 to 2340)	133 (91 to 185)	4410 (3970 to 4947)	159 (103 to 244)	723 (597 to 879)	106 (68 to 152)	143 (128 to 163)	2 (1 to 3)
**Southeast Asia, east Asia, and Oceania**												
Number in 2016	550 361 (449 370 to 682 790)	205 425 (146 556 to 276 087)	1 526 594 (1 272 874 to 1 808 117)	180 002 (131 683 to 241 026)	2 502 188 (2 132 437 to 2 892 026)	174 839 (118 980 to 245 338)	4 437 027 (4 038 292 to 4 890 923)	139 661 (93 654 to 205 769)	857 399 (707 955 to 1 034 575)	122 431 (78 147 to 177 225)	191 143 (169 137 to 217 737)	2340 (1387 to 3768)
Per 100 000 population	447 (365 to 555)	167 (119 to 224)	1240 (1034 to 1469)	146 (107 to 196)	2033 (1733 to 2350)	142 (97 to 199)	3605 (3281 to 3974)	115 (77 to 161)	697 (575 to 841)	99 (64 to 144)	155 (137 to177)	2 (1 to 3)
**South Asia**												
Number in 2016	1 086 215 (868 208 to 1 396 888)	402 373 (287 052 to 580 075)	3 898 006 (3 087 552 to 4 731 270)	502 557 (361 986 to 692 218)	5 081 442 (4 378 467 to 5 833 039)	334 920 (227 698 to 468 422)	6 317 097 (5 639 628 to 7 100 960)	301 941 (199 879 to 459 045)	1 158 930 (952 782 to 1 412 005)	156 229 (100 111 to 223 956)	93 109 (82 862 to 105 772)	1119 (643 to 1731)
Per 100 000 population	707 (565 to 909)	262 (187 to 378)	2538 (2010 to 3080)	327 (236 to 451)	3308 (2850 to 3797)	218 (148 to 305)	4112 (3671 to 4623)	150 (89 to 235)	754 (620 to 919)	102 (65 to 146)	61 (54 to 69)	1 (0 to 1)
**Sub-Saharan Africa**												
Number in 2016	1 156 803 (835 013 to 1 732 553)	483 345 (314 795 to 790 918)	3 667 141 (2 864 624 to 4 530 288)	518 025 (348 768 to 836 328)	4 246 819 (3 670 811 to 4 818 705)	335 226 (222 613 to 464 832)	6 926 422 (6 177 799 to 7 764 018)	330 748 (195 372 to 601 938)	1 099 306 (891 925 to 1 346 171)	153 107 (97 244 to 224 706)	284 197 (251 986 to 325 635)	3436 (2051 to 5535)
Per 100 000 population	739 (533 to 1106)	309 (201 to 505)	2342 (1829 to 2893)	331 (223 to 534)	2712 (2344 to 3077)	214 (142 to 297)	4423 (3945 to 4958)	225 (137 to 378)	702 (570 to 860)	98 (62 to 144)	181 (161 to 208)	2 (1 to 4)
**North Africa and Middle East**												
Number in 2016	458 924 (321 791 to 815 593)	188 755 (117 642 to 365 278)	1 622 198 (1 326 628 to 2 015 277)	215 668 (141 656 to 399 087)	1 323 243 (1 114 347 to 1 548 598)	103 404 (67 791 to 147 662)	3 125 867 (2 732 186 to 3 667 064)	157 408 (93 086 to 324 135)	496 079 (407 784 to 598 783)	68 195 (43 754 to 98 484)	105 648 (92 244 to 121 683)	1285 (732 to 2062)
Per 100 000 population	726 (509 to 1291)	299 (186 to 578)	2567 (2099 to 3189)	341 (224 to 632)	2094 (1763 to 2451)	164 (107 to 234)	4947 (4324 to 5803)	226 (127 to 429)	785 (645 to 948)	108 (69 to 156)	167 (146 to 193)	2 (1 to 3)
**Global**												
Number in 2016	3 809 557 (3 023 986 to 5 199 796)	1 468 478 (1 022 639 to 2 256 241)	12 534 231 (10 183 368 to 15 146 763)	1 684 149 (1 200 213 to 2 541 784)	15 452 005 (13 352 922 to 17 650 432)	1 098 262 (741 048 to 1 529 677)	25 222 595 (22 718 169 to 28 355 185)	1 096 187 (694 904 to 1 827 213)	4 567 675 (3 755 747 to 5 480 485)	644 290 (413 473 to 929 339)	890 229 (794 104 to 1 022 157)	10 826 (6477 to 17 138)
Per 100 000 population	603 (479 to 823)	232 (162 to 357)	1983 (1611 to 2397)	266 (190 to 402)	2445 (2113 to 2793)	174 (117 to 242)	3991 (3595 to 4487)	173 (110 to 289)	723 (594 to 867)	102 (65 to 147)	141 (125 to 162)	2 (1 to 3)

Data in parentheses are 95% uncertainty intervals unless otherwise stated. ASD=autism spectrum disorder. ADHD=attention deficit hyperactivity disorder. YLD=years lived with disability.

Country-specific prevalence estimates for each disability are presented in the appendix. For all disabilities, India had the highest number of affected children, except for ADHD, for which the highest number of affected children was in China (table 3). On the African continent, Nigeria, Democratic Republic of the Congo, and Ethiopia were among the top ten countries with the highest number of children with any disability. The USA was one of the top ten countries for intellectual disability, hearing loss, ASD, and ADHD. The highest prevalence of each disability was found in LMICs, apart from ASD and ADHD, which were highest in Sweden and Australia. The top ten countries accounted for more than half of the global prevalence of all developmental disabilities except for ADHD (48·2%; table 3).

**Table 3:** Top ten countries with specific developmental disabilities in children younger than 5 years in 2016

	**Epilepsy**		**Intellectual disability**		**Hearing loss**		**Vision loss**		**Autism spectrum disorder**		**ADHD**	
	Cases	YLDs	Cases	YLDs	Cases	YLDs	Cases	YLDs	Cases	YLDs	Cases	YLDs
**Ranking by number in 2016**												
1	India	India	India	India	India	India	India	India	India	India	China	China
2	China	Nigeria	China	Nigeria	China	China	China	Nigeria	China	China	India	India
3	Nigeria	China	Nigeria	China	Nigeria	Nigeria	DR Congo	China	Nigeria	Nigeria	Nigeria	Nigeria
4	Pakistan	Pakistan	Pakistan	Pakistan	Pakistan	Bangladesh	Nigeria	Pakistan	Pakistan	Pakistan	USA	USA
5	Indonesia	Ethiopia	DR Congo	Ethiopia	Bangladesh	Pakistan	Indonesia	Ethiopia	Indonesia	Indonesia	Ethiopia	Ethiopia
6	Ethiopia	Indonesia	Ethiopia	DR Congo	DR Congo	DR Congo	Brazil	DR Congo	USA	USA	DR Congo	DR Congo
7	Egypt	Egypt	Egypt	Egypt	Indonesia	Ethiopia	Pakistan	Egypt	Ethiopia	Brazil	Egypt	Egypt
8	DR Congo	DR Congo	USA	Indonesia	Ethiopia	Indonesia	Philippines	Iraq	Brazil	Ethiopia	Brazil	Brazil
9	Bangladesh	Iraq	Indonesia	USA	Brazil	Brazil	Egypt	Indonesia	Bangladesh	Bangladesh	Indonesia	Indonesia
10	Tanzania	Tanzania	Bangladesh	Iraq	USA	Philippines	Ethiopia	Bangladesh	DR Congo	DR Congo	Iran	Iran
Top 10 total (proportion of global)*	1 979 233 (52·0%)	761 996 (51·9%)	6 830 618 (54·5%)	881 855 (52·4%)	8 872 948 (57·4%)	610 796 (55·6%)	13 427 729 (53·2%)	572 788 (52·3%)	2 366 873 (51·8%)	329 781 (51·2%)	429 470 (48·2%)	5218 (48·2%)
**Ranking per 100 000 population**												
1	Mauritania	Dominican Republic	Afghanistan	Dominican Republic	Bangladesh	Yemen	DR Congo	Dominican Republic	Sweden	Australia	Australia	Australia
2	Dominican Republic	Iraq	Yemen	Iraq	Yemen	Bangladesh	Central African Republic	Iraq	Iran	Sweden	Cuba	Cuba
3	Iraq	Mauritania	Sudan	Afghanistan	DR Congo	DR Congo	Angola	Suriname	Syria	Japan	Trinidad and Tobago	Virgin Islands
4	Ghana	Ghana	Niger	Palestine	Central African Republic	Central African Republic	Afghanistan	Guyana	Saudi Arabia	New Zealand	Suriname	Dominican Republic
5	Kenya	São Tomé and Príncipe	Liberia	Suriname	Kenya	Madagascar	Iraq	Palestine	Libya	Canada	Virgin Islands	Trinidad and Tobago
6	São Tomé and Príncipe	Kenya	Central African Republic	Dominica	Bhutan	Kenya	South Sudan	Dominica	Kuwait	Singapore	Dominican Republic	Barbados
7	Cape Verde	Suriname	Somalia	The Gambia	Madagascar	Angola	Mauritania	Gabon	Bahrain	Greenland	Grenada	Suriname
8	The Gambia	Eritrea	India	Eritrea	Pakistan	South Africa	Congo (Brazzaville)	Mauritania	United Arab Emirates	Brunei	Barbados	Saint Lucia
9	Senegal	Senegal	DR Congo	Guyana	Angola	Somalia	Egypt	Egypt	Jordan	Iran	Saint Lucia	Antigua and Barbuda
10	Eritrea	Guyana	Palestine	Sudan	India	Burundi	Comoros	Afghanistan	Lebanon	Kuwait	Saint Vincent and the Grenadines	Saint Vincent and the Grenadines

ADHD=attention deficit hyperactivity disorder. YLD=years lived with disability.

* Percentages indicate proportions of the global count that are accounted for by the top ten countries.

### Regional and national YLDs of developmental disabilities in 2016

The highest YLDs for all disabilities except for ASD were reported in sub-Saharan Africa (the highest YLDs for ASD were recorded in south Asia; table 2). The lowest YLDs for all developmental disabilities except ASD and ADHD were reported in North America. The lowest YLDs for ASD were reported in western Europe, and for ADHD in central Europe, eastern Europe, and central Asia. However, the highest YLDs per 100 000 population for intellectual disability and vision loss were reported in north Africa and the Middle East, while the highest YLDs per 100 000 population for hearing loss were recorded in south Asia. The top ten countries accounted for more than half of the YLDs for all disabilities except ADHD which amounted to 48·2%. On a regional basis, the highest YLDs per 100 000 population for the six disabilities combined was recorded in north Africa and the Middle East (758 [95% UI 540–1090]) and the lowest in North America (346 [254–451]; table 1).

Country-specific YLDs for each disability are presented in the appendix. The highest YLDs for all disabilities except ADHD were found in India, while the highest YLDs for ADHD were recorded in China. The highest YLDs for epilepsy, intellectual disability, and vision loss were in Dominican Republic, for hearing loss in Yemen, and for ADHD and ASD in Australia (table 3).

### Leading causes of developmental disabilities in 2016

The underlying causes of the four disorders conceptually modelled as impairments in GBD 2016 are presented in the appendix. Refraction and accommodation disorders (85·2%) were the leading cause of vision loss globally. The major causes of hearing loss were otitis media (57·1%) and congenital anomalies (21·1%). Congenital anomalies (39·7%) and neonatal disorders, including preterm birth complications, infections, and birth asphyxia (21·0%), were the prominent known causes of intellectual disability, whereas idiopathic causes accounted for 29·0%. Neonatal disorders (51·8%) were the leading cause of epilepsy. The extent to which these causes contributed to each impairment varied across the regions and by country.

## Discussion

About 52·9 million (8·4%) children worldwide had one of the six developmental disabilities investigated by the GBD group in 2016 compared with 53·0 million (8·9%) in 1990. This marginal change is in sharp contrast with the trend in under-5 mortality, which substantially declined from 11 million (95% UI 10·8–11·3) in 1990 to 5 million (4·8–5·2) in 2016.^14^ The disproportionately high burden of development disabilities in LMICs has been corroborated by a 2018 systematic review.^21^ The increasing burden reflects the absence of any systematic global initiative to curtail this burden among the increasing number of children who are surviving the first 5 years of life. It is likely that improving neonatal survival rates, particularly for those born prematurely, is contributing substantially to this burden, especially during the MDG era.

As with most health conditions, the dearth of population-based data for developmental disabilities, especially in LMICs,^21^ has led to a reliance on statistical estimation of trends in health outcomes to guide health policy and interventions. This practice is underpinned by the view that the absence of evidence is not evidence of absence. In this study, we aimed to distil the best available epidemiological data on the global burden of developmental disabilities among children younger than 5 years from the extensive GBD 2016 database to facilitate an objective assessment of progress in promoting early childhood development under the SDG framework.

Although a direct comparison with our study is impracticable because of differences in methods, Lu and colleagues^11^ estimated a declining trend between 2007 and 2010 in the number of developmentally at-risk children exposed to stunting or extreme poverty in LMICs. When combining data from our study with data from other studies^11^, ^12^ of early childhood development based on stunting, extreme poverty, and low cognition, we obtained a crude estimate of 350 million for the number of children at risk of not realising their developmental potential in LMICs, or three in every five children younger than 5 years (appendix). This estimate is likely to be an underestimate since we have excluded prominent disabilities such as idiopathic cerebral palsy and other motor disorders from the study. However, given that this estimate includes neither separate estimates of cerebral palsy nor quantification of the role of home life and access to education, there is at best sparse information about the effects of modest growth and nutritional deficits on the long-term neurocognitive development of young children. Our study also suggests that the burden is higher in males than in females for most developmental disabilities.

Globally, YLDs associated with developmental disabilities have not improved since 1990. The only exception perhaps was the modest decline in vision loss, which might be attributable to effects of the Vision 2020 global initiative, which was launched in 1999 by WHO.^22^ Although hearing and vision loss were the most prevalent among the six disabilities from 1990 to 2016, intellectual disability and epilepsy were associated with the highest YLDs in children younger than 5 years. It is possible that rehabilitation with glasses and hearing aids alleviated the effects of impairment in some children with sensory losses, whereas children with intellectual disability and epilepsy experience greater societal stigma and support services are often inadequate in LMIC settings. However, over the entire life course, hearing loss was found to be the third leading cause of YLDs globally in 2016, and the second leading cause if combined with vision loss.^13^ Recognising the need for comprehensive early detection and intervention for all children with or at risk of any developmental disability from birth is perhaps more crucial than in later years, given the substantial YLDs associated with these conditions over a life course.

Summary global estimates often mask significant variations at the regional and national levels. Although minimal decline was observed between 1990 and 2016 globally, the burden of all developmental disabilities substantially increased in sub-Saharan Africa and in north Africa and the Middle East. Moreover, south Asia and sub-Saharan Africa, which frequently account for the highest child mortality globally, had the highest number of children with developmental disabilities, although south Asia showed some modest progress. This finding is consistent with other reports on the risk of suboptimal development among children younger than 5 years.^10^, ^11^, ^12^ For example, regardless of the differences in case definition, India, China, and Nigeria were the three leading countries with the highest number of children with or at risk of developmental disabilities. In 2016, Democratic Republic of the Congo displaced Nigeria from the third position for vision loss. Except for the USA, China, and Brazil, the ten leading countries for developmental disabilities were predominantly from south Asia and sub-Saharan Africa. This pattern of disease burden exemplifies the ethical rationale of the disability-inclusive framework of the SDGs that seeks to promote and ensure safety nets for the survivors of acute childhood illnesses in LMICs to set them on the trajectory of optimal early childhood development.

Given the variability in population sizes, the countries with the highest prevalence were usually not among the leading countries by actual number of children. Population-based data in some high-income countries, such as the USA, in fact show significant increasing trends in the prevalence of most developmental disabilities due in part to increases in the diagnosis and recognition of developmental disorders such as ASD and ADHD.^23^, ^24^, ^25^ This trend is consistent with the observed increases in the overall burden of developmental disabilities between 1990 and 2016 in North America. Moreover, the prevalence of all disabilities in the USA in GBD 2016 (4·1%, 95% UI 3·8–4·5) is comparable to the 5·2% reported among children younger than 6 years in the study by Houtrow and colleagues.^23^ The low prevalence of ADHD that we found globally compared with other disabilities could be partly attributable to the fact that no incidence was assumed before age 2 years in our modelling strategy (appendix). Also, except for severely affected children, the onset of ASD symptoms typically occurs by age 3 years, but may not fully manifest until school age or later.^25^ Additional information about the GBD method for identification and classification of ASD, which is applicable to ADHD, can be found in an earlier report by Baxter and colleagues.^26^ Although the GBD estimates are quite conservative, our findings underscore the need for intervention in some infants with ASD and ADHD from early childhood.

Identification of the major causes, as well as the effects, of developmental disabilities should be an urgent priority in regions with the largest prevalence and absolute burden for several reasons.^9^, ^17^, ^27^, ^28^ First, most of the countries in those regions lack functional health-care and social-care systems to support children with disabilities. Second, the lifetime costs of supporting children with disabilities are substantial and might further impoverish many poor families and communities. Third, societal stigma and unfavourable cultural beliefs place children at great risk of neglect, violence, or even infanticide. Fourth, educational and vocational opportunities are limited and rarely allow full economic participation and independence of children with developmental disabilities when they transition into adulthood. A range of interventions for children with or at risk of developmental disabilities are well described in the literature,^8^, ^9^, ^17^, ^29^ and include primary prevention aimed at reducing the incidence of developmental disabilities, secondary prevention through early detection of disabilities at a time when the brain is still very sensitive to interventions and change, and tertiary prevention through comprehensive community-based rehabilitation programmes (now termed community-based inclusive development), as exemplified by some early childhood development initiatives in high-burden LMICs such as Bangladesh and India.^30^, ^31^ Some disabilities might be less amenable than others to prevention. For example, the GBD study did not investigate the causes of ASD and ADHD, which are often attributed to complex interactions between genetic and environmental factors.^32^ However, several risk factors amenable to intervention have been reported in the literature and merit attention and efforts to equip local health and educational systems to provide requisite services for the affected children and their families.^33^ The newly launched Nurturing Care Framework for Early Childhood Development by WHO and its partners to facilitate optimal early childhood development in LMICs^34^ should necessarily also prioritise actions to address the specific needs of children with or at risk of developmental disabilities under the SDG era, especially in nations with a high burden.^35^ A cursory or symbolic reference to developmental disabilities in this framework will not be adequate or effective in galvanising the required attention by policy makers for the affected children and their families.

The disability-inclusive aspirations of the SDGs agenda are reinforced by the UN's Conventions on the Rights of the Child and the Rights of Persons with Disabilities. Since 2016, the GBD study has introduced a component for monitoring health-related targets and indicators periodically under the SDGs. Presently, 50 health-related indicators that directly involve health services, health outcomes, and risk factors with well established causal links have been identified, of which 37 are monitored in GBD 2016.^36^ Children with developmental disabilities are not yet captured within this framework because SDG 4 (to ensure inclusive and equitable quality education and promote lifelong learning opportunities for all) is completely omitted from the considered health-related indicators. Moreover, developmental disabilities have not yet been explicitly linked with SDG 3, which seeks to ensure healthy lives and promote wellbeing at all ages. It is hoped that the growing evidence on health-related causes of developmental disabilities reported in this study, and elsewhere, will facilitate the development of specific and explicit health-related indicators to address the special needs of the affected children.^37^, ^38^

Similar to any modelling endeavour in epidemiology, GBD 2016 has limitations that have been extensively described elsewhere in line with the GATHER reporting guidelines.^13^, ^14^ For example, most of the uncertainty in the YLD estimates are likely to result from limitations in the calculations of disability weights. These uncertainties might be minimised in the future by removing some of the ambiguities in lay descriptions, providing better definitions for disorders in surveys, and increasing the general volume and quality of survey data in this area. Additionally, the non-fatal models, especially for conditions such as ASD and ADHD, continue to rely on sparse data in many regions and exemplify the unique challenges associated with measuring disability in childhood, particularly for more subtle and difficult to diagnose conditions such as ASD and ADHD.^38^, ^39^ Ultimately, a measure of functional limitations consistent with ICF is needed to complement the YLDs for developmental disabilities in childhood.

Additional limitations of this study deserve mention. First, direct and separate prevalence and YLDs estimates for other disabilities, such as cerebral palsy, communication, and motor disorders, were not provided, which will have resulted in underestimation of the overall number of children with developmental disabilities. There is no evidence to suggest, for example, that there has been any improvement in the global burden of cerebral palsy between 1990 and 2016.^40^, ^41^ Second, age-standardised prevalence estimates and severity patterns of impairments were not reported in this analysis but are freely available at the Global Health Data Exchange for download and interactive visualisation for all the 195 countries and territories in GBD 2016. Third, we did not estimate the incidence of developmental delays and disabilities. Finally, it was difficult to completely and precisely account for children with multiple disabilities and across multiple developmental domains. These overlaps were partially captured by estimation of prevalence of sequelae (most children with ASD also have intellectual disability), ensuring the total number of cases of each impairment equals the sum across all GBD causes, and accounting for the overlap between epilepsy, blindness, and intellectual disability. Therefore, the sum of the impairments and causes is not equal to the total number of children with developmental disability. Some residual overlap is certainly likely, however, especially for hearing and vision loss. We believe that the findings in the GBD study provide valuable baseline data for action and further refinements as more and better primary input sources become available for all developmental disabilities. This viewpoint is without prejudice to other ICF-oriented approaches to quantifying the actual burden of developmental disabilities at the family and societal levels. The annual updating of GBD allows for expedient incorporation of such insights and data as they become available. Additionally, because future GBD iterations will continue to refine the methods, incorporate new data sources, and reanalyse the entire time series, our estimates for 1990–2016 might not be identical to those in subsequent GBD reports.

In conclusion, although the burden of mortality among children younger than 5 years has been halved between 1990 and 2016, there has been no corresponding improvement in non-fatal health outcomes among children with developmental disabilities globally. This lack of improvement might be attributed to absent or inadequate systematic policies and interventions to address the needs of survivors of childhood illnesses who develop life-long disabilities from early childhood, especially in sub-Saharan Africa. This report underscores the importance of developing explicit health-related indicators for monitoring progress to address the needs and rights of children with or at risk of developmental disabilities within the framework of the disability-inclusive mandates of the SDGs and beyond. More crucially, local health and educational systems should be appropriately equipped to support affected children and their families optimally.
