# Correction to *Lancet Glob Health* 2018; 6: e1100–21

**DOI:** 10.1016/S2214-109X(18)30443-1

**Published:** 2018-09-17

*Global Research on Developmental Disabilities Collaborators. Developmental disabilities among children younger than 5 years in 195 countries and territories, 1990–2016: a systematic analysis for the Global Burden of Disease Study 2016.* Lancet Glob Health *2018; **6**: e1100–21*—The following sentence should have been included in the Acknowledgments section of this Article: “We acknowledge the contribution of Charles R J Newton (University of Oxford, Oxford, UK) to an earlier version of this Article.”. This correction has been made as of Sept 17, 2018.

