# Supplementary material for: Developmental disabilities among children younger than 5 years in 195 countries and territories, 1990–2016: a systematic analysis for the Global Burden of Disease Study 2016
Source: Lancet Glob Health. 2018 Aug 29;6(10):e1100–21. doi: 10.1016/S2214-109X(18)30309-7 (PMC6139259; doi:10.1016/S2214-109X(18)30309-7)
Supplement: Supplementary appendix [file mmc1.pdf]

THE LANCET
Global Health

Supplementary appendix

This appendix formed part of the original submission and has been peer reviewed. We post it as supplied by the authors.

Supplement to: Global Research on Developmental Disabilities Collaborators. Developmental disabilities among children younger than 5 years in 195 countries and territories, 1990–2016: a systematic analysis for the Global Burden of Disease Study 2016. *Lancet Glob Health* 2018; published online Aug 29. http://dx.doi.org/10.1016/S2214-109X(18)30309-7.

Table S1. Global cause attribution of developmental disorders modelled as impairments in the GBD 2016 based on ICD-9 and ICD-10 codes

Epilepsy		Intellectual Disability		Hearing Loss		Vision Loss	
	No of cases		No of cases		No of cases		No of cases
**Neurological disorders**		**Congenital birth defects**		**Sense organ diseases**		**Sense organ diseases:**	
Epilepsy and seizures [**ICD10**: G40-G41.9, Z82.0; **ICD9**: 345-345.91]	**1,688,524.15**	Congenital heart anomalies [ICD10: Q20-Q27, Q27.1-Q28.9; **ICD9**: 745-747.9]	3,170,535.57	Age-related and other hearing loss [**ICD10**: H71-H75.83, H80-H80.93, H83-H83.93, H90-H91, H91.1-H91.93, H94-H94.83; **ICD9**: 384-385.9, 387-387.9, 388.1-388.2, 389-389.9]	**3,304,304.41**	Refraction and accommodation disorders [**ICD10**: H52-H52.7; **ICD9**: 367-367.9]	21,486,933.69
**Neonatal disorders**		Down syndrome [**ICD10**: Q90-Q90.9; **ICD9**: 758.0]	242,867.92	Otitis media [**ICD10**: H65-H70.93; **ICD9**: 381-383.9]	**8,829,401.26**	Other vision loss [**ICD10**: H27-H27.9, H31-H35.23, H35.4-H36.8, H46-H51.9, H53-H54.9; **ICD9**: 360.8-362.43, 362.6-363.9, 368-369.9, 377-378.9]	491,838.77
Neonatal sepsis and other neonatal infections [**ICD10**: P36-P36.9, P38-P39.9; **ICD9**: 771, 771.4-771.89]	1,046,598.56	Other chromosomal abnormalities [**ICD10**: Q91-Q93.9, Q95, Q95.2-Q95.9, Q97-Q97.9, Q99-Q99.9; **ICD9**: 758, 758.1-758.2, 758.4-758.5, 758.8-758.9]	1,258,075.67	Congenital birth defects [**ICD10**: P96.0, Q00-Q07.9, Q10-Q18.9, Q20-Q28.9, Q30-Q45.9, Q50-Q61.9, Q63-Q64.19, Q65-Q87.89, Q89-Q89.8, Q90-Q93.9, Q95-Q99.9, Z13.7-Z13.79, Z14-Z15.89, Z82.7-Z82.79, Z87.7-Z87.798; **ICD9**: 237.7-237.79, 740-753.19, 753.3, 753.5, 754.3-759.9, V13.6-V13.69, V18.61 V18.9, V19.5-V19.8, V82.3]	**3,254,446.81**		**21,978,772.46**
Neonatal preterm birth complications [**ICD10**: P07.2-P07.39, P22-P22.9, P25-P28.9, P61.2, P77-P77.9; **ICD9**: 765.21-765.9, 769-770, 770.2-770.9, 776.6, 777.5-777.53]	698,789.15	Neural tube defects [**ICD10**: Q00-Q01.9, Q05-Q05.9, Q07.01, Q07.03; **ICD9**: 740-741.93, 742.0]	202,573.68	Meningitis [**ICD10**: A39-A39.9, A87-A87.9, D86.81, G00-G03.9, G06-G09.9, Z20.811, Z22.31; **ICD9**: 036-036.9, 047-049.9, 054.72, 320-320.3, 320.5-322.9, 324-326.9, V01.84]	**63,852.81**	Vitamin A deficiency [**ICD10**: E50-E50.9, E64.1; **ICD9**: 264-264.9]	**1,369,894.67**
Neonatal encephalopathy due to birth asphyxia and trauma [**ICD10**: P02-P03.9, P10-P15.9, P20-P21.9, P24-P24.9, P90-P91.9; **ICD9**: 761.7-763.9, 767-768, 768.2-768.9, 779.0- 779.2]	152,712.73	Klinefelter syndrome [**ICD10**: Q98-Q98.9; **ICD9**: 758.7]	101,174.64	**ALL CAUSES**	**15,452,005.29**	**Neonatal disorders**	
Hemolytic disease and other neonatal jaundice [**ICD10**: P55-P59.9; **ICD9**: 773-774.9]	59,849.97	Digestive congenital anomalies [**ICD10**: Q38-Q38.0, Q38.3-Q38.4, Q38.6-Q43, Q43.1-Q45.8, Q79.0-Q79.59; **ICD9**: 750-751, 751.1-751.9, 756.6-756.79]	6,709.30			Neonatal sepsis and other neonatal infections [**ICD10**: P36-P36.9, P38-P39.9; **ICD9**: 771, 771.4-771.89]	890,895.18
	**1,957,950.40**		**4,981,936.79**			Neonatal preterm birth complications [**ICD10**: P07.2-P07.39, P22-P22.9, P25-P28.9, P61.2, P77-P77.9; **ICD9**: 765.21-765.9, 769-770, 770.2-770.9, 776.6, 777.5-777.53]	800,143.14
Meningitis [**ICD10**: A39-A39.9, A87-A87.9, D86.81, G00-G03.9, G06-G09.9, Z20.811, Z22.31; **ICD9**: 036-036.9, 047-049.9, 054.72, 320-320.3, 320.5-322.9, 324-326.9, V01.84]	93,416.35	**Neonatal disorders**				Neonatal encephalopathy due to birth asphyxia and trauma [**ICD10**: P02-P03.9, P10-P15.9, P20-P21.9, P24-P24.9, P90-P91.9; **ICD9**: 761.7-763.9, 767-768, 768.2-768.9, 779.0-779.2]	26,791.30
Malaria [**ICD10**: B50-B50.0, B50.8-B52.0, B52.8-B53.1, B53.8-B54.0, P37.3-P37.4; **ICD9**: 084-084.9]	33,291.44	Neonatal sepsis and other neonatal infections [**ICD10**: P36-P36.9, P38-P39.9; **ICD9**: 771, 771.4-771.89]	1,118,018.95			Hemolytic disease and other neonatal jaundice [**ICD10**: P55-P59.9; **ICD9**: 773-774.9]	11,310.72
Encephalitis [**ICD10**: A83-A85.2, A85.8-A86.0, B94.1, F07.1, G04-G05.8, Z24.1; **ICD9**: 062-064.9, 310.89, 323-323.9, V05.0-V05.1]	33,271.59	Neonatal preterm birth complications [**ICD10**: P07.2-P07.39, P22-P22.9, P25-P28.9, P61.2, P77-P77.9; **ICD9**: 765.21-765.9, 769-770, 770.2-770.9, 776.6, 777.5-777.53]	1,111,132.73				**1,729,140.34**
Zika virus [**ICD10**: U06-U06.9; **ICD9**: 066.3]	2,394.02	Neonatal encephalopathy due to birth asphyxia and trauma [**ICD10**: P02-P03.9, P10-P15.9, P20-P21.9, P24-P24.9, P90-P91.9; **ICD9**: 761.7-763.9, 767-768, 768.2-768.9, 779.0-779.2]	371,874.10			Meningitis [**ICD10**: A39-A39.9, A87-A87.9, D86.81, G00-G03.9, G06-G09.9, Z20.811, Z22.31; **ICD9**: 036-036.9, 047-049.9, 054.72, 320-320.3, 320.5-322.9, 324-326.9, V01.84]	99,037.45
Cystic echinococcosis [**ICD10**: B67-B67.4, B67.8-B67.99; **ICD9**: 122-122.4, 122.8-122.9]	406.71	Hemolytic disease and other neonatal jaundice [**ICD10**: P55-P59.9; **ICD9**: 773-774.9]	27,707.98			Encephalitis [**ICD10**: A83-A85.2, A85.8-A86.0, B94.1, F07.1, G04-G05.8, Z24.1; **ICD9**: 062-064.9, 310.89, 323-323.9, V05.0-V05.1]	39,521.45
Food-borne trematodiases [**ICD10**: B66-B66.9, B72.0; **ICD9**: 121-121.9]	283.28		**2,628,733.76**			Malaria [**ICD10**: B50-B50.0, B50.8-B52.0, B52.8-B53.1, B53.8-B54.0, P37.3-P37.4; **ICD9**: 084-084.9]	6,225.44
Tetanus [**ICD10**: A33-A35.0, Z23.5; **ICD9**: 037-037.9, 771.3, V03.7]	18.92	Meningitis [**ICD10**: A39-A39.9, A87-A87.9, D86.81, G00-G03.9, G06-G09.9, Z20.811, Z22.31; **ICD9**: 036-036.9, 047-049.9, 054.72, 320-320.3, 320.5-322.9, 324-326.9, V01.84]	226,348.77			Tetanus [**ICD10**: A33-A35.0, Z23.5; **ICD9**: 037-037.9, 771.3, V03.7]	3.53
	**69,665.97**	Encephalitis [**ICD10**: A83-A85.2, A85.8-A86.0, B94.1, F07.1, G04-G05.8, Z24.1; **ICD9**: 062-064.9, 310.89, 323-323.9, V05.0-V05.1]	62,894.60				**144,787.87**
**ALL CAUSES**	**3,809,556.87**	Iodine deficiency [**ICD10**: E00-E02; **ICD9**: 244.2]	17,262.55				
		Malaria [**ICD10**: B50-B50.0, B50.8-B52.0, B52.8-B53.1, B53.8-B54.0, P37.3-P37.4; **ICD9**: 084-084.9]	15,329.97			**ALL CAUSES**	**25,222,595.34**
		Zika virus [**ICD10**: U06-U06.9; **ICD9**: 066.3]	11,814.44				
		Tetanus [**ICD10**: A33-A35.0, Z23.5; **ICD9**: 037-037.9, 771.3, V03.7]	7,186.57				
			**340,836.89**				
		Autistic spectrum disorders [**ICD10**: F84-F84.9; **ICD9**: 299-299.91]	**925,073.95**				
		Alcohol use disorders [**ICD10**: F10-F10.99, G31.2, R78.0, X45-X45.9, X65-X65.9, Y15-Y15.9, Z81.1; **ICD9**: 291-291.9, 303-303.93, 305-305.03, 790.3, E860-E860.19, V11.3]	**23,701.43**				
		Idiopathic developmental intellectual disability [**ICD10**: F70-F79.9, Z81.0; **ICD9**: 317-319.9, V18.4]	**3,633,948.49**				
		**ALL CAUSES**	**12,534,231.32**				

Table S2. Global and country-specific prevalence of developmental disabilities in 2016 (with 95% uncertainty interval)																				
			Epilepsy			Intellectual disability			Hearing loss			Vision loss			Autism spectrum disorder			Attention-deficit/hyperactivity disorder		
Loc-ID	Location	Metric	Value	Upper limit	Lower limit	Value	Upper limit	Lower limit	Value	Upper limit	Lower limit	Value	Upper limit	Lower limit	Value	Upper limit	Lower limit	Value	Upper limit	Lower limit
1	Global	Number	3,809,556.87	5,199,795.75	3,023,986.21	12,534,231.32	15,146,762.85	10,183,368.42	15,452,005.29	17,650,431.78	13,352,921.73	25,222,595.34	28,355,185.45	22,718,169.23	4,567,674.82	5,480,484.55	3,755,747.03	890,229.45	1,022,156.83	794,104.47
1	Global	Rate	602.81	822.79	478.50	1,983.36	2,396.75	1,611.37	2,445.05	2,792.92	2,112.90	3,991.10	4,486.79	3,594.81	722.77	867.21	594.29	140.87	161.74	125.66
6	China	Number	221,717.45	271,597.10	179,393.75	754,768.14	890,469.54	630,839.02	1,256,628.17	1,475,629.71	1,060,777.02	1,957,057.69	2,166,437.25	1,777,600.55	422,163.85	513,128.48	346,690.31	132,059.55	147,966.90	117,961.91
6	China	Rate	365.52	447.75	295.75	1,244.30	1,468.02	1,039.99	2,071.66	2,432.71	1,748.79	3,226.38	3,571.57	2,930.53	695.97	845.94	571.55	217.71	243.94	194.47
7	North Korea	Number	10,300.89	14,468.91	6,067.55	40,006.41	48,392.68	32,760.07	80,099.84	91,690.76	69,089.13	120,772.34	132,324.95	110,946.42	19,902.58	24,687.43	15,750.11	6,109.51	7,078.43	5,302.28
7	North Korea	Rate	348.12	488.98	205.05	1,352.03	1,635.45	1,107.14	2,707.00	3,098.72	2,334.89	4,081.54	4,471.96	3,749.47	672.61	834.32	532.28	206.47	239.22	179.19
8	Taiwan	Number	3,634.18	5,378.94	1,767.56	5,623.98	6,576.26	4,786.16	25,974.37	29,986.21	22,568.32	24,549.60	27,559.10	22,048.94	6,991.40	8,638.83	5,619.83	2,139.11	2,477.32	1,854.02
8	Taiwan	Rate	358.38	530.43	174.30	554.60	648.50	471.98	2,561.41	2,957.03	2,225.53	2,420.91	2,717.68	2,174.31	689.44	851.90	554.19	210.94	244.30	182.83
10	Cambodia	Number	10,322.60	15,372.79	5,574.53	29,692.47	37,568.40	23,471.30	38,992.77	46,097.02	32,408.77	80,607.43	97,557.75	65,504.38	13,021.38	16,169.36	10,409.16	1,596.74	2,112.17	1,191.00
10	Cambodia	Rate	547.97	816.05	295.92	1,576.20	1,994.29	1,245.95	2,069.90	2,447.02	1,720.39	4,278.97	5,178.77	3,477.24	691.23	858.34	552.56	84.76	112.12	63.22
11	Indonesia	Number	124,866.94	158,320.17	97,052.70	282,766.88	339,352.65	233,345.46	436,961.07	512,784.02	368,857.40	957,552.15	1,049,048.02	876,671.40	159,285.31	193,284.20	130,677.09	19,274.19	22,599.28	16,454.15
11	Indonesia	Rate	549.44	696.63	427.05	1,244.22	1,493.21	1,026.76	1,922.70	2,256.33	1,623.03	4,213.39	4,615.98	3,857.50	700.88	850.48	575.00	84.81	99.44	72.40
12	Laos	Number	4,847.31	7,568.75	2,593.23	16,837.64	21,092.48	13,335.08	23,188.79	27,435.80	19,224.27	27,256.12	30,894.80	24,235.65	7,751.51	9,694.81	6,234.54	944.88	1,249.81	704.96
12	Laos	Rate	428.84	669.61	229.42	1,489.63	1,866.06	1,179.76	2,051.52	2,427.26	1,700.78	2,411.36	2,733.28	2,144.14	685.78	857.70	551.57	83.59	110.57	62.37
13	Malaysia	Number	15,235.55	21,682.64	8,589.66	22,703.25	29,392.01	17,492.11	34,400.86	42,644.63	27,422.68	54,233.98	62,930.21	47,000.36	17,979.57	22,231.26	14,416.35	1,653.25	1,936.70	1,425.43
13	Malaysia	Rate	593.36	844.45	334.53	884.20	1,144.70	681.25	1,339.77	1,660.84	1,068.00	2,112.20	2,450.88	1,830.47	700.23	865.82	561.46	64.39	75.43	55.51
14	Maldives	Number	165.65	236.68	85.63	324.85	420.47	252.15	590.88	703.70	491.49	1,003.66	1,232.61	791.80	221.33	271.30	179.46	27.35	36.19	20.36
14	Maldives	Rate	528.51	755.15	273.20	1,036.45	1,341.52	804.48	1,885.22	2,245.19	1,568.11	3,202.22	3,932.69	2,526.27	706.17	865.60	572.58	87.25	115.47	64.95
15	Myanmar	Number	24,403.37	35,637.54	13,022.91	64,716.82	82,962.46	50,332.21	118,004.36	135,380.71	103,198.70	167,227.30	193,686.21	144,699.99	32,088.49	39,944.52	25,790.96	3,974.89	5,257.10	2,965.91
15	Myanmar	Rate	523.95	765.16	279.61	1,389.50	1,781.24	1,080.66	2,533.61	2,906.69	2,215.73	3,590.45	4,158.54	3,106.78	688.96	857.63	553.74	85.34	112.87	63.68
16	Philippines	Number	61,435.70	91,048.12	33,324.75	148,331.25	190,078.79	115,261.98	219,952.85	260,765.63	182,431.53	636,076.02	692,658.39	584,103.45	80,346.25	99,034.35	64,371.13	9,800.42	12,965.78	7,306.31
16	Philippines	Rate	531.12	787.12	288.09	1,282.33	1,643.24	996.45	1,901.51	2,254.34	1,577.13	5,498.92	5,988.08	5,049.61	694.60	856.16	556.49	84.73	112.09	63.16
17	Sri Lanka	Number	11,125.58	15,224.55	6,427.06	17,132.64	22,030.30	13,059.74	31,069.72	36,159.83	26,712.14	56,018.43	64,363.48	49,539.75	10,570.20	13,174.73	8,473.82	1,313.44	1,737.39	979.74
17	Sri Lanka	Rate	736.08	1,007.27	425.22	1,133.51	1,457.54	864.04	2,055.59	2,392.36	1,767.29	3,706.22	4,258.33	3,277.58	699.33	871.65	560.63	86.90	114.95	64.82
18	Thailand	Number	19,834.29	27,763.85	11,708.61	47,922.49	59,310.16	37,281.33	60,854.15	71,504.47	51,743.25	71,623.67	88,071.90	56,861.44	21,745.36	26,845.06	17,453.25	3,688.75	4,290.09	3,198.45
18	Thailand	Rate	641.09	897.40	378.45	1,548.97	1,917.05	1,205.02	1,966.96	2,311.20	1,672.47	2,315.05	2,846.70	1,837.90	702.86	867.70	564.13	119.23	138.67	103.38
19	Timor-Leste	Number	779.86	1,181.88	391.40	2,293.85	2,931.44	1,780.22	3,357.65	3,989.53	2,826.70	7,331.50	8,838.67	5,955.57	1,146.33	1,423.29	919.28	141.60	187.31	105.61
19	Timor-Leste	Rate	471.13	713.99	236.45	1,385.75	1,770.93	1,075.46	2,028.40	2,410.13	1,707.65	4,429.07	5,339.58	3,597.85	692.52	859.83	555.35	85.54	113.16	63.80
20	Vietnam	Number	35,738.14	51,826.96	19,944.54	70,228.99	82,453.13	59,959.17	135,089.48	159,475.95	114,026.32	204,945.98	238,737.92	179,180.34	53,375.00	65,914.77	42,744.65	6,533.45	8,647.93	4,861.78
20	Vietnam	Rate	473.13	686.12	264.04	929.74	1,091.57	793.78	1,788.41	2,111.25	1,509.56	2,713.22	3,160.58	2,372.11	706.62	872.63	565.88	86.49	114.49	64.36
22	Fiji	Number	231.41	323.70	140.54	552.93	659.01	458.39	1,050.37	1,216.18	887.89	1,681.40	1,931.83	1,486.55	353.48	433.53	279.04	64.67	75.89	55.75
22	Fiji	Rate	455.22	636.77	276.48	1,087.71	1,296.39	901.74	2,066.27	2,392.44	1,746.64	3,307.62	3,800.27	2,924.32	695.35	852.84	548.91	127.21	149.29	109.67
23	Kiribati	Number	54.07	77.62	32.49	219.85	257.45	185.16	331.41	378.07	286.08	595.40	658.91	540.17	93.86	116.01	75.02	17.03	19.98	14.68
23	Kiribati	Rate	400.04	574.25	240.32	1,626.41	1,904.58	1,369.81	2,451.71	2,796.93	2,116.41	4,404.72	4,874.54	3,996.10	694.34	858.23	555.01	125.95	147.81	108.58
24	Marshall Islands	Number	41.32	57.66	25.91	137.85	168.41	113.47	223.05	256.26	191.71	371.75	416.29	333.77	68.58	84.19	54.98	12.42	14.57	10.70
24	Marshall Islands	Rate	417.05	581.98	261.46	1,391.23	1,699.68	1,145.24	2,251.17	2,586.36	1,934.82	3,751.92	4,201.42	3,368.64	692.17	849.70	554.86	125.31	147.05	108.01
25	Federated States of Micronesia	Number	40.32	54.73	25.46	131.10	154.43	110.04	211.79	244.06	180.61	348.55	390.91	312.19	66.76	83.11	53.66	12.08	14.17	10.41
25	Federated States of Micronesia	Rate	421.29	571.82	266.00	1,369.71	1,613.50	1,149.73	2,212.77	2,549.95	1,887.04	3,641.60	4,084.26	3,261.72	697.50	868.30	560.65	126.16	148.06	108.77
26	Papua New Guinea	Number	3,761.91	5,473.97	2,231.53	17,109.57	20,057.72	14,396.29	26,761.48	30,455.11	23,218.87	52,762.47	58,668.05	47,588.79	7,506.32	9,471.36	5,971.80	1,345.78	1,579.42	1,160.31
26	Papua New Guinea	Rate	349.10	507.98	207.08	1,587.74	1,861.33	1,335.96	2,483.43	2,826.19	2,154.68	4,896.28	5,444.31	4,416.17	696.58	878.93	554.17	124.89	146.57	107.67
27	Samoa	Number	116.24	160.43	71.58	312.35	369.57	259.43	625.99	720.99	536.92	1,049.48	1,177.36	943.58	192.53	242.36	153.73	34.98	41.05	30.15
27	Samoa	Rate	423.03	583.84	260.49	1,136.72	1,344.94	944.12	2,278.09	2,623.83	1,953.96	3,819.26	4,284.66	3,433.89	700.66	882.01	559.47	127.29	149.40	109.73
28	Solomon Islands	Number	348.26	473.45	219.55	1,325.26	1,564.50	1,114.43	2,048.13	2,338.22	1,763.85	3,664.33	4,056.43	3,312.75	583.28	728.97	464.69	105.24	123.51	90.73
28	Solomon Islands	Rate	417.94	568.18	263.48	1,590.44	1,877.54	1,337.42	2,457.94	2,806.07	2,116.78	4,397.53	4,868.08	3,975.60	699.98	874.83	557.67	126.30	148.22	108.89
29	Tonga	Number	59.24	82.71	36.94	162.64	193.09	135.33	303.56	348.12	259.81	461.37	513.99	415.23	93.44	116.33	74.66	17.13	20.10	14.76
29	Tonga	Rate	440.16	614.56	274.43	1,208.44	1,434.64	1,005.47	2,255.47	2,586.52	1,930.37	3,428.00	3,818.94	3,085.16	694.23	864.30	554.70	127.26	149.35	109.70
30	Vanuatu	Number	158.05	222.37	99.20	575.02	672.36	484.65	970.62	1,108.27	840.37	1,652.14	1,831.72	1,496.39	283.41	350.36	225.94	50.98	59.83	43.95
30	Vanuatu	Rate	389.54	548.07	244.50	1,417.23	1,657.15	1,194.50	2,392.25	2,731.50	2,071.22	4,071.97	4,514.57	3,688.08	698.51	863.51	556.86	125.64	147.45	108.32
33	Armenia	Number	1,131.55	1,776.49	621.83	3,642.73	4,311.93	3,062.17	4,430.05	5,165.28	3,752.07	8,922.59	10,179.32	7,912.38	1,614.09	2,011.53	1,282.29	289.44	338.56	251.79
33	Armenia	Rate	511.16	802.49	280.90	1,645.54	1,947.83	1,383.28	2,001.19	2,333.32	1,694.93	4,030.62	4,598.32	3,574.27	729.14	908.67	579.25	130.75	152.94	113.74
34	Azerbaijan	Number	4,370.69	7,327.55	2,006.99	13,878.16	16,137.50	11,895.14	19,052.49	22,182.95	16,063.52	37,162.64	42,591.30	32,705.27	6,966.70	8,725.21	5,592.66	1,265.93	1,480.92	1,101.39
34	Azerbaijan	Rate	456.84	765.90	209.78	1,450.59	1,686.74	1,243.32	1,991.43	2,318.63	1,679.01	3,884.36	4,451.78	3,418.46	728.18	911.99	584.56	132.32	154.79	115.12
35	Georgia	Number	1,594.43	2,483.55	832.16	5,281.09	6,129.60	4,521.59	6,716.10	7,802.89	5,688.99	13,430.67	15,341.05	11,758.87	2,405.80	2,994.47	1,929.66	437.41	511.49	380.36
35	Georgia	Rate	478.52	745.37	249.75	1,584.96	1,839.62	1,357.02	2,015.64	2,341.81	1,707.38	4,030.82	4,604.17	3,529.08	722.03	898.70	579.13	131.27	153.51	114.15
36	Kazakhstan	Number	9,109.86	14,293.80	5,163.57	27,665.79	32,318.91	23,656.30	38,897.76	45,299.70	32,810.23	76,719.24	88,032.47	67,860.11	14,093.74	17,650.91	11,197.19	2,539.89	2,969.35	2,207.58
36	Kazakhstan	Rate	464.99	729.60	263.56	1,412.14	1,649.65	1,207.49	1,985.45	2,312.23	1,674.73	3,915.97	4,493.43	3,463.78	719.39	900.95	571.54	129.64	151.56	112.68
37	Kyrgyzstan	Number	3,208.40	4,985.00	1,797.38	15,151.13	17,578.72	12,885.42	15,956.03	18,423.64	13,643.66	33,347.00	37,471.18	29,848.59	5,350.19	6,632.61	4,239.40	967.44	1,130.99	840.80
37	Kyrgyzstan	Rate	429.12	666.74	240.40	2,026.44	2,351.13	1,723.41	2,134.10	2,464.14	1,824.82	4,460.12	5,011.72	3,992.21	715.58	887.10	567.01	129.39	151.27	112.46
38	Mongolia	Number	1,677.33	2,616.41	885.35	6,469.65	7,438.38	5,549.29	7,666.08	8,908.50	6,509.67	15,658.80	17,754.47	14,015.88	2,626.78	3,264.40	2,096.35	476.09	556.51	413.62

Table S2. Global and country-specific prevalence of developmental disabilities in 2016 (with 95% uncertainty interval)																				
			Epilepsy			Intellectual disability			Hearing loss			Vision loss			Autism spectrum disorder			Attention-deficit/hyperactivity disorder		
Loc-ID	Location	Metric	Value	Upper limit	Lower limit	Value	Upper limit	Lower limit	Value	Upper limit	Lower limit	Value	Upper limit	Lower limit	Value	Upper limit	Lower limit	Value	Upper limit	Lower limit
38	Mongolia	Rate	450.51	702.74	237.80	1,737.68	1,997.87	1,490.48	2,059.03	2,392.73	1,748.43	4,205.79	4,768.67	3,764.52	705.53	876.78	563.06	127.87	149.47	111.09
39	Tajikistan	Number	5,030.62	7,759.96	2,795.13	25,370.51	29,271.65	21,599.52	24,766.55	28,532.25	21,159.63	53,912.62	60,464.78	48,280.98	8,066.75	10,012.92	6,410.85	1,446.97	1,691.49	1,257.36
39	Tajikistan	Rate	441.86	681.59	245.51	2,228.41	2,571.07	1,897.19	2,175.36	2,506.12	1,858.55	4,735.40	5,310.91	4,240.74	708.54	879.48	563.10	127.09	148.57	110.44
40	Turkmenistan	Number	2,516.74	4,097.27	1,269.70	9,072.93	10,515.39	7,840.28	11,440.79	13,300.50	9,707.00	22,563.17	25,728.88	19,935.54	4,101.07	5,089.13	3,262.04	742.16	867.57	644.88
40	Turkmenistan	Rate	435.77	709.44	219.85	1,570.97	1,820.74	1,357.54	1,980.97	2,302.98	1,680.76	3,906.81	4,454.95	3,451.83	710.10	881.18	564.82	128.50	150.22	111.66
41	Uzbekistan	Number	15,201.89	23,454.22	8,236.26	59,657.34	69,352.22	50,665.45	68,752.41	79,691.23	58,565.63	139,442.84	158,028.68	123,310.64	23,753.29	29,357.36	19,066.46	4,323.89	5,054.88	3,757.91
41	Uzbekistan	Rate	455.05	702.08	246.54	1,785.78	2,075.99	1,516.62	2,058.04	2,385.48	1,753.11	4,174.09	4,730.43	3,691.18	711.03	878.78	570.74	129.43	151.31	112.49
43	Albania	Number	866.50	1,406.99	426.79	2,607.69	3,048.27	2,215.87	3,734.20	4,332.04	3,149.99	6,540.28	8,220.96	5,138.41	1,349.73	1,669.53	1,075.03	238.29	278.60	207.16
43	Albania	Rate	467.62	759.31	230.32	1,407.30	1,645.07	1,195.84	2,015.24	2,337.88	1,699.96	3,529.61	4,436.62	2,773.06	728.41	901.00	580.16	128.60	150.35	111.80
44	Bosnia and Herzegovina	Number	735.64	1,115.65	397.33	2,490.34	2,915.50	2,110.39	3,483.87	4,043.70	2,968.88	6,925.85	7,760.29	6,121.76	1,251.72	1,553.90	999.15	225.59	263.74	196.09
44	Bosnia and Herzegovina	Rate	430.47	652.83	232.50	1,457.25	1,706.03	1,234.92	2,038.62	2,366.22	1,737.27	4,052.73	4,541.02	3,582.22	732.46	909.28	584.67	132.01	154.33	114.74
45	Bulgaria	Number	1,315.22	2,096.11	697.58	6,097.91	6,904.03	5,416.71	6,372.77	7,418.23	5,373.52	10,465.21	12,765.64	8,276.45	2,425.35	3,018.40	1,952.35	435.00	508.55	378.08
45	Bulgaria	Rate	395.40	630.16	209.72	1,833.25	2,075.59	1,628.45	1,915.88	2,230.18	1,615.47	3,146.21	3,837.80	2,488.19	729.15	907.44	586.94	130.78	152.89	113.66
46	Croatia	Number	698.38	1,130.26	363.28	3,331.10	3,780.55	2,964.72	3,765.12	4,392.43	3,160.90	5,264.45	6,155.78	4,524.74	1,436.92	1,772.78	1,148.10	258.01	301.63	224.25
46	Croatia	Rate	354.96	574.47	184.64	1,693.08	1,921.52	1,506.86	1,913.67	2,232.51	1,606.57	2,675.73	3,128.76	2,299.76	730.34	901.04	583.54	131.14	153.31	113.98
47	Czech Republic	Number	2,059.97	3,442.97	973.25	7,568.37	8,675.78	6,628.55	10,212.70	11,959.47	8,542.66	13,165.59	15,672.29	11,089.93	3,973.00	4,904.47	3,153.89	708.58	828.39	615.87
47	Czech Republic	Rate	378.14	632.01	178.66	1,389.28	1,592.57	1,216.77	1,874.69	2,195.33	1,568.13	2,416.73	2,876.88	2,035.72	729.30	900.29	578.94	130.07	152.06	113.05
48	Hungary	Number	1,781.83	2,883.68	939.13	7,034.31	7,995.53	6,303.16	8,595.69	10,061.85	7,207.61	11,707.78	13,667.40	10,099.34	3,294.59	4,069.87	2,627.36	588.49	687.99	511.50
48	Hungary	Rate	393.67	637.10	207.49	1,554.11	1,766.48	1,392.58	1,899.07	2,223.00	1,592.40	2,586.64	3,019.58	2,231.28	727.89	899.17	580.47	130.02	152.00	113.01
49	Macedonia	Number	435.44	678.66	223.01	1,712.84	1,996.84	1,477.44	2,207.28	2,571.15	1,861.32	3,728.61	4,602.68	2,950.52	813.82	1,010.94	646.80	146.66	171.45	127.46
49	Macedonia	Rate	387.98	604.68	198.71	1,526.14	1,779.19	1,316.40	1,966.69	2,290.90	1,658.44	3,322.21	4,101.00	2,628.92	725.11	900.75	576.30	130.67	152.77	113.57
50	Montenegro	Number	146.43	232.49	80.71	547.58	632.89	473.29	706.49	825.00	595.11	1,182.89	1,472.38	931.38	266.47	330.57	213.75	47.51	55.55	41.30
50	Montenegro	Rate	402.05	638.33	221.60	1,503.45	1,737.68	1,299.47	1,939.77	2,265.15	1,633.96	3,247.76	4,042.61	2,557.22	731.63	907.62	586.87	130.45	152.51	113.39
51	Poland	Number	5,758.56	9,083.08	3,022.63	26,690.32	30,536.90	23,500.80	35,327.47	41,435.34	29,617.21	92,977.01	102,426.85	84,423.96	13,667.33	16,901.00	10,941.84	2,433.95	2,845.47	2,115.45
51	Poland	Rate	308.05	485.89	161.69	1,427.77	1,633.54	1,257.15	1,889.80	2,216.54	1,584.34	4,973.70	5,479.21	4,516.16	731.12	904.10	585.32	130.20	152.22	113.16
52	Romania	Number	3,566.74	5,675.78	1,915.47	12,536.72	14,435.01	10,882.85	16,817.71	19,616.00	14,141.13	27,711.99	34,246.89	21,859.87	6,360.73	7,881.61	5,102.90	1,139.05	1,331.64	990.00
52	Romania	Rate	406.06	646.16	218.07	1,427.25	1,643.36	1,238.96	1,914.62	2,233.19	1,609.90	3,154.89	3,898.85	2,488.65	724.14	897.29	580.94	129.68	151.60	112.71
53	Serbia	Number	1,918.31	3,216.08	942.32	7,358.75	8,350.96	6,523.31	8,238.15	9,567.08	6,967.47	14,089.27	17,616.22	11,103.28	3,014.37	3,724.98	2,411.60	539.24	630.40	468.65
53	Serbia	Rate	463.68	777.37	227.77	1,778.71	2,018.54	1,576.77	1,991.27	2,312.49	1,684.13	3,405.56	4,258.08	2,683.81	728.61	900.38	582.91	130.34	152.38	113.28
54	Slovakia	Number	1,089.64	1,772.32	553.17	3,954.33	4,519.86	3,455.05	5,409.34	6,337.44	4,521.57	6,807.27	8,078.48	5,743.30	2,096.11	2,591.80	1,679.14	376.99	440.72	327.63
54	Slovakia	Rate	377.94	614.73	191.87	1,371.56	1,567.71	1,198.38	1,876.22	2,198.13	1,568.30	2,361.09	2,802.01	1,992.06	727.03	898.96	582.41	130.76	152.86	113.64
55	Slovenia	Number	394.50	644.20	198.75	1,578.10	1,791.89	1,399.70	1,964.79	2,310.83	1,644.91	2,625.35	3,112.46	2,250.36	772.84	951.75	609.07	138.62	162.06	120.48
55	Slovenia	Rate	372.61	608.45	187.72	1,490.51	1,692.44	1,322.02	1,855.75	2,182.58	1,553.62	2,479.65	2,939.72	2,125.46	729.95	898.92	575.27	130.93	153.06	113.79
57	Belarus	Number	2,015.96	2,956.94	1,272.80	9,008.68	10,301.87	7,829.20	11,157.17	13,019.74	9,364.89	16,278.65	19,515.80	13,714.48	4,190.25	5,170.61	3,327.63	747.03	873.35	649.31
57	Belarus	Rate	350.05	513.45	221.01	1,564.28	1,788.83	1,359.47	1,937.34	2,260.76	1,626.13	2,826.64	3,388.74	2,381.40	727.60	897.83	577.81	129.71	151.65	112.75
58	Estonia	Number	224.99	331.71	134.31	988.64	1,130.23	861.02	1,287.71	1,505.48	1,081.63	1,180.32	1,357.92	1,027.47	498.04	617.00	396.92	88.94	103.98	77.30
58	Estonia	Rate	328.28	483.99	195.96	1,442.49	1,649.07	1,256.27	1,878.84	2,196.58	1,578.16	1,722.16	1,981.29	1,499.14	726.67	900.24	579.13	129.77	151.72	112.79
59	Latvia	Number	329.57	497.41	197.81	1,881.90	2,100.13	1,694.89	2,008.39	2,350.26	1,696.92	3,449.09	3,982.48	3,065.16	767.39	949.68	615.96	135.38	158.26	117.66
59	Latvia	Rate	311.49	470.12	186.96	1,778.67	1,984.92	1,601.91	1,898.22	2,221.34	1,603.83	3,259.89	3,764.01	2,897.02	725.30	897.58	582.17	127.95	149.58	111.20
60	Lithuania	Number	503.24	765.59	307.05	2,239.34	2,567.39	1,947.44	2,844.54	3,325.77	2,376.79	4,024.11	4,794.01	3,396.54	1,094.37	1,356.41	876.20	195.52	228.57	169.92
60	Lithuania	Rate	334.82	509.36	204.29	1,489.88	1,708.14	1,295.67	1,892.53	2,212.70	1,581.33	2,677.32	3,189.55	2,259.79	728.11	902.45	582.95	130.08	152.07	113.05
61	Moldova	Number	1,009.23	1,621.60	611.60	4,549.97	5,294.27	3,879.58	4,658.80	5,393.31	3,948.20	7,506.74	8,935.56	6,386.89	1,638.00	2,032.72	1,298.02	292.29	341.72	254.06
61	Moldova	Rate	448.75	721.04	271.95	2,023.13	2,354.08	1,725.05	2,071.52	2,398.12	1,755.56	3,337.86	3,973.18	2,839.92	728.33	903.85	577.16	129.97	151.95	112.97
62	Russia	Number	35,359.59	52,110.21	21,317.32	131,429.31	153,963.23	111,671.78	180,587.92	211,051.17	151,765.01	343,496.71	386,915.51	306,922.86	68,095.82	85,034.27	53,932.80	12,147.28	14,201.09	10,557.73
62	Russia	Rate	377.08	555.71	227.33	1,401.57	1,641.87	1,190.87	1,925.80	2,250.66	1,618.43	3,663.07	4,126.09	3,273.04	726.18	906.81	575.14	129.54	151.44	112.59
63	Ukraine	Number	9,371.14	13,876.67	5,912.59	40,697.04	47,048.22	35,051.04	48,704.02	56,559.65	41,189.48	69,400.30	81,931.39	60,405.69	17,926.21	22,063.77	14,316.47	3,208.06	3,750.58	2,788.51
63	Ukraine	Rate	381.57	565.02	240.74	1,657.07	1,915.67	1,427.18	1,983.09	2,302.95	1,677.12	2,825.78	3,336.01	2,459.55	729.90	898.37	582.93	130.62	152.71	113.54
66	Brunei	Number	122.81	194.74	59.27	407.71	463.05	361.88	325.33	396.41	267.63	943.80	1,101.21	813.27	237.12	293.10	186.80	35.95	42.40	31.04
66	Brunei	Rate	372.43	590.58	179.75	1,236.43	1,404.26	1,097.45	986.60	1,202.15	811.63	2,862.19	3,339.54	2,466.34	719.10	888.85	566.49	109.02	128.57	94.14
67	Japan	Number	12,684.66	16,419.63	9,738.72	77,859.80	88,036.41	69,523.49	53,460.35	65,125.65	43,659.58	145,854.99	171,235.30	125,812.70	37,659.81	45,371.05	30,636.07	5,469.52	6,208.00	4,815.57
67	Japan	Rate	252.89	327.36	194.16	1,552.29	1,755.18	1,386.09	1,065.84	1,298.41	870.44	2,907.91	3,413.92	2,508.33	750.82	904.56	610.79	109.05	123.77	96.01
68	South Korea	Number	6,399.03	10,092.26	3,255.26	29,262.47	33,375.49	25,921.76	21,625.19	26,474.50	17,695.35	60,073.23	71,164.08	51,702.38	14,814.26	18,276.24	12,040.82	2,333.23	2,683.58	2,019.88
68	South Korea	Rate	300.85	474.49	153.05	1,375.78	1,569.15	1,218.71	1,016.71	1,244.70	831.95	2,824.35	3,345.79	2,430.79	696.49	859.26	566.10	109.70	126.17	94.97
69	Singapore	Number	479.44	754.68	229.03	1,722.93	1,947.68	1,536.06	1,734.79	2,116.17	1,412.11	4,952.82	5,868.00	4,263.92	1,279.96	1,578.03	1,018.25	194.18	229.05	167.65
69	Singapore	Rate	271.07	426.69	129.49	974.12	1,101.19	868.47	980.82	1,196.45	798.39	2,800.26	3,317.69	2,410.77	723.67	892.20	575.70	109.79	129.50	94.79
71	Australia	Number	3,903.39	5,959.11	2,141.06	20,120.71	22,642.96	18,000.71	16,266.90	19,937.87	13,238.92	34,231.55	39,828.96	29,981.13	11,438.17	13,809.87	9,354.61	4,888.26	5,721.06	4,193.43
71	Australia	Rate	261.88	399.80	143.64	1,349.91	1,519.12	1,207.67	1,091.35	1,337.64	888.20	2,296.61	2,672.14	2,011.44	767.39	926.51	627.60	327.96	383.83	281.34
72	New Zealand	Number	792.53	1,184.85	438.42	4,339.80	4,790.59	3,977.45	3,201.19	3,928.82	2,597.53	6,763.34	7,879.86	5,905.52	2,139.82	2,627.00	1,733.12	775.47	921.23	663.91
72	New Zealand	Rate	277.40	414.72	153.46	1,519.03	1,676.81	1,392.19	1,120.49	1,375.17	909.19	2,367.32	2,758.12	2,067.06	748.98	919.51	606.63	271.43	322.45	232.38

Table S2. Global and country-specific prevalence of developmental disabilities in 2016 (with 95% uncertainty interval)																				
			Epilepsy			Intellectual disability			Hearing loss			Vision loss			Autism spectrum disorder			Attention-deficit/hyperactivity disorder		
Loc-ID	Location	Metric	Value	Upper limit	Lower limit	Value	Upper limit	Lower limit	Value	Upper limit	Lower limit	Value	Upper limit	Lower limit	Value	Upper limit	Lower limit	Value	Upper limit	Lower limit
74	Andorra	Number	9.07	13.81	4.90	44.56	50.28	39.60	37.40	52.99	26.08	63.97	88.19	49.22	19.21	23.60	15.26	4.87	7.01	3.31
74	Andorra	Rate	296.95	452.02	160.37	1,458.68	1,645.75	1,296.34	1,224.27	1,734.61	853.83	2,093.94	2,886.68	1,611.22	628.70	772.63	499.61	159.39	229.51	108.28
75	Austria	Number	1,183.45	1,805.94	614.53	6,440.15	7,131.05	5,876.14	4,950.46	6,983.36	3,457.07	8,933.67	12,471.77	6,900.11	2,495.22	3,093.75	1,964.51	632.57	910.95	429.81
75	Austria	Rate	294.43	449.29	152.89	1,602.23	1,774.11	1,461.91	1,231.61	1,737.37	860.07	2,222.58	3,102.81	1,716.66	620.78	769.68	488.74	157.37	226.63	106.93
76	Belgium	Number	1,939.88	3,151.14	873.08	7,557.55	8,857.12	6,688.57	8,002.00	11,295.03	5,567.90	14,141.49	19,104.73	11,242.84	3,913.91	4,865.52	3,130.96	994.62	1,432.41	675.85
76	Belgium	Rate	306.96	498.62	138.15	1,195.87	1,401.51	1,058.37	1,266.20	1,787.27	881.04	2,237.68	3,023.04	1,779.01	619.32	769.90	495.43	157.38	226.66	106.94
77	Cyprus	Number	159.34	240.36	91.03	812.68	919.45	722.01	631.38	887.98	440.20	1,068.89	1,475.96	821.84	308.66	380.70	246.19	77.92	112.22	52.95
77	Cyprus	Rate	321.08	484.34	183.43	1,637.56	1,852.70	1,454.86	1,272.23	1,789.27	887.01	2,153.81	2,974.05	1,656.00	621.94	767.10	496.07	157.02	226.12	106.69
78	Denmark	Number	750.45	1,131.38	422.73	4,712.44	5,196.28	4,292.51	2,001.53	2,505.31	1,584.71	4,061.58	4,831.78	3,453.83	1,818.02	2,212.85	1,458.22	285.69	344.24	237.61
78	Denmark	Rate	260.22	392.30	146.58	1,634.01	1,801.78	1,488.41	694.02	868.70	549.49	1,408.33	1,675.39	1,197.60	630.39	767.29	505.63	99.06	119.36	82.39
79	Finland	Number	739.27	1,085.83	428.01	6,516.07	7,115.40	6,009.74	2,464.87	3,029.16	1,987.84	6,033.93	8,473.90	4,572.08	1,819.51	2,227.25	1,472.23	687.23	799.39	602.80
79	Finland	Rate	254.52	373.83	147.36	2,243.38	2,449.72	2,069.06	848.62	1,042.89	684.38	2,077.39	2,917.43	1,574.09	626.43	766.81	506.87	236.60	275.22	207.53
80	France	Number	16,505.38	27,144.63	7,102.25	52,615.77	58,527.07	47,593.97	54,699.45	66,093.15	44,054.53	65,340.11	75,061.54	57,543.16	23,376.99	29,105.15	18,380.12	6,346.86	7,352.59	5,582.59
80	France	Rate	421.16	692.64	181.22	1,342.57	1,493.41	1,214.43	1,395.74	1,686.47	1,124.12	1,667.25	1,915.31	1,468.30	596.50	742.66	469.00	161.95	187.61	142.45
81	Germany	Number	18,779.23	29,624.32	8,735.04	48,838.21	53,807.53	44,802.77	77,207.83	89,565.09	65,199.46	75,767.95	106,231.31	58,317.32	21,281.26	26,299.23	16,771.38	3,876.86	4,804.25	3,176.20
81	Germany	Rate	547.57	863.79	254.70	1,424.03	1,568.93	1,306.36	2,251.23	2,611.55	1,901.09	2,209.25	3,097.50	1,700.42	620.52	766.84	489.02	113.04	140.08	92.61
82	Greece	Number	1,415.84	2,151.54	769.61	7,376.90	8,301.74	6,506.72	6,254.40	8,786.75	4,381.95	7,715.39	9,185.40	6,556.29	2,954.70	3,659.51	2,354.05	756.18	1,088.89	513.75
82	Greece	Rate	299.62	455.30	162.86	1,561.09	1,756.80	1,376.94	1,323.54	1,859.43	927.30	1,632.72	1,943.80	1,387.43	625.27	774.42	498.16	160.02	230.43	108.72
83	Iceland	Number	69.48	103.35	38.57	368.67	413.42	330.41	266.11	377.09	184.73	363.90	424.64	312.05	127.78	160.44	100.40	36.19	43.88	30.18
83	Iceland	Rate	323.81	481.66	179.77	1,718.24	1,926.79	1,539.89	1,240.22	1,757.46	860.93	1,695.98	1,979.09	1,454.33	595.53	747.76	467.91	168.69	204.52	140.66
84	Ireland	Number	917.84	1,422.63	463.81	5,259.53	5,732.49	4,837.35	4,250.77	5,967.70	2,940.52	7,222.25	10,224.04	5,495.32	2,220.44	2,736.89	1,770.09	617.26	736.37	508.49
84	Ireland	Rate	272.86	422.93	137.89	1,563.60	1,704.20	1,438.09	1,263.70	1,774.13	874.18	2,147.09	3,039.49	1,633.69	660.11	813.65	526.23	183.50	218.91	151.17
85	Israel	Number	2,372.73	3,512.70	1,417.71	14,599.95	16,670.41	12,859.75	10,940.05	15,388.33	7,677.89	29,927.84	34,596.72	26,096.98	5,195.81	6,366.60	4,106.29	1,493.03	1,787.26	1,272.29
85	Israel	Rate	283.23	419.30	169.23	1,742.75	1,989.89	1,535.03	1,305.88	1,836.86	916.49	3,572.39	4,129.70	3,115.11	620.21	759.96	490.16	178.22	213.34	151.87
86	Italy	Number	7,012.16	10,598.74	3,655.02	45,543.28	50,474.34	41,363.10	33,356.75	47,364.93	23,192.86	58,328.22	67,660.52	50,999.40	15,882.42	19,455.34	12,439.78	4,753.07	5,533.34	4,131.64
86	Italy	Rate	275.96	417.11	143.84	1,792.35	1,986.41	1,627.84	1,312.75	1,864.04	912.75	2,295.49	2,662.76	2,007.07	625.05	765.66	489.56	187.06	217.76	162.60
87	Luxembourg	Number	87.05	131.79	47.07	446.88	497.14	403.96	362.83	513.79	251.70	716.83	964.40	578.04	186.99	230.18	149.58	47.08	67.80	31.99
87	Luxembourg	Rate	288.15	436.26	155.81	1,479.32	1,645.68	1,337.24	1,201.07	1,700.81	833.19	2,372.93	3,192.48	1,913.51	618.99	761.96	495.16	155.84	224.44	105.90
88	Malta	Number	59.39	88.69	33.94	328.32	365.48	299.87	244.67	344.50	171.68	418.36	570.20	323.70	119.35	147.67	94.59	30.30	43.63	20.58
88	Malta	Rate	311.31	464.92	177.91	1,721.08	1,915.87	1,571.94	1,282.61	1,805.89	899.95	2,193.10	2,989.06	1,696.85	625.66	774.10	495.86	158.82	228.70	107.91
89	Netherlands	Number	2,952.08	4,616.38	1,522.15	11,685.00	13,108.51	10,617.81	10,747.54	15,136.32	7,483.97	14,770.49	17,156.42	13,004.99	5,403.21	6,623.34	4,255.10	1,448.29	1,681.00	1,251.69
89	Netherlands	Rate	339.38	530.72	174.99	1,343.35	1,507.00	1,220.66	1,235.58	1,740.13	860.39	1,698.07	1,972.37	1,495.10	621.17	761.44	489.18	166.50	193.25	143.90
90	Norway	Number	919.19	1,350.00	501.67	4,533.56	4,919.82	4,176.50	2,701.76	3,317.45	2,197.09	5,120.66	6,004.44	4,348.44	1,784.81	2,184.56	1,431.48	409.31	476.95	353.97
90	Norway	Rate	309.33	454.31	168.82	1,525.65	1,655.63	1,405.49	909.21	1,116.40	739.37	1,723.22	2,020.63	1,463.35	600.63	735.15	481.73	137.74	160.50	119.12
91	Portugal	Number	946.06	1,517.35	455.65	5,017.13	5,963.78	4,352.28	5,580.38	7,850.85	3,868.37	9,754.89	13,207.39	7,556.66	2,638.16	3,240.32	2,121.53	677.10	975.04	460.04
91	Portugal	Rate	223.29	358.12	107.54	1,184.12	1,407.54	1,027.20	1,317.05	1,852.92	912.99	2,302.30	3,117.15	1,783.49	622.65	764.77	500.71	159.81	230.12	108.58
92	Spain	Number	5,894.19	9,231.31	3,180.00	32,629.44	38,869.87	27,160.15	28,363.70	39,977.17	19,940.65	49,641.64	56,305.74	44,237.67	13,441.41	16,573.75	10,519.41	5,241.47	6,022.10	4,681.49
92	Spain	Rate	272.17	426.27	146.84	1,506.70	1,794.86	1,254.15	1,309.72	1,845.99	920.78	2,292.26	2,599.98	2,042.72	620.67	765.31	485.75	242.03	278.08	216.17
93	Sweden	Number	1,429.14	2,089.21	920.29	9,762.80	10,850.84	8,797.31	5,937.90	7,278.26	4,880.78	7,241.55	8,643.62	6,315.73	4,732.77	5,669.72	3,881.67	852.30	970.31	752.30
93	Sweden	Rate	249.88	365.29	160.91	1,706.97	1,897.21	1,538.16	1,038.21	1,272.56	853.38	1,266.14	1,511.29	1,104.27	827.50	991.32	678.69	149.02	169.65	131.54
94	Switzerland	Number	1,492.25	2,100.13	950.81	6,091.79	6,875.80	5,477.16	5,057.44	7,127.92	3,510.94	8,125.89	11,379.28	6,118.49	2,598.76	3,220.93	2,058.10	622.09	743.80	521.53
94	Switzerland	Rate	358.08	503.95	228.16	1,461.81	1,649.94	1,314.32	1,213.60	1,710.44	842.50	1,949.91	2,730.61	1,468.21	623.61	772.90	493.87	149.28	178.48	125.15
95	United Kingdom	Number	13,284.67	16,796.70	10,530.10	55,815.22	62,198.26	50,153.20	43,189.94	51,807.13	35,776.03	85,538.35	101,040.38	73,966.90	24,817.11	29,884.19	20,253.96	6,561.45	7,545.54	5,758.13
95	United Kingdom	Rate	340.22	430.17	269.68	1,429.43	1,592.90	1,284.43	1,106.10	1,326.79	916.23	2,190.64	2,587.65	1,894.30	635.57	765.34	518.71	168.04	193.24	147.47
97	Argentina	Number	10,645.15	16,775.28	5,548.75	36,134.29	42,253.05	30,875.17	43,608.88	52,778.63	35,635.91	107,841.20	121,517.71	96,086.66	24,918.18	30,448.04	19,783.17	8,345.86	9,744.25	7,239.01
97	Argentina	Rate	297.21	468.37	154.92	1,008.88	1,179.71	862.04	1,217.57	1,473.59	994.96	3,010.94	3,392.80	2,682.76	695.72	850.11	552.35	233.02	272.06	202.11
98	Chile	Number	4,486.79	7,469.85	1,831.37	11,650.85	13,569.33	9,901.81	14,529.15	17,733.49	11,801.23	38,131.72	44,140.48	33,354.46	8,397.38	10,218.44	6,662.27	2,804.10	3,256.12	2,459.68
98	Chile	Rate	372.63	620.37	152.09	967.60	1,126.93	822.34	1,206.64	1,472.76	980.09	3,166.83	3,665.85	2,770.08	697.40	848.64	553.30	232.88	270.42	204.28
99	Uruguay	Number	851.87	1,356.91	395.17	2,706.28	3,147.41	2,312.65	2,908.33	3,548.91	2,376.55	7,772.45	8,862.92	6,879.82	1,653.09	2,013.35	1,317.06	552.81	645.42	479.55
99	Uruguay	Rate	358.55	571.13	166.33	1,139.07	1,324.75	973.40	1,224.12	1,493.74	1,000.29	3,271.43	3,730.41	2,895.72	695.79	847.42	554.35	232.68	271.66	201.84
101	Canada	Number	4,735.55	7,039.64	2,617.62	24,436.48	27,763.25	21,376.28	24,408.33	29,466.57	19,844.67	26,367.76	30,252.00	23,199.33	14,902.86	17,941.98	12,050.92	4,372.09	5,130.77	3,793.48
101	Canada	Rate	244.14	362.92	134.95	1,259.80	1,431.30	1,102.03	1,258.34	1,519.12	1,023.07	1,359.36	1,559.61	1,196.02	768.30	924.98	621.27	225.40	264.51	195.57
102	United States	Number	66,883.85	81,923.18	53,649.84	285,439.93	313,361.91	260,307.13	248,735.51	304,701.23	203,200.08	285,170.05	328,043.70	252,439.33	136,667.37	164,543.41	111,487.00	42,314.51	47,540.29	37,812.68
102	United States	Rate	340.15	416.63	272.84	1,451.64	1,593.64	1,323.82	1,264.97	1,549.59	1,033.40	1,450.26	1,668.30	1,283.81	695.04	836.80	566.98	215.20	241.77	192.30
105	Antigua and Barbuda	Number	34.41	51.70	20.19	66.13	88.78	53.79	116.12	135.85	97.87	193.19	216.41	174.16	44.23	54.25	35.53	17.60	20.33	15.41
105	Antigua and Barbuda	Rate	563.99	847.35	330.84	1,083.88	1,455.06	881.68	1,903.23	2,226.52	1,604.16	3,166.32	3,546.90	2,854.55	724.85	889.17	582.31	288.39	333.15	252.64
106	The Bahamas	Number	209.11	331.20	123.33	389.75	521.87	314.77	624.68	728.47	526.48	1,065.75	1,212.94	948.48	241.37	296.11	194.28	95.33	110.13	83.46
106	The Bahamas	Rate	633.60	1,003.54	373.71	1,180.97	1,581.29	953.76	1,892.80	2,207.28	1,595.24	3,229.26	3,675.26	2,873.93	731.36	897.21	588.68	288.85	333.70	252.89
107	Barbados	Number	120.02	198.98	73.06	200.93	283.85	155.88	281.27	329.14	235.71	438.65	514.28	389.34	107.46	131.34	85.12	43.02	49.70	37.68

Table S2. Global and country-specific prevalence of developmental disabilities in 2016 (with 95% uncertainty interval)																				
			**Epilepsy**			**Intellectual disability**			**Hearing loss**			**Vision loss**			**Autism spectrum disorder**			**Attention-deficit/hyperactivity disorder**		
**Loc-ID**	**Location**	**Metric**	**Value**	**Upper limit**	**Lower limit**	**Value**	**Upper limit**	**Lower limit**	**Value**	**Upper limit**	**Lower limit**	**Value**	**Upper limit**	**Lower limit**	**Value**	**Upper limit**	**Lower limit**	**Value**	**Upper limit**	**Lower limit**
107	Barbados	Rate	811.57	1,345.53	494.01	1,358.72	1,919.45	1,054.09	1,901.96	2,225.68	1,593.93	2,966.19	3,477.62	2,632.77	726.69	888.14	575.57	290.91	336.06	254.80
108	Belize	Number	335.07	570.05	199.04	692.72	911.02	555.69	977.26	1,130.75	827.54	1,860.51	2,097.25	1,672.87	343.39	421.28	274.87	135.76	156.83	118.92
108	Belize	Rate	705.28	1,199.90	418.97	1,458.11	1,917.61	1,169.66	2,057.03	2,380.12	1,741.88	3,916.19	4,414.49	3,521.23	722.80	886.74	578.57	285.76	330.11	250.31
109	Cuba	Number	2,940.15	4,468.57	1,626.82	6,355.28	7,653.43	5,300.78	11,614.24	13,562.92	9,726.84	20,793.44	23,572.42	18,562.51	4,404.33	5,424.16	3,533.26	1,768.04	2,042.60	1,547.96
109	Cuba	Rate	490.42	745.36	271.36	1,060.06	1,276.60	884.17	1,937.26	2,262.31	1,622.44	3,468.36	3,931.90	3,096.24	734.65	904.75	589.35	294.91	340.71	258.20
110	Dominica	Number	46.52	86.60	24.89	84.62	126.40	63.33	103.90	121.16	87.11	193.22	228.92	167.42	38.77	47.24	30.69	15.39	17.78	13.48
110	Dominica	Rate	874.57	1,628.32	468.00	1,590.95	2,376.50	1,190.72	1,953.43	2,277.99	1,637.72	3,632.90	4,304.11	3,147.82	728.92	888.22	577.01	289.39	334.32	253.39
111	Dominican Republic	Number	9,480.27	17,350.08	5,274.12	16,625.66	24,451.46	12,431.22	18,763.80	21,767.52	15,825.61	40,269.70	47,910.37	35,008.06	6,842.71	8,357.22	5,452.50	2,743.05	3,168.87	2,402.03
111	Dominican Republic	Rate	1,007.26	1,843.41	560.37	1,766.45	2,597.92	1,320.79	1,993.62	2,312.76	1,681.44	4,278.58	5,090.39	3,719.54	727.03	887.94	579.32	291.44	336.69	255.21
112	Grenada	Number	64.63	102.70	38.01	125.94	170.06	100.56	173.19	201.22	145.92	314.34	358.56	280.92	63.43	77.41	50.84	25.33	29.26	22.18
112	Grenada	Rate	742.95	1,180.61	436.89	1,447.72	1,954.93	1,156.01	1,990.90	2,313.15	1,677.44	3,613.43	4,121.72	3,229.31	729.20	889.83	584.42	291.15	336.35	254.95
113	Guyana	Number	572.77	1,001.43	340.98	1,147.83	1,580.10	911.02	1,335.83	1,545.42	1,128.21	2,618.56	3,035.00	2,331.69	473.36	582.08	380.42	188.03	217.21	164.67
113	Guyana	Rate	876.60	1,532.63	521.84	1,756.68	2,418.25	1,394.26	2,044.40	2,365.18	1,726.66	4,007.54	4,644.87	3,568.51	724.44	890.83	582.21	287.77	332.43	252.01
114	Haiti	Number	8,480.22	15,601.05	4,475.33	31,581.03	39,448.83	26,288.99	35,046.94	40,228.65	30,034.07	75,888.32	84,378.49	68,434.46	10,945.45	13,446.68	8,738.94	4,347.49	5,022.19	3,807.59
114	Haiti	Rate	558.14	1,026.81	294.55	2,078.56	2,596.39	1,730.25	2,306.67	2,647.71	1,976.74	4,994.71	5,553.51	4,504.12	720.39	885.02	575.17	286.14	330.54	250.60
115	Jamaica	Number	2,217.21	3,660.18	1,294.72	4,073.12	5,443.08	3,268.05	5,499.99	6,397.49	4,634.35	10,151.38	11,748.51	9,040.39	2,016.53	2,513.70	1,632.80	799.21	923.28	699.85
115	Jamaica	Rate	801.40	1,322.95	467.97	1,472.21	1,967.37	1,181.22	1,987.94	2,312.34	1,675.06	3,669.16	4,246.44	3,267.60	728.86	908.56	590.17	288.87	333.71	252.96
116	Saint Lucia	Number	74.44	124.83	44.95	138.30	194.89	109.55	191.10	221.63	160.54	347.79	397.33	309.36	69.89	86.71	55.57	27.98	32.33	24.51
116	Saint Lucia	Rate	772.75	1,295.93	466.68	1,435.77	2,023.15	1,137.26	1,983.85	2,300.73	1,666.57	3,610.46	4,124.78	3,211.52	725.52	900.15	576.93	290.50	335.59	254.47
117	Saint Vincent and the Grenadines	Number	71.76	121.40	43.30	134.55	188.66	105.97	185.46	215.46	156.94	351.58	401.96	313.85	66.27	82.42	52.82	26.55	30.66	23.25
117	Saint Vincent and the Grenadines	Rate	784.90	1,327.88	473.64	1,471.76	2,063.57	1,159.07	2,028.56	2,356.74	1,716.59	3,845.66	4,396.65	3,432.95	724.85	901.48	577.80	290.36	335.42	254.35
118	Suriname	Number	420.84	763.63	217.71	742.38	1,096.19	556.13	926.64	1,072.22	781.19	1,788.30	2,121.73	1,550.05	339.09	416.45	272.01	135.78	156.87	118.86
118	Suriname	Rate	905.85	1,643.69	468.61	1,597.96	2,359.51	1,197.05	1,994.56	2,307.93	1,681.49	3,849.27	4,566.97	3,336.44	729.89	896.40	585.49	292.26	337.67	255.84
119	Trinidad and Tobago	Number	453.76	717.51	270.78	781.98	1,042.46	630.74	1,330.08	1,550.52	1,114.95	2,280.33	2,563.28	2,035.63	501.91	621.92	401.31	202.88	234.36	177.68
119	Trinidad and Tobago	Rate	658.70	1,041.56	393.08	1,135.15	1,513.27	915.61	1,930.78	2,250.79	1,618.49	3,310.21	3,720.94	2,954.98	728.59	902.81	582.56	294.50	340.20	257.93
121	Bolivia	Number	6,319.07	10,761.14	3,441.33	17,207.62	22,051.08	13,884.57	29,315.09	34,261.23	24,855.88	71,492.65	80,583.93	64,288.26	9,777.33	12,147.66	7,937.49	2,232.24	2,619.86	1,929.33
121	Bolivia	Rate	461.72	786.29	251.45	1,257.32	1,611.22	1,014.51	2,141.98	2,503.38	1,816.16	5,223.79	5,888.07	4,697.38	714.40	887.60	579.97	163.10	191.43	140.97
122	Ecuador	Number	8,810.88	13,834.17	4,271.56	17,730.11	20,651.38	14,898.35	36,626.12	42,955.53	30,790.57	82,934.36	93,289.97	74,689.94	12,844.72	15,686.37	10,302.04	2,929.10	3,437.80	2,531.61
122	Ecuador	Rate	492.64	773.51	238.84	991.34	1,154.68	833.01	2,047.87	2,401.76	1,721.59	4,637.09	5,216.10	4,176.12	718.18	877.07	576.02	163.77	192.22	141.55
123	Peru	Number	23,496.56	37,658.71	12,464.55	42,890.92	56,334.83	34,491.97	73,615.99	85,954.60	62,137.19	173,766.89	196,746.12	155,616.59	25,233.80	31,205.68	20,186.38	5,717.73	6,711.01	4,941.73
123	Peru	Rate	670.25	1,074.23	355.56	1,223.48	1,606.98	983.90	2,099.93	2,451.89	1,772.49	4,956.77	5,612.27	4,439.03	719.80	890.16	575.83	163.10	191.43	140.96
125	Colombia	Number	18,265.81	30,878.38	7,607.66	34,454.47	40,392.96	29,156.17	71,697.23	83,374.66	60,450.06	157,262.27	177,875.08	141,763.64	26,855.84	33,387.16	21,520.08	4,698.20	5,420.58	4,124.12
125	Colombia	Rate	511.17	864.14	212.90	964.21	1,130.40	815.94	2,006.46	2,333.25	1,691.70	4,401.01	4,977.86	3,967.28	751.57	934.35	602.24	131.48	151.70	115.41
126	Costa Rica	Number	1,446.13	2,460.29	601.23	2,607.99	3,051.18	2,202.48	6,040.16	7,040.49	5,069.58	11,791.58	13,341.56	10,400.69	2,291.90	2,844.30	1,833.89	394.95	517.44	290.64
126	Costa Rica	Rate	475.39	808.77	197.64	857.33	1,003.02	724.02	1,985.58	2,314.42	1,666.53	3,876.25	4,385.78	3,419.02	753.42	935.01	602.86	129.83	170.10	95.54
127	El Salvador	Number	2,533.38	4,090.62	1,034.99	5,839.10	6,806.19	5,011.48	11,034.29	12,836.52	9,317.89	23,575.53	26,901.29	20,865.50	4,013.80	4,982.50	3,213.83	691.48	905.94	508.85
127	El Salvador	Rate	474.48	766.14	193.85	1,093.62	1,274.75	938.61	2,066.64	2,404.19	1,745.17	4,415.52	5,038.41	3,907.95	751.75	933.18	601.93	129.51	169.68	95.30
128	Guatemala	Number	7,758.90	12,710.78	3,659.84	22,247.97	25,868.70	18,980.62	42,334.14	48,791.93	36,028.81	81,355.77	91,977.15	72,358.99	14,695.95	17,983.59	11,805.80	2,512.67	3,291.39	1,849.51
128	Guatemala	Rate	393.51	644.66	185.62	1,128.36	1,311.99	962.65	2,147.07	2,474.59	1,827.28	4,126.14	4,664.83	3,669.85	745.34	912.08	598.76	127.44	166.93	93.80
129	Honduras	Number	4,276.57	6,925.64	1,903.04	10,711.21	12,511.33	9,085.44	20,295.37	23,391.90	17,336.01	43,437.46	48,796.08	38,747.62	7,091.44	8,774.33	5,702.87	1,210.06	1,585.25	890.55
129	Honduras	Rate	451.54	731.23	200.93	1,130.93	1,320.99	959.27	2,142.86	2,469.80	1,830.40	4,586.28	5,152.06	4,091.11	748.74	926.42	602.13	127.76	167.38	94.03
130	Mexico	Number	62,549.97	80,264.36	47,659.55	115,171.22	133,287.92	98,935.39	236,930.95	274,493.43	198,822.13	448,525.17	504,939.74	402,641.39	88,383.38	106,466.69	72,857.02	11,186.17	12,668.11	9,937.31
130	Mexico	Rate	535.92	687.70	408.34	986.78	1,142.00	847.67	2,030.01	2,351.84	1,703.50	3,842.93	4,326.29	3,449.81	757.26	912.20	624.23	95.84	108.54	85.14
131	Nicaragua	Number	2,787.38	4,472.53	1,207.32	6,870.78	7,969.37	5,813.54	12,997.86	15,021.19	11,048.58	27,693.48	31,377.20	24,570.87	4,559.37	5,681.54	3,643.27	788.52	1,032.97	580.35
131	Nicaragua	Rate	457.59	734.23	198.20	1,127.94	1,308.29	954.38	2,133.79	2,465.95	1,813.79	4,546.30	5,151.03	4,033.67	748.49	932.71	598.10	129.45	169.58	95.27
132	Panama	Number	1,387.83	2,323.20	612.97	3,126.48	3,635.48	2,663.94	6,774.63	7,883.88	5,678.14	13,610.18	15,708.41	11,930.23	2,609.05	3,209.87	2,101.46	448.42	587.47	330.00
132	Panama	Rate	400.27	670.03	176.79	901.71	1,048.51	768.31	1,953.88	2,273.80	1,637.64	3,925.32	4,530.48	3,440.81	752.48	925.76	606.09	129.33	169.43	95.18
133	Venezuela	Number	14,155.39	23,324.77	6,261.63	27,011.99	31,470.04	22,877.86	56,674.25	66,117.59	47,750.32	107,253.59	122,154.94	94,960.71	21,693.83	26,800.63	17,224.75	4,755.18	5,634.17	4,059.92
133	Venezuela	Rate	490.44	808.13	216.95	935.88	1,090.34	792.64	1,963.58	2,290.76	1,654.40	3,715.99	4,232.28	3,290.08	751.62	928.56	596.78	164.75	195.21	140.66
135	Brazil	Number	68,266.81	88,709.86	52,459.56	178,469.77	205,054.99	155,165.48	303,644.71	352,311.54	259,332.05	743,924.88	831,062.49	671,449.98	107,197.86	131,072.39	88,222.26	21,008.61	23,349.84	18,960.29
135	Brazil	Rate	441.05	573.13	338.93	1,153.05	1,324.81	1,002.48	1,961.77	2,276.19	1,675.48	4,806.30	5,369.28	4,338.06	692.58	846.82	569.98	135.73	150.86	122.50
136	Paraguay	Number	2,724.43	4,259.80	1,471.98	7,277.05	8,545.82	6,167.25	12,407.54	14,502.91	10,573.97	30,764.45	34,839.00	27,406.12	4,362.85	5,381.86	3,442.42	829.99	971.05	717.79
136	Paraguay	Rate	431.93	675.35	233.37	1,153.71	1,354.86	977.76	1,967.10	2,299.30	1,676.40	4,877.42	5,523.40	4,344.98	691.69	853.24	545.76	131.59	153.95	113.80
139	Algeria	Number	33,772.54	58,520.28	18,916.15	103,333.78	130,705.10	83,870.96	86,783.85	103,120.84	71,350.91	217,496.98	269,244.72	179,293.90	35,705.94	43,117.37	28,800.47	6,895.18	8,647.69	5,480.99
139	Algeria	Rate	742.32	1,286.27	415.78	2,271.27	2,872.89	1,843.48	1,907.50	2,266.59	1,568.29	4,780.57	5,917.98	3,940.87	784.81	947.72	633.03	151.56	190.08	120.47
140	Bahrain	Number	632.42	969.36	317.89	1,660.22	2,005.25	1,368.02	1,808.27	2,153.01	1,485.70	4,441.35	5,572.00	3,631.62	785.04	961.19	631.15	150.79	189.13	119.86
140	Bahrain	Rate	634.33	972.29	318.85	1,665.23	2,011.31	1,372.15	1,813.74	2,159.51	1,490.19	4,454.77	5,588.84	3,642.60	787.41	964.09	633.06	151.25	189.70	120.23
141	Egypt	Number	88,722.45	160,637.42	46,507.19	297,971.95	374,732.11	238,212.84	210,539.60	251,076.50	175,225.76	612,693.59	718,223.00	533,150.80	85,429.91	104,321.00	68,348.28	22,190.25	25,244.70	19,253.65
141	Egypt	Rate	813.48	1,472.86	426.42	2,732.06	3,435.86	2,184.14	1,930.40	2,302.08	1,606.62	5,617.69	6,585.28	4,888.38	783.29	956.50	626.67	203.46	231.46	176.53

Table S2. Global and country-specific prevalence of developmental disabilities in 2016 (with 95% uncertainty interval)

			Epilepsy			Intellectual disability			Hearing loss			Vision loss			Autism spectrum disorder			Attention-deficit/hyperactivity disorder		
Loc-ID	Location	Metric	Value	Upper limit	Lower limit	Value	Upper limit	Lower limit	Value	Upper limit	Lower limit	Value	Upper limit	Lower limit	Value	Upper limit	Lower limit	Value	Upper limit	Lower limit
142	Iran	Number	58,464.69	92,330.87	28,445.77	168,770.83	207,027.13	139,908.75	146,675.37	174,346.33	119,392.60	266,341.61	310,309.75	233,830.55	64,647.12	78,071.78	52,237.70	18,329.34	20,528.97	16,514.34
142	Iran	Rate	724.95	1,144.88	352.72	2,092.71	2,567.08	1,734.83	1,818.74	2,161.85	1,480.44	3,302.56	3,847.76	2,899.44	801.61	968.07	647.73	227.28	254.55	204.77
143	Iraq	Number	76,074.02	167,810.37	42,286.20	201,119.30	285,334.45	159,385.35	162,107.81	191,784.71	133,243.55	466,311.83	595,399.52	390,761.81	60,170.00	74,429.26	48,649.27	6,538.06	7,565.32	5,717.16
143	Iraq	Rate	987.33	2,177.94	548.81	2,610.24	3,703.24	2,068.60	2,103.93	2,489.09	1,729.31	6,052.07	7,727.44	5,071.53	780.92	965.99	631.40	84.85	98.19	74.20
144	Jordan	Number	5,851.64	8,260.60	3,160.96	20,638.59	24,457.50	17,160.69	18,105.86	21,504.47	14,874.39	43,275.99	54,127.69	35,674.53	7,559.03	9,320.83	6,086.60	1,434.09	1,798.55	1,139.93
144	Jordan	Rate	609.38	860.25	329.18	2,149.27	2,546.97	1,787.09	1,885.52	2,239.44	1,549.00	4,506.70	5,636.78	3,715.09	787.19	970.66	633.85	149.34	187.30	118.71
145	Kuwait	Number	1,563.51	2,385.10	797.44	4,473.12	5,307.07	3,767.20	4,840.19	5,768.96	3,977.39	11,314.01	14,349.44	9,256.28	2,225.13	2,768.95	1,785.10	426.00	534.28	338.62
145	Kuwait	Rate	554.75	846.26	282.94	1,587.12	1,883.01	1,336.65	1,717.36	2,046.89	1,411.22	4,014.34	5,091.35	3,284.24	789.50	982.46	633.37	151.15	189.57	120.15
146	Lebanon	Number	2,099.59	3,502.11	1,049.67	6,832.20	8,487.07	5,651.44	5,660.69	6,758.64	4,631.23	11,573.37	13,460.68	10,079.99	2,523.30	3,091.82	2,062.37	581.56	668.17	504.66
146	Lebanon	Rate	654.46	1,091.64	327.19	2,129.66	2,645.50	1,761.61	1,764.49	2,106.73	1,443.60	3,607.53	4,195.82	3,142.03	786.54	963.75	642.86	181.28	208.27	157.31
147	Libya	Number	2,866.44	5,097.83	1,603.03	9,862.29	12,237.63	8,055.67	8,395.09	10,022.38	6,869.24	18,480.04	21,394.51	15,945.90	3,667.12	4,456.51	2,942.49	720.46	903.53	572.66
147	Libya	Rate	619.13	1,101.09	346.24	2,130.17	2,643.23	1,739.96	1,813.27	2,164.75	1,483.70	3,991.54	4,621.04	3,444.19	792.07	962.57	635.55	155.61	195.15	123.69
148	Morocco	Number	16,947.88	30,016.86	9,014.73	58,823.88	73,960.75	48,024.08	44,933.30	53,719.63	37,009.72	105,206.86	120,246.27	94,511.80	18,011.31	22,154.52	14,565.79	3,473.17	4,355.79	2,760.72
148	Morocco	Rate	739.64	1,309.99	393.42	2,567.18	3,227.78	2,095.86	1,960.97	2,344.42	1,615.17	4,591.42	5,247.77	4,124.67	786.05	966.86	635.68	151.58	190.09	120.48
149	Palestine	Number	9,318.47	18,844.69	5,193.37	30,302.54	39,385.08	24,270.03	23,451.10	27,685.23	19,534.84	59,224.54	69,776.73	50,959.51	8,496.95	10,413.95	6,883.67	1,604.11	2,011.82	1,275.11
149	Palestine	Rate	858.51	1,736.17	478.47	2,791.78	3,628.56	2,236.01	2,160.56	2,550.65	1,799.75	5,456.38	6,428.56	4,694.92	782.83	959.44	634.19	147.79	185.35	117.48
150	Oman	Number	2,165.43	3,267.36	948.81	7,176.84	8,589.60	5,977.52	6,041.62	7,387.47	4,864.21	20,080.52	24,861.90	16,561.28	3,473.60	4,224.67	2,783.47	660.83	828.79	525.29
150	Oman	Rate	488.40	736.93	214.00	1,618.68	1,937.32	1,348.19	1,362.64	1,666.19	1,097.09	4,529.01	5,607.42	3,735.28	783.45	952.84	627.79	149.05	186.93	118.48
151	Qatar	Number	660.92	979.47	321.86	2,057.58	2,365.67	1,789.49	2,106.93	2,510.02	1,723.59	4,628.67	5,384.50	3,986.20	973.09	1,204.53	781.12	183.26	229.85	145.68
151	Qatar	Rate	533.83	791.12	259.97	1,661.92	1,910.76	1,445.38	1,701.78	2,027.36	1,392.15	3,738.60	4,349.10	3,219.68	785.97	972.91	630.92	148.02	185.65	117.66
152	Saudi Arabia	Number	19,534.15	31,784.35	12,760.68	47,852.30	61,540.59	39,545.56	45,732.68	54,059.40	38,612.01	109,884.26	127,589.18	95,375.91	19,758.79	24,123.19	16,087.96	3,780.60	4,371.64	3,299.76
152	Saudi Arabia	Rate	783.85	1,275.42	512.05	1,920.18	2,469.46	1,586.86	1,835.13	2,169.26	1,549.40	4,409.36	5,119.81	3,827.18	792.87	968.00	645.57	151.71	175.42	132.41
153	Syria	Number	11,420.27	18,429.74	5,710.07	44,502.52	54,367.70	36,493.69	35,079.62	41,790.86	29,021.60	73,343.35	95,471.76	59,875.28	14,012.82	17,297.81	11,260.86	2,760.80	3,462.36	2,194.45
153	Syria	Rate	646.31	1,043.00	323.15	2,518.54	3,076.84	2,065.30	1,985.27	2,365.08	1,642.43	4,150.74	5,403.06	3,388.54	793.03	978.94	637.29	156.24	195.95	124.19
154	Tunisia	Number	5,907.74	10,836.85	3,223.27	18,598.08	23,848.56	15,014.33	15,791.69	18,749.40	13,011.78	30,553.25	35,471.19	27,014.86	6,555.24	8,069.97	5,274.88	1,690.84	1,947.28	1,470.92
154	Tunisia	Rate	707.99	1,298.70	386.28	2,228.81	2,858.04	1,799.33	1,892.49	2,246.95	1,559.35	3,661.53	4,250.90	3,237.49	785.59	967.11	632.15	202.63	233.36	176.28
155	Turkey	Number	35,477.26	51,626.23	17,120.33	112,642.86	135,870.41	93,135.73	111,805.65	133,988.77	91,664.39	221,095.81	244,601.62	200,853.58	48,232.69	59,279.83	38,805.08	15,112.12	17,477.11	13,031.50
155	Turkey	Rate	576.06	838.28	277.99	1,829.05	2,206.20	1,512.30	1,815.45	2,175.65	1,488.41	3,590.06	3,971.73	3,261.37	783.18	962.56	630.10	245.38	283.79	211.60
156	United Arab Emirates	Number	5,006.62	7,645.43	2,568.06	13,315.33	16,113.34	11,048.93	14,472.11	17,257.85	11,854.30	40,827.38	50,979.52	34,676.10	6,420.06	7,857.70	5,227.47	714.36	848.53	590.83
156	United Arab Emirates	Rate	613.96	937.55	314.92	1,632.85	1,975.96	1,354.92	1,774.70	2,116.31	1,453.68	5,006.62	6,251.57	4,252.30	787.29	963.58	641.04	87.60	104.05	72.45
157	Yemen	Number	26,536.33	45,881.14	14,024.50	148,768.63	182,180.31	121,461.76	171,005.72	191,022.09	151,876.19	253,429.18	289,704.82	220,384.48	36,019.77	44,238.58	29,083.26	4,831.39	5,700.35	4,147.77
157	Yemen	Rate	573.54	991.65	303.12	3,215.42	3,937.56	2,625.22	3,696.04	4,128.67	3,282.58	5,477.50	6,261.55	4,763.29	778.52	956.15	628.59	104.42	123.20	89.65
160	Afghanistan	Number	29,095.10	56,222.45	14,464.94	191,733.89	234,165.69	155,312.59	119,602.31	142,701.11	99,551.89	325,517.75	372,577.25	284,658.69	38,541.73	47,321.88	31,197.48	7,238.64	9,078.19	5,753.80
160	Afghanistan	Rate	586.92	1,134.14	291.79	3,867.73	4,723.68	3,133.02	2,412.66	2,878.62	2,008.20	6,566.46	7,515.76	5,742.24	777.48	954.59	629.33	146.02	183.13	116.07
161	Bangladesh	Number	84,923.58	121,711.53	57,779.00	205,260.64	250,141.65	166,424.83	679,629.23	788,409.60	585,586.22	444,418.77	508,274.95	392,596.71	105,804.24	130,411.65	85,332.90	9,345.34	11,114.36	7,865.41
161	Bangladesh	Rate	592.65	849.38	403.22	1,432.44	1,745.65	1,161.42	4,742.90	5,502.04	4,086.60	3,101.44	3,547.07	2,739.80	738.37	910.10	595.51	65.22	77.56	54.89
162	Bhutan	Number	501.97	748.33	324.00	1,237.49	1,574.52	967.42	2,536.86	3,074.27	2,061.11	1,949.21	2,291.43	1,654.78	576.17	718.88	457.51	48.35	59.63	38.97
162	Bhutan	Rate	643.00	958.56	415.02	1,585.15	2,016.86	1,239.21	3,249.56	3,937.95	2,640.16	2,496.81	2,935.19	2,119.68	738.04	920.84	586.04	61.93	76.38	49.92
163	India	Number	823,482.41	1,058,323.00	657,410.91	3,190,464.53	3,872,888.30	2,523,762.73	3,533,324.00	4,026,636.56	3,096,058.16	5,097,650.84	5,690,437.75	4,575,076.44	850,811.93	1,037,775.28	696,988.94	66,937.19	75,229.70	60,271.03
163	India	Rate	734.33	943.75	586.24	2,845.06	3,453.60	2,250.53	3,150.80	3,590.70	2,760.87	4,545.77	5,074.38	4,079.77	758.70	925.42	621.53	59.69	67.09	53.75
164	Nepal	Number	27,619.11	38,097.14	18,349.00	81,673.71	103,169.58	63,239.86	121,927.10	151,151.45	98,177.76	112,737.05	130,501.28	98,297.62	29,531.01	36,572.65	23,636.85	2,449.56	3,021.10	1,974.55
164	Nepal	Rate	692.06	954.61	459.77	2,046.51	2,585.14	1,584.61	3,055.15	3,787.43	2,460.06	2,824.87	3,270.00	2,463.06	739.96	916.41	592.27	61.38	75.70	49.48
165	Pakistan	Number	149,687.85	213,459.68	96,105.95	419,407.14	522,918.13	320,831.82	744,025.05	904,474.19	603,178.22	660,340.86	766,426.85	574,718.38	172,207.12	211,460.36	137,285.35	14,329.02	17,673.21	11,551.77
165	Pakistan	Rate	648.81	925.22	416.56	1,817.88	2,266.53	1,390.61	3,224.90	3,920.35	2,614.41	2,862.18	3,322.00	2,491.06	746.41	916.55	595.05	62.11	76.60	50.07
168	Angola	Number	31,867.71	52,079.41	17,946.22	93,135.47	117,986.58	73,019.74	155,090.88	175,083.77	135,494.69	330,944.69	357,909.98	305,322.82	34,445.79	42,879.32	27,447.53	8,739.34	10,218.96	7,561.70
168	Angola	Rate	649.53	1,061.48	365.78	1,898.28	2,404.80	1,488.29	3,161.06	3,568.55	2,761.65	6,745.30	7,294.91	6,223.08	702.07	873.96	559.43	178.12	208.28	154.12
169	Central African Republic	Number	3,860.39	6,355.79	2,403.75	21,582.15	26,680.96	16,861.00	25,690.09	29,034.15	22,460.93	59,406.23	64,149.72	55,245.63	5,224.83	6,566.52	4,171.23	1,309.26	1,530.91	1,133.00
169	Central African Republic	Rate	520.24	856.52	323.94	2,908.47	3,595.60	2,272.23	3,462.07	3,912.72	3,026.90	8,005.74	8,644.99	7,445.05	704.11	884.92	562.13	176.44	206.31	152.69
170	Congo	Number	5,488.95	9,367.83	3,178.98	14,798.89	19,077.63	11,433.73	22,033.13	24,938.80	19,254.38	43,747.40	48,032.26	40,104.76	5,256.63	6,507.28	4,196.78	1,340.27	1,567.18	1,159.65
170	Congo	Rate	736.61	1,257.15	426.62	1,985.99	2,560.20	1,534.39	2,956.82	3,346.76	2,583.92	5,870.85	6,445.87	5,382.01	705.43	873.27	563.20	179.86	210.31	155.62
171	Democratic Republic of the Congo	Number	85,360.35	143,939.07	48,984.09	393,939.05	486,574.49	306,119.91	487,797.04	540,249.22	436,580.37	1,127,252.97	1,218,698.77	1,043,348.02	98,104.69	121,286.89	77,889.82	24,834.30	29,038.89	21,487.86
171	Democratic Republic of the Congo	Rate	607.24	1,023.95	348.46	2,802.40	3,461.39	2,177.67	3,470.09	3,843.22	3,105.74	8,019.04	8,669.57	7,422.16	697.90	862.81	554.09	176.67	206.58	152.86
172	Equatorial Guinea	Number	618.37	1,018.06	347.69	1,418.62	1,837.56	1,089.64	2,593.64	2,934.88	2,242.03	4,683.66	5,114.68	4,296.29	696.28	855.21	562.66	176.28	206.13	152.53
172	Equatorial Guinea	Rate	623.80	1,026.98	350.74	1,431.06	1,853.68	1,099.20	2,616.39	2,960.62	2,261.70	4,724.74	5,159.54	4,333.97	702.39	862.71	567.59	177.83	207.93	153.87
173	Gabon	Number	2,189.52	3,791.92	1,252.30	4,268.37	6,018.94	3,186.53	6,729.04	7,617.77	5,846.06	12,925.23	14,580.84	11,760.64	1,722.31	2,121.23	1,367.04	439.80	514.26	380.54
173	Gabon	Rate	894.25	1,548.71	511.47	1,743.30	2,458.28	1,301.45	2,748.30	3,111.28	2,387.67	5,278.97	5,955.16	4,803.32	703.43	866.36	558.33	179.62	210.03	155.42
175	Burundi	Number	14,066.45	22,284.15	7,696.08	57,514.71	72,428.98	43,852.15	60,689.27	71,838.24	50,260.34	82,650.92	100,309.84	67,177.80	15,026.46	18,855.62	11,987.54	3,802.87	4,446.70	3,290.54
175	Burundi	Rate	656.58	1,040.16	359.23	2,684.62	3,380.78	2,046.89	2,832.80	3,353.20	2,346.01	3,857.91	4,682.17	3,135.67	701.39	880.13	559.54	177.51	207.56	153.59
176	Comoros	Number	845.54	1,321.33	493.16	2,161.13	2,803.05	1,637.49	2,380.77	2,806.66	1,986.73	5,133.97	5,703.54	4,646.01	655.72	806.41	524.14	169.50	198.20	146.61

Table S2. Global and country-specific prevalence of developmental disabilities in 2016 (with 95% uncertainty interval)																				
			Epilepsy			Intellectual disability			Hearing loss			Vision loss			Autism spectrum disorder			Attention-deficit/hyperactivity disorder		
Loc-ID	Location	Metric	Value	Upper limit	Lower limit	Value	Upper limit	Lower limit	Value	Upper limit	Lower limit	Value	Upper limit	Lower limit	Value	Upper limit	Lower limit	Value	Upper limit	Lower limit
176	Comoros	Rate	911.34	1,424.14	531.54	2,329.29	3,021.16	1,764.91	2,566.03	3,025.05	2,141.32	5,533.46	6,147.35	5,007.53	706.75	869.16	564.93	182.69	213.62	158.02
177	Djibouti	Number	1,536.38	2,572.42	876.91	3,619.86	4,683.89	2,779.83	4,473.33	5,257.00	3,745.62	6,190.90	7,550.66	5,084.30	1,211.56	1,493.16	965.23	312.54	365.46	270.38
177	Djibouti	Rate	891.07	1,491.95	508.59	2,099.44	2,716.56	1,612.24	2,594.44	3,048.95	2,172.38	3,590.60	4,379.23	2,948.79	702.68	866.00	559.82	181.27	211.96	156.82
178	Eritrea	Number	7,272.80	11,409.51	4,485.33	20,631.33	25,992.08	15,790.05	20,967.20	24,784.06	17,336.93	32,331.69	38,358.02	27,141.75	5,628.59	6,983.05	4,467.07	1,451.16	1,696.89	1,255.22
178	Eritrea	Rate	914.42	1,434.54	563.95	2,594.01	3,268.03	1,985.31	2,636.24	3,116.14	2,179.80	4,065.12	4,822.82	3,412.58	707.69	877.99	561.65	182.46	213.35	157.82
179	Ethiopia	Number	122,741.77	190,003.46	76,695.74	375,169.19	471,454.04	288,782.67	417,160.13	496,182.64	346,862.64	522,753.39	589,375.81	469,732.67	107,894.90	134,669.27	85,829.23	27,318.74	31,944.27	23,634.64
179	Ethiopia	Rate	803.04	1,243.11	501.79	2,454.56	3,084.51	1,889.38	2,729.29	3,246.30	2,269.37	3,420.14	3,856.02	3,073.25	705.91	881.08	561.54	178.73	209.00	154.63
180	Kenya	Number	64,085.26	92,430.11	48,420.83	141,386.77	178,212.64	108,465.90	214,574.15	241,111.97	188,506.58	287,412.01	317,308.87	263,987.72	46,744.92	56,807.53	37,965.47	11,858.16	13,368.25	10,566.11
180	Kenya	Rate	974.90	1,406.10	736.60	2,150.85	2,711.07	1,650.04	3,264.22	3,667.92	2,867.66	4,372.26	4,827.07	4,015.92	711.11	864.19	577.55	180.39	203.37	160.74
181	Madagascar	Number	29,704.14	47,692.34	17,660.80	95,458.54	120,407.24	73,603.56	126,045.77	141,436.03	111,382.50	149,265.51	180,926.06	123,476.01	27,281.65	33,851.78	21,979.39	6,930.53	8,103.91	5,996.65
181	Madagascar	Rate	763.84	1,226.41	454.15	2,454.72	3,096.28	1,892.72	3,241.27	3,637.04	2,864.21	3,838.37	4,652.52	3,175.19	701.55	870.50	565.20	178.22	208.39	154.20
182	Malawi	Number	23,690.64	37,640.71	13,815.60	79,989.69	99,394.04	61,423.17	86,881.68	102,936.53	72,340.72	85,501.56	99,773.32	75,692.39	22,553.70	27,902.04	17,944.50	5,716.86	6,684.76	4,946.52
182	Malawi	Rate	740.02	1,175.78	431.56	2,498.63	3,104.76	1,918.67	2,713.91	3,215.42	2,259.70	2,670.80	3,116.61	2,364.40	704.51	871.57	560.53	178.58	208.81	154.51
183	Mauritius	Number	412.98	589.34	211.96	631.93	811.97	484.93	1,185.75	1,417.88	978.39	1,995.55	2,347.40	1,729.80	462.25	575.26	366.86	56.33	74.51	42.02
183	Mauritius	Rate	624.23	890.81	320.38	955.19	1,227.32	732.99	1,792.31	2,143.18	1,478.87	3,016.36	3,548.19	2,614.66	698.70	869.52	554.52	85.14	112.62	63.51
184	Mozambique	Number	40,406.86	66,500.55	22,185.44	120,991.66	152,823.25	92,653.13	137,773.64	162,066.19	114,338.65	217,309.95	260,551.07	179,208.82	34,821.97	43,087.35	28,220.41	8,894.00	10,399.80	7,695.60
184	Mozambique	Rate	813.63	1,339.05	446.72	2,436.27	3,077.23	1,865.65	2,774.19	3,263.35	2,302.31	4,375.73	5,246.43	3,608.53	701.17	867.60	568.24	179.09	209.41	154.96
185	Rwanda	Number	16,228.90	25,650.53	9,640.12	43,397.31	54,796.48	33,471.54	48,882.21	57,593.57	40,859.69	68,985.54	82,470.75	56,741.47	13,128.35	16,449.52	10,420.12	3,369.70	3,940.16	2,916.08
185	Rwanda	Rate	866.87	1,370.13	514.93	2,318.08	2,926.96	1,787.89	2,611.05	3,076.37	2,182.53	3,684.88	4,405.19	3,030.86	701.25	878.65	556.59	179.99	210.46	155.76
186	Seychelles	Number	50.30	72.24	28.51	75.87	98.86	58.78	151.06	180.34	124.55	256.76	315.06	204.00	58.83	73.38	47.03	7.22	9.56	5.39
186	Seychelles	Rate	602.25	865.04	341.34	908.45	1,183.74	703.90	1,808.82	2,159.36	1,491.37	3,074.52	3,772.51	2,442.77	704.49	878.61	563.11	86.51	114.45	64.49
187	Somalia	Number	9,010.21	14,176.23	5,141.11	38,163.20	47,939.05	29,102.49	37,416.71	44,392.40	31,247.49	59,425.37	72,075.65	48,863.21	9,269.09	11,509.30	7,405.67	2,349.55	2,747.34	2,032.96
187	Somalia	Rate	683.21	1,074.94	389.83	2,893.79	3,635.06	2,206.75	2,837.19	3,366.13	2,369.39	4,506.03	5,465.26	3,705.14	702.84	872.71	561.55	178.16	208.32	154.15
189	Tanzania	Number	77,064.19	117,808.09	44,189.21	192,472.43	244,196.70	147,930.80	201,307.23	234,152.59	169,540.87	333,840.67	398,670.07	273,862.61	63,325.26	78,734.29	50,024.23	16,159.11	18,895.01	13,981.08
189	Tanzania	Rate	855.99	1,308.56	490.83	2,137.90	2,712.43	1,643.15	2,236.03	2,600.86	1,883.18	3,708.15	4,428.25	3,041.94	703.39	874.55	555.65	179.49	209.88	155.30
190	Uganda	Number	66,875.61	105,243.89	37,823.68	169,086.77	210,080.56	131,455.74	156,395.86	183,349.07	130,054.33	276,477.23	332,990.38	227,874.42	52,930.90	65,275.59	42,329.82	13,420.09	15,692.14	11,612.19
190	Uganda	Rate	889.58	1,399.96	503.13	2,249.20	2,794.50	1,748.63	2,080.39	2,438.92	1,729.99	3,677.72	4,429.46	3,031.20	704.09	868.30	563.07	178.51	208.74	154.47
191	Zambia	Number	22,117.14	34,372.76	12,664.69	56,515.44	72,169.12	43,465.30	71,241.73	83,374.85	59,562.27	91,987.34	103,058.92	83,205.08	20,079.50	25,065.08	15,960.69	5,070.07	5,928.44	4,387.03
191	Zambia	Rate	778.16	1,209.36	445.59	1,988.41	2,539.17	1,529.26	2,506.54	2,933.42	2,095.61	3,236.44	3,625.98	2,927.45	706.47	881.88	561.55	178.38	208.58	154.35
193	Botswana	Number	1,644.48	2,264.49	1,009.09	3,904.76	4,861.90	2,997.37	6,285.52	7,378.97	5,194.52	10,207.61	11,339.97	9,184.02	1,917.13	2,371.14	1,531.77	479.77	561.00	415.09
193	Botswana	Rate	621.19	855.39	381.18	1,474.99	1,836.54	1,132.23	2,374.30	2,787.34	1,962.18	3,855.83	4,283.57	3,469.18	724.18	895.67	578.61	181.23	211.91	156.79
194	Lesotho	Number	1,369.31	2,050.66	834.72	5,520.40	6,789.08	4,304.60	6,658.93	7,845.75	5,548.64	12,256.24	13,480.78	11,081.68	1,857.08	2,325.66	1,495.08	465.08	543.82	402.46
194	Lesotho	Rate	530.28	794.14	323.25	2,137.83	2,629.14	1,667.00	2,578.73	3,038.34	2,148.76	4,746.34	5,220.56	4,291.49	719.17	900.63	578.99	180.11	210.60	155.85
195	Namibia	Number	2,068.36	3,050.00	1,213.68	5,561.66	6,868.54	4,339.42	8,178.32	9,614.65	6,819.40	14,286.76	15,689.31	13,124.10	2,384.25	3,002.30	1,891.38	597.83	699.05	517.26
195	Namibia	Rate	621.43	916.35	364.64	1,670.96	2,063.61	1,303.75	2,457.13	2,888.66	2,048.84	4,292.37	4,713.76	3,943.05	716.33	902.02	568.25	179.62	210.03	155.41
196	South Africa	Number	34,489.85	49,785.52	25,133.62	82,534.35	102,640.40	64,597.87	152,122.87	170,578.84	133,913.16	211,153.24	231,110.35	194,196.36	36,012.03	43,825.45	29,261.21	9,002.07	10,148.48	8,021.25
196	South Africa	Rate	688.26	993.49	501.55	1,647.00	2,048.22	1,289.07	3,035.66	3,403.95	2,672.28	4,213.63	4,611.88	3,875.25	718.63	874.55	583.92	179.64	202.52	160.07
197	Swaziland	Number	1,254.66	1,840.62	736.57	3,533.10	4,389.65	2,774.66	5,243.70	6,178.41	4,351.64	9,718.58	10,616.59	8,942.01	1,500.54	1,855.43	1,213.35	376.33	440.04	325.62
197	Swaziland	Rate	601.65	882.64	353.21	1,694.24	2,104.99	1,330.55	2,514.54	2,962.77	2,086.76	4,660.40	5,091.03	4,288.01	719.56	889.75	581.84	180.46	211.02	156.15
198	Zimbabwe	Number	17,933.48	29,525.18	10,399.18	61,514.42	77,652.92	47,504.16	52,476.52	60,774.35	44,421.08	114,399.75	127,470.59	104,055.18	18,104.04	22,582.03	14,450.97	4,538.32	5,306.63	3,927.33
198	Zimbabwe	Rate	708.07	1,165.75	410.59	2,428.79	3,066.00	1,875.62	2,071.95	2,399.57	1,753.89	4,516.88	5,032.96	4,108.45	714.81	891.61	570.57	179.19	209.52	155.06
200	Benin	Number	15,499.59	24,779.99	8,360.80	48,076.33	59,302.47	37,812.85	47,769.41	55,131.06	40,888.30	84,728.76	100,345.61	70,073.61	13,308.83	16,633.04	10,739.14	3,524.63	4,101.18	3,073.57
200	Benin	Rate	811.18	1,296.88	437.57	2,516.11	3,103.64	1,978.96	2,500.05	2,885.32	2,139.92	4,434.34	5,251.66	3,667.35	696.53	870.50	562.04	184.46	214.64	160.86
201	Burkina Faso	Number	19,892.08	32,141.19	11,810.66	86,744.99	106,468.76	69,223.80	83,387.22	95,539.46	71,444.43	93,692.92	106,421.58	83,902.13	22,280.29	27,856.98	17,811.07	5,902.69	6,868.35	5,147.09
201	Burkina Faso	Rate	622.65	1,006.06	369.69	2,715.23	3,332.61	2,166.79	2,610.13	2,990.51	2,236.30	2,932.71	3,331.13	2,626.24	697.40	871.96	557.51	184.76	214.99	161.11
202	Cameroon	Number	29,726.60	49,666.03	15,858.38	89,584.67	110,131.23	70,990.61	92,678.16	106,440.77	78,313.37	142,864.49	169,381.46	118,785.03	27,009.00	33,983.33	21,371.69	7,161.06	8,332.01	6,245.57
202	Cameroon	Rate	766.70	1,280.97	409.01	2,310.53	2,840.46	1,830.96	2,390.32	2,745.28	2,019.83	3,684.71	4,368.62	3,063.66	696.61	876.49	551.21	184.70	214.90	161.08
203	Cape Verde	Number	705.93	1,042.02	419.75	1,405.93	1,760.83	1,088.11	1,710.22	1,979.95	1,441.21	2,250.08	2,648.38	1,871.28	525.50	652.23	419.83	141.66	164.83	123.54
203	Cape Verde	Rate	940.22	1,387.86	559.06	1,872.55	2,345.23	1,449.25	2,277.83	2,637.08	1,919.54	2,996.87	3,527.36	2,492.34	699.91	868.70	559.16	188.67	219.53	164.54
204	Chad	Number	15,316.70	25,851.12	8,360.29	66,160.32	80,710.15	53,096.48	68,030.43	77,844.75	58,050.93	126,121.94	136,949.97	116,224.93	18,315.91	22,821.97	14,716.30	4,811.77	5,598.55	4,196.67
204	Chad	Rate	579.85	978.65	316.50	2,504.64	3,055.45	2,010.08	2,575.43	2,946.98	2,197.64	4,774.61	5,184.53	4,399.94	693.39	863.97	557.12	182.16	211.94	158.87
205	Cote d'Ivoire	Number	23,362.29	38,318.97	13,207.87	81,694.72	100,021.92	65,030.74	86,489.44	99,470.89	73,897.67	164,318.97	179,927.39	150,900.22	24,725.22	30,553.53	19,596.33	6,542.29	7,611.81	5,706.42
205	Cote d'Ivoire	Rate	656.48	1,076.77	371.14	2,295.64	2,810.63	1,827.38	2,430.37	2,795.15	2,076.54	4,617.39	5,055.99	4,240.32	694.78	858.56	550.66	183.84	213.89	160.35
206	The Gambia	Number	3,435.73	5,037.21	2,230.17	9,536.48	11,797.48	7,532.48	9,199.79	10,550.88	7,832.14	15,370.48	18,381.50	12,768.93	2,556.04	3,161.61	2,024.48	680.67	791.99	593.61
206	The Gambia	Rate	937.11	1,373.92	608.29	2,601.11	3,217.80	2,054.51	2,509.27	2,877.79	2,136.24	4,192.35	5,013.62	3,482.77	697.17	862.34	552.18	185.66	216.02	161.91
207	Ghana	Number	42,534.95	66,718.48	25,229.55	98,420.58	125,579.88	77,096.44	102,240.41	117,867.13	86,864.95	177,143.32	199,352.53	160,016.07	30,584.39	38,298.88	24,494.63	8,147.86	9,481.04	7,104.38
207	Ghana	Rate	978.62	1,535.02	580.47	2,264.40	2,889.26	1,773.79	2,352.28	2,711.81	1,998.53	4,075.60	4,586.58	3,681.55	703.67	881.16	563.56	187.46	218.13	163.45
208	Guinea	Number	13,398.06	21,655.56	7,805.44	55,120.64	68,084.48	43,714.27	52,031.34	59,802.15	44,577.86	90,531.95	106,954.00	74,928.17	14,089.53	17,416.75	11,247.73	3,743.97	4,356.03	3,265.59
208	Guinea	Rate	658.42	1,064.22	383.58	2,708.79	3,345.86	2,148.24	2,556.97	2,938.85	2,190.68	4,449.00	5,256.02	3,682.18	692.40	855.91	552.75	183.99	214.07	160.48

Table S2. Global and country-specific prevalence of developmental disabilities in 2016 (with 95% uncertainty interval)

			Epilepsy			Intellectual disability			Hearing loss			Vision loss			Autism spectrum disorder			Attention-deficit/hyperactivity disorder		
Loc-ID	Location	Metric	Value	Upper limit	Lower limit	Value	Upper limit	Lower limit	Value	Upper limit	Lower limit	Value	Upper limit	Lower limit	Value	Upper limit	Lower limit	Value	Upper limit	Lower limit
209	Guinea-Bissau	Number	1,910.71	3,120.08	1,143.79	8,377.55	10,264.18	6,696.04	7,890.48	9,061.63	6,716.90	13,493.89	15,894.53	11,243.99	2,158.03	2,680.60	1,736.91	573.92	667.76	500.56
209	Guinea-Bissau	Rate	616.42	1,006.57	369.00	2,702.68	3,311.33	2,160.21	2,545.55	2,923.38	2,166.94	4,353.27	5,127.74	3,627.43	696.20	864.79	560.34	185.15	215.43	161.49
210	Liberia	Number	5,449.46	8,517.36	3,050.58	20,748.50	25,321.70	16,597.63	18,146.55	20,861.71	15,437.20	31,429.07	37,573.90	26,139.01	4,986.26	6,231.35	4,019.87	1,327.00	1,544.15	1,157.02
210	Liberia	Rate	764.49	1,194.88	427.96	2,910.77	3,552.33	2,328.45	2,545.74	2,926.65	2,165.65	4,409.12	5,271.17	3,666.99	699.51	874.18	563.94	186.16	216.63	162.32
211	Mali	Number	18,499.67	30,099.66	10,793.14	81,692.46	99,965.11	65,310.25	82,673.80	95,213.69	70,523.59	112,668.03	127,658.36	100,757.68	21,928.60	27,281.16	17,483.77	5,800.36	6,749.13	5,058.16
211	Mali	Rate	587.29	955.54	342.64	2,593.39	3,173.47	2,073.33	2,624.55	3,022.64	2,238.83	3,576.74	4,052.62	3,198.64	696.14	866.06	555.04	184.14	214.26	160.58
212	Mauritania	Number	5,388.79	8,333.41	3,200.73	11,684.99	14,732.54	9,070.20	12,110.29	13,894.32	10,224.31	30,385.85	33,398.94	27,835.35	3,606.97	4,471.85	2,892.19	961.83	1,119.20	838.68
212	Mauritania	Rate	1,047.64	1,620.11	622.26	2,271.70	2,864.18	1,763.35	2,354.38	2,701.22	1,987.73	5,907.37	6,493.15	5,411.52	701.24	869.38	562.28	186.99	217.59	163.05
213	Niger	Number	20,908.16	34,755.66	11,794.11	113,666.29	138,513.98	90,485.80	100,250.36	114,976.80	86,223.21	191,329.58	229,289.60	159,145.39	25,614.38	31,880.13	20,645.82	6,727.17	7,827.87	5,865.70
213	Niger	Rate	567.60	943.53	320.18	3,085.74	3,760.29	2,456.45	2,721.54	3,121.32	2,340.74	5,194.10	6,224.62	4,320.38	695.36	865.46	560.48	182.63	212.51	159.24
214	Nigeria	Number	200,665.61	334,181.09	113,012.73	625,430.23	785,275.72	494,245.94	765,042.97	872,347.51	661,204.53	1,112,426.35	1,330,972.17	920,430.08	206,735.87	258,640.64	164,484.40	55,203.06	63,081.13	49,102.43
214	Nigeria	Rate	679.44	1,131.52	382.65	2,117.67	2,658.90	1,673.49	2,590.39	2,953.72	2,238.80	3,766.61	4,506.60	3,116.53	700.00	875.74	556.94	186.91	213.59	166.26
215	Sao Tome and Principe	Number	322.31	490.63	188.37	733.47	914.70	568.54	805.81	928.69	684.14	1,312.46	1,543.09	1,095.08	231.32	288.37	185.24	62.20	72.37	54.24
215	Sao Tome and Principe	Rate	967.94	1,473.43	565.68	2,202.69	2,746.95	1,707.39	2,419.93	2,788.95	2,054.54	3,941.46	4,634.07	3,288.65	694.67	866.01	556.30	186.78	217.33	162.90
216	Senegal	Number	23,481.74	35,465.46	14,017.91	62,082.54	76,865.63	49,227.08	64,005.54	73,404.19	54,599.47	104,747.61	117,392.48	94,797.89	17,731.24	21,893.20	14,139.76	4,717.83	5,489.41	4,114.38
216	Senegal	Rate	923.75	1,395.18	551.45	2,442.27	3,023.82	1,936.55	2,517.92	2,887.65	2,147.89	4,120.68	4,618.11	3,729.26	697.53	861.26	556.25	185.60	215.95	161.86
217	Sierra Leone	Number	6,162.66	10,393.59	3,424.21	27,371.47	33,497.20	21,951.23	23,285.27	26,924.89	19,780.70	43,489.81	51,914.89	36,029.66	7,173.88	8,944.67	5,688.61	1,907.40	2,219.14	1,663.82
217	Sierra Leone	Rate	592.59	999.43	329.27	2,632.00	3,221.05	2,110.80	2,239.08	2,589.06	1,902.09	4,181.92	4,992.07	3,464.56	689.83	860.11	547.01	183.41	213.39	159.99
218	Togo	Number	8,876.71	13,612.05	5,393.48	29,489.97	36,396.44	23,470.03	26,879.79	30,952.85	22,835.95	44,527.38	52,285.95	37,365.89	7,586.01	9,388.77	6,007.02	2,019.52	2,349.61	1,761.60
218	Togo	Rate	811.60	1,244.56	493.13	2,696.28	3,327.74	2,145.87	2,457.63	2,830.03	2,087.90	4,071.16	4,780.53	3,416.38	693.59	858.42	549.22	184.65	214.83	161.06
298	American Samoa	Number	30.18	42.79	17.44	65.64	77.82	53.94	152.39	176.18	128.84	235.28	266.69	207.57	52.38	64.68	41.61	9.47	11.11	8.16
298	American Samoa	Rate	404.39	573.27	233.64	879.40	1,042.64	722.61	2,041.60	2,360.37	1,726.13	3,152.23	3,572.98	2,780.97	701.79	866.52	557.44	126.81	148.83	109.32
305	Bermuda	Number	18.60	29.08	9.51	34.98	40.94	30.12	80.13	94.06	66.77	130.08	145.84	116.36	31.77	39.31	25.33	12.53	14.47	10.98
305	Bermuda	Rate	423.94	662.98	216.71	797.42	933.37	686.75	1,826.79	2,144.31	1,522.16	2,965.57	3,324.86	2,652.82	724.36	896.14	577.57	285.62	329.92	250.26
349	Greenland	Number	10.43	16.64	5.29	48.52	54.96	42.95	43.74	52.37	35.76	51.53	58.94	45.76	24.99	30.56	19.91	7.76	9.52	6.39
349	Greenland	Rate	305.55	487.67	155.00	1,421.86	1,610.34	1,258.63	1,281.68	1,534.52	1,047.77	1,509.98	1,727.00	1,340.97	732.23	895.41	583.54	227.25	278.97	187.13
351	Guam	Number	66.50	94.88	38.68	130.41	156.34	106.73	322.56	375.39	271.10	488.04	560.02	427.52	116.18	145.17	93.27	21.01	24.66	18.11
351	Guam	Rate	397.07	566.49	230.92	778.63	933.47	637.25	1,925.91	2,241.36	1,618.70	2,913.99	3,343.76	2,552.64	693.71	866.80	556.92	125.46	147.23	108.15
376	Northern Mariana Islands	Number	63.06	90.71	36.02	130.52	156.05	106.79	301.64	351.32	254.83	451.55	517.84	397.68	106.96	132.94	85.07	19.14	22.47	16.50
376	Northern Mariana Islands	Rate	413.25	594.42	236.07	855.34	1,022.66	699.79	1,976.72	2,302.27	1,669.95	2,959.11	3,393.49	2,606.07	700.95	871.19	557.50	125.44	147.22	108.15
385	Puerto Rico	Number	1,319.47	2,104.55	738.58	2,475.58	3,171.48	2,048.45	3,812.01	4,473.33	3,173.69	6,173.49	7,076.59	5,490.69	1,538.15	1,907.88	1,221.20	604.02	691.24	539.79
385	Puerto Rico	Rate	629.59	1,004.19	352.42	1,181.23	1,513.29	977.43	1,818.92	2,134.47	1,514.34	2,945.71	3,376.63	2,619.91	733.93	910.36	582.70	288.21	329.83	257.56
422	Virgin Islands, U.S.	Number	33.59	51.32	19.83	59.74	79.40	47.72	100.36	117.75	83.51	161.46	184.55	143.07	40.58	49.72	32.03	16.13	18.64	14.12
422	Virgin Islands, U.S.	Rate	608.33	929.37	359.19	1,081.77	1,437.95	864.25	1,817.41	2,132.46	1,512.36	2,923.94	3,342.13	2,590.98	734.87	900.35	580.03	292.15	337.51	255.78
435	South Sudan	Number	17,184.50	29,488.98	9,829.33	59,873.79	75,217.18	45,863.19	84,151.05	99,752.27	69,653.41	171,992.11	205,003.52	143,532.30	20,131.35	24,776.16	16,110.63	5,054.38	5,910.18	4,372.73
435	South Sudan	Rate	600.80	1,030.98	343.65	2,093.28	2,629.71	1,603.45	2,942.05	3,487.50	2,435.19	6,013.12	7,167.25	5,018.12	703.82	866.21	563.25	176.71	206.63	152.88
522	Sudan	Number	26,439.77	53,497.74	13,554.95	129,975.65	160,948.23	105,579.71	87,250.48	103,535.84	72,416.26	227,651.79	282,520.04	190,493.57	32,477.90	40,132.08	26,075.89	6,248.68	7,837.54	4,966.76
522	Sudan	Rate	630.51	1,275.75	323.24	3,099.51	3,838.11	2,517.75	2,080.65	2,469.01	1,726.90	5,428.78	6,737.22	4,542.67	774.50	957.02	621.83	149.01	186.90	118.44

Table S3. Global and country-specific YLDs of developmental disabilities in 2016 (with 95% uncertainty interval)																				
			Epilepsy			Intellectual disability			Hearing loss			Vision loss			Autism spectrum disorder			Attention-deficit/hyperactivity disorder		
Loc-ID	Location	Metric	Value	Upper limit	Lower limit	Value	Upper limit	Lower limit	Value	Upper limit	Lower limit	Value	Upper limit	Lower limit	Value	Upper limit	Lower limit	Value	Upper limit	Lower limit
1	Global	Number	1,468,477.56	2,256,241.20	1,022,639.02	1,684,148.69	2,541,783.70	1,200,212.68	1,098,261.54	1,529,677.07	741,048.17	1,096,187.12	1,827,212.69	694,904.17	644,290.27	929,338.81	413,472.79	10,826.08	17,138.21	6,477.41
1	Global	Rate	232.36	357.02	161.82	266.49	402.20	189.92	173.78	242.05	117.26	173.46	289.13	109.96	101.95	147.05	65.43	1.71	2.71	1.02
6	China	Number	76,760.99	102,415.12	55,889.72	82,090.29	107,325.90	60,516.83	78,121.40	109,660.96	53,881.27	59,395.31	86,954.53	39,718.78	60,128.85	88,015.12	38,044.79	1,617.13	2,628.76	968.42
6	China	Rate	126.55	168.84	92.14	135.33	176.94	99.77	128.79	180.79	88.83	97.92	143.35	65.48	99.13	145.10	62.72	2.67	4.33	1.60
7	North Korea	Number	3,838.06	6,079.26	1,984.19	4,122.25	5,565.19	2,928.71	6,432.49	9,141.05	4,252.85	2,655.83	4,177.12	1,668.23	2,809.70	4,021.04	1,785.89	74.95	133.85	35.65
7	North Korea	Rate	129.71	205.45	67.06	139.31	188.08	98.98	217.39	308.92	143.73	89.75	141.17	56.38	94.95	135.89	60.35	2.53	4.52	1.20
8	Taiwan	Number	1,212.28	2,124.47	553.26	896.70	1,274.81	625.74	1,491.21	2,057.61	986.72	691.13	1,066.36	431.85	997.33	1,459.49	635.55	26.49	44.99	12.47
8	Taiwan	Rate	119.55	209.50	54.56	88.43	125.71	61.71	147.05	202.91	97.30	68.15	105.16	42.59	98.35	143.93	62.67	2.61	4.44	1.23
10	Cambodia	Number	4,120.78	6,981.64	1,989.67	3,325.33	4,635.51	2,312.13	3,370.92	4,865.71	2,144.83	2,937.91	4,383.01	1,978.18	1,848.01	2,678.98	1,145.65	19.42	37.34	6.66
10	Cambodia	Rate	218.75	370.61	105.62	176.52	246.07	122.74	178.94	258.29	113.86	155.96	232.67	105.01	98.10	142.21	60.82	1.03	1.98	0.35
11	Indonesia	Number	50,479.25	70,431.22	34,715.75	36,368.11	50,506.38	26,369.27	32,629.54	45,673.02	22,680.73	30,004.62	45,354.55	19,960.39	22,774.37	32,989.16	14,691.90	236.46	385.54	133.78
11	Indonesia	Rate	222.12	309.91	152.75	160.03	222.24	116.03	143.58	200.97	99.80	132.03	199.57	87.83	100.21	145.16	64.65	1.04	1.70	0.59
12	Laos	Number	2,018.99	3,449.72	939.33	1,928.87	2,687.30	1,358.22	1,954.77	2,851.70	1,258.40	1,051.81	1,616.26	659.59	1,099.78	1,622.15	701.72	11.33	21.99	4.71
12	Laos	Rate	178.62	305.20	83.10	170.65	237.75	120.16	172.94	252.29	111.33	93.05	142.99	58.35	97.30	143.51	62.08	1.00	1.95	0.42
13	Malaysia	Number	5,378.17	9,062.64	2,640.13	3,139.74	4,453.07	2,165.18	1,747.37	2,515.67	1,131.57	2,328.28	3,495.78	1,569.04	2,578.68	3,765.77	1,588.89	20.32	38.96	7.17
13	Malaysia	Rate	209.46	352.95	102.82	122.28	173.43	84.32	68.05	97.98	44.07	90.68	136.15	61.11	100.43	146.66	61.88	0.79	1.52	0.28
14	Maldives	Number	54.03	92.49	26.25	38.41	54.47	26.42	43.67	62.96	27.95	32.18	48.39	21.10	31.37	46.04	19.85	0.33	0.62	0.11
14	Maldives	Rate	172.39	295.10	83.74	122.55	173.78	84.29	139.32	200.87	89.16	102.67	154.38	67.31	100.09	146.90	63.32	1.05	1.99	0.36
15	Myanmar	Number	9,992.49	16,491.84	4,871.21	7,645.07	10,859.42	5,307.20	10,914.38	15,224.18	7,080.36	7,835.80	11,303.80	5,316.07	4,584.28	6,670.46	2,903.03	49.24	96.17	18.02
15	Myanmar	Rate	214.54	354.09	104.59	164.14	233.16	113.95	234.34	326.87	152.02	168.24	242.70	114.14	98.43	143.22	62.33	1.06	2.06	0.39
16	Philippines	Number	24,645.04	40,186.80	12,324.75	19,135.51	26,967.20	13,325.86	17,384.47	25,191.73	11,076.87	16,152.80	24,979.79	10,157.16	11,529.47	16,829.18	7,398.45	119.64	230.05	43.57
16	Philippines	Rate	213.06	347.42	106.55	165.43	233.13	115.20	150.29	217.78	95.76	139.64	215.95	87.81	99.67	145.49	63.96	1.03	1.99	0.38
17	Sri Lanka	Number	3,808.00	6,241.98	1,992.35	2,288.40	3,313.59	1,570.73	2,482.38	3,496.13	1,626.74	1,902.10	2,958.48	1,226.76	1,526.48	2,233.23	962.63	15.99	31.12	6.00
17	Sri Lanka	Rate	251.94	412.97	131.81	151.40	219.23	103.92	164.24	231.31	107.63	125.84	195.74	81.16	100.99	147.75	63.69	1.06	2.06	0.40
18	Thailand	Number	7,106.16	11,439.09	3,670.70	5,765.26	8,187.12	4,085.85	4,638.28	6,601.72	3,068.48	2,811.28	4,551.93	1,790.82	3,142.64	4,631.45	1,991.65	45.11	83.34	19.11
18	Thailand	Rate	229.69	369.74	118.65	186.35	264.63	132.06	149.92	213.38	99.18	90.87	147.13	57.88	101.58	149.70	64.37	1.46	2.69	0.62
19	Timor-Leste	Number	310.64	532.83	138.51	264.12	359.20	184.61	276.56	400.47	179.92	268.49	391.60	177.28	162.11	238.21	104.29	1.72	3.34	0.62
19	Timor-Leste	Rate	187.66	321.89	83.68	159.56	217.00	111.52	167.07	241.93	108.69	162.20	236.57	107.10	97.93	143.90	63.00	1.04	2.02	0.37
20	Vietnam	Number	13,270.76	21,844.18	6,810.85	10,324.12	15,326.15	7,005.73	10,283.45	14,277.92	6,830.86	9,502.54	14,452.75	6,208.49	7,685.25	11,157.21	4,894.43	79.17	152.92	30.48
20	Vietnam	Rate	175.69	289.19	90.17	136.68	202.90	92.75	136.14	189.02	90.43	125.80	191.34	82.19	101.74	147.71	64.80	1.05	2.02	0.40
22	Fiji	Number	94.31	150.46	49.97	71.77	102.95	50.41	72.58	101.22	48.55	58.62	90.98	37.95	50.15	74.33	31.69	0.79	1.40	0.34
22	Fiji	Rate	185.52	295.99	98.31	141.19	202.52	99.17	142.77	199.12	95.51	115.31	178.98	74.66	98.65	146.22	62.34	1.55	2.76	0.66
23	Kiribati	Number	22.01	35.14	11.51	22.95	31.67	16.51	28.42	39.63	18.67	16.28	24.84	10.34	13.24	19.47	8.42	0.21	0.37	0.09
23	Kiribati	Rate	162.82	259.94	85.17	169.78	234.28	122.12	210.21	293.21	138.15	120.46	183.75	76.51	97.95	144.04	62.32	1.54	2.76	0.69
24	Marshall Islands	Number	16.58	26.79	9.03	18.21	32.39	11.67	17.44	24.20	11.61	11.54	17.24	7.55	9.76	14.48	6.11	0.15	0.28	0.07
24	Marshall Islands	Rate	167.34	270.35	91.13	183.80	326.86	117.78	175.97	244.22	117.19	116.43	174.00	76.16	98.49	146.17	61.65	1.52	2.81	0.67
25	Federated States of Micronesia	Number	15.71	24.48	8.96	14.95	20.52	10.78	16.43	23.06	10.94	11.00	16.51	7.02	9.50	14.04	6.09	0.15	0.27	0.06
25	Federated States of Micronesia	Rate	164.17	255.75	93.61	156.18	214.36	112.66	171.64	240.90	114.34	114.91	172.53	73.33	99.25	146.66	63.63	1.56	2.77	0.67
26	Papua New Guinea	Number	1,572.15	2,612.63	853.34	1,826.99	2,554.76	1,327.97	2,272.30	3,187.09	1,513.13	1,519.31	2,269.34	968.15	1,062.39	1,541.94	662.91	16.13	29.06	7.36
26	Papua New Guinea	Rate	145.89	242.45	79.19	169.54	237.08	123.23	210.87	295.76	140.42	140.99	210.59	89.84	98.59	143.09	61.52	1.50	2.70	0.68
27	Samoa	Number	42.98	67.15	24.11	37.80	52.39	26.65	48.17	68.52	31.98	31.60	47.51	20.19	27.45	40.00	17.27	0.43	0.82	0.19
27	Samoa	Rate	156.40	244.39	87.73	137.55	190.64	96.97	175.30	249.36	116.38	115.00	172.91	73.46	99.89	145.55	62.85	1.58	2.99	0.71
28	Solomon Islands	Number	141.96	218.11	82.15	142.38	192.96	103.24	174.71	245.42	118.16	103.31	154.88	66.61	82.90	123.72	52.78	1.28	2.36	0.58
28	Solomon Islands	Rate	170.36	261.76	98.59	170.87	231.57	123.89	209.67	294.53	141.81	123.98	185.87	79.94	99.49	148.48	63.35	1.54	2.84	0.70
29	Tonga	Number	22.04	35.20	11.95	20.32	29.02	14.19	23.15	32.64	15.65	14.75	23.14	9.12	13.24	19.28	8.46	0.21	0.37	0.09
29	Tonga	Rate	163.74	261.51	88.76	150.94	215.61	105.47	172.00	242.51	116.27	109.58	171.91	67.76	98.35	143.25	62.89	1.53	2.76	0.66
30	Vanuatu	Number	64.65	102.81	35.83	64.28	87.19	46.66	79.72	110.49	52.36	46.58	70.51	29.71	40.22	59.57	25.58	0.62	1.13	0.27
30	Vanuatu	Rate	159.35	253.40	88.32	158.43	214.89	115.01	196.48	272.33	129.06	114.80	173.79	73.24	99.12	146.82	63.05	1.52	2.78	0.65
33	Armenia	Number	427.12	749.53	206.28	560.16	843.95	385.14	303.79	426.34	198.32	397.52	659.15	245.94	230.30	335.52	149.78	3.48	6.37	1.56
33	Armenia	Rate	192.94	338.59	93.18	253.04	381.24	173.98	137.23	192.59	89.59	179.57	297.76	111.10	104.03	151.56	67.66	1.57	2.88	0.71
34	Azerbaijan	Number	1,489.51	2,901.14	638.39	1,878.71	2,589.79	1,349.06	1,230.91	1,763.61	815.82	1,204.94	1,809.86	760.35	987.51	1,428.97	633.01	15.36	27.36	6.77
34	Azerbaijan	Rate	155.69	303.24	66.73	196.37	270.69	141.01	128.66	184.34	85.27	125.94	189.17	79.47	103.22	149.36	66.16	1.61	2.86	0.71
35	Georgia	Number	597.82	1,068.25	290.55	744.49	1,047.46	529.50	456.52	644.67	299.85	522.01	801.20	340.56	343.75	501.78	219.48	5.28	10.02	2.18
35	Georgia	Rate	179.42	320.60	87.20	223.44	314.37	158.91	137.01	193.48	89.99	156.66	240.46	102.21	103.17	150.59	65.87	1.58	3.01	0.65

Table S3. Global and country-specific YLDs of developmental disabilities in 2016 (with 95% uncertainty interval)																				
			Epilepsy			Intellectual disability			Hearing loss			Vision loss			Autism spectrum disorder			Attention-deficit/hyperactivity disorder		
Loc-ID	Location	Metric	Value	Upper limit	Lower limit	Value	Upper limit	Lower limit	Value	Upper limit	Lower limit	Value	Upper limit	Lower limit	Value	Upper limit	Lower limit	Value	Upper limit	Lower limit
36	Kazakhstan	Number	3,396.18	5,801.41	1,704.11	4,281.21	5,995.04	3,042.78	2,465.43	3,499.01	1,617.17	2,970.44	4,583.43	1,917.46	2,007.28	3,009.49	1,299.86	30.95	54.78	13.85
36	Kazakhstan	Rate	173.35	296.12	86.98	218.53	306.00	155.31	125.84	178.60	82.55	151.62	233.95	97.87	102.46	153.61	66.35	1.58	2.80	0.71
37	Kyrgyzstan	Number	1,183.88	2,038.22	589.66	1,858.46	2,487.93	1,326.09	1,212.95	1,710.46	800.73	1,122.33	1,711.15	703.01	761.05	1,113.90	472.48	11.74	20.85	5.33
37	Kyrgyzstan	Rate	158.34	272.61	78.87	248.57	332.76	177.36	162.23	228.77	107.10	150.11	228.86	94.03	101.79	148.98	63.19	1.57	2.79	0.71
38	Mongolia	Number	631.96	1,076.94	302.45	924.29	1,265.35	660.02	528.64	749.73	353.59	553.36	849.40	360.33	375.07	547.40	235.03	5.83	10.49	2.50
38	Mongolia	Rate	169.74	289.25	81.24	248.26	339.86	177.27	141.99	201.37	94.97	148.63	228.14	96.78	100.74	147.03	63.13	1.57	2.82	0.67
39	Tajikistan	Number	1,929.86	3,309.33	966.16	3,100.96	4,209.57	2,220.89	1,942.94	2,738.27	1,279.12	1,807.50	2,784.03	1,161.25	1,141.31	1,673.69	713.47	17.82	31.52	8.08
39	Tajikistan	Rate	169.51	290.67	84.86	272.37	369.75	195.07	170.66	240.51	112.35	158.76	244.53	102.00	100.25	147.01	62.67	1.57	2.77	0.71
40	Turkmenistan	Number	966.53	1,755.29	440.19	1,346.36	1,935.52	959.96	744.10	1,051.57	489.59	835.51	1,310.00	508.84	580.53	861.18	370.87	9.07	16.36	4.07
40	Turkmenistan	Rate	167.35	303.93	76.22	233.12	335.13	166.22	128.84	182.08	84.77	144.67	226.83	88.11	100.52	149.11	64.22	1.57	2.83	0.70
41	Uzbekistan	Number	5,614.31	10,026.97	2,698.49	7,804.00	10,746.65	5,585.92	4,864.98	6,863.26	3,186.20	4,896.45	7,574.32	3,127.76	3,388.52	5,000.31	2,141.59	52.68	93.04	22.18
41	Uzbekistan	Rate	168.06	300.15	80.78	233.61	321.69	167.21	145.63	205.45	95.38	146.57	226.73	93.63	101.43	149.68	64.11	1.58	2.79	0.66
43	Albania	Number	267.53	495.96	113.21	346.60	467.44	249.67	253.82	357.45	165.81	201.65	303.83	127.11	194.74	288.21	122.67	2.92	5.31	1.23
43	Albania	Rate	144.38	267.66	61.09	187.05	252.26	134.74	136.98	192.90	89.48	108.82	163.97	68.60	105.10	155.54	66.20	1.58	2.87	0.66
44	Bosnia and Herzegovina	Number	238.23	444.69	119.59	338.40	461.39	241.21	234.73	332.51	155.11	218.14	335.34	137.00	182.70	268.78	116.63	2.76	5.16	1.25
44	Bosnia and Herzegovina	Rate	139.40	260.22	69.98	198.02	269.99	141.15	137.36	194.57	90.76	127.65	196.23	80.17	106.91	157.28	68.25	1.61	3.02	0.73
45	Bulgaria	Number	444.28	815.35	209.17	691.90	935.92	485.68	394.76	560.62	259.51	375.41	586.91	237.02	352.16	521.29	221.28	5.36	9.96	2.46
45	Bulgaria	Rate	133.57	245.12	62.88	208.01	281.37	146.01	118.68	168.54	78.02	112.86	176.45	71.26	105.87	156.72	66.52	1.61	3.00	0.74
46	Croatia	Number	228.09	409.09	114.24	498.43	670.02	359.67	229.94	322.39	151.42	225.10	347.49	142.38	209.89	306.70	133.79	3.18	5.79	1.37
46	Croatia	Rate	115.93	207.92	58.06	253.34	340.55	182.81	116.87	163.86	76.96	114.41	176.61	72.37	106.68	155.89	68.00	1.62	2.94	0.70
47	Czech Republic	Number	637.31	1,146.19	284.14	944.81	1,300.69	666.25	599.89	844.79	400.67	565.34	856.95	362.14	579.85	844.45	361.69	8.63	15.20	3.78
47	Czech Republic	Rate	116.99	210.40	52.16	173.43	238.76	122.30	110.12	155.07	73.55	103.78	157.31	66.48	106.44	155.01	66.39	1.58	2.79	0.69
48	Hungary	Number	566.74	1,038.25	277.36	833.32	1,126.48	600.75	516.34	723.95	343.24	476.42	722.76	301.47	479.72	696.48	308.18	7.27	13.36	3.25
48	Hungary	Rate	125.21	229.38	61.28	184.11	248.88	132.73	114.08	159.95	75.83	105.26	159.68	66.61	105.99	153.88	68.09	1.61	2.95	0.72
49	Macedonia	Number	145.26	260.10	67.71	228.48	308.97	165.39	143.01	201.93	95.27	133.28	206.40	84.67	118.07	172.62	75.23	1.78	3.25	0.78
49	Macedonia	Rate	129.42	231.75	60.33	203.58	275.30	147.36	127.42	179.92	84.88	118.75	183.91	75.44	105.20	153.80	67.03	1.58	2.90	0.69
50	Montenegro	Number	46.73	86.50	23.32	72.09	98.55	50.86	44.83	63.05	30.00	42.76	66.07	26.86	38.90	57.61	24.73	0.58	1.02	0.26
50	Montenegro	Rate	128.31	237.49	64.02	197.93	270.58	139.65	123.09	173.10	82.38	117.40	181.41	73.75	106.82	158.17	67.89	1.61	2.81	0.70
51	Poland	Number	1,927.73	3,621.47	959.98	3,445.78	4,739.36	2,434.50	2,122.74	2,988.85	1,398.02	2,476.76	3,765.81	1,577.14	1,976.19	2,847.01	1,256.11	29.57	54.05	12.61
51	Poland	Rate	103.12	193.73	51.35	184.33	253.53	130.23	113.55	159.89	74.79	132.49	201.45	84.37	105.71	152.30	67.19	1.58	2.89	0.67
52	Romania	Number	1,134.69	2,014.44	554.86	1,732.44	2,301.85	1,233.20	1,043.00	1,478.76	699.62	910.46	1,364.18	577.57	919.53	1,337.70	576.13	13.99	25.71	6.11
52	Romania	Rate	129.18	229.34	63.17	197.23	262.06	140.39	118.74	168.35	79.65	103.65	155.31	65.75	104.68	152.29	65.59	1.59	2.93	0.70
53	Serbia	Number	628.73	1,212.97	285.21	993.51	1,337.55	718.81	538.04	757.08	357.14	493.04	758.53	302.91	438.00	637.75	274.87	6.57	11.97	2.88
53	Serbia	Rate	151.97	293.19	68.94	240.14	323.30	173.75	130.05	183.00	86.32	119.17	183.35	73.22	105.87	154.15	66.44	1.59	2.89	0.70
54	Slovakia	Number	334.32	631.37	150.27	589.26	781.06	424.98	316.80	447.66	208.12	269.56	412.24	171.78	304.77	451.53	191.96	4.68	8.80	2.12
54	Slovakia	Rate	115.96	218.99	52.12	204.39	270.91	147.41	109.88	155.27	72.19	93.50	142.98	59.58	105.71	156.61	66.58	1.62	3.05	0.73
55	Slovenia	Number	119.86	239.05	54.40	238.64	323.88	173.09	112.77	161.62	73.65	110.47	168.43	69.32	113.07	165.86	70.78	1.69	3.09	0.78
55	Slovenia	Rate	113.21	225.79	51.38	225.39	305.91	163.48	106.51	152.65	69.57	104.34	159.08	65.48	106.79	156.65	66.85	1.59	2.92	0.74
57	Belarus	Number	704.42	1,176.77	372.36	1,405.81	1,914.89	998.95	703.62	999.03	461.42	648.43	1,031.88	397.45	611.70	897.28	380.96	9.16	16.49	4.08
57	Belarus	Rate	122.32	204.34	64.66	244.11	332.50	173.46	122.18	173.47	80.12	112.59	179.18	69.01	106.22	155.80	66.15	1.59	2.86	0.71
58	Estonia	Number	71.48	121.81	37.47	150.78	203.95	109.67	76.71	107.67	50.52	53.79	84.86	34.21	72.50	107.10	46.61	1.08	1.96	0.50
58	Estonia	Rate	104.29	177.73	54.68	219.99	297.58	160.02	111.93	157.10	73.71	78.48	123.82	49.92	105.79	156.26	68.01	1.58	2.86	0.73
59	Latvia	Number	110.99	189.41	57.20	273.57	362.28	199.21	123.40	176.18	80.46	113.68	177.11	71.67	111.68	162.78	70.46	1.64	2.96	0.75
59	Latvia	Rate	104.91	179.02	54.06	258.56	342.40	188.28	116.63	166.52	76.04	107.45	167.40	67.74	105.55	153.85	66.59	1.55	2.80	0.71
60	Lithuania	Number	176.48	301.34	90.37	400.05	544.27	283.28	170.64	237.70	113.79	165.08	255.90	103.97	159.94	234.59	99.04	2.39	4.46	1.05
60	Lithuania	Rate	117.41	200.49	60.13	266.16	362.11	188.48	113.53	158.15	75.71	109.83	170.26	69.18	106.41	156.08	65.90	1.59	2.97	0.70
61	Moldova	Number	392.92	719.86	199.76	716.53	1,049.85	497.58	338.38	472.69	222.17	384.65	696.79	221.23	236.22	344.42	148.34	3.56	6.27	1.70
61	Moldova	Rate	174.71	320.08	88.82	318.61	466.82	221.25	150.46	210.18	98.79	171.03	309.82	98.37	105.03	153.15	65.96	1.58	2.79	0.75
62	Russia	Number	13,076.64	21,900.97	6,812.60	22,361.74	32,254.51	15,750.62	11,109.75	15,570.84	7,346.85	14,105.15	22,039.63	8,900.74	9,932.73	14,719.98	6,336.35	149.20	270.55	66.12
62	Russia	Rate	139.45	233.55	72.65	238.47	343.96	167.97	118.47	166.05	78.35	150.42	235.03	94.92	105.92	156.97	67.57	1.59	2.89	0.71
63	Ukraine	Number	3,423.01	5,831.63	1,860.16	6,000.00	8,301.72	4,261.51	3,265.12	4,616.89	2,147.40	3,118.71	4,955.61	1,911.97	2,608.10	3,811.93	1,646.24	39.09	71.49	17.64
63	Ukraine	Rate	139.38	237.45	75.74	244.30	338.02	173.52	132.95	187.99	87.44	126.99	201.78	77.85	106.19	155.21	67.03	1.59	2.91	0.72
66	Brunei	Number	43.54	82.77	18.59	59.34	81.61	41.43	21.03	30.54	13.33	39.26	59.44	25.20	37.23	54.83	23.88	0.45	0.84	0.18
66	Brunei	Rate	132.05	251.01	56.37	179.95	247.50	125.64	63.79	92.61	40.43	119.05	180.25	76.41	112.91	166.27	72.42	1.37	2.56	0.56

Table S3. Global and country-specific YLDs of developmental disabilities in 2016 (with 95% uncertainty interval)																				
			Epilepsy			Intellectual disability			Hearing loss			Vision loss			Autism spectrum disorder			Attention-deficit/hyperactivity disorder		
Loc-ID	Location	Metric	Value	Upper limit	Lower limit	Value	Upper limit	Lower limit	Value	Upper limit	Lower limit	Value	Upper limit	Lower limit	Value	Upper limit	Lower limit	Value	Upper limit	Lower limit
67	Japan	Number	3,747.16	5,488.45	2,514.80	9,253.63	12,436.67	6,576.65	3,695.60	5,162.37	2,446.89	5,366.94	7,911.34	3,578.89	6,099.65	8,770.62	3,881.87	67.07	107.59	38.26
67	Japan	Rate	74.71	109.42	50.14	184.49	247.95	131.12	73.68	102.92	48.78	107.00	157.73	71.35	121.61	174.86	77.39	1.34	2.15	0.76
68	South Korea	Number	2,035.63	3,702.20	953.70	3,720.33	5,069.31	2,554.94	1,531.69	2,153.74	1,004.94	2,458.54	3,635.10	1,589.91	2,282.49	3,305.81	1,432.21	28.81	51.25	11.52
68	South Korea	Rate	95.71	174.06	44.84	174.91	238.33	120.12	72.01	101.26	47.25	115.59	170.90	74.75	107.31	155.42	67.34	1.35	2.41	0.54
69	Singapore	Number	140.49	255.49	62.21	249.07	340.19	172.36	111.68	158.57	72.42	186.83	276.06	123.86	203.17	295.00	128.05	2.36	4.35	1.00
69	Singapore	Rate	79.43	144.45	35.17	140.82	192.34	97.45	63.14	89.65	40.95	105.63	156.08	70.03	114.87	166.79	72.40	1.34	2.46	0.57
71	Australia	Number	1,222.71	2,229.80	619.50	2,756.60	3,749.22	1,939.83	962.03	1,372.97	627.35	1,340.67	2,021.91	870.23	1,855.92	2,693.92	1,180.01	59.67	100.32	30.89
71	Australia	Rate	82.03	149.60	41.56	184.94	251.54	130.14	64.54	92.11	42.09	89.95	135.65	58.38	124.51	180.74	79.17	4.00	6.73	2.07
72	New Zealand	Number	255.78	458.94	121.44	638.61	855.96	466.25	198.10	281.45	126.81	273.26	413.81	176.21	344.74	499.66	222.32	9.51	16.02	4.67
72	New Zealand	Rate	89.53	160.64	42.51	223.53	299.60	163.20	69.34	98.51	44.39	95.65	144.84	61.68	120.67	174.89	77.82	3.33	5.61	1.64
74	Andorra	Number	2.54	4.84	1.24	5.44	7.35	3.88	2.25	3.33	1.38	2.59	4.12	1.60	3.09	4.57	1.93	0.06	0.11	0.02
74	Andorra	Rate	83.05	158.32	40.53	178.20	240.43	127.14	73.73	108.95	45.07	84.78	134.80	52.49	101.03	149.66	63.11	1.95	3.65	0.81
75	Austria	Number	349.32	629.05	163.28	791.74	1,057.52	566.15	309.24	462.94	190.45	338.03	531.31	209.86	399.74	577.38	254.51	7.81	14.75	3.14
75	Austria	Rate	86.91	156.50	40.62	196.98	263.10	140.85	76.93	115.17	47.38	84.10	132.18	52.21	99.45	143.64	63.32	1.94	3.67	0.78
76	Belgium	Number	569.25	1,112.18	246.29	916.28	1,251.13	636.62	528.05	794.20	312.51	535.49	836.87	330.06	625.00	906.28	389.56	12.09	22.18	5.10
76	Belgium	Rate	90.07	175.99	38.97	144.99	197.97	100.73	83.56	125.67	49.45	84.73	132.42	52.23	98.90	143.41	61.64	1.91	3.51	0.81
77	Cyprus	Number	49.03	84.43	24.97	96.82	129.82	68.63	42.51	62.52	25.24	44.71	71.03	27.45	49.43	71.63	31.22	0.96	1.81	0.40
77	Cyprus	Rate	98.80	170.13	50.32	195.09	261.59	138.30	85.67	125.99	50.86	90.09	143.13	55.31	99.59	144.34	62.91	1.94	3.65	0.80
78	Denmark	Number	231.61	404.01	122.02	578.05	764.74	414.19	133.02	193.61	85.97	206.16	308.23	132.84	302.67	447.07	188.89	3.51	6.76	1.47
78	Denmark	Rate	80.31	140.09	42.31	200.43	265.17	143.62	46.12	67.13	29.81	71.49	106.88	46.06	104.95	155.02	65.50	1.22	2.34	0.51
79	Finland	Number	221.06	388.34	114.76	697.39	921.32	500.91	174.45	248.73	112.85	234.96	372.57	145.73	290.71	423.20	191.21	8.49	14.37	4.29
79	Finland	Rate	76.11	133.70	39.51	240.10	317.20	172.46	60.06	85.63	38.85	80.89	128.27	50.17	100.09	145.70	65.83	2.92	4.95	1.48
80	France	Number	4,908.64	9,286.02	2,011.43	6,495.54	9,164.48	4,574.52	3,158.87	4,617.55	2,014.14	3,131.19	5,060.50	1,946.13	3,596.44	5,319.40	2,295.27	78.02	134.96	36.21
80	France	Rate	125.25	236.95	51.32	165.74	233.85	116.73	80.60	117.82	51.39	79.90	129.13	49.66	91.77	135.73	58.57	1.99	3.44	0.92
81	Germany	Number	5,636.51	10,755.39	2,513.08	6,491.17	8,918.96	4,530.68	4,352.13	6,166.11	2,846.28	3,601.94	5,732.77	2,238.93	3,420.13	4,953.17	2,155.45	47.53	89.74	20.39
81	Germany	Rate	164.35	313.61	73.28	189.27	260.06	132.11	126.90	179.79	82.99	105.03	167.16	65.28	99.72	144.42	62.85	1.39	2.62	0.59
82	Greece	Number	419.34	773.15	205.33	846.69	1,150.65	591.13	440.77	666.06	260.77	344.56	519.30	222.03	474.82	702.36	295.33	9.16	17.80	3.74
82	Greece	Rate	88.74	163.61	43.45	179.17	243.50	125.09	93.27	140.95	55.18	72.92	109.89	46.99	100.48	148.63	62.50	1.94	3.77	0.79
83	Iceland	Number	21.35	38.17	10.80	43.00	58.44	30.51	16.80	25.62	9.97	18.14	29.13	11.44	19.70	28.88	12.27	0.45	0.79	0.21
83	Iceland	Rate	99.52	177.90	50.33	200.39	272.37	142.21	78.30	119.38	46.45	84.55	135.76	53.33	91.79	134.61	57.20	2.08	3.67	1.00
84	Ireland	Number	279.15	525.12	128.10	669.34	896.03	469.28	267.51	400.45	162.39	291.77	461.83	178.30	357.76	526.93	230.34	7.61	13.52	3.58
84	Ireland	Rate	82.99	156.11	38.08	198.99	266.38	139.51	79.53	119.05	48.28	86.74	137.30	53.01	106.36	156.65	68.48	2.26	4.02	1.07
85	Israel	Number	747.06	1,286.57	370.94	1,630.31	2,211.79	1,134.44	735.10	1,122.16	441.81	1,281.31	1,887.04	864.31	833.01	1,223.84	533.96	18.52	32.25	8.52
85	Israel	Rate	89.17	153.57	44.28	194.61	264.01	135.41	87.75	133.95	52.74	152.95	225.25	103.17	99.43	146.09	63.74	2.21	3.85	1.02
86	Italy	Number	2,083.81	3,814.47	1,034.66	5,187.82	6,993.90	3,689.63	2,295.72	3,518.99	1,373.35	2,206.16	3,261.47	1,454.66	2,552.61	3,767.57	1,601.15	58.69	103.31	26.73
86	Italy	Rate	82.01	150.12	40.72	204.17	275.24	145.20	90.35	138.49	54.05	86.82	128.35	57.25	100.46	148.27	63.01	2.31	4.07	1.05
87	Luxembourg	Number	25.01	46.74	12.19	57.05	77.31	40.89	21.19	32.07	12.49	25.63	40.02	15.76	29.85	43.10	18.87	0.58	1.11	0.25
87	Luxembourg	Rate	82.79	154.71	40.35	188.84	255.93	135.36	70.14	106.16	41.34	84.86	132.48	52.16	98.82	142.68	62.45	1.92	3.66	0.81
88	Malta	Number	18.35	32.39	9.52	39.08	52.24	28.37	16.42	24.74	9.92	17.22	26.95	10.76	19.10	27.81	11.82	0.37	0.70	0.15
88	Malta	Rate	96.19	169.80	49.91	204.84	273.86	148.72	86.10	129.68	52.01	90.28	141.25	56.43	100.14	145.79	61.98	1.94	3.68	0.76
89	Netherlands	Number	934.30	1,665.46	434.07	1,590.29	2,192.96	1,111.57	671.71	1,006.43	408.10	747.25	1,240.20	453.29	863.65	1,272.69	542.31	17.74	32.20	8.28
89	Netherlands	Rate	107.41	191.47	49.90	182.83	252.11	127.79	77.22	115.70	46.92	85.91	142.58	52.11	99.29	146.31	62.35	2.04	3.70	0.95
90	Norway	Number	273.58	485.42	140.03	569.99	762.69	416.96	177.78	255.41	114.60	222.76	341.63	143.35	279.36	406.43	177.16	5.01	8.90	2.14
90	Norway	Rate	92.07	163.36	47.12	191.81	256.66	140.32	59.83	85.95	38.57	74.96	114.97	48.24	94.01	136.77	59.62	1.69	3.00	0.72
91	Portugal	Number	283.78	541.79	122.65	605.35	813.78	423.13	384.81	571.11	229.79	336.74	522.72	211.26	421.44	615.07	261.70	8.37	15.96	3.60
91	Portugal	Rate	66.98	127.87	28.95	142.87	192.07	99.86	90.82	134.79	54.23	79.47	123.37	49.86	99.47	145.17	61.76	1.98	3.77	0.85
92	Spain	Number	1,931.61	3,495.50	921.89	3,994.77	5,631.85	2,751.00	1,950.26	2,875.10	1,205.57	2,134.02	3,385.41	1,333.03	2,187.79	3,211.07	1,396.97	64.14	112.32	30.21
92	Spain	Rate	89.19	161.41	42.57	184.46	260.06	127.03	90.06	132.76	55.67	98.54	156.33	61.55	101.02	148.27	64.51	2.96	5.19	1.39
93	Sweden	Number	469.57	817.64	261.63	1,264.86	1,699.18	912.61	405.08	585.16	262.04	395.06	657.32	243.99	710.38	1,029.60	456.84	10.37	17.01	5.19
93	Sweden	Rate	82.10	142.96	45.74	221.15	297.09	159.56	70.83	102.31	45.82	69.07	114.93	42.66	124.21	180.02	79.88	1.81	2.97	0.91
94	Switzerland	Number	461.25	760.99	257.66	805.20	1,097.33	574.32	306.52	449.51	183.15	414.22	654.51	252.04	415.12	602.18	262.44	7.46	13.27	3.60
94	Switzerland	Rate	110.68	182.61	61.83	193.22	263.32	137.81	73.55	107.87	43.95	99.40	157.06	60.48	99.61	144.50	62.98	1.79	3.19	0.86
95	United Kingdom	Number	4,042.00	5,796.30	2,803.77	6,712.26	9,048.00	4,859.87	3,663.53	5,069.82	2,458.41	3,485.67	5,141.34	2,312.12	4,086.11	5,971.06	2,608.23	80.44	127.28	48.03
95	United Kingdom	Rate	103.52	148.44	71.80	171.90	231.72	124.46	93.82	129.84	62.96	89.27	131.67	59.21	104.65	152.92	66.80	2.06	3.26	1.23

Table S3. Global and country-specific YLDs of developmental disabilities in 2016 (with 95% uncertainty interval)																				
			Epilepsy			Intellectual disability			Hearing loss			Vision loss			Autism spectrum disorder			Attention-deficit/hyperactivity disorder		
Loc-ID	Location	Metric	Value	Upper limit	Lower limit	Value	Upper limit	Lower limit	Value	Upper limit	Lower limit	Value	Upper limit	Lower limit	Value	Upper limit	Lower limit	Value	Upper limit	Lower limit
97	Argentina	Number	3,764.95	6,828.71	1,725.35	5,399.90	7,502.84	3,793.71	3,133.93	4,527.57	2,033.16	3,595.33	5,404.91	2,356.09	3,899.12	5,622.33	2,428.61	102.44	184.36	52.50
97	Argentina	Rate	105.12	190.66	48.17	150.77	209.48	105.92	87.50	126.41	56.77	100.38	150.91	65.78	108.86	156.98	67.81	2.86	5.15	1.47
98	Chile	Number	1,398.08	2,782.48	561.35	1,730.01	2,363.79	1,243.58	1,044.27	1,485.94	672.46	1,206.93	1,792.61	772.72	1,325.47	1,937.01	851.00	34.62	58.69	16.63
98	Chile	Rate	116.11	231.08	46.62	143.68	196.31	103.28	86.73	123.41	55.85	100.24	148.88	64.17	110.08	160.87	70.67	2.88	4.87	1.38
99	Uruguay	Number	287.13	539.21	123.40	362.56	492.99	254.24	211.98	302.93	140.12	243.02	362.68	156.89	259.36	379.37	161.79	6.85	11.85	3.36
99	Uruguay	Rate	120.85	226.96	51.94	152.60	207.50	107.01	89.22	127.50	58.97	102.29	152.65	66.03	109.17	159.68	68.10	2.88	4.99	1.41
101	Canada	Number	1,397.74	2,584.90	688.32	3,040.91	4,140.62	2,153.92	1,502.71	2,168.77	996.98	1,070.13	1,600.96	679.20	2,327.43	3,429.26	1,492.88	53.48	90.51	27.22
101	Canada	Rate	72.06	133.26	35.49	156.77	213.46	111.04	77.47	111.81	51.40	55.17	82.54	35.02	119.99	176.79	76.96	2.76	4.67	1.40
102	United States	Number	20,599.24	28,493.71	14,324.87	36,228.50	48,173.70	26,236.01	15,151.96	21,071.92	10,221.83	12,519.44	18,989.28	8,303.52	21,756.93	31,740.27	13,888.53	519.32	828.63	312.30
102	United States	Rate	104.76	144.91	72.85	184.24	244.99	133.43	77.06	107.16	51.98	63.67	96.57	42.23	110.65	161.42	70.63	2.64	4.21	1.59
105	Antigua and Barbuda	Number	12.52	21.25	6.48	13.53	25.21	8.01	6.94	9.94	4.53	9.49	16.07	5.68	6.47	9.37	4.15	0.22	0.37	0.12
105	Antigua and Barbuda	Rate	205.13	348.30	106.19	221.81	413.26	131.26	113.69	162.98	74.31	155.49	263.32	93.16	105.97	153.54	67.97	3.54	6.01	1.89
106	The Bahamas	Number	81.56	145.34	43.55	82.64	144.63	50.12	36.97	52.57	24.23	67.20	125.65	38.01	35.36	51.35	22.07	1.16	1.92	0.62
106	The Bahamas	Rate	247.13	440.39	131.95	250.40	438.24	151.87	112.01	159.30	73.41	203.63	380.71	115.18	107.16	155.60	66.89	3.51	5.83	1.87
107	Barbados	Number	48.75	92.37	25.12	49.17	93.44	28.10	17.11	24.30	11.00	38.55	79.24	20.32	15.78	23.49	10.27	0.53	0.90	0.27
107	Barbados	Rate	329.63	624.59	169.83	332.52	631.85	190.02	115.70	164.29	74.40	260.69	535.81	137.44	106.68	158.83	69.44	3.57	6.09	1.86
108	Belize	Number	139.13	259.66	72.58	144.36	261.78	85.57	67.06	93.89	43.77	118.84	219.79	64.67	50.24	73.79	31.60	1.67	2.76	0.87
108	Belize	Rate	292.86	546.55	152.78	303.86	551.02	180.12	141.16	197.63	92.13	250.15	462.64	136.13	105.75	155.32	66.52	3.51	5.81	1.83
109	Cuba	Number	993.75	1,684.01	515.63	1,069.23	1,672.97	707.52	722.41	1,018.11	479.69	918.10	1,469.33	569.50	648.15	931.54	416.46	21.71	36.72	11.42
109	Cuba	Rate	165.76	280.89	86.01	178.35	279.05	118.02	120.50	169.82	80.01	153.14	245.09	94.99	108.11	155.38	69.47	3.62	6.13	1.90
110	Dominica	Number	20.24	41.31	9.36	21.77	44.21	11.15	6.68	9.48	4.50	16.76	36.74	7.83	5.71	8.30	3.62	0.19	0.32	0.10
110	Dominica	Rate	380.49	776.61	176.02	409.22	831.13	209.67	125.65	178.28	84.64	315.13	690.77	147.26	107.28	156.06	68.10	3.52	5.93	1.83
111	Dominican Republic	Number	4,173.10	8,237.37	1,969.44	4,407.43	8,355.00	2,382.64	1,213.53	1,707.71	803.34	3,830.26	7,741.23	1,887.25	1,002.12	1,462.12	629.19	33.67	58.90	17.62
111	Dominican Republic	Rate	443.38	875.21	209.25	468.28	887.70	253.15	128.93	181.44	85.35	406.96	822.49	200.52	106.47	155.35	66.85	3.58	6.26	1.87
112	Grenada	Number	26.73	46.74	13.99	29.32	53.45	16.76	11.21	16.08	7.43	21.17	40.22	11.71	9.34	13.40	6.06	0.31	0.54	0.16
112	Grenada	Rate	307.31	537.29	160.86	337.01	614.47	192.68	128.91	184.85	85.40	243.32	462.32	134.59	107.41	154.09	69.70	3.53	6.19	1.84
113	Guyana	Number	254.32	494.26	129.93	253.36	484.29	147.03	92.72	131.85	62.60	212.13	430.78	113.92	69.33	100.19	44.31	2.30	3.98	1.23
113	Guyana	Rate	389.22	756.44	198.85	387.76	741.17	225.01	141.90	201.79	95.80	324.65	659.29	174.35	106.10	153.34	67.81	3.51	6.09	1.88
114	Haiti	Number	3,875.87	8,089.88	1,762.72	4,920.98	9,205.82	2,969.62	2,870.17	4,036.90	1,902.18	3,709.74	7,455.15	1,890.72	1,577.57	2,303.22	992.75	53.01	93.30	27.52
114	Haiti	Rate	255.10	532.45	116.02	323.88	605.90	195.45	188.90	265.70	125.19	244.16	490.67	124.44	103.83	151.59	65.34	3.49	6.14	1.81
115	Jamaica	Number	894.35	1,620.61	463.29	887.77	1,601.00	520.98	365.43	503.77	238.68	738.24	1,405.46	401.79	294.82	421.59	190.42	9.71	16.54	5.08
115	Jamaica	Rate	323.26	585.76	167.45	320.88	578.67	188.30	132.08	182.08	86.27	266.83	508.00	145.23	106.56	152.38	68.83	3.51	5.98	1.84
116	Saint Lucia	Number	30.14	54.21	15.70	31.19	58.13	17.33	12.39	17.39	8.11	24.66	46.83	13.66	10.25	15.16	6.51	0.34	0.57	0.18
116	Saint Lucia	Rate	312.90	562.75	162.99	323.81	603.41	179.89	128.65	180.53	84.14	256.02	486.19	141.80	106.42	157.34	67.53	3.54	5.88	1.84
117	Saint Vincent and the Grenadines	Number	30.39	57.59	15.65	31.24	59.89	17.86	12.31	17.59	8.06	25.24	47.61	13.61	9.67	13.99	6.11	0.32	0.54	0.17
117	Saint Vincent and the Grenadines	Rate	332.38	629.91	171.14	341.66	655.14	195.38	134.70	192.36	88.21	276.07	520.73	148.85	105.82	152.98	66.84	3.53	5.89	1.85
118	Suriname	Number	184.49	368.52	83.76	191.41	387.65	99.37	58.92	82.55	39.43	157.52	341.76	73.84	49.49	72.81	30.77	1.65	2.82	0.81
118	Suriname	Rate	397.12	793.23	180.30	412.01	834.42	213.89	126.83	177.69	84.86	339.06	735.64	158.95	106.52	156.73	66.22	3.55	6.06	1.75
119	Trinidad and Tobago	Number	176.14	305.65	92.86	168.85	299.27	103.02	77.83	111.18	50.92	136.29	251.52	76.40	74.03	107.83	45.59	2.46	4.16	1.35
119	Trinidad and Tobago	Rate	255.69	443.70	134.80	245.11	434.43	149.54	112.98	161.39	73.92	197.84	365.11	110.91	107.46	156.53	66.18	3.57	6.04	1.96
121	Bolivia	Number	2,573.92	4,822.24	1,223.72	3,188.39	5,491.62	1,939.37	1,914.96	2,688.61	1,269.31	3,031.82	5,229.30	1,761.86	1,397.32	2,044.62	889.29	26.75	46.87	12.74
121	Bolivia	Rate	188.07	352.35	89.41	232.97	401.26	141.70	139.92	196.45	92.75	221.53	382.09	128.73	102.10	149.40	64.98	1.95	3.42	0.93
122	Ecuador	Number	3,028.21	5,417.29	1,358.39	2,882.94	4,135.12	2,020.69	2,181.76	3,062.83	1,470.99	2,720.52	4,054.96	1,800.63	1,874.88	2,711.57	1,183.36	35.93	62.57	16.97
122	Ecuador	Rate	169.32	302.90	75.95	161.19	231.21	112.98	121.99	171.25	82.25	152.11	226.72	100.68	104.83	151.61	66.16	2.01	3.50	0.95
123	Peru	Number	8,926.04	16,029.01	4,300.79	9,441.13	16,201.59	5,694.74	4,550.86	6,331.20	3,029.01	8,774.11	15,081.48	5,243.87	3,633.16	5,375.19	2,297.94	69.73	124.91	32.30
123	Peru	Rate	254.62	457.23	122.68	269.31	462.16	162.45	129.82	180.60	86.40	250.29	430.21	149.58	103.64	153.33	65.55	1.99	3.56	0.92
125	Colombia	Number	5,744.44	11,090.51	2,250.66	5,609.14	7,720.88	3,958.44	4,692.56	6,658.18	3,117.20	4,947.34	7,369.62	3,219.88	3,876.11	5,718.72	2,512.14	57.87	102.04	25.81
125	Colombia	Rate	160.76	310.37	62.98	156.97	216.07	110.78	131.32	186.33	87.24	138.45	206.24	90.11	108.47	160.04	70.30	1.62	2.86	0.72
126	Costa Rica	Number	404.46	825.10	157.12	395.65	538.82	274.82	387.83	547.93	256.20	353.14	525.56	232.28	331.74	485.23	210.00	4.76	8.63	1.98
126	Costa Rica	Rate	132.96	271.23	51.65	130.06	177.13	90.34	127.49	180.12	84.22	116.09	172.77	76.36	109.05	159.51	69.03	1.57	2.84	0.65
127	El Salvador	Number	804.78	1,532.39	323.23	869.32	1,180.01	619.51	764.08	1,090.95	494.65	720.56	1,083.45	473.61	577.88	846.25	371.84	8.47	15.94	3.70
127	El Salvador	Rate	150.73	287.00	60.54	162.82	221.01	116.03	143.11	204.33	92.64	134.96	202.92	88.70	108.23	158.50	69.64	1.59	2.99	0.69
128	Guatemala	Number	2,738.25	5,225.63	1,183.79	3,390.43	4,687.13	2,424.37	3,048.87	4,328.47	2,026.70	2,701.20	3,965.03	1,755.13	2,096.63	3,064.25	1,348.41	30.55	57.27	13.22
128	Guatemala	Rate	138.88	265.03	60.04	171.95	237.72	122.96	154.63	219.53	102.79	137.00	201.10	89.02	106.34	155.41	68.39	1.55	2.90	0.67

Table S3. Global and country-specific YLDs of developmental disabilities in 2016 (with 95% uncertainty interval)																				
			Epilepsy			Intellectual disability			Hearing loss			Vision loss			Autism spectrum disorder			Attention-deficit/hyperactivity disorder		
Loc-ID	Location	Metric	Value	Upper limit	Lower limit	Value	Upper limit	Lower limit	Value	Upper limit	Lower limit	Value	Upper limit	Lower limit	Value	Upper limit	Lower limit	Value	Upper limit	Lower limit
129	Honduras	Number	1,546.21	2,835.95	643.63	1,595.15	2,177.88	1,137.46	1,493.48	2,122.45	981.53	1,261.33	1,867.99	806.49	1,022.45	1,469.91	637.47	14.78	26.96	6.29
129	Honduras	Rate	163.25	299.43	67.96	168.42	229.95	120.10	157.69	224.10	103.63	133.18	197.23	85.15	107.95	155.20	67.31	1.56	2.85	0.66
130	Mexico	Number	20,509.60	29,518.19	13,281.22	18,848.15	25,607.96	13,591.59	15,104.77	21,249.87	10,096.38	14,896.69	22,125.47	9,941.36	12,806.18	18,568.23	8,134.04	136.76	220.94	79.20
130	Mexico	Rate	175.72	252.91	113.79	161.49	219.41	116.45	129.42	182.07	86.51	127.63	189.57	85.18	109.72	159.09	69.69	1.17	1.89	0.68
131	Nicaragua	Number	901.46	1,706.41	376.29	1,041.70	1,436.43	731.94	950.70	1,358.54	622.35	846.08	1,252.85	543.58	656.23	942.93	414.45	9.70	18.08	4.09
131	Nicaragua	Rate	147.99	280.13	61.77	171.01	235.81	120.16	156.07	223.02	102.17	138.90	205.67	89.24	107.73	154.80	68.04	1.59	2.97	0.67
132	Panama	Number	448.09	844.80	189.03	527.37	726.76	371.21	420.94	594.54	278.95	464.25	688.02	300.50	376.39	550.25	239.57	5.43	10.35	2.35
132	Panama	Rate	129.23	243.65	54.52	152.10	209.61	107.06	121.40	171.47	80.45	133.89	198.43	86.67	108.55	158.70	69.10	1.57	2.98	0.68
133	Venezuela	Number	4,642.82	9,071.96	2,007.74	4,547.81	6,301.36	3,174.14	3,593.31	4,996.73	2,380.36	3,662.16	5,410.96	2,406.23	3,130.15	4,549.03	1,999.77	58.02	103.66	27.20
133	Venezuela	Rate	160.86	314.31	69.56	157.57	218.32	109.97	124.50	173.12	82.47	126.88	187.47	83.37	108.45	157.61	69.29	2.01	3.59	0.94
135	Brazil	Number	23,621.48	33,819.27	16,264.71	33,361.03	45,703.72	24,668.41	20,182.46	28,058.05	13,938.46	22,477.41	33,857.65	14,452.83	15,865.72	23,203.51	10,243.43	256.29	405.34	150.31
135	Brazil	Rate	152.61	218.50	105.08	215.54	295.28	159.38	130.39	181.28	90.05	145.22	218.75	93.38	102.50	149.91	66.18	1.66	2.62	0.97
136	Paraguay	Number	992.24	1,741.97	499.23	1,179.23	1,757.53	817.06	878.75	1,238.36	578.41	1,080.00	1,673.53	688.72	641.92	952.81	405.96	10.11	18.23	4.43
136	Paraguay	Rate	157.31	276.17	79.15	186.96	278.64	129.54	139.32	196.33	91.70	171.22	265.32	109.19	101.77	151.06	64.36	1.60	2.89	0.70
139	Algeria	Number	14,057.84	27,872.30	6,908.19	15,222.63	28,151.24	9,737.98	6,252.76	8,946.36	3,960.06	11,718.82	23,621.15	6,596.38	4,943.10	7,287.00	3,157.98	83.99	154.94	36.81
139	Algeria	Rate	308.99	612.63	151.84	334.59	618.76	214.04	137.44	196.64	87.04	257.58	519.19	144.99	108.65	160.17	69.41	1.85	3.41	0.81
140	Bahrain	Number	221.47	392.98	98.28	222.71	368.93	148.82	114.44	166.56	72.71	189.82	330.89	117.89	109.22	158.19	69.40	1.83	3.35	0.83
140	Bahrain	Rate	222.14	394.17	98.58	223.38	370.05	149.27	114.78	167.07	72.93	190.40	331.89	118.24	109.55	158.66	69.61	1.84	3.36	0.84
141	Egypt	Number	37,247.50	72,369.74	17,393.38	39,314.68	75,002.33	24,951.41	15,610.20	22,321.15	10,049.70	32,013.06	64,477.65	19,092.97	11,763.43	17,271.34	7,685.53	270.02	468.29	138.33
141	Egypt	Rate	341.52	663.55	159.48	360.47	687.68	228.78	143.13	204.66	92.14	293.52	591.19	175.06	107.86	158.36	70.47	2.48	4.29	1.27
142	Iran	Number	22,042.80	41,125.00	10,267.63	22,048.10	38,447.40	14,772.50	10,092.13	14,610.95	6,487.58	13,816.37	27,733.98	8,229.84	8,978.20	13,270.48	5,892.84	222.16	391.64	112.17
142	Iran	Rate	273.32	509.94	127.32	273.39	476.74	183.18	125.14	181.17	80.44	171.32	343.89	102.05	111.33	164.55	73.07	2.75	4.86	1.39
143	Iraq	Number	33,937.71	84,579.73	17,183.85	35,937.34	84,106.35	20,357.54	12,521.72	18,148.53	7,901.75	30,261.67	75,744.29	15,200.63	8,177.14	11,836.82	5,315.16	79.37	148.18	29.94
143	Iraq	Rate	440.46	1,097.72	223.02	466.42	1,091.58	264.21	162.51	235.54	102.55	392.75	983.05	197.28	106.13	153.63	68.98	1.03	1.92	0.39
144	Jordan	Number	2,093.32	3,537.58	1,060.98	2,524.31	3,485.68	1,773.13	1,325.76	1,912.45	849.44	1,800.83	2,725.45	1,139.88	1,049.80	1,541.39	675.83	17.70	31.51	7.97
144	Jordan	Rate	218.00	368.40	110.49	262.88	362.99	184.65	138.06	199.16	88.46	187.54	283.82	118.71	109.32	160.52	70.38	1.84	3.28	0.83
145	Kuwait	Number	503.61	929.99	217.15	576.76	833.43	398.54	281.88	408.21	181.21	443.94	689.41	280.42	311.89	460.81	202.67	5.18	9.37	2.23
145	Kuwait	Rate	178.69	329.97	77.05	204.64	295.71	141.41	100.01	144.84	64.30	157.52	244.61	99.50	110.66	163.50	71.91	1.84	3.33	0.79
146	Lebanon	Number	772.86	1,541.11	352.63	980.91	1,725.44	639.63	386.90	557.42	242.31	623.14	1,278.65	353.63	351.21	505.21	224.73	7.07	12.34	3.50
146	Lebanon	Rate	240.91	480.38	109.92	305.76	537.84	199.38	120.60	173.75	75.53	194.24	398.57	110.23	109.48	157.48	70.05	2.20	3.85	1.09
147	Libya	Number	1,138.70	2,317.07	541.59	1,410.31	2,539.49	896.17	569.15	837.30	358.88	1,159.91	2,208.74	719.49	509.11	737.77	321.99	8.69	15.37	3.92
147	Libya	Rate	245.95	500.47	116.98	304.62	548.51	193.56	122.93	180.85	77.52	250.53	477.07	155.40	109.96	159.35	69.55	1.88	3.32	0.85
148	Morocco	Number	7,157.31	14,759.13	3,358.64	8,244.83	15,529.03	5,266.03	3,435.04	4,970.86	2,195.23	5,453.82	11,789.00	2,995.12	2,476.50	3,650.23	1,586.51	42.11	76.92	18.96
148	Morocco	Rate	312.36	644.12	146.58	359.82	677.72	229.82	149.91	216.94	95.80	238.01	514.49	130.71	108.08	159.30	69.24	1.84	3.36	0.83
149	Palestine	Number	3,936.28	8,636.09	1,862.15	4,605.68	9,373.44	2,734.35	1,986.54	2,869.94	1,249.29	3,447.54	7,726.21	1,794.68	1,178.58	1,705.98	768.81	19.62	36.88	8.46
149	Palestine	Rate	362.65	795.65	171.56	424.32	863.58	251.92	183.02	264.41	115.10	317.62	711.82	165.34	108.58	157.17	70.83	1.81	3.40	0.78
150	Oman	Number	691.64	1,351.15	239.38	819.64	1,099.34	586.94	291.00	414.70	192.16	720.95	1,054.30	466.97	481.44	702.27	305.32	8.02	14.56	3.69
150	Oman	Rate	156.00	304.74	53.99	184.86	247.95	132.38	65.63	93.53	43.34	162.61	237.79	105.32	108.59	158.39	68.86	1.81	3.28	0.83
151	Qatar	Number	213.54	385.05	99.65	272.75	373.14	194.86	116.48	170.81	74.75	184.40	270.13	124.73	136.05	200.36	87.37	2.23	4.02	1.01
151	Qatar	Rate	172.48	311.00	80.49	220.30	301.39	157.39	94.08	137.97	60.37	148.94	218.19	100.74	109.89	161.83	70.57	1.80	3.25	0.82
152	Saudi Arabia	Number	7,685.14	14,575.47	4,329.93	8,505.52	15,666.13	5,260.07	2,791.14	3,905.36	1,884.66	6,910.33	13,492.63	3,989.76	2,755.90	3,945.16	1,775.83	46.43	75.79	26.18
152	Saudi Arabia	Rate	308.38	584.87	173.75	341.30	628.64	211.07	112.00	156.71	75.63	277.29	541.42	160.10	110.59	158.31	71.26	1.86	3.04	1.05
153	Syria	Number	4,154.46	8,120.23	1,895.38	4,990.16	8,773.71	3,351.30	2,705.23	3,813.46	1,764.90	3,580.89	7,073.17	2,152.49	1,922.93	2,781.91	1,212.19	33.34	59.62	15.18
153	Syria	Rate	235.11	459.55	107.27	282.41	496.53	189.66	153.10	215.82	99.88	202.65	400.29	121.82	108.82	157.44	68.60	1.89	3.37	0.86
154	Tunisia	Number	2,403.10	5,120.41	1,179.71	2,736.75	5,157.31	1,713.31	1,150.01	1,656.93	734.71	1,840.38	4,121.73	993.53	909.49	1,342.23	580.40	20.77	37.50	10.26
154	Tunisia	Rate	287.99	613.63	141.38	327.98	618.06	205.32	137.82	198.57	88.05	220.55	493.95	119.07	108.99	160.85	69.56	2.49	4.49	1.23
155	Turkey	Number	11,621.63	21,937.64	4,599.81	11,415.53	15,121.32	8,299.30	7,613.74	11,000.45	4,787.89	5,653.84	8,575.59	3,694.65	6,670.91	9,667.49	4,297.42	184.92	320.95	96.39
155	Turkey	Rate	188.71	356.21	74.69	185.36	245.53	134.76	123.63	178.62	77.74	91.80	139.25	59.99	108.32	156.98	69.78	3.00	5.21	1.57
156	United Arab Emirates	Number	1,813.81	3,326.71	875.62	1,820.91	2,876.05	1,255.44	864.17	1,254.88	558.56	1,557.59	2,630.25	981.45	891.94	1,292.34	564.09	8.73	16.61	3.43
156	United Arab Emirates	Rate	222.43	407.95	107.38	223.30	352.69	153.95	105.97	153.88	68.50	191.01	322.54	120.35	109.38	158.48	69.17	1.07	2.04	0.42
157	Yemen	Number	11,346.33	20,729.52	5,560.83	16,220.82	25,969.55	11,082.86	16,746.54	23,114.32	11,325.17	10,867.79	19,288.01	6,698.74	4,855.94	7,131.82	3,140.35	57.87	111.10	24.35
157	Yemen	Rate	245.23	448.04	120.19	350.59	561.29	239.54	361.95	499.58	244.78	234.89	416.88	144.78	104.95	154.14	67.87	1.25	2.40	0.53
160	Afghanistan	Number	13,794.41	29,471.14	6,042.25	21,368.18	37,043.49	14,231.23	11,305.20	16,086.67	7,349.57	13,787.32	27,511.77	8,068.97	5,254.92	7,618.94	3,346.91	88.11	168.36	38.29
160	Afghanistan	Rate	278.27	594.50	121.89	431.05	747.25	287.08	228.05	324.51	148.26	278.12	554.98	162.77	106.00	153.69	67.52	1.78	3.40	0.77

Table S3. Global and country-specific YLDs of developmental disabilities in 2016 (with 95% uncertainty interval)																				
			Epilepsy			Intellectual disability			Hearing loss			Vision loss			Autism spectrum disorder			Attention-deficit/hyperactivity disorder		
Loc-ID	Location	Metric	Value	Upper limit	Lower limit	Value	Upper limit	Lower limit	Value	Upper limit	Lower limit	Value	Upper limit	Lower limit	Value	Upper limit	Lower limit	Value	Upper limit	Lower limit
161	Bangladesh	Number	30,589.80	49,054.23	18,325.21	33,970.30	52,491.59	23,107.58	48,964.36	67,763.40	32,536.60	24,850.70	39,885.08	15,773.62	14,445.34	21,217.91	8,994.17	112.75	222.93	37.47
161	Bangladesh	Rate	213.48	342.33	127.89	237.07	366.32	161.26	341.71	472.90	227.06	173.42	278.34	110.08	100.81	148.07	62.77	0.79	1.56	0.26
162	Bhutan	Number	177.02	288.60	102.33	192.30	291.93	132.33	155.09	220.73	99.93	112.59	201.98	69.04	78.02	113.81	49.51	0.58	1.17	0.15
162	Bhutan	Rate	226.75	369.68	131.08	246.32	373.94	169.51	198.66	282.74	128.01	144.23	258.73	88.43	99.94	145.78	63.42	0.74	1.50	0.19
163	India	Number	306,008.29	439,166.06	217,777.39	395,583.05	544,194.07	286,635.52	232,015.36	325,299.93	159,169.76	233,730.90	360,515.41	154,052.91	114,372.85	165,266.55	73,029.39	802.50	1,273.98	463.09
163	India	Rate	272.88	391.62	194.20	352.76	485.28	255.60	206.90	290.08	141.94	208.43	321.49	137.37	101.99	147.37	65.12	0.72	1.14	0.41
164	Nepal	Number	9,912.00	15,664.51	5,872.70	10,996.00	15,840.18	7,722.89	7,634.99	10,797.08	5,016.45	6,661.43	10,791.11	4,290.50	4,026.14	5,970.57	2,555.94	30.32	61.29	9.25
164	Nepal	Rate	248.37	392.51	147.15	275.53	396.91	193.51	191.31	270.54	125.70	166.92	270.40	107.51	100.88	149.61	64.04	0.76	1.54	0.23
165	Pakistan	Number	55,683.90	88,897.38	32,923.04	61,849.74	88,277.19	43,184.02	46,149.97	65,740.53	30,143.81	36,585.77	57,135.08	23,367.00	23,306.90	34,044.83	14,876.98	172.88	345.56	57.90
165	Pakistan	Rate	241.36	385.32	142.70	268.08	382.63	187.18	200.03	284.95	130.66	158.58	247.65	101.28	101.02	147.56	64.48	0.75	1.50	0.25
168	Angola	Number	13,941.04	24,778.58	7,145.04	15,653.78	26,626.24	9,987.10	12,627.86	17,454.76	8,312.99	11,818.25	21,506.77	6,579.85	4,800.61	7,070.76	3,033.61	106.38	182.11	51.42
168	Angola	Rate	284.15	505.04	145.63	319.05	542.70	203.56	257.38	355.76	169.44	240.88	438.35	134.11	97.85	144.12	61.83	2.17	3.71	1.05
169	Central African Republic	Number	1,705.74	3,064.38	933.92	2,658.79	3,937.21	1,876.33	2,421.39	3,426.76	1,606.81	1,641.58	2,796.92	973.26	726.95	1,064.44	461.38	15.86	27.50	7.73
169	Central African Republic	Rate	229.87	412.96	125.86	358.31	530.59	252.86	326.31	461.80	216.54	221.22	376.92	131.16	97.97	143.45	62.18	2.14	3.71	1.04
170	Congo	Number	2,382.76	4,405.25	1,267.18	2,562.27	4,470.19	1,607.45	1,757.41	2,437.97	1,177.49	1,889.84	3,622.51	974.45	739.74	1,089.04	474.89	16.25	29.25	7.69
170	Congo	Rate	319.76	591.18	170.05	343.85	599.89	215.72	235.84	327.17	158.02	253.61	486.14	130.77	99.27	146.15	63.73	2.18	3.93	1.03
171	Democratic Republic of the Congo	Number	36,595.19	65,331.87	18,967.66	50,110.05	77,910.43	33,511.22	45,932.51	63,067.28	31,078.33	33,097.79	57,621.58	19,184.46	13,638.34	19,795.59	8,718.44	298.68	541.09	147.41
171	Democratic Republic of the Congo	Rate	260.33	464.76	134.93	356.47	554.24	238.39	326.75	448.65	221.08	235.45	409.91	136.47	97.02	140.82	62.02	2.12	3.85	1.05
172	Equatorial Guinea	Number	259.27	469.89	132.20	273.71	478.90	164.94	171.69	240.71	114.05	207.65	396.65	109.30	97.35	141.70	61.80	2.14	3.86	1.02
172	Equatorial Guinea	Rate	261.54	474.01	133.36	276.11	483.10	166.39	173.20	242.82	115.05	209.47	400.13	110.26	98.20	142.95	62.34	2.15	3.89	1.03
173	Gabon	Number	949.01	1,818.07	488.72	929.87	1,797.16	524.35	486.08	687.23	326.30	753.60	1,547.32	374.63	241.22	351.54	152.47	5.32	9.24	2.49
173	Gabon	Rate	387.60	742.54	199.60	379.78	734.00	214.16	198.53	280.68	133.27	307.79	631.96	153.01	98.52	143.58	62.27	2.17	3.78	1.02
175	Burundi	Number	5,962.96	10,456.82	2,963.53	6,737.83	10,363.48	4,563.42	5,072.66	7,227.54	3,192.99	3,035.00	6,253.77	1,604.36	2,130.21	3,123.19	1,353.65	46.55	83.06	22.99
175	Burundi	Rate	278.33	488.09	138.33	314.50	483.74	213.01	236.78	337.36	149.04	141.66	291.91	74.89	99.43	145.78	63.18	2.17	3.88	1.07
176	Comoros	Number	352.98	595.60	189.04	334.78	567.27	208.84	181.64	264.68	118.06	232.67	444.89	124.76	91.42	135.00	57.44	2.06	3.70	1.01
176	Comoros	Rate	380.44	641.94	203.75	360.83	611.41	225.09	195.78	285.27	127.25	250.77	479.51	134.47	98.53	145.51	61.91	2.22	3.99	1.09
177	Djibouti	Number	648.80	1,168.97	332.09	592.47	1,048.96	367.74	328.58	478.30	208.47	390.11	757.40	197.72	168.28	247.10	108.90	3.77	6.44	1.89
177	Djibouti	Rate	376.29	677.98	192.61	343.62	608.38	213.28	190.57	277.40	120.91	226.26	439.28	114.67	97.60	143.31	63.16	2.19	3.74	1.10
178	Eritrea	Number	3,142.73	5,309.60	1,734.97	3,084.97	5,062.33	2,051.59	1,646.20	2,400.11	1,061.31	2,011.90	3,734.09	1,191.55	786.92	1,162.99	492.41	17.62	30.93	8.82
178	Eritrea	Rate	395.14	667.59	218.14	387.88	636.50	257.95	206.98	301.77	133.44	252.96	469.49	149.82	98.94	146.23	61.91	2.21	3.89	1.11
179	Ethiopia	Number	51,639.08	87,283.20	29,420.71	54,419.80	89,488.40	35,649.95	32,985.76	47,514.24	20,988.40	33,296.96	64,214.42	18,553.03	15,113.42	22,730.54	9,602.88	334.78	588.47	169.14
179	Ethiopia	Rate	337.85	571.05	192.49	356.04	585.48	233.24	215.81	310.86	137.32	217.85	420.13	121.38	98.88	148.72	62.83	2.19	3.85	1.11
180	Kenya	Number	26,183.80	42,274.93	17,457.58	23,170.91	39,530.76	14,860.93	17,207.84	24,363.62	11,570.57	16,076.18	30,495.49	9,279.10	6,625.82	9,624.86	4,197.36	144.17	228.62	84.49
180	Kenya	Rate	398.32	643.11	265.57	352.49	601.36	226.07	261.77	370.63	176.02	244.56	463.91	141.16	100.80	146.42	63.85	2.19	3.48	1.29
181	Madagascar	Number	12,549.44	21,256.76	6,865.59	13,773.34	22,127.38	9,175.66	10,289.19	14,341.50	6,712.15	7,969.54	15,589.63	4,371.41	3,791.72	5,525.70	2,398.91	84.41	143.54	39.13
181	Madagascar	Rate	322.71	546.62	176.55	354.18	569.01	235.95	264.59	368.79	172.60	204.94	400.89	112.41	97.50	142.09	61.69	2.17	3.69	1.01
182	Malawi	Number	9,983.75	17,412.35	5,122.72	11,347.80	18,088.13	7,495.00	6,968.70	10,146.19	4,511.74	6,505.87	12,675.53	3,594.79	3,167.81	4,770.68	2,018.22	69.56	118.51	32.30
182	Malawi	Rate	311.86	543.91	160.02	354.47	565.02	234.12	217.68	316.94	140.93	203.22	395.94	112.29	98.95	149.02	63.04	2.17	3.70	1.01
183	Mauritius	Number	151.20	254.61	69.35	93.23	141.33	62.13	83.89	122.41	54.14	80.48	124.81	50.94	65.73	94.58	42.05	0.69	1.34	0.25
183	Mauritius	Rate	228.54	384.86	104.82	140.92	213.63	93.91	126.80	185.02	81.84	121.65	188.65	76.99	99.36	142.96	63.56	1.04	2.02	0.38
184	Mozambique	Number	17,024.63	30,092.95	8,852.36	16,970.54	27,861.32	11,075.94	11,083.85	16,047.40	7,042.53	10,535.31	19,903.65	5,868.46	4,864.11	7,152.91	3,064.69	108.37	194.35	47.87
184	Mozambique	Rate	342.81	605.95	178.25	341.72	561.01	223.02	223.18	323.13	141.81	212.14	400.78	118.17	97.94	144.03	61.71	2.18	3.91	0.96
185	Rwanda	Number	6,808.37	11,972.01	3,770.66	6,675.81	11,353.91	4,292.88	3,788.68	5,506.59	2,362.55	4,098.41	8,246.66	2,182.56	1,859.84	2,716.10	1,198.57	41.19	72.73	19.31
185	Rwanda	Rate	363.67	639.49	201.41	356.59	606.47	229.30	202.37	294.14	126.20	218.92	440.50	116.58	99.34	145.08	64.02	2.20	3.88	1.03
186	Seychelles	Number	18.23	30.75	9.21	11.28	17.04	7.59	10.50	15.14	6.73	10.01	15.65	6.39	8.47	12.18	5.34	0.09	0.17	0.03
186	Seychelles	Rate	218.25	368.16	110.34	135.03	204.05	90.94	125.71	181.29	80.55	119.90	187.44	76.47	101.48	145.84	63.99	1.05	2.00	0.40
187	Somalia	Number	3,884.68	6,878.96	2,010.55	4,752.37	7,165.60	3,221.03	3,159.05	4,593.11	2,028.76	2,530.70	4,609.58	1,437.29	1,297.07	1,895.00	826.62	28.83	50.47	14.28
187	Somalia	Rate	294.56	521.61	152.45	360.36	543.34	244.24	239.54	348.28	153.83	191.89	349.53	108.99	98.35	143.69	62.68	2.19	3.83	1.08
189	Tanzania	Number	31,794.49	52,404.71	17,020.30	30,059.45	46,750.96	19,522.36	14,095.05	20,299.01	9,029.70	19,194.67	35,478.99	10,760.53	8,905.47	13,289.37	5,719.36	194.18	350.02	94.41
189	Tanzania	Rate	353.16	582.09	189.05	333.89	519.29	216.85	156.56	225.47	100.30	213.21	394.08	119.52	98.92	147.61	63.53	2.16	3.89	1.05
190	Uganda	Number	28,189.57	46,545.20	14,472.79	25,193.71	39,880.72	16,663.55	10,639.55	15,229.76	6,811.83	14,691.32	27,126.67	8,164.97	7,478.06	11,124.20	4,710.07	162.80	286.73	79.65
190	Uganda	Rate	374.98	619.15	192.52	335.13	530.50	221.66	141.53	202.59	90.61	195.42	360.84	108.61	99.47	147.97	62.65	2.17	3.81	1.06
191	Zambia	Number	9,326.41	15,976.67	4,780.86	8,606.75	14,213.72	5,534.62	5,137.45	7,383.99	3,276.94	5,109.10	10,075.43	2,624.64	2,847.31	4,196.34	1,767.17	61.43	107.79	30.41
191	Zambia	Rate	328.14	562.12	168.21	302.82	500.09	194.73	180.75	259.80	115.29	179.76	354.49	92.34	100.18	147.64	62.18	2.16	3.79	1.07

Table S3. Global and country-specific YLDs of developmental disabilities in 2016 (with 95% uncertainty interval)

			Epilepsy			Intellectual disability			Hearing loss			Vision loss			Autism spectrum disorder			Attention-deficit/hyperactivity disorder		
Loc-ID	Location	Metric	Value	Upper limit	Lower limit	Value	Upper limit	Lower limit	Value	Upper limit	Lower limit	Value	Upper limit	Lower limit	Value	Upper limit	Lower limit	Value	Upper limit	Lower limit
193	Botswana	Number	655.92	1,027.56	372.03	580.43	870.63	401.38	457.26	668.75	288.87	381.22	628.89	232.16	277.67	407.33	176.04	5.85	9.95	2.81
193	Botswana	Rate	247.77	388.15	140.53	219.25	328.87	151.62	172.73	252.61	109.12	144.00	237.56	87.70	104.89	153.86	66.50	2.21	3.76	1.06
194	Lesotho	Number	599.09	992.25	331.58	732.48	1,036.06	518.88	569.67	831.00	356.53	420.82	700.74	259.08	267.10	390.78	170.37	5.68	10.07	2.65
194	Lesotho	Rate	232.00	384.26	128.41	283.66	401.23	200.94	220.61	321.81	138.07	162.97	271.37	100.33	103.44	151.34	65.98	2.20	3.90	1.03
195	Namibia	Number	832.12	1,361.62	442.23	834.65	1,287.22	551.50	629.44	921.21	385.22	560.48	981.13	326.03	340.18	500.64	219.70	7.27	12.52	3.44
195	Namibia	Rate	250.00	409.09	132.87	250.76	386.74	165.69	189.11	276.77	115.74	168.39	294.78	97.95	102.21	150.41	66.01	2.18	3.76	1.03
196	South Africa	Number	13,986.96	22,889.30	9,080.80	13,247.25	21,528.58	8,802.25	12,368.26	17,196.20	8,370.46	9,242.19	16,669.50	5,377.52	5,166.20	7,535.36	3,284.60	109.19	175.97	64.14
196	South Africa	Rate	279.11	456.76	181.21	264.35	429.61	175.65	246.81	343.16	167.04	184.43	332.65	107.31	103.09	150.37	65.55	2.18	3.51	1.28
197	Swaziland	Number	532.12	866.37	288.77	518.30	774.37	358.02	414.88	601.63	261.66	339.51	560.28	199.48	216.40	320.39	137.01	4.58	7.77	2.14
197	Swaziland	Rate	255.17	415.45	138.48	248.54	371.34	171.68	198.95	288.50	125.47	162.81	268.67	95.66	103.77	153.64	65.70	2.19	3.73	1.03
198	Zimbabwe	Number	7,611.30	13,876.03	3,866.11	8,933.16	15,476.85	5,613.35	3,929.22	5,529.97	2,631.36	5,726.91	11,518.80	2,880.48	2,620.61	3,877.55	1,680.70	55.19	96.04	26.71
198	Zimbabwe	Rate	300.52	547.87	152.65	352.71	611.08	221.63	155.14	218.34	103.89	226.12	454.80	113.73	103.47	153.10	66.36	2.18	3.79	1.05
200	Benin	Number	6,522.87	11,068.48	3,334.45	6,441.67	10,285.84	4,318.06	3,674.39	5,143.83	2,395.69	4,642.17	7,981.87	2,784.52	1,826.39	2,713.76	1,156.13	42.30	73.29	20.27
200	Benin	Rate	341.38	579.28	174.51	337.13	538.32	225.99	192.30	269.21	125.38	242.95	417.74	145.73	95.59	142.03	60.51	2.21	3.84	1.06
201	Burkina Faso	Number	8,284.97	14,445.85	4,341.34	10,498.45	16,191.64	7,323.11	6,636.45	9,282.72	4,395.60	5,763.13	10,592.21	3,441.57	3,043.04	4,430.09	1,939.88	70.39	124.42	34.91
201	Burkina Faso	Rate	259.33	452.17	135.89	328.61	506.82	229.22	207.73	290.56	137.59	180.39	331.55	107.73	95.25	138.67	60.72	2.20	3.89	1.09
202	Cameroon	Number	12,404.77	22,886.03	6,016.98	12,319.93	19,946.20	8,183.33	6,786.62	9,651.30	4,466.26	7,360.10	13,660.48	4,108.75	3,753.85	5,439.68	2,371.91	87.38	155.14	43.62
202	Cameroon	Rate	319.94	590.27	155.19	317.75	514.45	211.06	175.04	248.92	115.19	189.83	352.33	105.97	96.82	140.30	61.18	2.25	4.00	1.13
203	Cape Verde	Number	267.94	440.23	146.49	253.83	410.54	165.10	113.56	161.24	74.52	144.95	280.96	83.30	72.87	106.94	44.98	1.73	2.96	0.86
203	Cape Verde	Rate	356.87	586.34	195.11	338.07	546.80	219.90	151.25	214.75	99.25	193.06	374.21	110.95	97.06	142.43	59.91	2.30	3.94	1.14
204	Chad	Number	6,378.17	11,460.31	3,080.43	7,634.90	10,785.82	5,426.26	5,327.10	7,507.37	3,494.65	4,437.59	7,125.58	2,738.01	2,493.95	3,665.76	1,594.40	57.90	97.44	29.67
204	Chad	Rate	241.46	433.85	116.62	289.03	408.32	205.42	201.67	284.21	132.30	167.99	269.75	103.65	94.41	138.78	60.36	2.19	3.69	1.12
205	Cote d'Ivoire	Number	9,893.55	17,059.13	5,314.85	11,056.15	17,584.00	7,603.80	6,359.01	9,009.54	4,163.79	7,100.70	12,752.99	4,213.75	3,416.30	5,044.68	2,161.86	79.42	138.70	36.63
205	Cote d'Ivoire	Rate	278.01	479.36	149.35	310.68	494.11	213.67	178.69	253.17	117.00	199.53	358.36	118.41	96.00	141.76	60.75	2.23	3.90	1.03
206	The Gambia	Number	1,413.62	2,314.80	821.90	1,423.68	2,324.76	946.47	717.93	1,035.84	463.85	916.69	1,726.96	526.72	352.94	509.69	227.83	8.15	14.41	3.97
206	The Gambia	Rate	385.57	631.37	224.17	388.31	634.09	258.15	195.82	282.53	126.52	250.03	471.03	143.66	96.27	139.02	62.14	2.22	3.93	1.08
207	Ghana	Number	17,759.46	30,423.57	9,726.34	16,141.99	27,011.66	10,173.43	7,200.92	10,334.02	4,751.95	11,084.26	21,464.91	5,808.99	4,251.88	6,243.90	2,679.68	98.40	169.20	51.31
207	Ghana	Rate	408.60	699.97	223.78	371.38	621.47	234.06	165.67	237.76	109.33	255.02	493.85	133.65	97.82	143.66	61.65	2.26	3.89	1.18
208	Guinea	Number	5,670.27	10,017.53	2,971.62	6,796.42	10,516.59	4,724.04	4,188.17	5,934.36	2,734.69	3,986.62	7,260.44	2,378.70	1,934.87	2,852.19	1,207.85	45.25	79.65	22.54
208	Guinea	Rate	278.65	492.29	146.03	334.00	516.81	232.15	205.82	291.63	134.39	195.91	356.80	116.90	95.08	140.16	59.36	2.22	3.91	1.11
209	Guinea-Bissau	Number	815.27	1,468.13	426.83	1,048.21	1,629.46	715.09	628.70	895.66	409.17	604.79	1,113.12	353.85	297.26	435.22	183.13	6.94	12.31	3.36
209	Guinea-Bissau	Rate	263.01	473.63	137.70	338.16	525.68	230.69	202.83	288.95	132.00	195.11	359.10	114.15	95.90	140.41	59.08	2.24	3.97	1.08
210	Liberia	Number	2,223.76	3,736.07	1,134.36	2,408.89	3,534.99	1,684.97	1,492.91	2,110.69	974.05	1,362.49	2,305.26	840.61	686.23	1,003.14	435.09	16.22	30.20	7.94
210	Liberia	Rate	311.97	524.13	159.14	337.94	495.92	236.38	209.44	296.10	136.65	191.14	323.40	117.93	96.27	140.73	61.04	2.28	4.24	1.11
211	Mali	Number	7,628.63	13,371.44	3,977.38	10,123.02	15,129.81	6,910.43	6,543.97	9,195.08	4,306.61	6,316.65	10,891.00	3,928.44	2,988.66	4,410.82	1,934.26	69.75	121.13	32.06
211	Mali	Rate	242.18	424.49	126.27	321.36	480.31	219.38	207.74	291.91	136.72	200.53	345.74	124.71	94.88	140.02	61.40	2.21	3.85	1.02
212	Mauritania	Number	2,230.90	3,853.83	1,184.73	1,956.22	3,375.63	1,224.49	856.35	1,203.17	560.52	1,543.71	2,905.15	889.56	499.99	737.00	320.53	11.68	20.65	5.79
212	Mauritania	Rate	433.71	749.23	230.33	380.31	656.26	238.06	166.48	233.91	108.97	300.12	564.80	172.94	97.20	143.28	62.31	2.27	4.02	1.13
213	Niger	Number	8,780.30	15,666.20	4,381.93	11,936.46	16,997.07	8,381.36	8,514.82	12,216.40	5,532.54	6,581.43	11,004.79	3,995.06	3,491.16	5,156.00	2,191.39	80.97	142.48	40.53
213	Niger	Rate	238.36	425.30	118.96	324.04	461.43	227.53	231.16	331.64	150.19	178.67	298.75	108.46	94.78	139.97	59.49	2.20	3.87	1.10
214	Nigeria	Number	81,849.66	146,226.59	41,501.48	89,953.48	145,972.83	58,902.37	56,430.33	78,010.11	37,323.72	59,551.10	107,427.70	33,990.91	28,378.31	42,222.87	17,629.09	660.53	1,105.43	339.18
214	Nigeria	Rate	277.14	495.12	140.52	304.58	494.26	199.44	191.07	264.14	126.38	201.64	363.74	115.09	96.09	142.96	59.69	2.24	3.74	1.15
215	Sao Tome and Principe	Number	134.30	223.04	73.76	117.81	200.98	75.51	58.99	83.44	38.64	82.26	160.42	46.44	32.18	47.09	20.82	0.76	1.31	0.37
215	Sao Tome and Principe	Rate	403.31	669.81	221.52	353.79	603.56	226.76	177.15	250.58	116.04	247.03	481.75	139.48	96.65	141.41	62.53	2.28	3.94	1.11
216	Senegal	Number	9,912.90	16,454.22	5,309.46	9,455.27	15,395.68	6,176.01	4,909.49	6,942.72	3,240.25	6,363.89	11,805.13	3,620.49	2,450.15	3,516.78	1,533.77	56.65	97.80	27.22
216	Senegal	Rate	389.96	647.29	208.87	371.96	605.65	242.96	193.13	273.12	127.47	250.35	464.40	142.43	96.39	138.35	60.34	2.23	3.85	1.07
217	Sierra Leone	Number	2,593.51	4,628.38	1,290.46	3,308.47	5,007.39	2,318.75	1,682.29	2,348.32	1,103.47	1,908.28	3,327.98	1,191.76	986.21	1,476.63	610.99	23.03	41.05	11.54
217	Sierra Leone	Rate	249.39	445.06	124.09	318.14	481.50	222.97	161.77	225.81	106.11	183.50	320.01	114.60	94.83	141.99	58.75	2.21	3.95	1.11
218	Togo	Number	3,755.19	6,440.30	1,999.55	3,971.30	6,348.86	2,669.99	2,094.52	2,991.55	1,363.60	2,446.04	4,470.14	1,451.12	1,056.10	1,557.42	676.42	24.59	42.19	12.05
218	Togo	Rate	343.34	588.84	182.82	363.10	580.48	244.12	191.50	273.52	124.67	223.64	408.71	132.68	96.56	142.40	61.85	2.25	3.86	1.10
298	American Samoa	Number	11.04	17.86	5.79	8.64	11.79	6.07	10.06	14.18	6.63	7.54	11.27	4.98	7.46	10.90	4.70	0.12	0.21	0.05
298	American Samoa	Rate	147.97	239.28	77.55	115.72	157.93	81.26	134.80	189.98	88.82	101.05	151.00	66.78	99.94	146.02	63.03	1.57	2.77	0.73
305	Bermuda	Number	5.43	9.53	2.55	5.67	8.41	3.89	4.29	6.05	2.82	4.57	6.97	2.96	4.68	6.89	2.97	0.15	0.26	0.08
305	Bermuda	Rate	123.74	217.24	58.21	129.30	191.78	88.79	97.84	137.90	64.29	104.10	158.93	67.46	106.70	156.98	67.69	3.48	5.82	1.77

Table S3. Global and country-specific YLDs of developmental disabilities in 2016 (with 95% uncertainty interval)																				
			Epilepsy			Intellectual disability			Hearing loss			Vision loss			Autism spectrum disorder			Attention-deficit/hyperactivity disorder		
**Loc-ID**	**Location**	**Metric**	**Value**	**Upper limit**	**Lower limit**	**Value**	**Upper limit**	**Lower limit**	**Value**	**Upper limit**	**Lower limit**	**Value**	**Upper limit**	**Lower limit**	**Value**	**Upper limit**	**Lower limit**	**Value**	**Upper limit**	**Lower limit**
349	Greenland	Number	3.60	6.56	1.63	6.14	8.37	4.36	2.71	3.80	1.79	2.22	3.53	1.40	3.86	5.62	2.47	0.09	0.17	0.05
349	Greenland	Rate	105.38	192.22	47.83	180.03	245.16	127.67	79.48	111.47	52.42	65.15	103.36	41.09	113.23	164.75	72.36	2.76	4.90	1.37
351	Guam	Number	24.60	41.35	12.43	19.52	28.46	13.40	19.28	26.89	12.76	17.49	26.56	11.13	16.60	24.20	10.65	0.26	0.47	0.11
351	Guam	Rate	146.85	246.91	74.20	116.56	169.91	79.99	115.11	160.57	76.17	104.44	158.57	66.47	99.13	144.47	63.58	1.54	2.82	0.64
376	Northern Mariana Islands	Number	21.12	35.62	10.72	16.35	22.20	11.53	19.61	27.96	13.13	14.04	21.08	9.34	15.32	22.49	9.53	0.23	0.43	0.10
376	Northern Mariana Islands	Rate	138.41	233.43	70.28	107.13	145.47	75.53	128.53	183.25	86.06	92.03	138.16	61.20	100.37	147.36	62.44	1.53	2.79	0.68
385	Puerto Rico	Number	472.01	877.66	243.98	525.24	876.86	327.95	211.37	301.74	138.50	392.79	721.76	231.01	226.26	328.64	144.83	7.36	12.33	3.79
385	Puerto Rico	Rate	225.22	418.78	116.42	250.62	418.40	156.48	100.86	143.98	66.09	187.42	344.39	110.23	107.96	156.81	69.11	3.51	5.89	1.81
422	Virgin Islands, U.S.	Number	12.52	22.42	6.61	13.76	24.90	8.06	5.50	7.87	3.58	9.97	17.87	5.82	5.95	8.74	3.79	0.20	0.34	0.10
422	Virgin Islands, U.S.	Rate	226.77	405.93	119.77	249.17	450.89	146.02	99.64	142.46	64.84	180.57	323.63	105.48	107.77	158.22	68.71	3.61	6.18	1.88
435	South Sudan	Number	7,158.36	12,876.07	3,719.29	8,219.75	13,206.44	5,591.29	6,586.81	9,518.81	4,157.16	6,719.59	11,558.75	4,202.87	2,800.21	4,135.62	1,795.50	60.81	108.82	28.34
435	South Sudan	Rate	250.27	450.17	130.03	287.38	461.72	195.48	230.29	332.79	145.34	234.93	404.11	146.94	97.90	144.59	62.77	2.13	3.80	0.99
522	Sudan	Number	11,767.79	24,778.58	5,294.23	15,996.62	30,127.78	10,328.53	7,161.34	10,185.74	4,568.86	11,249.80	24,410.33	6,164.24	4,412.89	6,353.15	2,808.68	75.79	135.57	34.74
522	Sudan	Rate	280.62	590.89	126.25	381.47	718.45	246.30	170.78	242.90	108.95	268.27	582.11	147.00	105.23	151.50	66.98	1.81	3.23	0.83

Appendix 1

Section 2. Nonfatal outcome estimation

The GBD 2016 nonfatal estimation process is visually represented in Appendix Figures 1a and 1b illustrating the steps necessary to estimate incidence, prevalence, and YLDs for disease and injury sequelae in GBD 2016. Appendix Figure 1a outlines the general process of nonfatal outcome estimation from data inputs to finalization of YLD burden results; steps 3b and 3c of that process identify alternative modelling approaches employed for certain causes and injuries. Alternative approaches are illustrated in greater detail in Appendix Figure 1b. Conceptually, the estimation effort is divided into eight major components: (1) compiling data sources through data identification and extraction; (2) data adjustment; (3) estimation of prevalence and incidence by cause and sequelae using DisMod-MR 2.1 or alternative modelling strategies for selected cause groups; (4) estimation by impairment; (5) severity distributions; (6) incorporation of disability weights; (7) comorbidity adjustment; and (8) the estimation of YLDs by sequelae and causes. Appendix Section 3 contains additional detail specific to each disease, impairment and injury and their sequelae. Nonfatal modelling strategies vary significantly between causes.

2.1 Data sources, identification and extraction

2.1.1 Systematic reviews

For GBD 2015, updated systematic reviews were conducted for 101 causes and sequelae using data available through January of 2015. For GBD 2016 we conducted literature reviews for 116 causes and 4 impairments. For other diseases, only a small fraction of the existing data appears in the published literature and other sources predominate such as survey data, disease registers, notification data, hospital inpatient data, or claims data. Data were systematically screened from household surveys archived in the Global Health Data Exchange (ghdx.healthdata.org), including Demographic and Health Surveys, Multiple Indicator Cluster Surveys, Living Standards Measurement Surveys, and Reproductive Health Surveys. Other national health surveys were identified based on survey series that had yielded usable data for past rounds of GBD, sources suggested to us by in-country collaborators, and surveys identified in major multinational survey data catalogs, such as the International Household Survey Network and the World Health Organization (WHO) Central Data Catalog, as well as through country Ministry of Health and Central Statistical Office websites. Case notifications reported to the WHO were updated through 2016. Citations for all data sources used for nonfatal estimation in GBD 2016 are provided in searchable form through a web-tool (http://ghdx.healthdata.org/). A description of the search terms employed for cause-specific systematic reviews are detailed by cause in Appendix Section 3.

2.1.2 Survey data preparation

For GBD 2016, survey data for which we had access to the unit record data constituted a substantial part of the underlying data used in the estimation process. During extraction, we concentrated on demographic variables (such as location, sex, age), survey design variables (such as sampling strategy and sampling weights), and the variables used to define the population estimate (such as prevalence or a proportion) and a measure of uncertainty (standard error, confidence interval or sample size and number of cases).

Appendix Figure 1a: Overview analytical flowchart for DisMod-MR 2.1 modelling strategies and injuries, GBD 2016

Ovals represent data inputs, square boxes represent analytical steps, cylinders represent databases, and parallelograms represent intermediate and final results. The flowchart is color-coded by major estimation component: raw data sources, in pink; data adjustments, in yellow; DisMod-MR 2.1 estimation, in purple; alternative modelling strategies, in light green; injury modeling strategy, in dark green; estimation of impairments and underlying causes, in brown; severity distributions and comorbidity correction, in blue; disability weights in orange; and cause of death and demographic inputs, in grey. GBD = Global Burden of Disease; TB=tuberculosis; HIV =human immunodeficiency virus; AIDS=acquired immunodeficiency syndrome; SF-12=Short Form 12 questions; MEPS=Medical Expenditure Panel Surveys; CSMR=cause-specific mortality rate; SMR=standardized mortality ratio; YLDs=years lived with disability; YLLs=years of life lost.

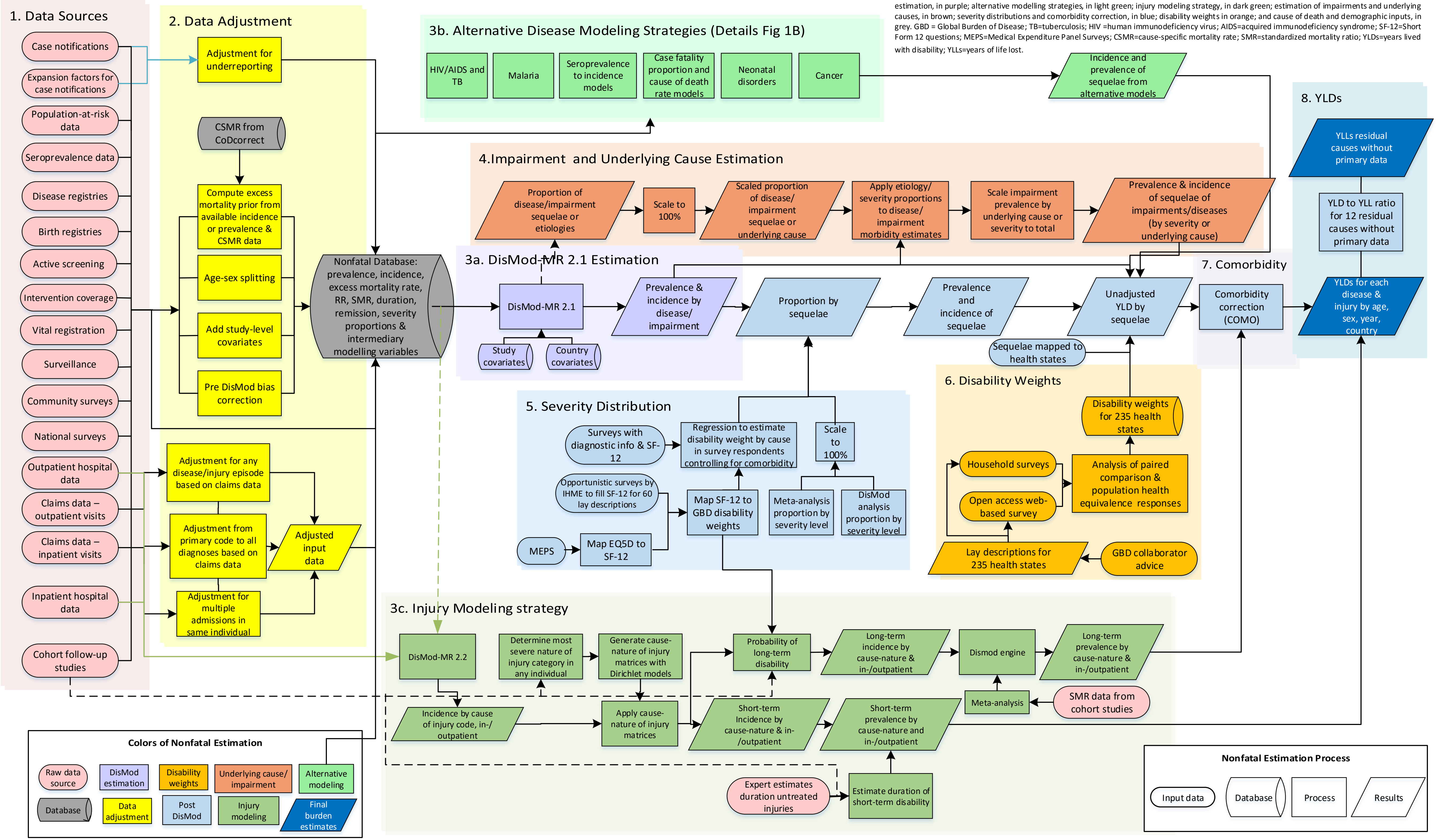

Appendix Figure 1b: Analytical flowchart for modelling strategies other than DisMod-MR 2.1 and injuries for selected nonfatal cause groups, GBD 2016

Ovals represent data inputs, square boxes represent analytical steps, cylinders represent databases, and parallelograms represent intermediate and final results. The flowchart is color-coded by major estimation component: raw data sources, in pink; data adjustments, in yellow; DisMod-MR 2.1 estimation, in purple; alternative modelling strategies, in light green; injury modeling strategy, in dark green; estimation of impairments and underlying causes, in brown; severity distributions and comorbidity correction, in blue; disability weights in orange; and cause of death and demographic inputs, in grey. GBD = Global Burden of Disease; TB=tuberculosis; HIV =human immunodeficiency virus; AIDS=acquired immunodeficiency syndrome; SF-12=Short Form 12 questions; MEPS=Medical Expenditure Panel Surveys; CSMR=cause-specific mortality rate; SMR=standardized mortality ratio; YLDs=years lived with disability; YLLs=years of life lost.

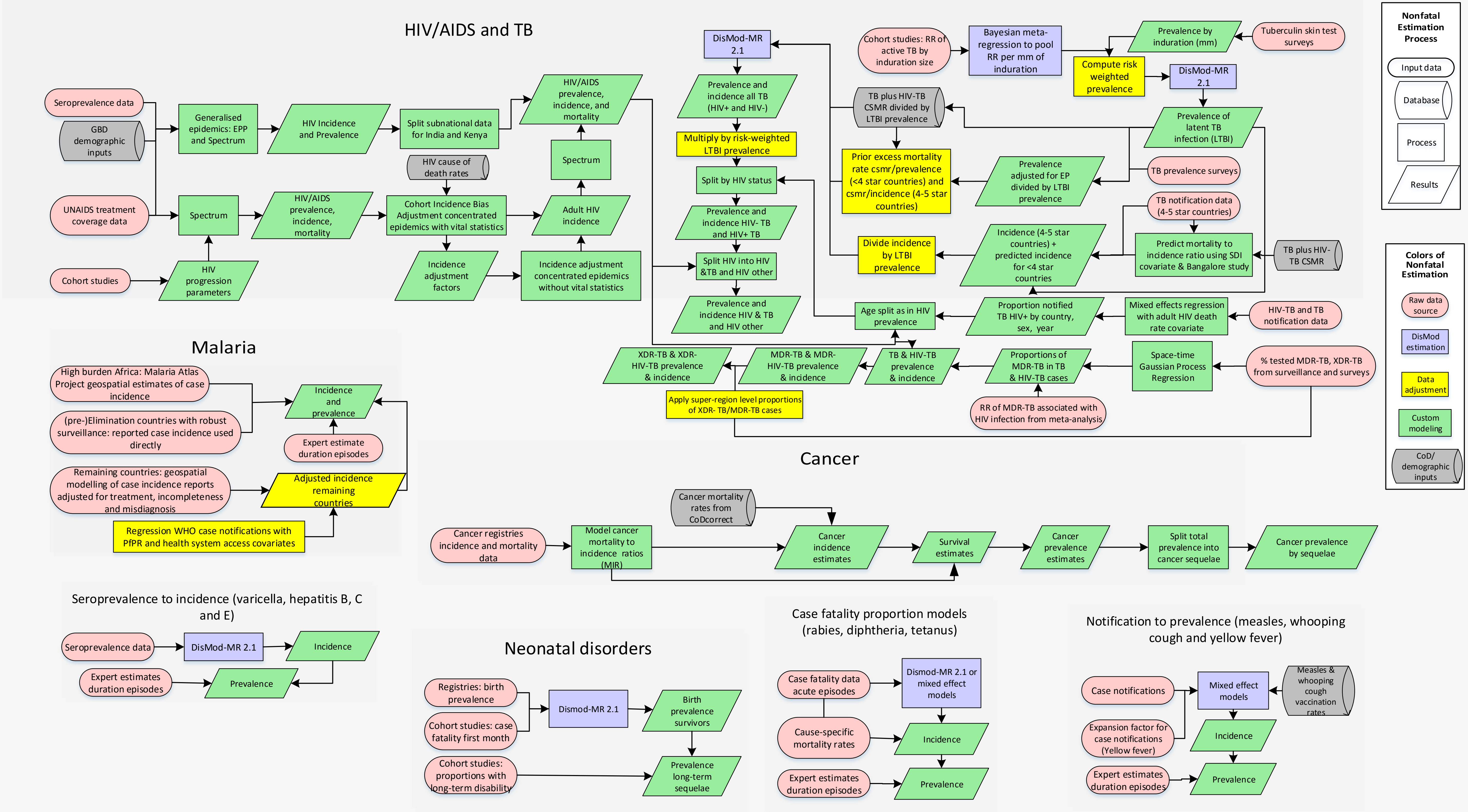

2.1.3 Disease Registers

For GBD 2016 nonfatal estimation, disease registries were an important source for a select number of conditions such as cancers, end-stage renal disease, and congenital disorders. The GHDx source tool (http://ghdx.healthdata.org/data-type/disease-registry)[2] provides a comprehensive list of registry data used in GBD estimation processes.

2.1.4 Estimation of Hospital Envelope

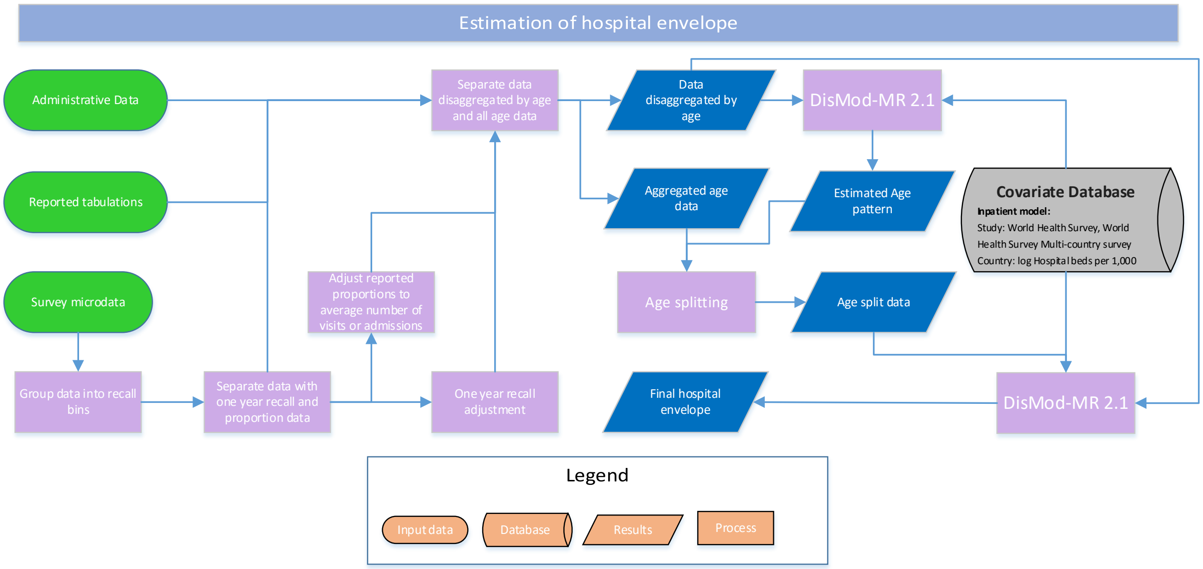

Input data and methodological summary

Case definition

We defined a hospital admission as the overnight admission into a formal healthcare facility but excluded admissions to long-term care (>120 days), nursing care facilities, and traditional or spiritual healers.

Input Data

We searched the GHDx for population surveys, administrative records, and censuses from January 1990 to September 2016. We applied five secondary data filters: “discharge”, “health facility”, “nationally representative”, “household”, or “outpatient.” We also applied ten keyword filters: “healthcare access”, “health care costs”, “healthcare economics”, “healthcare expenditure”, “healthcare services”, “healthcare use”, “outpatient facilities”, “patient counts”, “hospitals”, or “length of stay”. We applied no language restrictions to our search and required all returned records to either contain microdata or tabulated reports. We searched the returned records’ metadata looking for measures of inpatient care. For inclusion, we required all measures to be nationally or subnationally representative. Additionally, we consulted with experts and GBD collaborators to gather data sources that were not within the GHDx. In total, we accepted data sources from 2,855 location-years (1,560 from administrative records and 1,295 from population surveys).

Modelling Strategy

Data adjustment

We classified each of the accepted data sources into four data types: (a) proportion of survey respondents who were admitted into the hospital in the past 30 days; (b) proportion of survey respondents who were admitted to the hospital in the past year; (c) average number of admissions (utilization rate) reported by survey respondents in the past year; and (d) average number of visits reported by annual administrative records. We assigned measures reported by annual administrative records as our reference group as these data types were free from recall bias and most closely matched our case definition. In data sources where microdata was available, we extracted and binned the data based on gender and age groups of under-1, 1-4, 4-9, 10-14, through till 95+ years of age.

We crosswalked each of the three non-reference (survey) data types to the reference (administrative record) data type through the use of penalized spline regressions to account for non-systematic differences between the data types. For each non-reference data type and each sex, we looked for overlap between the non-reference data type and reference data type on the basis of location, year, age group, and sex. With the overlapping data, we calculated the ratio of the point estimate from the reference data type, μ_{ref}, to the non-reference data type, μ_s. We fit these ratios with a penalized spline regression shown below

$$\ln\left(\frac{\mu_{ref,i}}{\mu_{s,i}}\right) = h(age_i) + \varepsilon_i \ (1)$$

Where i denotes a given matched observation, $h(age_i)$ represents a basis function which estimated a cross-validated penalized spline over the population weighted mean age of the age group, and ε represents the residual. In the below figures, for each non-reference data type, we plot the ratio of μ_{ref} and μ_s across age and by sex and the predictions from the penalized spline regressions

Figure. Global age-sex specific crosswalks to equate each non-reference data type top the reference data type. For each non-reference data type and each sex, we plot the ratio of reference data points to non-reference data points, which were matched based on location, age group, year, and sex. Using a penalized spline regression, we estimated the crosswalk between each non-reference data type and the reference type. We plot the crosswalk and the associated prediction error below.

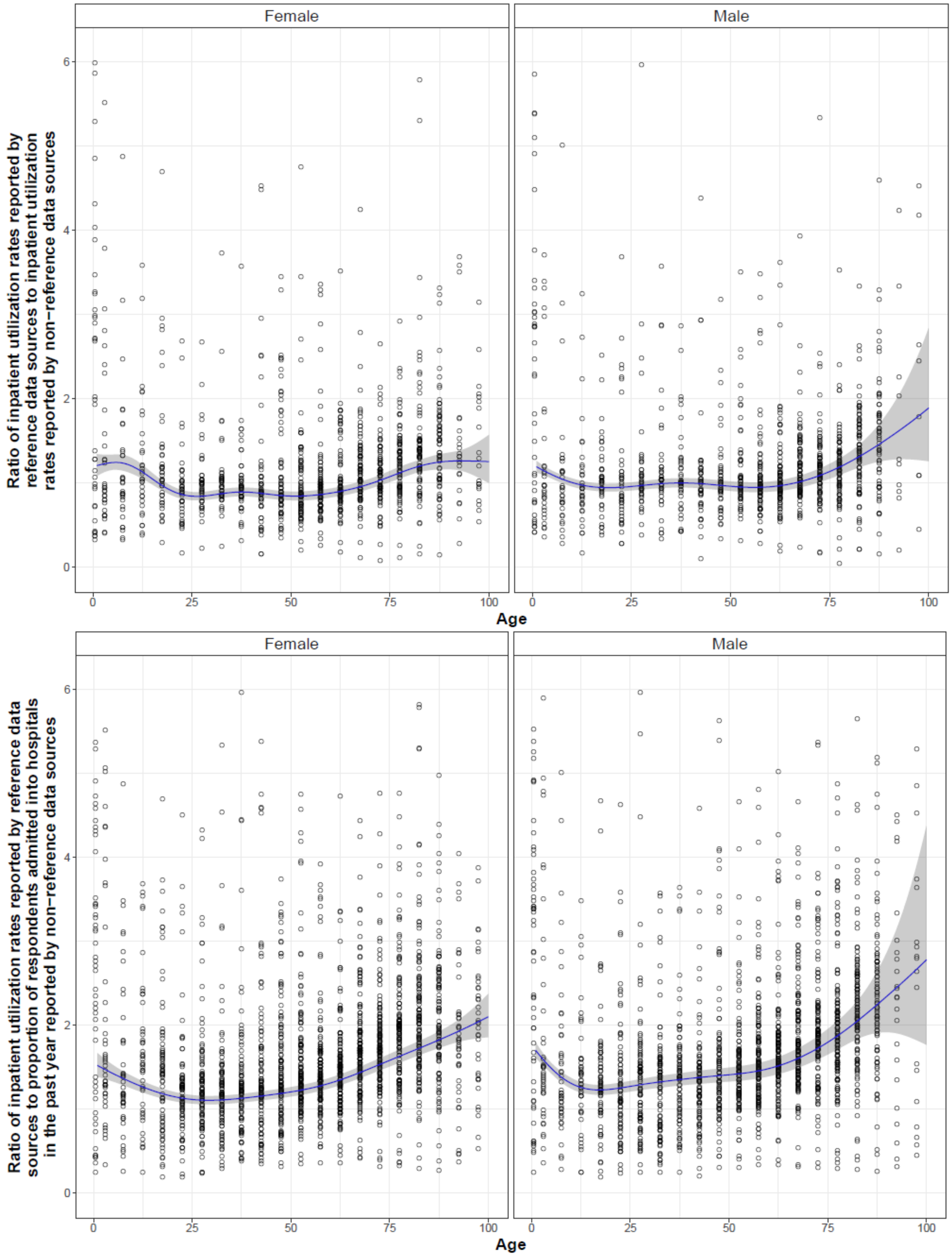

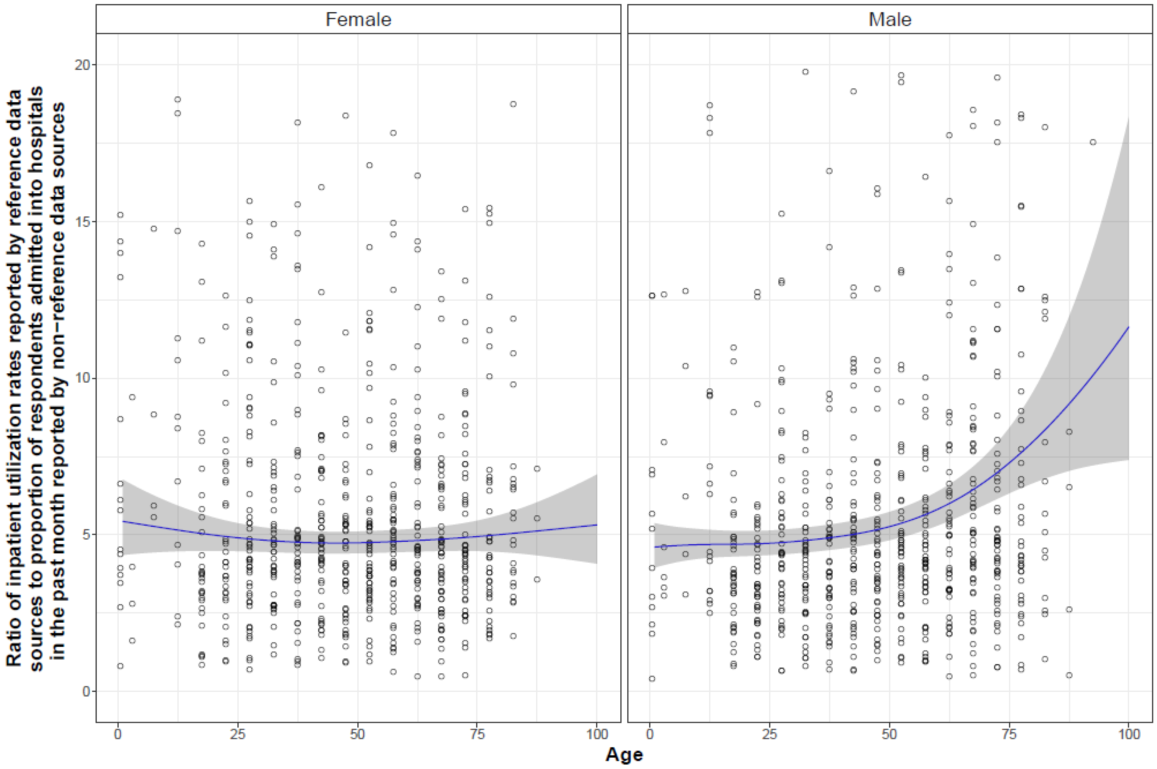

To crosswalk non-reference data types to reference data types, we multiplied non-reference data types by the exponentiated predictions from respective penalized spline regressions. Uncertainty from the adjustments were accounted for using the following equation

$$se_a = \sqrt{se_m^2 \cdot se_s^2 + se_m^2 \cdot \mu_s^2 + se_s^2 \cdot \mu_m^2} \ (2)$$

Where se_a, se_m, and se_s are the standard errors of the adjusted non-reference data point, the exponentiated crosswalk prediction, and the non-reference data point, respectively. μ_s and μ_m are the means of the non-reference data point and the exponentiated crosswalk predictions from the penalized spline regressions.

Age-sex splitting

DisMod-MR 2.1 is capable of conducting age-integration but its performance degrades while integrating across wide age categories (e.g. all ages). To remedy this issue, we ran a DisMod-MR 2.1 model with data that was disaggregated by age to estimate countries' age-pattern and then applied the estimated age-pattern to split aggregated all age data. This procedure was operationalized by calculating a constant, k, which was the ratio of the aggregated all age data point, $\mu_{all\ age}$, to the all age estimated utilization rate from the DisMod-MR 2.1 model, $\widehat{\mu_d}$

$$k = \frac{\mu_{all\ age}}{\widehat{\mu_d}} \ (3)$$

The constant, k, was then multiplied by age specific utilization rates from the DisMod-MR 2.1 model. The uncertainty from the data and the age-pattern were propagated following equation 2. The split data were then incorporated into the final DisMod-MR 2.1 model.

DisMod Modelling

To help explain variation in locations with little to no data, we used the country level covariate of natural log of hospital beds per 1,000. Study level covariates were used to denote World Health Surveys and World Health Organization's Multi-Country Survey Study on Health System Responsiveness. The country-level covariate of hospital beds per 1,000 was estimated using ST-GPR on data sourced from the World Bank. The study-level covariates were used as these two survey series were systematically higher than data points from other sources in the same locations and time period. Coefficients for the covariates are presented in below table.

Table. Estimated coefficients of the hospital envelope model.
Study-level covariates denote global dichotomous covariates that serve to adjust corresponding data points

Covariate	type	Coefficient	Exponentiated coefficient
World Health Survey	Study-level	0.44<br>(0.39 — 0.48)	1.55<br>(1.48 — 1.61)
World Health Organization Multi-country Survey Study on Health and Responsiveness	Study-level	0.49<br>(0.42 — 0.55)	1.63<br>(1.52 — 1.73)
log Hospital beds per 1,000	Country-level	0.17<br>(0.16 — 0.19)	1.19<br>(1.17 — 1.20)

2.1.5 Claims, inpatient hospital, and outpatient data

For GBD 2016, claims (linkage) data, inpatient hospital, and outpatient data played a key role in the nonfatal estimation process of many GBD causes.

Claims data

For GBD 2016, we accessed aggregate data derived from claims information in a database of US private health insurance and public insurance schemes of Medicaid and Medicare, for the years 2000, 2010, and 2012, commonly referred to as Marketscan. The population covered in each year was 3.3 million in 2000, 40.4 million in 2010, and 40.8 million in 2012. For each of these individuals, information on every health service encounter was collected and all episodes of care were linked to individuals by unique identifiers, which allowed us to aggregate data in multiple ways, including creating counts of claims and individuals for inpatient and outpatient care. Marketscan has fifteen diagnosis columns for both inpatient and outpatient episodes. We mapped all ICD-9 four- or five-digit-coded diagnoses to GBD causes (see Appendix Table 4). GBD conditions were categorized as "long-term" or "short-term" depending on cause duration. In a given year, for each individual in the claims data, a long-term case was defined as any mention in any diagnostic field associated with any claim, including inpatient and outpatient encounters. A short-term case was defined the same way, but assumed that claims within a condition-specific duration were the same case. In this way, an individual could have multiple short-term conditions in a given year, while avoiding double-counting cases with multiple claims from a single illness episode.

In GBD 2015, a subset of available facility types were used for short-term causes in outpatient claims data. In GBD 2016, we added more facility types, including the "office visit" facility type, which accounts for more than 50% of all outpatient data.

GBD 2016 Marketscan/Claims data extraction process

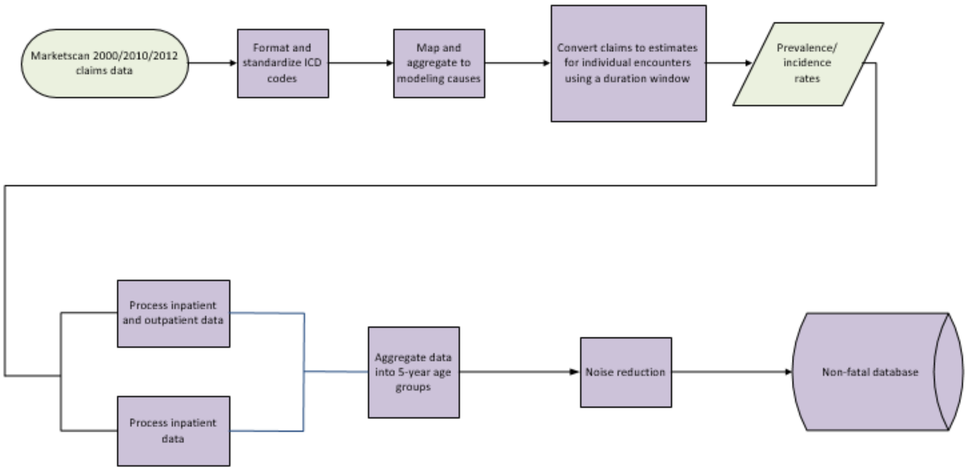

Inpatient hospital admissions

Inpatient hospital data were extracted from 3557 location-years in 41 countries. ICD coding was standardized across sources, and versions of ICD (Appendix Table 4).

For GBD 2015, one limitation of our use of hospital data in non-fatal disease estimation was the challenge of accessing accurate information on coverage populations for any given data source. Section 2.1.4 of the appendix describes the modelling strategy for the hospital envelope, an estimate of hospital utilization, i.e. the rate of inpatient episodes per capita by age, sex, year and location. For GBD 2016, we used this hospital utilization envelope in place of information on coverage population. We calculated demographic-specific (by age, sex, year, and location) cause fractions in each inpatient hospital data source and multiplied these by the hospital utilization envelope to produce a rate of incidence/prevalence in inpatient admissions. For countries with completed registration of all inpatient episodes, this method makes little difference compared to the previous approach of dividing inpatient episode numbers by population as the all-cause inpatient rates per population from these sources were inputs to the utilization model. For countries with incomplete coverage, where in the past we had to make assumptions on the proportion of the population covered or reject the source because of unknown catchment population, this method is an improvement.

Using the Marketscan claims data described above (with the exception of Marketscan 2000 data, due to the low coverage compared to two later years), we generated three scalars that were applied to the product of cause fractions generated by the inpatient hospital data and the utilization envelope on a

cause-by-cause basis. The scalars account for bias in inpatient hospital data from sources which were aggregated by ICD code and by primary diagnosis only. First, we corrected to account for multiple admissions for an individual. Second, we adjusted for non-primary diagnoses. Third, we corrected to account for inpatient and outpatient care. Combined with the uncorrected version (no scalar applied), this resulted in four types of incidence and prevalence estimates from inpatient hospital data: 1) (un-corrected) inpatient admissions by episode, primary diagnosis, 2) inpatient admissions by individual, primary diagnosis only, 3) inpatient hospital admissions, accounting for all diagnoses, 4) an estimate of any inpatient admission or outpatient visit by an individual, accounting for all diagnoses. These data were reviewed in conjunction with data from all other sources for each model that utilizes hospital data to determine which type of data adjustment was most appropriate as an input to non-fatal disease estimation.

The equations we used for each of the three scalars can be found below:

a) Adjustment to account for multiple admissions which gave us inpatient admissions by individual, primary diagnosis only
   a. $inpatient^{1°}_{episode} * \left(\frac{MS\ inpatient^{1°}_{indiv}}{MS\ inpatient^{1°}_{episode}}\right) = inpatient^{1°}_{indiv}$
b) Adjustment for non-primary diagnoses which gave us inpatient admissions by individual, all diagnoses
   a. $inpatient^{1°}_{episode} * \left(\frac{MS\ inpatient^{all}_{indiv}}{MS\ inpatient^{1°}_{episode}}\right) = inpatient^{all}_{indiv}$
c) Adjustment to account for inpatient and outpatient care which gave us inpatient admissions and outpatient visits by individual, all diagnoses
   a. $inpatient^{1°}_{episode} * \left(\frac{MS\ inpatient^{all}_{indiv} \cup MS\ outpatient^{all}_{indiv}}{MS\ inpatient^{1°}_{episode}}\right) = inpatient|outpatient^{all}_{indiv}$

For maternal causes, a new bundle was created that had every maternity-related ICD code mapped to it. At the end of the process, each maternal cause rate was divided by the rate of this all maternity bundle in order to adjust the denominators from population to live births.

Congenital and neonatal causes with similar age patterns were aggregated to create correction factors because the data in individual causes were too sparse to make them reasonable, and would leave the age pattern mostly flat. Injuries used a separate correction factor as well.

These scalars were then smoothed by fitting a Loess curve to the observed data for each combination of cause and sex. The span of the Loess smoothing varied depending on how many observable data points there were. For cause/sex subsets with 5 or fewer observations a span (fraction of number of points to consider when fitting a curve) of 1 was set. For 6 to 10 observations, a span of 0.75 was set, and for 11 or more observations, a span of 0.5 was set.

In cases where the third scalar, accounting for inpatient and outpatient care, was greater than 50, we determined it to be unstable and did not apply this scalar to the hospital data. Exceptions to this rule

were for congenital and neonatal causes (preterm births, neonatal hemoloytic disease, encephalopathy, sepsis), where babies hospitalized with these conditions often have comorbid states that make it very likely that the given code would not be listed as primary, as well as peripheral arterial disease and cirrhosis. For these causes, the exception is 100.

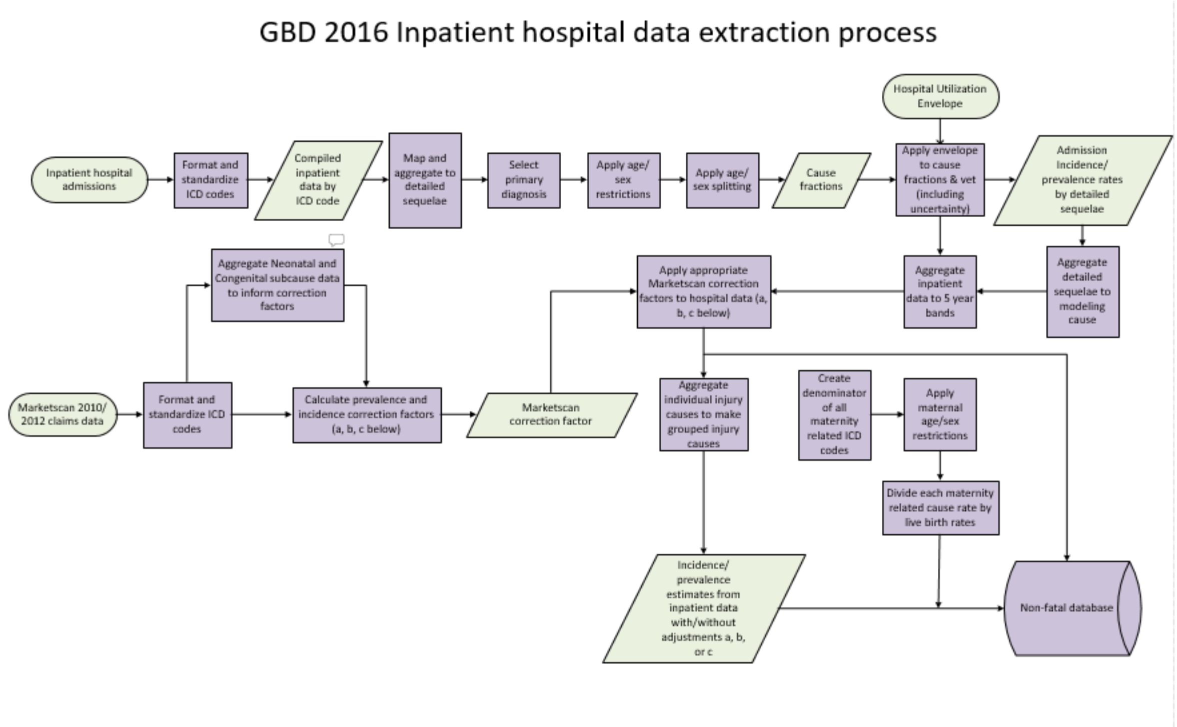

Outpatient

Outpatient encounter data were available from the US and Sweden for 68 location-years. In GBD 2016, Brazil and Mexico were included, but we dropped them this year due to lack of reliability (biases related to types of hospitals that were included in the datasets, etc). No changes were made in the processing of outpatient data from GBD 2015, aside from updates to the ICD mappings to GBD cause.

Similar to the inpatient hospital data, a scalar was calculated using Marketscan claims data to adjust for multiple visits per individual within one year (for long-term conditions), and within a cause-specific duration (for short-term causes). However, for the outpatient correction factor, we kept only 3 outpatient facility types (office, outpatient hospital, and outpatient NEC).

Table: Facility types used in outpatient Marketscan data

Outpatient Facility Name	Used in GBD 2015 for Incidence	Used in GBD 2016 for Incidence	Used in Outpatient Correction Factor
Office	no	yes	yes
Outpatient Hospital	yes	yes	yes
Independent Laboratory	yes	no	no
Emergency Room - Hospital	yes	yes	no
Patient Home	yes	yes	no
Inpatient Hospital	no	yes	no
Ambulatory Surgical Center	no	yes	no
End-Stage Renal Disease Facil	no	yes	no
Other Unlisted Facility	no	yes	no
Outpatient (NEC)	no	yes	yes
Skilled Nursing Facility	no	yes	no
Urgent Care Facility	no	yes	no
Ambulance (land)	no	yes	no
Independent Clinic	no	yes	no
Comprehensive Outpt Rehab Fac	no	yes	no
Nursing Facility	no	yes	no
Hospice	no	yes	no
Pharmacy (1)	no	no	no
Rural Health Clinic	no	yes	no
State/Local Public Health Clin	no	yes	no
Birthing Center	no	yes	no
Community Mental Health Center	no	yes	no
Federally Qualified Health Ctr	no	yes	no
Psych Facility Partial Hosp	no	yes	no
Residential Subst Abuse Facil	no	yes	no
Mass Immunization Center	no	yes	no
Custodial Care Facility	no	yes	no
Comprehensive Inpt Rehab Fac	no	yes	no
Psych Residential Treatmnt Ctr	no	yes	no
Inpatient Long-Term Care (NEC)	no	yes	no
Mobile Unit	no	yes	no
Assisted Living Facility	no	yes	no
School	no	yes	no
Inpatient Psychiatric Facility	no	yes	no
Walk-in Retail Health Clinic	no	yes	no
Military Treatment Facility	no	yes	no
Pharmacy (2)	no	no	no

Other Inpatient Care (NEC)	no	yes	no
Ambulance (air or water)	no	yes	no
Non-resident Subst Abuse Facil	no	yes	no
Intermed Care/Mental Retarded	no	yes	no
Group Home	no	yes	no
Adult Living Care Facility	no	yes	no
Temporary Lodging	no	yes	no
Homeless Shelter	no	yes	no
MISSING	no	yes	no

Table: Durations of causes

Duration in Days	GBD Cause
28	diarrheal diseases, clostridium difficile, pelvic inflammatory disease, acute otitis media, myocardial infarction due to ischemic heart disease, first ever acute hemorrhagic stroke, first ever acute ischemic stroke, upper respiratory infections, peptic ulcer disease, symptomatic episodes, gastritis and duodenitis, symptomatic episodes, appendicitis, vascular intestinal disorders, paralytic ileus and intestinal obstruction, gallbladder and biliary diseases, symptomatic episodes, pancreatitis cases, interstitial nephritis and urinary tract infections, acute urolithiasis, acute myocarditis, pelvic inflammatory disease due to gonococcal infection, cerebrovascular disease acute, pelvic inflammatory disease due to chlamydial infection
30	influenza
60	acute other sense organ diseases, lower respiratory infections
90	typhoid fever, paratyphoid fever, abscess and other bacterial skin diseases, other meningitis -- viral, gonococcal infection, trichomoniasis infection, adverse effects of medical treatment, foreign body in eyes, acute glomerulonephritis, cellulitis, impetigo, foreign body in other body part, pulmonary aspiration and foreign body in airway, other transport injuries, cyclist road injuries, motor vehicle road injuries, motorcyclist road injuries, other road injuries, pedestrian road injuries by road vehicle, poisonings, falls, fire, heat, and hot substances, environmental heat and cold exposure , other unintentional injuries, venomous animal contact, non-venomous animal contact, exposure to forces of nature, drowning, unintentional suffocation, other exposure to mechanical forces, unintentional firearm injuries, self-harm by other specified means, self-harm by firearm, sexual violence, assault by other means, assault by firearm, assault by sharp object, executions and police conflict, conflict and terrorism, typhoid and paratyphoid fever
120	acute encephalitis
180	endocarditis, early syphilis infection, chlamydial infection, maternal abortive outcome
365	diphtheria remission, whooping cough, meningitis nonfatal overall, mild impairment due to neonatal tetanus, varicella seroprevalence, measles, visceral leishmaniasis, cutaneous and mucocutaneous leishmaniasis, symptomatic cystic echinococcosis, disfigurement due to basal cell carcinoma, cutaneous squamous

	cell carcinoma, Guillain-Barré syndrome, ectopic pregnancy incidence ratio, maternal hemorrhage, hypertensive disorders of pregnancy, other maternal infections, obstructed labor, acute event, puerperal sepsis, decubitus ulcer, meningococcal meningitis incidence proportion, other meningitis incidence proportion, H influenza type b meningitis incidence proportion, pneumococcal meningitis incidence proportion, severe pre-eclampsia, eclampsia

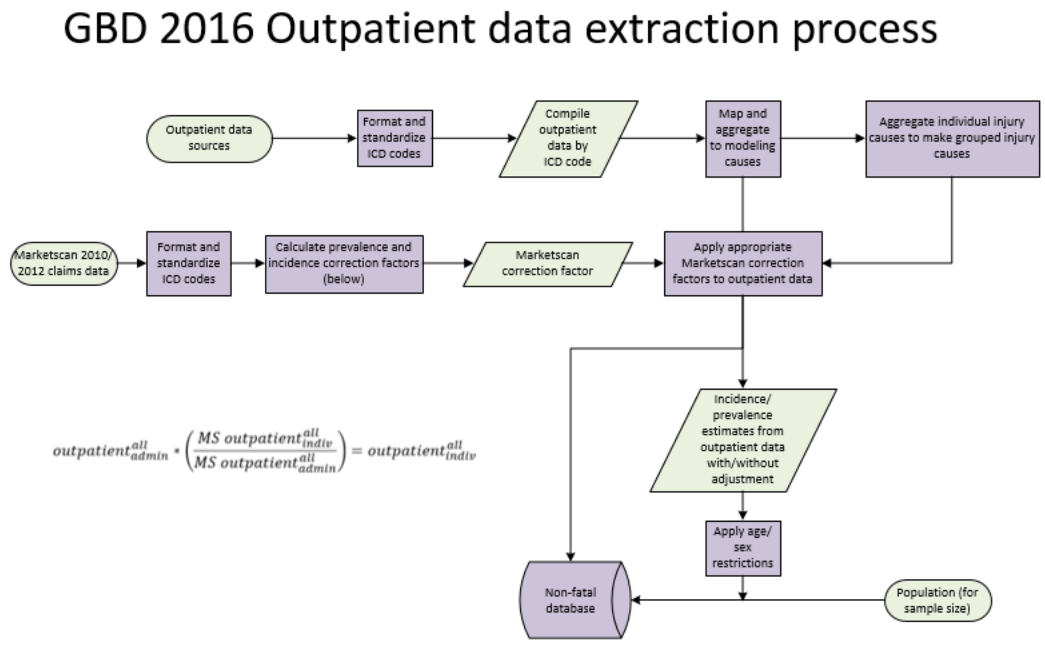

2.1.6 Case notifications

Case notifications, active screening, intervention coverage studies, and surveillance contributed to estimates of infectious diseases. If available, we extracted data from survey and administrative microdata; otherwise, data were extracted from published literature and reports. For many infectious diseases and neglected tropical diseases (NTDs), we made use of cases notified by countries to the World Health Organization (WHO) and other global monitoring entities. The causes for which we use WHO case notification data included tuberculosis (TB), measles, yellow fever, rabies, dengue, cholera, whooping cough, human African trypanosomiasis (HAT), meningitis, all sexually transmitted infections (STIs), and other infectious and NTDs, such as Ebola.

2.2 Data adjustment

In addition to the corrections applied to claims and hospital data, a number of other adjustments were applied to extracted nonfatal sources in order to make the data more consistent and suitable for modelling. In this second step of nonfatal estimation, commonly applied adjustments included age-sex splitting, adding study-level covariates, bias correction, adjustments for underreporting of notification data, and computing expected values of excess mortality.

Age-sex splitting was commonly applied to literature data reported by age or sex but not by age and sex. For GBD2016, we split all data reported in age groups with a width greater than 20 years, using age patterns from available survey microdata or regional patterns derived from an initial run of main modelling tool, DisMod-MR 2.1. We relied on the meta-regression component of DisMod-MR 2.1 for many of the bias correction of data for variations in study attributes such as case definitions and measurement method. DisMod-MR 2.1 calculates a single adjustment that is applied regardless of age, sex, or location. If enough data were available to differentiate these adjustments by age, sex, or location, or if detailed survey data were available to make more precise adjustments between different thresholds on a biochemical measure, we applied bias corrections to the data before entry into DisMod-MR 2.1. For instance, we crosswalked between 15 different case definitions with different thresholds of fasting plasma glucose or glycated hemoglobin levels for diabetes mellitus based on available survey data with individual records of the actual measurements. In another example, we corrected data reporting on one-year prevalence instead of point prevalence of alcohol dependence by age using studies reporting on both measures, as the average duration of alcohol dependence is greater in middle-aged and older individuals compared to young adults. The correction of notification data for underreporting relied on studies that had examined the gap between true incidence and notified cases.

In GBD 2016, we estimated expected values of excess mortality from prevalence or incidence and cause-specific mortality rate (CSMR) data for every cause for which deaths were estimated with the exception of a few causes with very low mortality rates such as uterine fibroids. We matched every prevalence data point (or incidence data for short-duration conditions) with the CSMR value corresponding to the age range, sex, year, and location of the data point. We restricted this to data points reporting age-groups spanning 20 years or less. The ratio of CSMR to prevalence (or incidence times a short duration) is conceptually equivalent to an excess mortality rate. To reflect a gradient in excess mortality, we added in all relevant models the log of lag distributed income (LDI) or the health access and quality index (HAQI) as a country covariate for excess mortality, with a strong prior that as LDI increases, excess mortality declines.

2.3 DisMod-MR 2.1 Estimation

a. Estimation of sequelae and causes

The most extensively used estimation method was the Bayesian meta-regression method DisMod-MR 2.1. For some causes such as HIV/AIDS or hepatitis B and C, disease-specific natural history models have been used where the underlying three state model in DisMod-MR 2.1 (susceptible, cases, dead) is insufficient to capture the complexity of a disease process. For some diseases with a range of sequelae differentiated

by severity, such as chronic obstructive pulmonary disease (COPD) or diabetes mellitus, DisMod-MR 2.1 was used to meta-analyze the data on overall prevalence with separate DisMod-MR 2.1 models of the proportions of cases with different severity levels or sequelae. Likewise, DisMod-MR 2.1 was used to meta-analyze data on the proportions of liver cancer and cirrhosis due to underlying etiologies such as hepatitis B, hepatitis C, and alcohol use.

b. DisMod-MR 2.1 description

Until GBD 2010, nonfatal estimates in burden of disease assessments were based on a single data source on prevalence, incidence, remission or a mortality risk selected by the researcher as most relevant to a particular location and time. For GBD 2010, we set a more ambitious goal: to evaluate all available information on a disease that passed a minimum quality standard. That required a different analytical tool that would be able to pool disparate information presented in varying age groupings and from data sources using different methods. The DisMod-MR 1.0 tool used in GBD 2010 evaluated and pooled all available data, adjusted data for systematic bias associated with methods that varied from the reference and produced estimates by world regions with uncertainty intervals using Bayesian statistical methods. For GBD 2013, the improved DisMod-MR 2.0 had increased computational speed allowing computations that were consistent between all disease parameters at the country rather than region level. The hundred-fold increase in speed of DisMod-MR 2.0 was partly due to a more efficient rewrite of the code in C++ but also by changing to a model specification using log rates rather than a negative binomial model used in DisMod-MR 1.0. In cross-validation tests, the log rates specification worked as well or better than the negative binomial specification.[3] For GBD2015, we rewrote the 'wrapper' code that organises the flow of data and settings at each level of the analytical cascade. The sequence of estimation occurs at five levels: global, super-region, region, country and, where applicable, subnational location. The super-region priors are generated at the global level with mixed-effects, nonlinear regression using all available data; the super-region fit, in turn, informs the region fit, and so on down the cascade. The wrapper gives analysts the choice to branch the cascade in terms of time and sex at different levels depending on data density. The default used in most models is to branch by sex after the global fit but to retain all years of data until the lowest level in the cascade. Appendix Figure 2 below summarizes the Dismod-MR process.

In updating the 'wrapper,' we consolidated the code base into a single language, Python, to make the code more transparent and efficient and to better deal with subnational estimation. The computational engine is limited to three levels of random effects; we differentiate estimates at the super-region, region and country level. In GBD 2013, the subnational units of China, the UK and Mexico were treated as 'countries' such that a random effect was estimated for every location with contributing data. However, the lack of a hierarchy between country and subnational units meant that the fit to country data contributed as much to the estimation of a subnational unit as the fits for all other countries in the region. We found inconsistency between the country fit and the aggregation of subnational estimates when the country's epidemiology varied from the average of the region. Adding an additional level of random effects required a prohibitively comprehensive rewrite of the underlying DisMod-MR engine. Instead, we added a fifth layer to the cascade, with subnational estimation informed by the country fit and country covariates, plus an adjustment based on the average of the residuals between the

subnational location's available data and its prior. This mimicked the impact of a random effect on estimates between subnationals.

In GBD 2015, we also improved how country covariates differentiate nonfatal estimates for diseases with sparse data. The coefficients for country covariates were re-estimated at each level of the cascade. For a given location, country coefficients were calculated using both data and prior information available for that location. In the absence of data, the coefficient of its parent location was used, in order to utilize the predictive power of our covariates in data sparse situations.

For GBD 2016, the computational engine (DisMod-MR 2.1) remained substantively unchanged from GBD 2015. We changed the prediction year set to generate fits for the years 1990, 1995, 2000, 2005, 2010, and 2016. We updated the age prediction sets to include age groups 80-84, 85-89, 90-94, and 95+, to comply with changes across all functional areas of the GBD. We also expanded the set of locations where subnational units are modeled; the set now includes: Brazil, China, England, India, Indonesia, Japan, Kenya, Mexico, Saudi Arabia, South Africa, Sweden, and the United States.

c. DisMod-MR 2.1 likelihood estimation

Analysts have the choice of using a Gaussian, log-Gaussian, Laplace or Log-Laplace likelihood function in DisMod-MR 2.1. The default log-Gaussian equation for the data likelihood is:

$$-log\left[p\left(y_j|\Phi\right)\right] = \log\left(\sqrt{2\pi}\right) + \log\left(\delta_j + s_j\right) + \frac{1}{2}\left(\frac{\log\left(a_j + \eta_j\right) - \log\left(m_j + \eta_j\right)}{\delta_j + s_j}\right)^2$$

where, y_j is a 'measurement value' (i.e., data point); Φ denotes all model random variables; η_j is the offset value, eta, for a particular 'integrand' (prevalence, incidence, remission, excess mortality rate, with-condition mortality rate, cause-specific mortality rate, relative risk or standardized mortality ratio) and a_j is the adjusted measurement for data point j, defined by:

$$a_j = e^{(-u_j - c_j)} y_j$$

where u_j is the total 'area effect' (i.e., the sum of the random effects at three levels of the cascade: super-region, region and country) and c_j is the total covariate effect (i.e., the mean combined fixed effects for sex, study level and country level covariates), defined by:

$$c_j = \sum_{k=0}^{K[I(j)]-1} \beta_{I(j),k} \hat{X}_{k,j}$$

with standard deviation

$$s_j = \sum_{l=0}^{L[I(j)]-1} \zeta_{I(j),l} \hat{Z}_{k,j}$$

where k denotes the mean value of each data point in relation to a covariate (also called x-covariate); I(j) denotes a data point for a particular integrand, j; $\beta_{I(j),k}$ is the multiplier of the kth x-covariate for the ith integrand; $\hat{X}_{k,j}$ is the covariate value corresponding to the data point j for covariate k; l denotes the standard deviation of each data point in relation to a covariate (also called z-covariate); $\zeta_{I(j),k}$ is the multiplier of the lth z-covariate for the ith integrand; and δ_j is the standard deviation for adjusted

measurement j, defined by:

$$\delta_j = log\left[y_j + e^{(-u_j - c_j)}\eta_j + c_j\right] - log\left[y_j + e^{(-u_j-c_j)}\eta_j\right]$$

Where m_j denotes the model for the jth measurement, not counting effects or measurement noise and defined by:

$$m_j = \frac{1}{B(j)-A(j)}\int_{A(j)}^{B(j)} I_j(\mathrm{a})\,\mathrm{da}$$

where A(j) is the lower bound of the age range for a data point; B(j) is the upper bound of the age range for a data point; and I_j denotes the function of age corresponding to the integrand for data point j.

2.4 Impairment and Underlying Cause Estimation

For GBD 2016, as in GBD 2015, we estimated the country-age-sex-year prevalence of nine impairments – step 4 of Appendix Figure 1a. Impairments in GBD are conditions or specific domains of functional health loss which are spread across many GBD causes as sequelae and for which there are better data to estimate the occurrence of the overall impairment than for each sequela based on the underlying cause. These impairments included: anaemia, epilepsy, hearing loss, heart failure, developmental intellectual disability, infertility, vision loss, Guillain-Barré syndrome, and pelvic inflammatory disease. Overall impairment prevalence was estimated using DisMod-MR 2.1. We constrained cause-specific estimates of impairments, as in the 19 causes of blindness, to sum to the total prevalence estimated for that impairment. Anaemia, epilepsy, hearing loss, heart failure, and developmental intellectual disability were estimated at different levels of severity. Estimates were made separately for primary infertility (those unable to conceive), secondary infertility (those having trouble conceiving again), and whether the impairment affected men and/or women. In the case of epilepsy, we determined the proportions with idiopathic and secondary epilepsy as well as the proportions with severe and less severe epilepsy using mixed effects regressions. The sparse data for the proportion of seizure-free, treated epilepsy were pooled in a random effects meta-analysis. DisMod-MR 2.1 models produced country-, age-, sex-, and year-specific severity levels of hearing loss and vision loss. Due to limited information on the severity levels of developmental intellectual disability, we assumed a similar distribution of severity globally, based on random effects meta-analysis of IQ-specific data for the overall impairment. This was supplemented by cause-specific severity distributions for chromosomal causes and iodine deficiency; the severity of developmental intellectual disability included in the long-term sequelae of causes including neonatal disorders, meningitis, encephalitis, neonatal tetanus, and malaria was estimated in combined health states of multiple impairments such as motor impairment, blindness, and/or seizures.[14] For GBD 2015, we changed the name of the intellectual disability impairment to specify that estimates reflect cases arising during the developmental period which we have defined as ages below 20. The severity of heart failure was derived from our Medical Expenditure Panel Surveys (MEPS) analysis and therefore was not specific for country, year, age, or sex.

A detailed description of the methods of each impairment can be found at the end of Section 3 of this appendix.

2.4.1 Impairment squeeze

For impairments like epilepsy, developmental intellectual disability, and blindness, mentioned above in Step 4, we often have better information regarding the total prevalence of the impairment rather than the prevalence of said impairment due to its various causes. For example, we have more data and a better idea of the total number of blind individuals (which we refer to herein as the blindness "envelope") in the world than we do for the number of individuals who are blind due to a specific cause like retinopathy of prematurity or cataract. We achieve this consistency by either "squeezing" or inflating the individual sequela prevalence values so that their sums fit into each appropriate envelope. Blindness, epilepsy and/or developmental intellectual disability appear in various combinations with motor impairment levels as sequelae for a number of neonatal disorders and infectious diseases like malaria and neonatal tetanus ("Moderate motor impairment with blindness and epilepsy due to neonatal tetanus," for example). This present an extra challenge as any squeeze or inflation of one of the impairments making up a sequela will affect the others. We set some rules on how to do these adjustments sequentially. First, when the envelope of an impairment is smaller than the sum of all contributing causes, we redistribute the 'excess' prevalent cases of combined impairment sequelae onto the sequelae that only have motor impairment (at mild, moderate or severe level) within the same cause grouping. Second, we apply the adjustments in a particular order such that we always fit at least one of the envelopes exactly where the other one or two envelopes may be exceeded by some amount. We first enforce a fit to the developmental intellectual disability impairment envelope, then epilepsy and, lastly, blindness. Thus, the developmental intellectual disability envelope will always match exactly, whereas the epilepsy and blindness envelopes may occasionally be exceeded on a draw-by-draw basis.

2.5 Severity Distribution

Sequelae were defined in terms of severity for 199 causes at Level 4 of the hierarchy (Appendix Table 3). We generally followed the same approach for estimating the distribution of severity as in GBD 2015. For Zika, we included sequelae for those with symptomatic acute infection, a small proportion with Guillain-Barré syndrome and the number of neonates with congenital Zika as reported to Pan American Health Organization (PAHO). For sexual violence, we estimated concurrent physical injuries and the more immediate psychological outcomes following sexual violence. For the added causes that were split from broader cause categories, the differentiation between drug-sensitive and drug-resistant tuberculosis, the creation of other leukemia, alcoholic and other cardiomyopathy, and self-harm by firearm or other means each follow the same pattern of assigning sequelae as for their parent causes. In cases in which severity was related to a particular impairment, such as mild, moderate, and severe heart failure due to ischemic heart disease, the analysis was driven by impairment estimation methods. Severity levels for conditions such as chronic kidney disease and COPD were modelled using DisMod-MR 2.1, while we performed meta-analyses to estimate the allocation of severity for causes such as rheumatoid arthritis, dementia, and multiple sclerosis.

For many causes we had inadequate data on severity from surveys or the epidemiological literature. For those diseases, we made use of three population surveys: the US Medical Expenditure Panel Survey (MEPS) 2000–2014, the [US] National Epidemiological Survey on Alcohol and Related Conditions (NESARC)

2000–2001 and 2004–2005, and the Australian National Survey of Mental Health and Wellbeing of Adults (NSMHWB) 1997.[20–22] Each dataset contained individual-level measurements of functional health status using the SF-12 Health Survey as well as diagnostic information on the conditions affecting each individual.

In order to use the data collected using SF-12 for measuring the distribution of severity, the individual SF-12 summary scores were mapped to an equivalent disability weight. A convenience sample of respondents was asked to complete SF-12 for the hypothetical individual living in a health state described using a selection of 60 of the 235 health states with their lay descriptions from the GBD Disability Weights (DW) surveys, reflecting the full range of severity. Each of these health states has a measured disability weight associated with it on a zero to one scale. In total, we collected 1,980 usable responses. To deal with heterogeneity in responses, we excluded from the statistical analysis responses that were more than two median absolute deviations from the median for each health state. After correcting for outliers, the rank order correlation between SF-12 scores for the hypothetical individuals in each health state characterized by the lay description with the measured disability weight was -0.815. The health states served as random effect groups, such that the composite score would be equal to the intercept plus the random effect estimated for that health state, or

$$DW_i = \alpha + U_{health\ state}.$$

The final relationship between SF-12 score and disability weight is shown below:

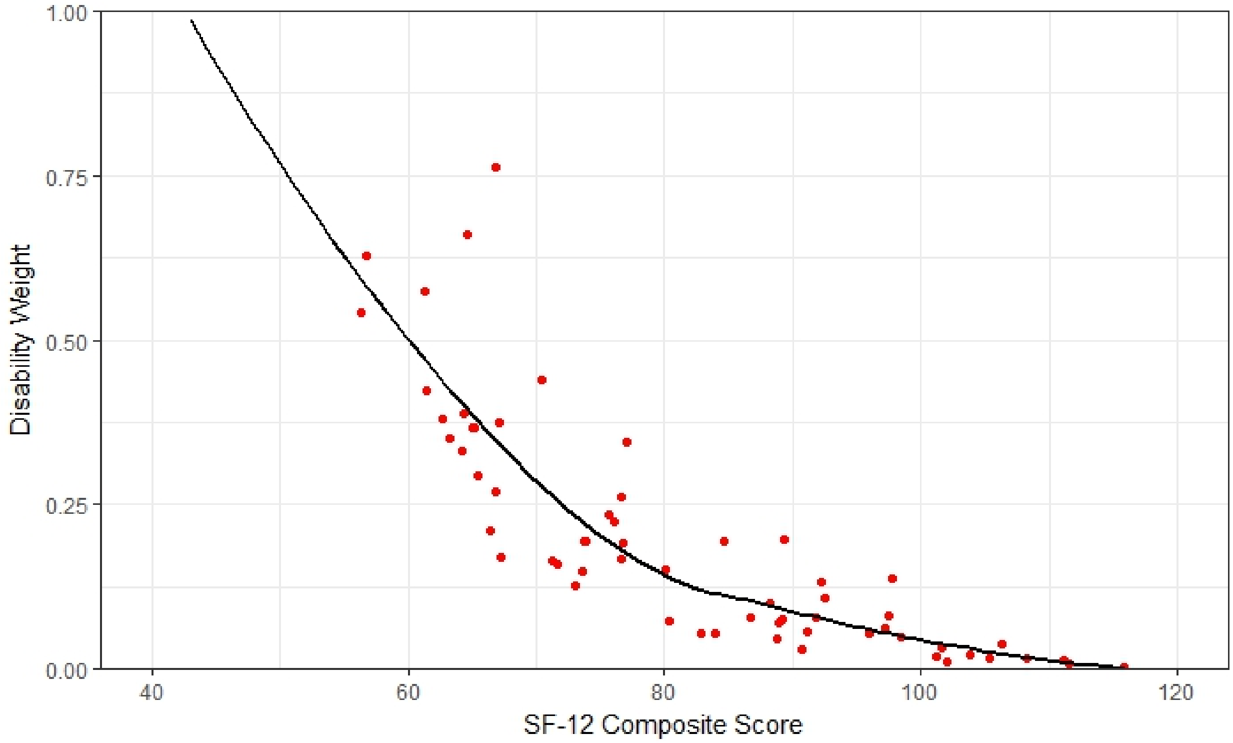

To generate a smooth mapping from SF-12 combined scores to the GBD disability weight space, we used LOESS regression on the random effects for each health state. Because disability weights are defined in the range from 0 to 1, we truncated the function at a combined SF-12 score of 116.36 (any combined score above this level was set to 0) and truncated the function at 42.7 so that any combined score less than that value was set to 1. All SF-12 survey data were thus transformed into disability weight space.

The second stage of the analysis was to build models predicting the transformed SF-12 scores as a function of the number of conditions suffered by each individual. First, variable selection was performed using LASSO regression to penalize the regression coefficients of highly correlated conditions. The tuning parameter, λ, controls the strength of the least-squares penalty. When λ=0, LASSO regression returns the same results as ordinary least-squares regression. Higher values of λ impose a stronger penalty and constrain a greater number of model parameters to 0. A 10-fold cross-validation was used to find the value of the λ that minimised the mean cross-validated error. This process resulted in a λ value of 0.0013 and eliminated 10 conditions from the analysis. Transformed SF-12 scores into the disability weight scale for the remaining 190 conditions were then modeled for each measure *m* of each individual *i* over *n* total conditions in the survey, as follows:

$$logit(DW)_{im} = \beta_0 + \beta_1 Condition1_{im} + \cdots + \beta_n Condition\, n_{im}$$

This equation effectively assumes that comorbid conditions act to change SF-12 scores in a multiplicative fashion rather than an additive fashion.

To estimate the comorbidity-corrected effect of each condition (i.e., in isolation) on total disability, we compared the predicted disability weight without the condition of interest ("counterfactual DW") with the predicted disability weight including the condition of interest. Following the multiplicative comorbidity equation, the joint effect can be written:

$$Condition\ specificDW = 1 - \frac{1 - predictedDW_m}{1 - counterfactualDW_m}$$

The mean of this condition-specific effect over all observations is the population marginal effect of a condition.

Using the model above, we estimate a counterfactual disability weight – the total individual disability weight excluding the effect of the condition of interest. We compared the observed distribution of functional health status with this counterfactual distribution to determine the marginal effect of the condition of interest. In other words, we estimate the health state for each individual and for each condition as the cumulative individual weight minus the effects of all comorbid conditions.

$$Health\ state\ DW = 1 - \frac{1 - individual\ cumulativeDW_m}{1 - counterfactualDW_m}$$

The estimation strategy for health state-specific severity distributions where there are multiple severity categories involved binning individuals' weights into severity cutoffs (e.g., mild, moderate, and severe) for which disability weights were derived. These bins were defined using results from the GBD Disability Weights Studies[23] for conditions which had multiple health states defined. Cutoffs were taken as the midpoints between levels of health state and cases distributed into severity bins accordingly. Cases were considered asymptomatic if the counterfactual weight was equal to or exceeded the individual cumulative weight.

2.6 Disability weights

To compute YLDs for a particular health outcome in a given population, the number of people living with that outcome was multiplied by a disability weight that represents the magnitude of health loss associated with the outcome. Disability weights are measured on a scale from 0 to 1, with 0 implying a state that is equivalent to full health and 1 a state equivalent to death.

Disability weights used in GBD studies prior to GBD 2010 have been criticized for the method used (person trade-off), the small elite panel of international public health experts who determined the weights and the lack of consistency over time as the GBD cause list expanded and additional disability weights from a study in the Netherlands[24] were added or others derived by ad-hoc methods.

GBD 2010 Disability Weights Measurement Study

For GBD 2010, a primary data collection effort focused on measuring health loss rather than welfare loss using a standardized approach of simple comparison questions directed to the general public across diverse communities.

Household surveys were conducted between Oct 28, 2009 and June 23, 2010 in five countries (Bangladesh, Indonesia, Peru, Tanzania and the USA) selected to provide diversity across culture, language and socioeconomic status.

Personal face-to-face computer-assisted interviews were conducted for all household surveys with the exception of the survey in the USA which was conducted as computer-assisted telephone interviews. Households were randomly selected using a multistage stratified sampling design where the probability of selection was proportional to the population size. In all cases, samples were designed to be representative for a given geographical area with national representation in the case of the USA.

For every contacted household, an adult respondent aged 18 years or older was randomly selected by the survey programme using the Kish approach. For face-to-face interviews, up to three visits were made to selected households to establish contact. When a respondent was identified, up to three return visits were made in order to do the survey at a time when the respondent was available. For the US telephone surveys, repeat calls were made up to seven times.

A web based survey was posted at a dedicated URL between July 26, 2010 and May 16, 2011. The survey was initially available in English with subsequent availability in Spanish and Mandarin. Recruitment of respondents occurred through several channels, such as news items and editorials in scientific journals,

announcements at scientific meetings, postings on websites of institutions participating in the GBD, social networking and communication mobilization channels as well as direct contact with individuals and groups with known global health interests by tapping into the professional networks of the study investigators and their colleagues. Participants in the web based survey were required to be aged 18 or older. Household surveys obtained oral informed consent from all participants; written informed consent was obtained from participants in the web survey. Ethical review board approval was obtained from each household survey site and at the University of Washington, Seattle, WA.

Standardized survey instruments were developed to obtain comparative assessments of the full array of disease and injury sequelae, parsimoniously captured in 220 unique health states. Lay descriptions of health states formed the basis for all comparisons. These descriptions used simple, non-clinical vocabulary that emphasized the major functional consequences and symptoms associated with each health state. Development of these descriptions involved an iterative process of detailed consultation with experts participating in the GBD 2010 study with the goal of both capturing the most relevant details of each health state while avoiding ambiguity and ensuring consistency. Where possible, health states were grounded in standard clinical classifications systems, for example, the Canadian Cardiovascular Society grading scale was referenced for descriptions of stages of angina,[25] while the New York Heart Association functional classification was referenced for severity of heart failure.[26] Pilot testing indicated that the lay descriptions in face-to-face interviews should not exceed 30 words.

A paired comparison question formed the basis of all surveys. The questions in the survey were framed with the following statement, "A person's health may limit how well parts of his body or mind work. As a result, some people are not able to do all of the things in life that others may do, and some people are more severely limited than others. I am going to ask you a series of questions about different health problems. In each question, I will describe two different people…" Descriptions of two hypothetical people, each with a particular health state, were presented to respondents who were then asked which person they regarded as "the healthier". Health pairs in all surveys were selected by a randomizing computer algorithm. In the five household surveys, paired comparisons were presented for a subset of 108 health states pertaining to chronic conditions. The framing of chronic and acute conditions is different as they were presented as causing life-long or temporary health loss. We chose to only field health states that could be framed as lasting a lifetime in the household surveys as we hypothesized that presenting differently framed comparisons would be difficult to convey in face-to-face interviews. In the web survey we considered this more feasible as respondents could read and refer to the framing of the question for each pair-wise comparison. All 220 health states were thus evaluated in the web survey.

In addition, the web survey included "population health equivalence" (PHE) questions relating to population health and health programmes specifically – such as "Imagine two different health programmes. The first programme prevented 1,000 people from getting an illness that causes rapid death. The second programme prevented 2,000 people from getting an illness that is not fatal but causes lifelong health problems resulting in varying examples of health states at moderate to severe disability. Which programme would you say produced the greater overall health benefits?" This information was used to anchor the results from the pair-wise comparisons on the 0–1 disability weight scale.

GBD 2013 European Disability Weights Measurement Study

The GBD 2010 disability weights were critically dependent on the ways that outcomes were described to survey respondents. Descriptions for health states were designed to balance validity and parsimony and this necessarily meant that some details of different health states had to be omitted. As lay descriptions were developed collaboratively through individual expert groups organised around a particular set of health issues – some amount of variability in language and detail inevitably occurred. Criticisms and suggestions for improvement came from a number of commentators on the GBD 2010 disability weights measurement study.[27–29]

The GBD 2013 Study expanded the list of disease and injury causes and sequelae which were mapped to 235 unique health states. Additional data for the European Disability Weights Measurement Study were collected between September 23, 2013 and November 11, 2013 in Hungary, Italy, the Netherlands and Sweden. The initiation of these surveys was connected to a project sponsored by the European Centre for Disease Prevention and Control (the Burden of Communicable Diseases in Europe project).[30] The four selected countries were chosen to be representative of the four regions of Europe (east, south, middle, and north) in terms of age, sex and education of the respondents. Respondents were recruited from standing internet panels in each country on the basis of quota sampling with reference to age, sex and education in such a way as to maintain population representativeness of these characteristics. Eligible participants were aged 18–65 and were preselected in the case of the Netherlands, where age, sex and education of respondents were already known, or in the case of the other three countries, invited to participate via a web-link and then selected on the basis of their individual characteristics.

The protocol for the European disability weights measurement study followed the protocol that was developed and implemented in the GBD 2010 disability weights measurement study. Lay descriptions for some health states that lacked mention of an important symptom or for which consistency of wording across different levels of severity had been noted were reworded. The European disability weights measurement study included 255 health states, of which 183 were used in the analyses of GBD 2013. Those 183 consisted of 135 of the 220 health states that were included in the European disability weights measurement study with unmodified lay descriptions; 30 from GBD 2010 for which alternative lay descriptions were included. Disability weights were estimated for additional sequelae that were incorporated into GBD 2013 but had not been included in GBD 2010.

Finding high correlation in resulting disability weight values between the country surveys and the web survey, we analyzed the results of all surveys together. We ran probit regression analyses on the answers to the pair-wise comparison questions, with dummies for each health state with a value of 1 for the first state in a pair, –1 for the second of a pair being chosen, and 0 for all states other than the pair being considered. This method formalizes the intuition that if two health states in a pair produce similar health loss, the answers are likely to be evenly split; a pair of health states with very different health loss, will get many more responses favoring one over the other. The statistical methods infer the distances between values attached to different health states based on the frequencies of responses to the paired comparisons. A second analytic step was needed to anchor the resulting estimates onto the 0–1 disability

weights scale. We anchored results from the probit regression analysis onto the 0–1 scale using population health equivalence data from the GBD 2010 web survey using a linear regression of the probit coefficients from the analysis of paired comparisons on the logit-transformed disability weight estimates derived from interval regression of the population health equivalence responses. Using numerical integration, we then estimated mean values for disability weights on the natural 0–1 scale. Uncertainty was estimated by bootstrapping with 1000 samples.

A complete listing of the lay descriptions and values for the 235 health states used in GBD 2016 is provided in Appendix Table 5.

2.7 Comorbidity correction (COMO)

The final stage in the estimation of YLDs is a micro-simulation, which adjusts for comorbidity. We refer to this micro-simulation process as "COMO". For GBD 2016, we estimated the co-occurrence of different diseases by simulating 40,000 individuals in each location-age-sex-year combination as exposed to the independent probability of having any of the sequelae included in GBD 2016 based on disease prevalence. We tested the contribution of dependent and independent comorbidity in the US MEPS data, and found that independent comorbidity was the dominant factor even though there are well-known examples of dependent comorbidity, i.e., clustering of conditions such as diabetes and stroke or anxiety and alcohol use disorders. Age was the main predictor of comorbidity such that age-specific microsimulations accommodated most of the required comorbidity correction.[31]

The two components necessary for the computation of YLDs, prevalence of each disease sequelae and disability weights, are the two inputs into COMO. The prevalence values are primarily produced using DisMod-MR 2.1. The disability weights have been described above.

The micro-simulation, as performed for each age-sex-location-year, can best be represented as a four-step process. First, simulants are exposed to independent probabilities of having each sequela, where the probability is equal to the prevalence estimate. For each simulant, the probability of having a disease sequela is equal to the estimated prevalence from that draw from the uncertainty distribution. Each simulant is determined to have or not have the disease sequelae based on a draw from a binomial distribution. From this simulation, simulants end up having from none to multiple disease sequelae. Second, the disability weight for each simulant is estimated based on the disease sequelae that they have acquired. The formula for the cumulative disability weight for a simulant is one minus the multiplicative sum of one minus each disability weight present:

$$Simulant\ DW_l = 1 - \prod_{k=i}^{j}(1 - DW_k)$$

where the DW_k is the disability weight for the kth disease sequela that the simulant *l* has acquired. Once the simulant disability weight is computed, the disability weight attributable to each sequela for the simulant is calculated using the following formula:

$$ADW_{lk} = \frac{DW_k}{\sum_{k=i}^{k=j} DW_k} * Simulant\ DW_l$$

where ADW_{lk} is the attributable DW for disease sequela *k* in simulant *l*; DW_k is the disability weight for disease sequela *k*, and simulant DW_l is the disability weight for simulant *l* from the combination of all sequelae that they have acquired. This formula apportions the overall simulant disability weight to each condition in proportion to the disability weight of each condition in isolation.

Finally, YLDs per capita in an age-sex-country-year are computed by taking the sum of the attributable disability weights for a disease sequela across simulants.

$$YLD\ Rate_k = \frac{\sum_{l=1}^{n} ADW_{lk}}{n}$$

The actual number of YLDs from disease sequela *k* in an age-sex-location-year is then computed as the YLD rate *k* times the appropriate age-sex-location-year population.

By repeating the simulation process for each age-sex-country-year 1,000 times, the uncertainty in the prevalence of each disease sequela and the disability weight is propagated into the final comorbidity corrected YLD results. We selected 40,000 simulants for each age-sex- location-year group on the basis of simulation testing, which has shown that results were stable for YLDs at this number of simulants even in the younger age groups when prevalence is relatively low. For any given location-year-age-sex group, sequelae with prevalence of less than one in 20,000 were excluded from the microsimulation.

2.8 YLD Computation, Uncertainty & Residual YLDs

For GBD 2016, we computed YLDs by sequela as prevalence multiplied by the disability weight for the health state associated with that sequela. The uncertainty ranges reported around YLDs incorporates uncertainty in prevalence and uncertainty in the disability weight. To do this, we take the 1,000 samples of comorbidity-corrected YLDs and 1,000 samples of the disability weight to generate 1,000 samples of the YLD distribution. We assume no correlation in the uncertainty in prevalence and disability weights. The 95% uncertainty interval is reported as the 25th and 975th values of the distribution. Uncertainty intervals for YLDs at different points in time (1990, 1995, 2000, 2005, 2010, and 2016) for a given disease or sequela are correlated because of the shared uncertainty in the disability weight. For this reason, changes in YLDs over time can be significant even if the uncertainty intervals of the two estimates of YLDs largely overlap as significance is determined by the uncertainty around the prevalence estimates.

Residual YLDs

Despite expanding our list of causes and sequelae in successive GBD iterations, many diseases remain for which we do not explicitly estimate disease prevalence and YLDs. Less common diseases and their sequelae were included in 35 residual categories (Appendix Table 6). For 22 of these residual categories, epidemiological data on incidence or prevalence were available and these were modelled accordingly. For

13 residual categories, epidemiological data on incidence and prevalence were not available but sufficient cause of death data allowed for cause of death estimates. For these residual categories, we estimated YLDs by multiplying the residual YLL estimates by the ratio of YLDs to YLL from the estimates of level 3 causes in the same disease category that were explicitly modelled. This scaling was undertaken for each country-sex-year. This approach made the simplifying assumption that the residual diseases caused disability proportionate to the ratio of disability to mortality in explicitly modelled diseases. We did not include causes with large disability but no or little mortality in estimating these ratios. For example, we estimated the YLDs from other neurological disorders from the YLD to YLL ratios for dementia, multiple sclerosis, and Parkinson's disease, but did not include the YLDs from headaches and epilepsy in the ratio.

2.9 Socio-Demographic Index (SDI) analysis & Epidemiological Transition

a. Development of revised SDI indicator

The Socio-demographic Index (SDI) is a composite indicator of development status constructed for GBD 2015 whose components are strongly correlated with health outcomes. It is the geometric mean of 0 to 1 indices of total fertility rate, mean education for those aged 15 and older, and lag distributed income per capita.

SDI was calculated using the Human Development Index (HDI) methodology, wherein an index value was determined for each of the covariate inputs (log LDI, mean educational attainment over age 15, and TFR). For GBD 2015 these indices were computed on the basis of a relative scale, in which the upper and lower bounds were established by the maximum and minimum observed values, respectively, for each input over the entire estimation period of 1980–2015.
Prompted by the observations that the scales (and by extension SDI) were sensitive to the addition of new subnational locations as GBD becomes more granular and to the length of the time period over which SDI is computed, for GBD 2016 we implemented fixed scales in determining individual indices. Thus, an index score of 0 now represents the minimum level of each covariate input past which selected health outcomes can get no worse. An index score of 1 represents the maximum level of each covariate input past which selected health outcomes cease to improve. As a composite, a location with an SDI of 0 would have a theoretical minimum level of development relevant to these health outcomes, while a location with an SDI of 1 would have a theoretical maximum level of development relevant to these health outcomes.

We selected the minima and maxima of the scales by examining the relationships each of the inputs had with life expectancy at birth and under-5 mortality and identifying points of limiting returns at both high and low values, if they occurred prior to theoretical limits (eg, a TFR of 0). The final scales are summarised in the table below.

Input	Lower bound	Upper bound
TFR	1·5[a]	8
LDI per capita	250 USD (5·52 log USD)[b]	60,000 USD (11·00 log USD)
Mean educational attainment for ages 15 and older	0 years	17 years

[a] The low point of limiting returns for TFR was identified at 1 during GBD 2015; however, incorporating feedback with regard to accounting for a pattern of TFR rebound in highly developed countries, we instead set the lower limit of TFR at 1·5.

[b] The minimum for the LDI scale was originally set at the theoretical limit of 0 USD, as we did not observe an asymptotic relationship between log(LDI) and E_0 or 5q0 at lower values of log(LDI). Empirically, however, we also did not observe an LDI below 350 USD (5·86 log USD) for the estimation period 1970–2016. In log-space, this meant that approximately half of our scale was not being utilised, compressing the observed variation in LDI and diminishing its meaningful contribution to SDI. Accordingly, we set the lower limit on LDI to 250 USD (5·52 log USD) to ensure we were fully utilising the range of the scale to capture its variation across space and time, as is the case with the other two inputs.

Using the limits on the scales described above, we computed the index scores underlying SDI analogously to GBD 2015 as follows:

$$I_{Cly} = {(C_{ly} - C_{low})}\Big/{(C_{high} - C_{low})}$$

Where I_{cly} – the index for covariate C, location l, and year y – is equal to the difference between the value of that covariate in that location-year and the lower bound of the covariate divided by the difference between the upper and lower bounds for that covariate. If the values of input covariates fell outside the upper or lower bounds (eg, LDI per capita greater than 60,000 USD), they were mapped to the respective upper or lower bounds. We also note that the index value for TFR was computed as $1 - I_{TFRly}$, as lower TFRs correspond to higher levels of development, and thus higher index scores. For GBD 2016 we expanded the computation of SDI to 755 national and subnational locations spanning the time period 1970–2016.

The composite Socio-demographic Index is the geometric mean of these three indices for a given location-year. The cutoff values used to determine quintiles for analysis were then computed using country-level estimates of SDI for the year 2016, excluding countries with populations less than 1 million.

Example calculation

Below we present the calculation of SDI for Mexico in the year 2010:

$$TFR = 2.43;\ Mean\ educ\ yrs\ pc = 9.23;\ lnLDI = 9.58$$

$$I_{TFR} = 1 - \frac{2.43 - 1.5}{8 - 1.5} = .855$$

$$I_{Educ} = \frac{9.23 - 0}{17 - 0} = .543$$

$$I_{lnLDI} = \frac{9.58 - 5.52}{11.00 - 5.52} = .741$$

$$SDI = \sqrt[3]{I_{TFR} * I_{Educ} * I_{lnLDI}} = \sqrt[3]{.855 * .543 * .741} = .701$$

b. *Age-sex-specific relationships between SDI and YLD rates*

In order to evaluate the relationship between SDI and morbidity, we fit a Gaussian process regression using a linear prior to the mean function within a stochastic partial differential equation (SPDE) framework.

We first assume the following:

$$\ln(Y_{iasc}) \sim N(\mu_i, \sigma^2)$$

Where $Y_{,iasc}$ is the cause-specific YLD rate for a given level of SDI (*i*), age group (*a*), sex (*s*), and cause (*c*). We then specify a linear prior to the mean $\mu_{i,}$:

$$\mu_i = \alpha + \beta(SDI) + z_i$$

Where

$$z_i \sim GP(0, \Sigma_M)$$

GP refers to a Gaussian processs, and Σ_M refers to the Matern covariance function.

For causes where the relationship between SDI and YLD rates was markedly non-linear (e.g., many neglected tropical diseases), we instead specified a continuous linear piecewise prior to the mean with either one or two knots depending on the nature of the distinct changes in direction in the data.

Using SPDE, we specified additional priors on the range, variance, and precision of the mean function, as well as selected the number of underlying bases. These hyperparameters were chosen empirically and were identical for all age-sex-cause combinations. Values for the selected hyperparameters are displayed in the table below.

Hyperparmeter	Value
Range	0.2
Variance	1
Precision	1×10^{10}
Number of bases	2 (mesh points at 0.3, 0.7)

Regressions were run separately by age-sex-cause, using observed cause-specific YLD rates from all years 1990-2016 to produce predictions per level of SDI from 0 to 1 in increments of .005. We fit models on observations from all countries estimated in GBD and included state and province level estimates in lieu of national estimates for Brazil, China, and India due to their large populations (> 200 million) and small number of state-level units modelled in GBD (BRA – 27, IND – 31, CHN – 33) relative to population. Though the United States and Indonesia also fall under the designation of large-population (> 200 million), we fit models on national-level observations instead of state/province-level observations for these two countries as a result of the undue influence from the relatively large number of state-level units modelled in GBD relative to population (USA – 51, IDN – 34). Country and region dummy variables used in GBD 2015 were no longer included in this analysis. All models were fit using the INLA package in R.

Due to the more reliable estimation at more aggregate levels of cause specificity, we imposed a top-down hierarchical scaling scheme, in which the Level 1 causes were scaled to the predictions for all-cause morbidity, then Level 2 causes were scaled to their scaled Level 1 parents, and so on.

Having a complete set of age specific YLD rates, we were then able to produce a full set of age-standardised rates for every SDI level. In order to produce other age-aggregates of our results, we used the same modelling framework as above. In this case, however, we regarded the logit of the share of population in each age group as the dependent variable to estimate a smoothed relationship between population age-structure and SDI. Predictions for each age group at each level of SDI were rescaled to sum to 100%.

Appendix 2

Epilepsy Impairment

Flowchart

Epilepsy

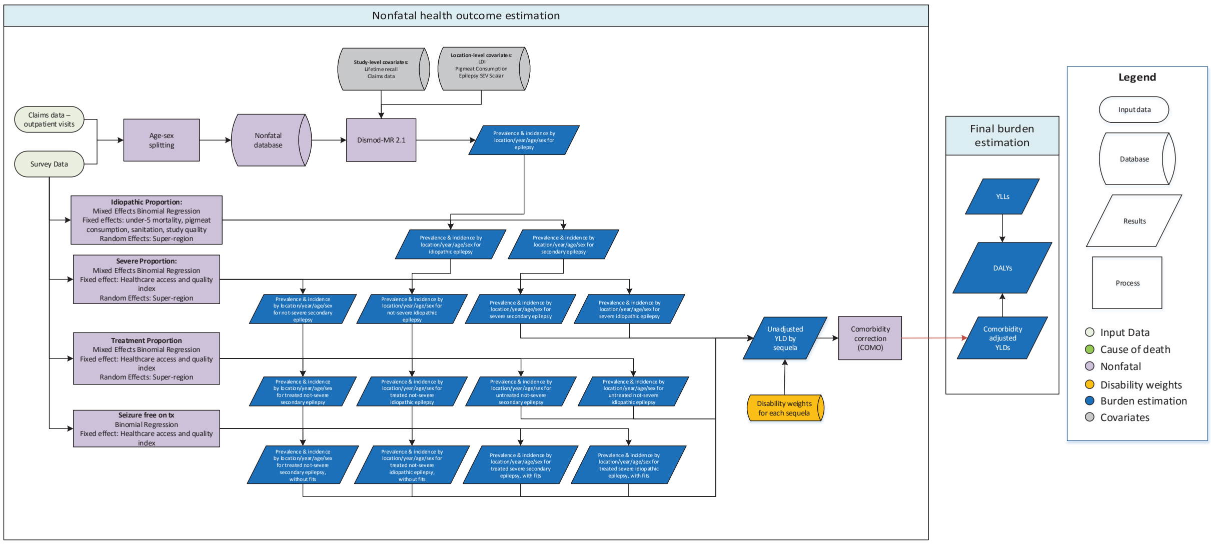

Case definition

Since GBD 2013, we have used the following definitions from the “Guidelines for Epidemiologic Studies on Epilepsy”: 1) Epilepsy: a condition characterized by recurrent (two or more) epileptic seizures, unprovoked by any immediate identified cause, and 2) “Active” epilepsy: a prevalent case of active epilepsy is defined as a person with epilepsy who has had at least one epileptic seizure in the previous five years, regardless of antiepileptic drug (AED) treatment. We also use the following ICD-10 codes for epilepsy: G40 (Neuro, epilepsy, total) and G41 (Neuro, epilepsy, status epilepticus). We defined severe epilepsy as having seizures one or more times per month.

Input data

Model inputs

For GBD 2016, we conducted a systematic review covering 10/1/2014 to 10/7/2016 using the following search string:

("2014/10/01"[PDAT] : "2016"[PDAT]) AND ("epilepsy"[MeSH Terms] OR "epilepsy, partial, motor"[MeSH Terms] OR "epilepsy, benign neonatal"[MeSH Terms] OR "epilepsy, reflex"[MeSH Terms] OR "myoclonic epilepsy, juvenile"[MeSH Terms] OR "epilepsy, frontal lobe"[MeSH Terms] OR "epilepsy, complex partial"[MeSH Terms] OR "epilepsy, post-traumatic"[MeSH Terms] OR "epilepsy, temporal lobe"[MeSH Terms] OR "epilepsy, absence"[MeSH Terms] OR "epilepsy, tonic-clonic"[MeSH Terms] OR "epilepsies, myoclonic"[MeSH Terms] OR "epilepsies, partial"[MeSH Terms] OR epilepsy[Title/Abstract]) AND (incidence[Title/Abstract] OR prevalence[Title/Abstract]) NOT(animals[MeSH] NOT humans[MeSH]).

Of 952 hits, 32 were marked for extraction. Additional data seeking efforts also led to the addition of 12 more sources on epilepsy prevalence in India subnationals. A flow chart documenting the review is displayed below.

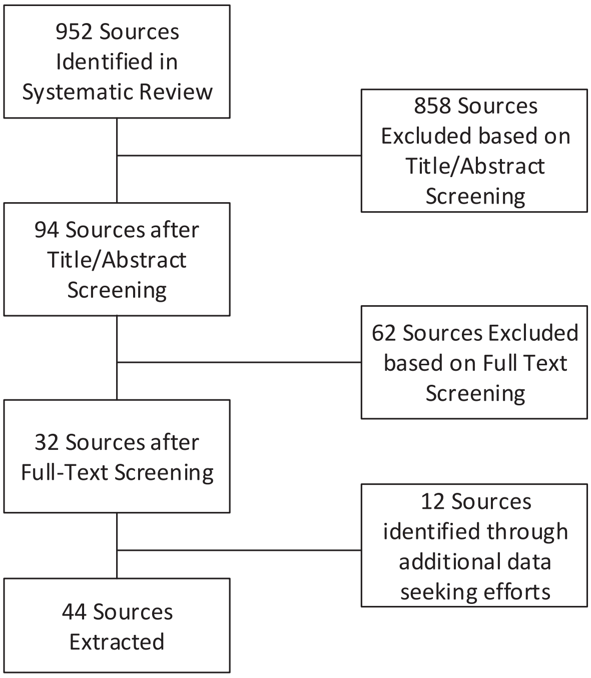

We included representative, population-based surveys that reported of prevalence, incidence, remission rate, excess mortality rate, relative risk of mortality, standardized mortality ratio, or with-condition mortality rate. We excluded studies with no clearly defined sample (eg, among clinic attenders or patient organization members with non-specific or non-representative catchment area). The table below details the model inputs used to estimate the epilepsy impairment.

	Prevalence	Incidence	Mortality risk
Studies	317	81	23
Countries/subnationals	192	51	23
GBD world regions	20	15	10

The inputs for the regressions used to split the epilepsy impairment envelope were also updated for GBD 2016. These regressions are used to determine the proportion of epilepsy that is primary or idiopathic, the proportion of epilepsy that is severe (one or more fits per month), the proportion of epilepsy that is untreated (the treatment gap), and the proportion of treated epilepsy that is treated without fits (no fits reported in the preceding year).

For GBD 2016, a new systematic review was conducted covering 1/1/2006 to 10/17/2016 using the search term:

("2006"[PDAT] : "2016"[PDAT]) AND ("epilepsy"[MeSH Terms] OR "epilepsy, partial, motor"[MeSH Terms] OR "epilepsy, benign neonatal"[MeSH Terms] OR "epilepsy, reflex"[MeSH Terms] OR "myoclonic epilepsy, juvenile"[MeSH Terms] OR "epilepsy, frontal lobe"[MeSH Terms] OR "epilepsy, complex partial"[MeSH Terms] OR "epilepsy, post-traumatic"[MeSH Terms] OR "epilepsy, temporal lobe"[MeSH Terms] OR

"epilepsy, absence"[MeSH Terms] OR "epilepsy, tonic-clonic"[MeSH Terms] OR "epilepsies, myoclonic"[MeSH Terms] OR "epilepsies, partial"[MeSH Terms] OR epilepsy[Title/Abstract]) AND (incidence[Title/Abstract] OR prevalence[Title/Abstract] OR epidemiology[Title/Abstract]) AND (sever*[Title/Abstract] OR treated[Title/Abstract] OR "drug resistant epilepsy"[MeSH] OR "treatment resistant"[Title/Abstract] OR proportion[Title/Abstract] OR "clinical characteristics"[Title/Abstracts] OR "treatment gap"[Title/Abstract]) NOT(animals[MeSH] NOT humans[MeSH])

Of 1,234 hits, 37 were marked for extraction. Additional data seeking efforts also led to the addition of five more sources on the treatment gap in India subnationals. A flow chart documenting the review is displayed below.

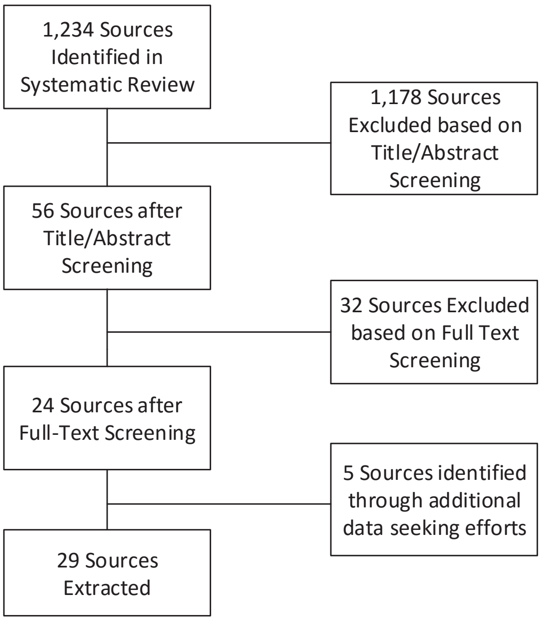

Severity splits & disability weights

The table below illustrates the severity levels, descriptions, and disability weights associated with epilepsy. These are calculated using regressions from literature (ie, frequency of seizures).

Severity level	Lay description	Disability weights (95% CI)
severe (seizures >= once per month)	This person has sudden seizures one or more times each month, with violent muscle contractions and stiffness, loss of consciousness, and loss of urine or bowel control. Between seizures the person has memory loss and difficulty concentrating.	0.552 (0.375–0.71)
less severe (seizures < once per month)	This person has sudden seizures two to five times a year, with	0.263 (0.173–0.367)

	violent muscle contractions and stiffness, loss of consciousness, and loss of urine or bowel control.	
Treated without fits	This person has a chronic disease that requires medication every day and causes some worry but minimal interference with daily activities.	0.049 (0.031–0.072)

Modelling strategy

We modelled the prevalence of epilepsy in two steps: first, we created an epilepsy impairment envelope. Second, we split the envelope into primary (or idiopathic) and secondary epilepsies. Each of these were subdivided into "severe" (on average one or more fits per month) and "non-severe." Non-severe cases were subdivided into "treated" and "un-treated." Finally, "treated" cases were divided into "treated cases with fits" (between one and 11 fits on average in the preceding year) and "treated cases without fits" (no fits reported in the preceding year).

In the first step, we used the DisMod-MR tool for the epilepsy impairment envelope to model a consistent fit between incidence, prevalence, remission, and standardized mortality ratio data while using meta-regression to correct data points with non-reference study quality characteristics. We found no systematic bias for the covariate "non-standard case definition" indicating studies that did not define "active epilepsy" and additionally the covariate was not significant as a "z-cov", which acts as a multiplier applied to the standard error and thus results in these data points being given less weight in the analysis than the "reference" data points. Therefore, we excluded this covariate from the model. We also included data on lifetime prevalence and therefore added a covariate on lifetime prevalence data points. We also included country-level covariates on prevalence for the SEV epilepsy scalar, which summarizes the epilepsy risk exposure level for each country, and pig meat consumption per capita, which is used as a proxy for the level of neurocysticercosis, a common cause of secondary epilepsy. We included cause-specific mortality rate (CSMR) results from the epilepsy mortality model as input data to the DisMod model. Where age-specific prevalence data was available, we calculated excess mortality rate (EMR) from prevalence and CSMR. We included the log of the lag distributed income (LDI) as a covariate on EMR to account for lower mortality in developed countries. We included Bayesian priors on remission to account for the scarcity of remission data. We set bounds on remission from 0 to 0.25 from age 0-60 and 0 to 0.05 from age 61-100. The table below indicates the covariates used in the estimation process, as well as parameters, betas, and exponentiated betas.

Measure	Variable Name	Beta	Exponentiated
prevalence	Recall Lifetime	0.18 (0.15 – 0.22)	1.20 (1.17–1.24)
prevalence	All MarketScan, year 2000	-0.89 (-0.94 – -0.83)	0.41 (0.39–0.43)
prevalence	All MarketScan, year 2010	-0.43 (-0.47 – -0.37)	0.65 (0.62–0.69)

prevalence	All MarketScan, year 2012	-0.35 (-0.41 – -0.30)	0.70 (0.67–0.74)
prevalence	Pig Meat Consumption (kg per capita)	0.0054 (0.00012 – 0.015)	1.01 (1.00–1.02)
Prevalence	Log-transformed age-standardized SEV scalar for epilepsy	0.79 (0.75-0.90)	2.21 (2.12-2.46)
excess mortality rate	LDI (I$ per capita)	-0.55 (-1 – -0.1)	0.58 (1.37–0.90)

In the second step, we used a mixed-effects generalized linear models (binomial family) to predict the proportion of idiopathic epilepsy, the proportion of severe epilepsy, the proportion of treated epilepsy and the proportion of epilepsy that is treated without fits.

Because not all of the data on the proportion of idiopathic epilepsy uses optimal case finding methods (using CT scans or MRI's in addition to EEG's in order to diagnose secondary epilepsy), for GBD 2016 we decided to do add a covariate to crosswalk studies with non-optimal case finding methods to those with adequate methods. The regression for the proportion of epilepsy that is idiopathic therefore has fixed effects on this study quality covariate as well as the under-5 mortality rate, the log of pig meat consumption (per capita), and the proportion of a country with access to proper sanitation, as well as a random effect on super-region.

We used similar models to predict the proportion of severe epilepsy and treatment gap based on the reported proportions extracted from the systematic review. To predict the proportion of severe epilepsy and the treatment gap, we used mixed-effects models with a fixed effect on the log of HAQ Index and a random effect on super-region.

For GBD 2015, a meta-analysis was used to generate two different pooled estimates for proportion of treated epilepsy that is seizure-free in developing and developed countries, as there was not enough data to run a regression. However, for GBD 2016 the expanded dataset allowed for the implementation of a generalized linear model (binomial family) to generate predictions for the proportion of treated epilepsy that is seizure-free. We used a fixed effect on the log of HAQ Index.

We tested a fixed effect on Socio-demographic Index (SDI) and random effects on region and country in different models, but they did not improve the model. We generated 1,000 draws of country-specific estimates for each year between 1980 and 2016 for each of the models. The table below shows the betas from these regressions.

Regression	covariate	beta	SE
Idiopathic	Under-5 Mortality	-65.82	7.50
Idiopathic	Pig meat consumption	-0.12	0.02
Idiopathic	Sanitation	0.45	0.16
Idiopathic	Study Quality	0.88	0.07
Severe	HAQ Index	-2.15	0.24

Treatment Gap	HAQ Index	-3.17	0.18
Treated without fits	HAQ Index	3.65	0.21

Appendix 3

Developmental Intellectual Disability Impairment

Flowchart

Case definition

Developmental intellectual disability is a condition of below-average intelligence or mental ability. Consistent with the American Association on Intellectual and Developmental Disabilities, we define developmental intellectual disability as a condition originating before age 18 (thus, does not include impairment due to stroke, Alzheimer's, etc.). We model the following severities, as measured by intelligence quotient (IQ) tests:

Type of intellectual disability	IQ
Profound	0 to 19
Severe	20 to 34
Moderate	35 to 49
Mild	50 to 69
Borderline	70 to 85

Input data

Model inputs

1) Prevalence data: Prevalence of intellectual disability (IQ <70) came from a systematic review starting 1/1/1990 using the following search string:

(((intellectual disability[MeSH Terms]) AND prevalence[Title/Abstract]) AND ('1990'[Date - Publication] : '3000'[Date - Publication]))

For GBD 2015, this search had 2,115 hits, of which 13 were extracted that had not been previously included in GBD. We included studies that estimate the general population prevalence of intellectual disability. We excluded studies that do not use a case definition based on intelligence quotient (IQ) or investigated non-representative groups, like hospital patients or people of a specific ethnicity.

2) IQ mean and standard deviation: Data for mean and SD of IQ came from three source types:
    a. IQ instruments: We conducted a systematic review for IQ scores from standardized tests designed to measure intelligence. Given the vast number of instruments that are used to measure IQ, we used instrument-specific search strings. In total, our search had 85,000 hits, of which 69 were extracted. We excluded non-general populations or non-school populations (eg, gifted or impaired populations). We extracted the mean and SD of IQ, in addition to whether or not a norming procedure had been applied to the data. Where available, we extracted the raw mean and SD as well as the normalized mean and SD.
    b. International Educational Attainment (IEA) surveys: Scores from standardized mathematics, reading, and/or science exams were extracted from school-based surveys, given that IEA tests measure a closely related concept to IQ in school-age populations.

c.

Survey	Country-Years
Programme for International Student Assessment (PISA)	73 country-years
Progress in International Reading Literacy Study (PIRLS)	212 country-years
Trends in International Mathematics and Science Study (TIMSS)	156 country-years

d. Cognitive function surveys: Scores from cognitive function surveys were used where IQ and IEA data were unavailable, particularly for older populations and infants.

Survey	Country-Years
Survey of Health, Ageing, and Retirement in Europe (SHARE)	131 country-years
WHO Study on Global AGEing and Adult Health (SAGE)	12 country-years
Longitudinal Study of Ageing (LSA)	TBD
Health and Retirement Survey (HRS)	TBD
Bayley Scales of Infant and Toddler Development	TBD; from literature

Severity split/disability weight

Health state	Description	Disability weight
Borderline intellectual functioning	This person is slow in learning at school. As an adult, the person has some difficulty doing complex or unfamiliar tasks but otherwise functions independently.	0.011 (0.005–0.02)
Intellectual disability/mental retardation, mild	This person has low intelligence and is slow in learning at school. As an adult, the person can live independently, but often needs help to raise children and can only work at simple supervised jobs.	0.043 (0.026–0.064)
Intellectual disability/mental retardation, moderate	This person has low intelligence, and is slow in learning to speak and to do even simple tasks. As an adult, the person requires a lot of support to live independently and raise children. The person can only work at the simplest supervised jobs.	0.1 (0.066–0.142)
Intellectual disability/mental retardation, severe	This person has very low intelligence and cannot speak more than a few words, needs constant supervision and help with most daily activities, and can do only the simplest tasks.	0.16 (0.107–0.226)
Intellectual disability/mental retardation, profound	This person has very low intelligence, has almost no language, and does not understand even the most basic requests or instructions. The person requires constant supervision and help for all activities.	0.2 (0.133–0.283)

Modelling strategy

We modelled the prevalence estimates of intellectual disability (ID), both aetiology-specific IDs and idiopathic ID, over multiple steps. For GBD 2016, our modelling strategy changed significantly. We additionally used IQ distribution data to inform the estimates of ID prevalence, as follows.

1) *Estimate Intellectual Disability Prevalence (IQ <70)*
First, we ran a DisMod-MR 2.1 model to estimate the total prevalence of intellectual disability of level IQ <70. We included lag-distributed income and education in the model.

2) *Estimate population mean and SD of Intelligence Quotient*
Second, we crosswalked International Educational Attainment data and cognitive function surveys to one standard IQ measure. We crosswalked by matching country/age/year sources for which we had two or more types of data. Using the IQ data and the data converted to IQ as inputs, we ran a single-parameter, continuous DisMod-MR 2.1 model estimating the mean and SE of IQ using standardized measure. In this model, we included log-transformed LDI (per capita), proportion underweight, and log-transformed education (age-standardized) as country-level covariates in this model. We additionally included as a covariate an indicator of instrument-type. These instrument-types were determined based upon similarity of theoretical constructs and concurrent validity.

3) *Fit parametric distribution to mean and SD of IQ and prevalence of intellectual disability*
We fit a distribution using the mean and SD of IQ, and the prevalence of ID using an ensemble approach. The ensemble approach takes a weighted average of multiple distributions, rather than a single distribution. The distribution is fit via a Method of Moments, where each potential distribution is weighted based upon in-sample fit. This allows us to adapt to different skew over time and geographies, and is a highly generalizable approach that does not specify the underlying distribution.

4) *Severity-specific intellectual disability*
We split the total prevalence of idiopathic ID into four severity levels: mild (IQ 50-69), moderate (IQ 35-49), severe (IQ 20-34), and profound (IQ below 20).

5) *Causal attribution*
We estimated prevalence of each etiology-specific ID by models from the following parent causes. Since we are modelling only developmental intellectual disability, causes such as stroke and Alzheimer's are not included in the causal attribution process.

Neonatal preterm birth complications (<28w, 28-32w, 32-36w)
Neonatal encephalopathy due to birth asphyxia and trauma
Hemolytic disease and other neonatal jaundice
Meningitis (pneumococcal, H influenza type B, meningococcal, other bacterial)
Encephalitis
Malaria
Neonatal tetanus
Iodine deficiency
African trypanosomiasis
Down syndrome
Klinefelter syndrome
Chromosomal unbalanced rearrangements

Neural tube defects
Hypertensive disorders of pregnancy (eclampsia, preeclampsia)
Autism
Fetal alcohol syndrome

For autism, we identified six studies reporting severity of intellectual disability. We conducted a meta-analysis to produce the following severity distribution which we applied to the prevalence of autism to produce severity-specific ID due to autism.

ID severity	Mean	SE
None	0.161	0.034
Borderline	0.161	0.034
Mild	0.375	0.037
Moderate	0.190	0.031
Severe	0.090	0.177
Profound	0.024	0.134

We calculated prevalence of idiopathic ID by subtracting all severity- and aetiology-specific IDs from the severity-specific envelope ID assuming the residuals to represent idiopathic. If the residual was less than 5% of the severity-specific envelope, the prevalence of all aetiology-specific IDs were proportionally squeezed to fit within 95% of the envelope, leaving 5% for idiopathic ID.

As we estimated the prevalence of individual aetiology-specific IDs by models from the respective parent causes, the squeezing may result in a distorted balance of prevalence estimates within their parent causes. With the aim to maintain consistencies of prevalence within each of the parent causes, we added the difference between the original and the squeezed prevalence estimates to the "motor impairment" sequela if the squeezed sequela represented "motor and cognitive impairment." For autism, we obtained the fraction of cases that result in ID from literature (0.29; 0.27–0.30 95% CI) and applied to the subtraction and squeezing processes. We assume all ID cases due to iodine deficiency (cretinism) to result in either severe or profound level, and Klinefelter syndrome cases that result in ID will have either borderline or mild level.

In GBD 2013, all aetiology-specific models were squeezed into the overall (IQ <70) envelope. In 2015, we squeeze each model into its discrete severity envelope. GBD 2016 methods are still in development and will be finalized later this summer.

Appendix 4

Hearing Impairment

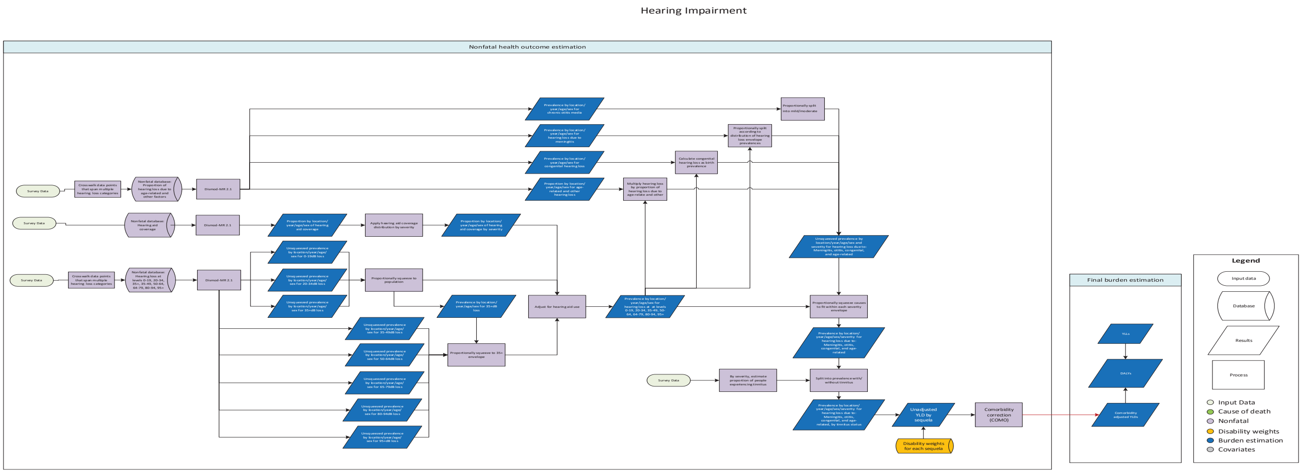

Case definition

For GBD 2016, hearing impairment modeled the following severities of hearing loss:

Severity thresholds of interest for hearing loss	
**Severity**	**Threshold (in decibels)**
None	0–19
Mild	20–34
Moderate	35–49
Moderately severe	50–64
Severe	65–79
Profound	80–94
Complete	95+

We model the following causes of hearing loss: congenital, meningitis, otitis, and age-related and other hearing loss. Hearing loss due to meningitis and otitis are modelled as part of their underlying cause as described in their respective sections. Congenital hearing loss is defined as hearing loss present at birth. Age-related and other hearing loss includes causes not identified as meningitis, otitis, or congenital. This includes presbycusis, the gradual increase in hearing loss over age frequently caused by the natural breakdown of neurons in the inner ear. For all causes, we estimate hearing loss with and without tinnitus, the perception of noise or ringing in the ears.

Input data

Model inputs

For the estimation of the severity-specific envelopes, we used a series of systematic reviews and survey extraction. Data sources up to 2008 were identified by a published systematic review (http://www.ncbi.nlm.nih.gov/pubmed/19444763). A systematic review covering 2008–2013 was conducted with the following search terms:

> (hearing impairment[Title/Abstract] OR deafness[Title/Abstract] OR hearing loss[Title/Abstract]) AND (prevalence[Title/Abstract]) AND ("2008"[PDAT] : "3000"[PDAT]) AND (cross sectional OR survey)

In addition, we extracted hearing loss measurement from the United States National Health and Examination Surveys (NHANES). Self-reported data, from both the literature and surveys, were excluded. This includes censuses in the Integrated Public Use Microdata Series (IPUMS), the WHO Studies on Global Ageing and Adult Health (SAGE), and the WHO Multi-Country Survey Study on Health and Responsiveness (MCSS). Systematic reviews and self-reported survey data (including MCSS, SAGE, and NHANES) were used to estimate hearing aid coverage.

For GBD 2016, we conducted a systematic review on November 30, 2016, using the following search terms:

(hearing impairment[Title/Abstract] OR deafness[Title/Abstract] OR hearing loss[Title/Abstract] OR audiometry[Title/Abstract]) AND (prevalence[Title/Abstract]) AND ("2008/11/26"[PDAT] : "3000"[PDAT]) AND (cross sectional OR survey)

This returned 239 results, of which 17 were accepted.

Where studies reported hearing loss spanning multiple thresholds (eg, 80+, rather than 80-94 and 95+), we crosswalked using ratios predicted by a linear regression on age, using NHANES microdata. Where studies reported severity categories that did not align with GBD thresholds, we crosswalked using NHANES microdata to the nearest GBD severity category, as long as the upper and lower thresholds were not more than 10dB different.

Health states and disability weights

Health state name	Health state description	Disability weight
Hearing loss, mild	This person has great difficulty hearing and understanding another person talking in a noisy place (for example, on an urban street).	0.01 (0.004–0.019)
Hearing loss, mild, with ringing	This person is unable to hear and understand another person talking, even in a quiet place, is unable to take part in a phone conversation. Difficulties with communicating and relating to others cause emotional impact at times (for example worry or depression).	0.021 (0.012–0.036)
Hearing loss, moderate	This person has great difficulty hearing and understanding another person talking in a noisy place (for example, on an urban street), and sometimes has annoying ringing in the ears.	0.027 (0.015–0.042)
Hearing loss, moderate, with ringing	This person is unable to hear and understand another person talking, even in a quiet place, and has great difficulty hearing anything in any other situation. Difficulties with communicating and relating to others often cause worry, depression or loneliness.	0.074 (0.048–0.107)
Hearing loss, moderately severe	(custom DW from hearing loss impairment envelope)	0.092 (0.064–0.129)
Hearing loss, moderately severe, with ringing	(custom DW from hearing loss impairment envelope)	0.167 (0.114–0.231)

Hearing loss, severe	This person is unable to hear and understand another person talking in a noisy place (for example, on an urban street), and has difficulty hearing another person talking even in a quiet place or on the phone.	0.158 (0.104–0.227)
Hearing loss, severe, with ringing	This person is unable to hear and understand another person talking, even in a quiet place, has great difficulty hearing anything in any other situation, Difficulties with communicating and relating to others often cause worry, depression, or loneliness.	0.261 (0.174–0.361)
Hearing loss, profound	This person is unable to hear and understand another person talking in a noisy place, has difficulty hearing another person talking even in a quiet place or on the phone, and has annoying ringing in the ears for 5 minutes at a time, almost every day.	0.204 (0.134–0.288)
Hearing loss, profound, with ringing	This person cannot hear at all in any situation, including even the loudest sounds, and cannot communicate verbally or use a phone. Difficulties with communicating and relating to others often cause worry, depression or loneliness.	0.277 (0.182–0.388)
Hearing loss, complete	This person is unable to hear and understand another person talking, even in a quiet place, and unable to take part in a phone conversation. Difficulties with communicating and relating to others cause emotional impact at times (for example worry or depression).	0.215 (0.143–0.307)
Hearing loss, complete, with ringing	This person cannot hear at all in any situation, including even the loudest sounds, and cannot communicate verbally or use a phone. Difficulties with communicating and relating to others often cause worry, depression or loneliness.	0.316 (0.211–0.436)

Modelling strategy

We modelled the prevalence of hearing loss over five steps. First, we ran three DisMod-MR 2.1 models to estimate the total prevalence estimates of hearing loss: normal hearing (0–19dB), mild hearing loss (20–34dB), and moderate hearing loss and above (35+ dB). We squeezed the prevalence estimates from these DisMod-MR 2.0 models to fit within the entire population of each country. We estimated prevalence of normal hearing for this squeezing purpose only, and hence did not form part of further analysis. Betas and exponentiated values, which can be interpreted as an odds ratio, are shown in the table below for each covariate.

Model	Covariate name	Type	Measure	Beta value	Exponentiated value
Hearing loss impairment at 35+ dB	Socio-demographic Index	Country covariate	Prevalence	-1.451 (-1.984 - -0.486)	0.234 (0.138 - 0.615)
Hearing loss impairment at 95+ dB	Socio-demographic Index	Country covariate	Prevalence	-0.584 (-1.595 - -0.024)	0.557 (0.203 - 0.976)
Hearing aids (proportion of total hearing loss)	LDI (I$ per capita)	Country covariate	Prevalence	0.726 (0.498 - 0.979)	2.066 (1.646 - 2.662)
Hearing loss impairment at 0-19 dB	Socio-demographic Index	Country covariate	Prevalence	0.058 (0.001 - 0.182)	1.059 (1.001 - 1.200)

Second, we ran five additional DisMod-MR 2.1 models for each severity levels of hearing loss above mild: moderate (35–49dB), moderately severe (50–64dB), severe (65–79dB), profound (80–94dB), and complete (95+). We then squeezed the prevalence estimates from these models to fit within the prevalence that were estimated for 35+dB in the first step. By the end of the second step, we had estimated prevalence of six severity levels of hearing loss, including mild (20–34dB). We also ran a DisMod-MR 2.0 model for the coverage of hearing aids, using (logged) lag distributed income (LDI) as a covariate.

Third, we adjusted the prevalence of each severity level by accounting for hearing aids. We assumed the use of hearing aids reduced the severity by one level. Data obtained from a survey in Norway provided detailed information on people with hearing aids, which was used to estimate the proportion of hearing

aids for each severity level. We ran a log-linear regression on age with binary indicator for severity levels. We calculated country-specific hearing aid coverage by multiplying the severity-specific coverage in Norway by the ratio of hearing aid coverage in a given country to that of Norway for each age-sex. We shifted the identified fraction of people in each severity level a level below, except for complete hearing loss, which we assumed was not correctable by hearing aids. This provided the adjusted prevalence of six severity levels of all-cause hearing loss.

Fourth, we estimated the prevalence of hearing loss due to multiple causes: otitis media, congenital, meningitis (pneumococcal, H influenza type B meningitis, meningococcal, and other bacterial), and age-related and other causes not classified elsewhere. For congenital hearing loss, we assumed that all hearing losses occurring at the time of birth are of congenital nature. We assumed that all hearing loss due to otitis media is at the mild or moderate level. We implemented proportional squeezes to scale cause-specific hearing loss prevalence to the total prevalence of each severity level.

Finally, we estimated the percent of people experiencing tinnitus for at least five minutes per day by severity level using data from the NHANES and two datasets from the United Kingdom. We calculated confidence intervals assuming a binomial distribution. We assumed the same distribution of tinnitus across all types of hearing loss.

Appendix 5

Vision Impairment

Flowcharts

Vision Impairment

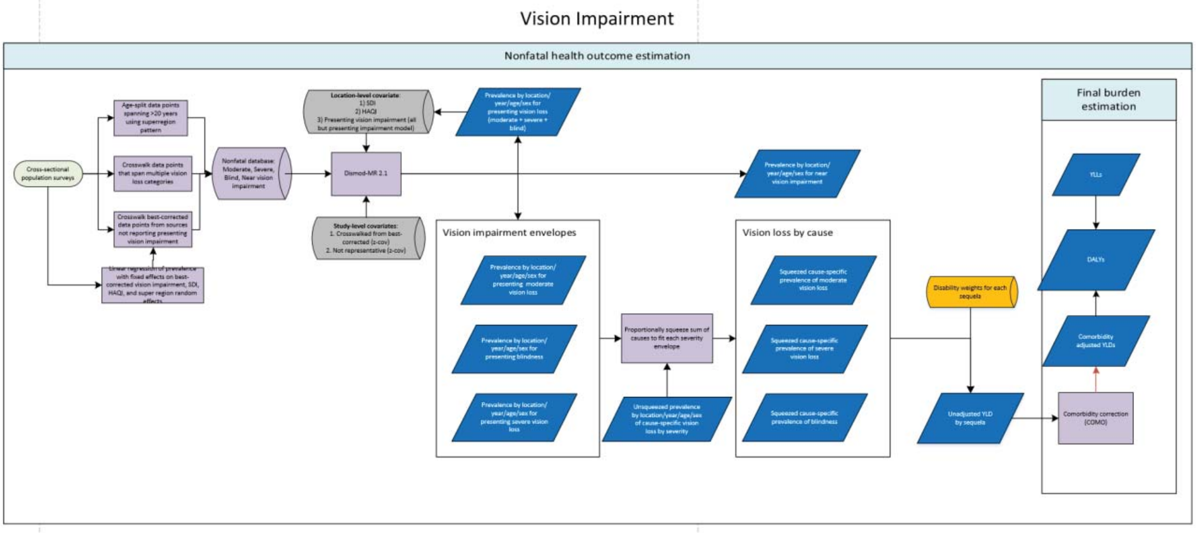

Diabetic Retinopathy

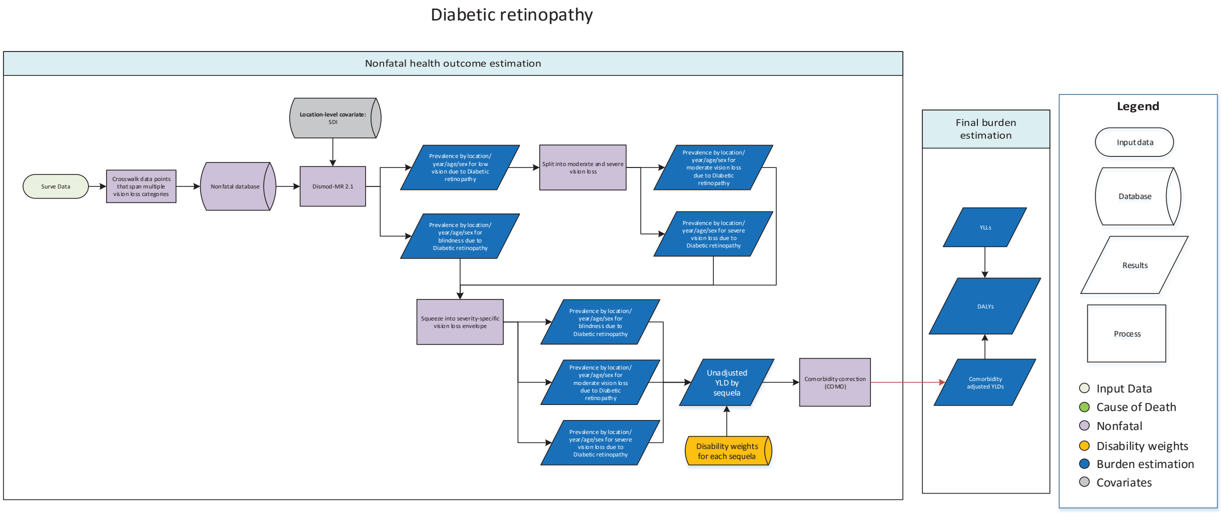

Macular Degeneration

Macular degeneration

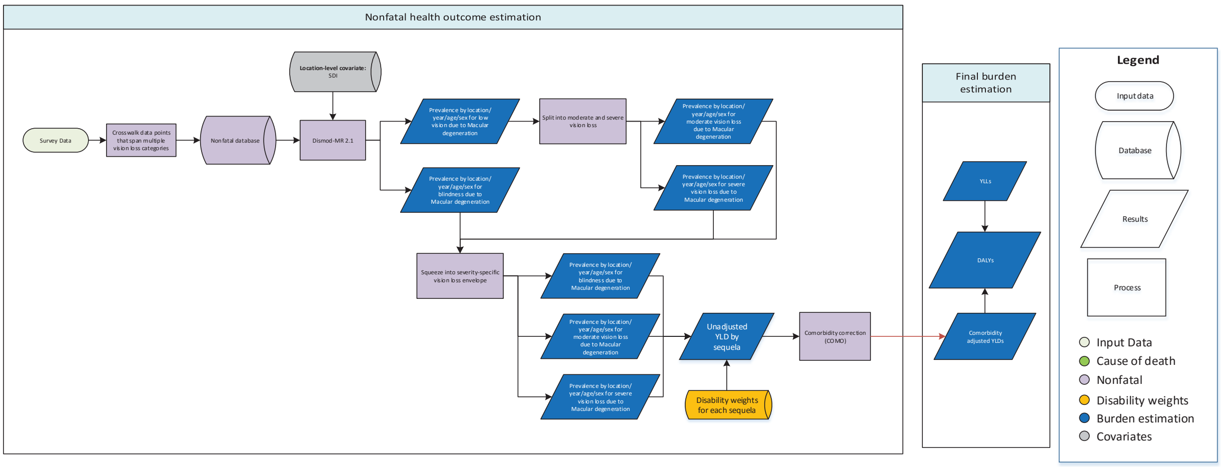

Cataract

Cataract

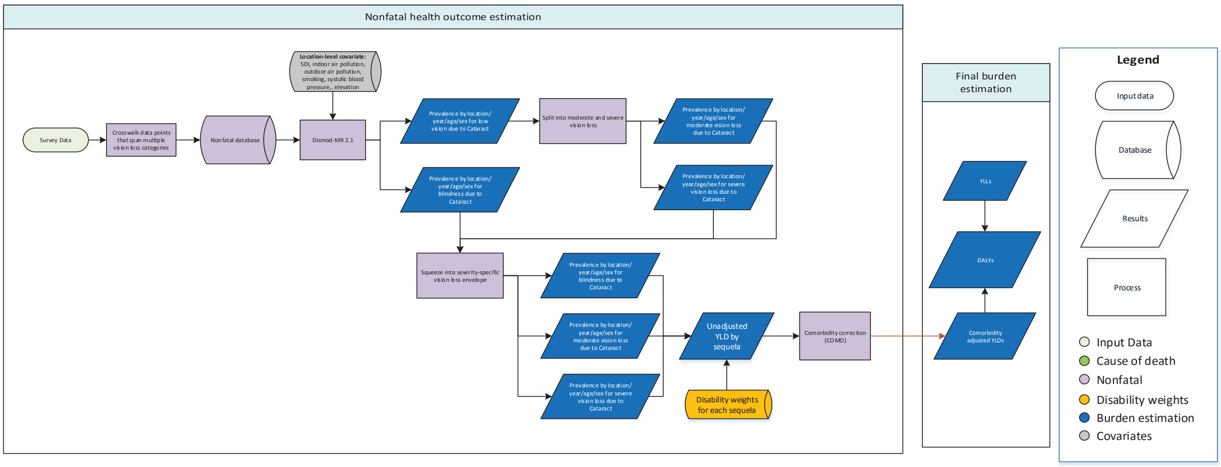

Glaucoma

Glaucoma

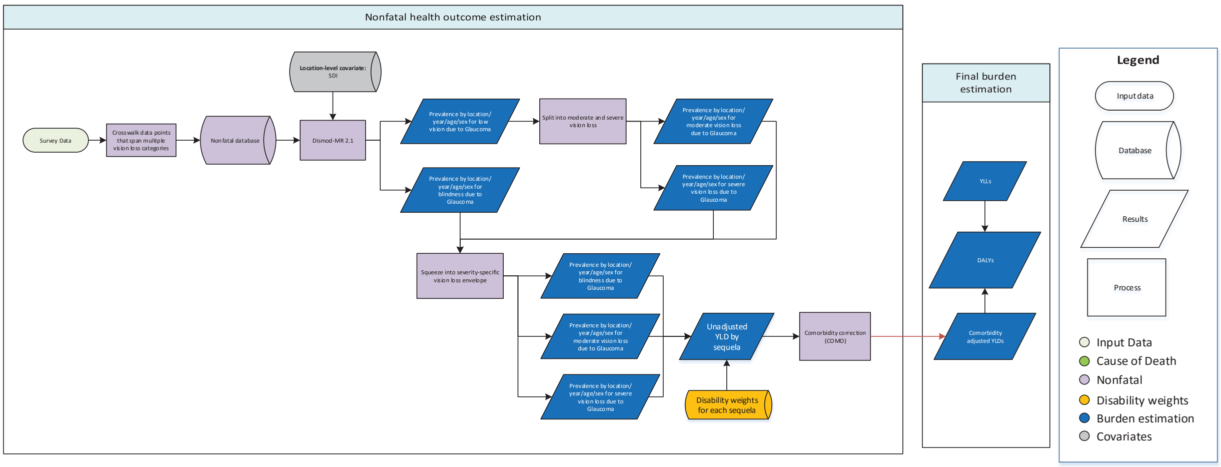

Trachoma

Trachoma

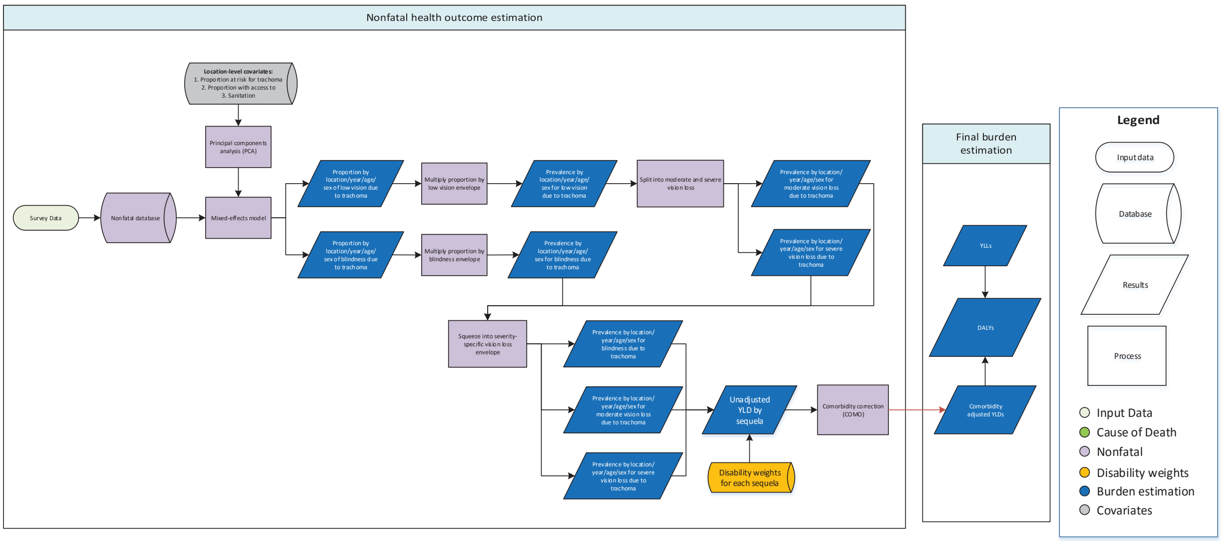

Other vision loss

Other vision loss

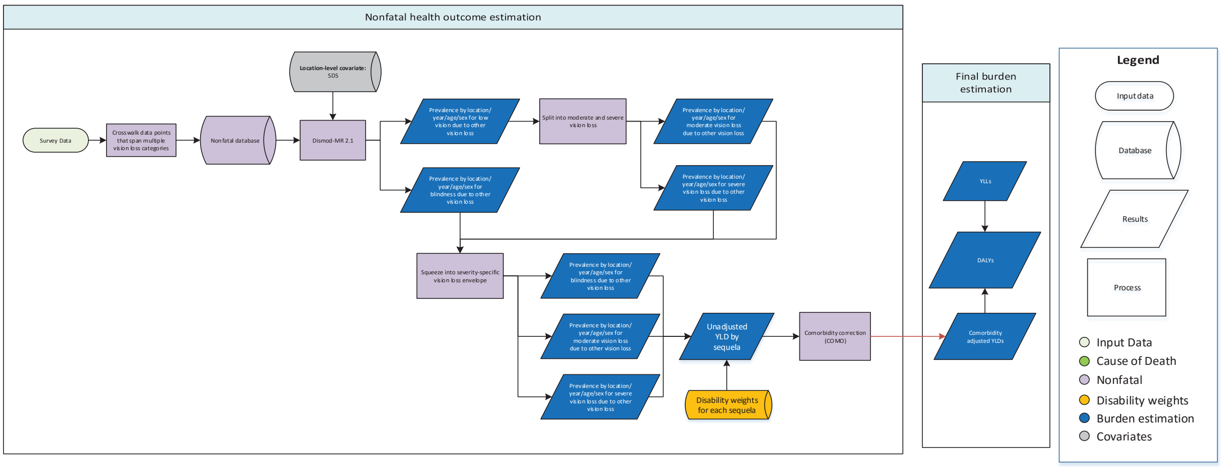

Case definition

We model vision impairment as visual acuity <6/18 according to the Snellen chart. The following impairments are modeled:

Condition	Case definition
Blindness	Visual acuity of <3/60 or <10% visual field around central fixation
Severe vision impairment	≥3/60 and <6/60
Moderate vision impairment	≥6/60 and <6/18
Near vision impairment envelope	Near visual acuity of <6/18 distance equivalent

Near vision impairment describes the progressive inability to focus on near objects as individuals age, and is also called presbyopia. This impairs the ability to read. The majority of presbyopia can be corrected by the use of reading glasses, contact lenses, or refractive surgery.

We model vision impairment due to the following causes: uncorrected refractive error, cataract, glaucoma, macular degeneration, diabetic retinopathy, trachoma, Vitamin A deficiency, retinopathy of prematurity, meningitis, encephalitis, onchocerciasis, and other vision loss. Vision loss due to vitamin A deficiency, retinopathy of prematurity, meningitis, encephalitis, and onchocerciasis are modelled as part of their underlying cause as described in their respective sections.

Refractive error is blurry vision due to the lens's inability to focus. The blurriness caused by refractive error can be addressed through the use of contact lenses, glasses, or refractive surgery. Cataract is

clouding of the lens of the eye due to protein buildup that impairs vision. Glaucoma is a condition with increased intraocular pressure which can lead to damage of the optic nerve. Macular degeneration is a deterioration of the macula, leading to central vision loss. Diabetic retinopathy is damage to the retina caused by damaged blood vessels that can leak blood into the retina and cause scarring of the retina. Trachoma results from a conjunctival bacterial infection (*Chlamydia trachomatis*) that produces inflammation and scarring which leads to an inversion of the eyelids and eyelashes scratching the cornea, which eventually leads to scarring of the cornea and vision impairment or blindness.

Input data

Model inputs

Data on overall vision impairment come from surveys measuring visual acuity in representative population-based studies, either from publications in peer-reviewed and grey literature or surveys for which we had the unit record data. Data were excluded if no test was used of visual acuity that can be converted to the Snellen scale, and if a study did not assess “presenting” or “best-corrected” vision. A subset of these studies that reported vision loss by cause were used to estimate the prevalence of vision loss due to cataract, glaucoma, macular degeneration, diabetic retinopathy, and other causes.

For GBD 2015, we conducted a systematic review for new sources since GBD 2013 (covering 1/1/2013 – 5/20/2015), using the following search string:

((((glaucoma[Title/Abstract] OR cataract[Title/Abstract] OR macular[Title/Abstract] OR 'refractive error'[Title/Abstract] OR presbyopia[Title/Abstract]) OR (('blindness'[MeSH Terms] OR 'blindness'[All Fields]) OR 'vision, low'[MeSH Terms])) AND ('2013'[PDAT] : '3000'[PDAT])) AND 'humans'[MeSH Terms]) AND (prevalence[Title/Abstract] OR incidence[Title/Abstract] OR epidemiology[Title/Abstract])

This yielded 1,169 results, of which we extracted 20 sources. Furthermore, we extracted from the following nationally representative surveys measuring visual acuity: the WHO Studies on Global Ageing and Adult Health (SAGE) and the United States National Health and Examination Surveys (NHANES).

For GBD 2016, we did a comprehensive extraction of the Rapid Assessment of Avoidable Blindness (RAAB) repository (http://raabdata.info/), a database of vision impairment studies in developing settings across the world. There are 266 site-years of data, the majority of which have publicly available reports or publications of the data. A standardized methodology was used by all sources in the repository, allowing inclusion of all available reports. In addition, we added two state-level national surveys from India.

Due to the sparse literature reporting measured near-vision visual acuity, we also extracted data from the following nationally representative studies measuring self-reported near vision loss: SAGE; NHANES; the Surveys of Health, Ageing, and Retirement in Europe (SHARE); the Multi-Country Survey Study on Health and Responsiveness (MCSS); and the World Health Surveys (WHS).

Several adjustments were made to raw data.

1) Where studies reported visual acuity spanning multiple thresholds (eg, <6/60, rather than separate severe and blind estimates), we crosswalked using ratios predicted by a linear regression on age, using data from studies reporting vision loss by each severity.

2) Some studies reported best-corrected vision impairment, but not presenting vision impairment (PVI). We crosswalked these data points using a linear regression of logit-transformed PVI prevalence with fixed effects on best-corrected VI, healthcare quality and access index (HAQI) and Socio-demographic Index (SDI) and super-region random effects. This gave us a predicted PVI data points for these studies not explicitly reporting PVI. These crosswalked data points were flagged with a study-level covariate that increased standard error in DisMod.
3) Where data points spanned more than 20 years of age, we age-split using an algorithm that applies the age-pattern of the super-region to split the data to five-year age groups.

Whereas other vision impairment aetiologies are modelled based on prevalence data, vision impairment due to trachoma is modelled as a proportion of the overall vision impairment envelope, a strategy that was chosen based on the nature of available data.

Health states and disability weights

Health state name	Health state description	Disability weight
Distance vision, severe impairment	This person has severe vision loss, which causes difficulty in daily activities, some emotional impact (for example, worry), and some difficulty going outside the home without assistance.	0.184 (0.125–0.259)
Distance vision, moderate impairment	This person has vision problems that make it difficult to recognize faces or objects across a room.	0.031 (0.019–0.049)
Distance vision blindness	This person is completely blind, which causes great difficulty in some daily activities, worry and anxiety, and great difficulty going outside the home without assistance.	0.187 (0.124–0.26)
Presbyopia	This person has difficulty seeing things that are nearer than 3 feet, but has no difficulty with seeing things at a distance.	0.011 (0.005–0.02)

Modelling strategy

We modelled the prevalence of vision loss in two steps. In the first step, we estimated the total prevalence estimates of presenting vision loss: moderate vision impairment, severe vision impairment, blindness, and near vision impairment (presbyopia). We directly derived prevalence of near vision impairment from this step, whereas the remaining three models that reflect different severity levels of distance vision loss continued to the next step.

1) **Estimate severity-specific vision impairment (the "envelopes")**

First, we ran five DisMod-MR 2.1 models to estimate the total prevalence estimates of presenting vision loss: moderate vision impairment, severe vision impairment, blindness, near vision impairment (presbyopia), and presenting vision impairment (moderate + severe + blindness). The presenting vision impairment model was used as a covariate in the severity-specific models to improve consistency across severities.

Betas and exponentiated values, which can be interpreted as an odds ratio, are shown in the table below for each covariate. The best-corrected covariate indicates whether the test measures visual acuity with the level of correction the patient presents with (best_corrected = 0) or the ophthalmologist provides additional correction via pinhole (best_corrected = 1). Rapid-assessment corrects for potential biases in cause-specific vision loss from studies using expedited visual acuity measurement. Socio-demographic

Index (SDI) and healthcare access and quality index (HAQI) are used as location covariates as a proxy measure of access to eye care such as cataract surgery. Non-representative studies are those not representative at the level they are used to model (eg, a state-level survey assigned to a country), including a z-cov adjusts for potential bias. Data points that were crosswalked from best-corrected visual acuity are flagged with a z-cov to adjust uncertainty in the crosswalk process. Non-standard severity definition is used to crosswalk between the self-report questionnaire of SHARE (nonstandard) and the other surveys, including SAGE and NHANES, which are crosswalked to examination data using the self-reported covariate.

Model	Covariate name	Type	Measure	Beta value	Exponentiated value
Vision impairment due to glaucoma unsqueezed	Socio-demographic Index	Country covariate	Prevalence	-0.235 (-0.690 - -0.008)	0.791 (0.502 - 0.992)
Vision impairment due to glaucoma unsqueezed	diagnostic rapid assessment of loss	Study-level x-covariate	Prevalence	0.012 (0.002 - 0.033)	1.012 (1.002 - 1.033)
Vision impairment due to glaucoma unsqueezed	Not representative	Study-level z-covariate	Prevalence	0.022 (0.003 - 0.067)	1.023 (1.003 - 1.069)
Blindness due to glaucoma unsqueezed	Socio-demographic Index	Country covariate	Prevalence	-0.256 (-0.690 - -0.010)	0.775 (0.501 - 0.990)
Blindness due to glaucoma unsqueezed	diagnostic rapid assessment of loss	Study-level x-covariate	Prevalence	0.010 (0.000 - 0.030)	1.010 (1.000 - 1.030)
Blindness due to glaucoma unsqueezed	Not representative	Study-level z-covariate	Prevalence	0.024 (0.000 - 0.085)	1.025 (1.000 - 1.089)
Vision impairment due to cataract unsqueezed	Elevation Over 1500m (proportion)	Country covariate	Prevalence	0.119 (0.006 - 0.319)	1.127 (1.006 - 1.376)
Vision impairment due to cataract unsqueezed	Indoor Air Pollution (All Cooking Fuels)	Country covariate	Prevalence	0.031 (0.000 - 0.111)	1.032 (1.000 - 1.118)
Vision impairment due to cataract unsqueezed	Outdoor Air Pollution (PM2.5)	Country covariate	Prevalence	0.008 (0.003 - 0.014)	1.008 (1.003 - 1.014)
Vision impairment due to cataract unsqueezed	Smoking Prevalence (Age-standardized, both sexes)	Country covariate	Prevalence	0.776 (0.035 - 1.675)	2.174 (1.036 - 5.340)
Vision impairment due to cataract unsqueezed	Socio-demographic Index	Country covariate	Prevalence	-0.612 (-0.962 - -0.157)	0.542 (0.382 - 0.855)
Vision impairment due to cataract unsqueezed	Systolic Blood Pressure (mmHg)	Country covariate	Prevalence	0.002 (0.000 - 0.007)	1.002 (1.000 - 1.007)

Vision impairment due to cataract unsqueezed	diagnostic rapid assessment of loss	Study-level x-covariate	Prevalence	0.031 (0.009 - 0.063)	1.031 (1.009 - 1.065)
Vision impairment due to cataract unsqueezed	Not representative	Study-level z-covariate	Prevalence	0.014 (0.003 - 0.039)	1.014 (1.003 - 1.039)
Blindness due to cataract unsqueezed	Elevation Over 1500m (proportion)	Country covariate	Prevalence	0.641 (0.420 - 0.868)	1.898 (1.522 - 2.382)
Blindness due to cataract unsqueezed	Indoor Air Pollution (All Cooking Fuels)	Country covariate	Prevalence	0.408 (0.143 - 0.660)	1.504 (1.153 - 1.936)
Blindness due to cataract unsqueezed	Outdoor Air Pollution (PM2.5)	Country covariate	Prevalence	0.000 (0.000 - 0.001)	1.000 (1.000 - 1.001)
Blindness due to cataract unsqueezed	Smoking Prevalence (Age-standardized, both sexes)	Country covariate	Prevalence	0.757 (0.036 - 1.723)	2.132 (1.036 - 5.601)
Blindness due to cataract unsqueezed	Socio-demographic Index	Country covariate	Prevalence	-0.965 (-1.000 - -0.864)	0.381 (0.368 - 0.421)
Blindness due to cataract unsqueezed	Systolic Blood Pressure (mmHg)	Country covariate	Prevalence	0.002 (0.000 - 0.008)	1.002 (1.000 - 1.009)
Blindness due to cataract unsqueezed	diagnostic rapid assessment of loss	Study-level x-covariate	Prevalence	0.002 (0.000 - 0.009)	1.002 (1.000 - 1.009)
Blindness due to cataract unsqueezed	Not representative	Study-level z-covariate	Prevalence	0.004 (0.000 - 0.010)	1.004 (1.000 - 1.010)
Vision impairment due to macular degeneration unsqueezed	Socio-demographic Index	Country covariate	Prevalence	0.350 (-0.432 - 0.921)	1.419 (0.650 - 2.512)
Vision impairment due to macular degeneration unsqueezed	diagnostic rapid assessment of loss	Study-level x-covariate	Prevalence	0.047 (0.005 - 0.126)	1.049 (1.005 - 1.134)
Vision impairment due to macular degeneration unsqueezed	Not representative	Study-level z-covariate	Prevalence	0.058 (0.008 - 0.150)	1.060 (1.008 - 1.162)
Blindness due to macular degeneration unsqueezed	Socio-demographic Index	Country covariate	Prevalence	0.328 (-0.563 - 0.959)	1.389 (0.570 - 2.609)
Blindness due to macular degeneration unsqueezed	diagnostic rapid assessment of loss	Study-level x-covariate	Prevalence	0.016 (0.002 - 0.050)	1.016 (1.002 - 1.052)
Blindness due to macular degeneration unsqueezed	Not representative	Study-level z-covariate	Prevalence	0.014 (0.001 - 0.047)	1.014 (1.001 - 1.048)

Near vision impairment due to presbyopia due to uncorrected refractive error	Socio-demographic Index	Country covariate	Prevalence	-1.803 (-1.999 - -1.451)	0.165 (0.135 - 0.234)
Near vision impairment due to presbyopia due to uncorrected refractive error	Non-standard severity definition	Study-level x-covariate	Prevalence	-0.195 (-0.200 - -0.186)	0.822 (0.819 - 0.830)
Near vision impairment due to presbyopia due to uncorrected refractive error	Self-reported	Study-level x-covariate	Prevalence	-0.102 (-0.120 - -0.089)	0.903 (0.886 - 0.915)
Vision impairment due to other vision loss unsqueezed	Socio-demographic Index	Country covariate	Prevalence	-0.113 (-0.351 - -0.006)	0.893 (0.704 - 0.994)
Vision impairment due to other vision loss unsqueezed	diagnostic rapid assessment of loss	Study-level x-covariate	Prevalence	0.054 (0.012 - 0.103)	1.056 (1.012 - 1.109)
Vision impairment due to other vision loss unsqueezed	Not representative	Study-level z-covariate	Prevalence	0.186 (0.126 - 0.239)	1.205 (1.135 - 1.270)
Blindness due to other vision loss unsqueezed	Socio-demographic Index	Country covariate	Prevalence	-0.179 (-0.472 - -0.005)	0.836 (0.624 - 0.995)
Blindness due to other vision loss unsqueezed	diagnostic rapid assessment of loss	Study-level x-covariate	Prevalence	0.164 (0.123 - 0.211)	1.178 (1.131 - 1.235)
Blindness due to other vision loss unsqueezed	Not representative	Study-level z-covariate	Prevalence	0.057 (0.019 - 0.107)	1.059 (1.020 - 1.113)
Vision impairment envelope	Socio-demographic Index	Country covariate	Prevalence	-1.899 (-1.997 - -1.605)	0.150 (0.136 - 0.201)
Blindness impairment envelope	Healthcare access and quality index	Country covariate	Prevalence	-0.020 (-0.024 - -0.013)	0.980 (0.976 - 0.987)
Blindness impairment envelope	Presenting vision impairment	Country covariate	Prevalence	0.506 (0.291 - 0.743)	1.659 (1.337 - 2.102)
Blindness impairment envelope	Socio-demographic Index	Country covariate	Prevalence	-0.115 (-0.345 - -0.002)	0.891 (0.708 - 0.998)
Blindness impairment envelope	Not representative	Study-level z-covariate	Prevalence	0.000 (0.000 - 0.002)	1.000 (1.000 - 1.002)
Blindness impairment envelope	best-corrected crosswalk	Study-level z-covariate	Prevalence	0.002 (0.000 - 0.007)	1.002 (1.000 - 1.007)
Moderate vision impairment envelope	Presenting vision impairment	Country covariate	Prevalence	0.775 (0.668 - 0.868)	2.170 (1.951 - 2.383)
Moderate vision impairment envelope	Socio-demographic Index	Country covariate	Prevalence	-0.041 (-0.170 - -0.000)	0.960 (0.844 - 1.000)

Moderate vision impairment envelope	Not representative	Study-level z-covariate	Prevalence	0.000 (0.000 - 0.002)	1.000 (1.000 - 1.002)
Moderate vision impairment envelope	best-corrected crosswalk	Study-level z-covariate	Prevalence	0.160 (0.130 - 0.194)	1.174 (1.139 - 1.214)
Severe vision impairment envelope	Presenting vision impairment	Country covariate	Prevalence	0.509 (0.383 - 0.636)	1.664 (1.466 - 1.889)
Severe vision impairment envelope	Socio-demographic Index	Country covariate	Prevalence	-0.018 (-0.056 - -0.001)	0.983 (0.945 - 0.999)
Severe vision impairment envelope	Not representative	Study-level z-covariate	Prevalence	0.001 (0.000 - 0.003)	1.001 (1.000 - 1.003)
Severe vision impairment envelope	best-corrected crosswalk	Study-level z-covariate	Prevalence	0.034 (0.011 - 0.062)	1.035 (1.011 - 1.064)
Vision impairment due to diabetes mellitus	Diabetes Age-Standardized Prevalence (proportion)	Country covariate	Prevalence	1.465 (0.673 - 2.255)	4.328 (1.961 - 9.535)
Vision impairment due to diabetes mellitus	Socio-demographic Index	Country covariate	Prevalence	-1.192 (-1.957 - -0.132)	0.304 (0.141 - 0.876)
Vision impairment due to diabetes mellitus	diagnostic rapid assessment of loss	Study-level x-covariate	Prevalence	0.011 (0.000 - 0.036)	1.011 (1.000 - 1.036)
Vision impairment due to diabetes mellitus	Not representative	Study-level z-covariate	Prevalence	0.089 (0.005 - 0.239)	1.093 (1.005 - 1.270)
Blindness due to diabetes mellitus unsqueezed	Diabetes Age-Standardized Prevalence (proportion)	Country covariate	Prevalence	3.805 (3.352 - 3.993)	44.905 (28.560 - 54.217)
Blindness due to diabetes mellitus unsqueezed	Socio-demographic Index	Country covariate	Prevalence	-1.594 (-1.989 - -0.597)	0.203 (0.137 - 0.550)
Blindness due to diabetes mellitus unsqueezed	diagnostic rapid assessment of loss	Study-level x-covariate	Prevalence	0.070 (0.002 - 0.230)	1.072 (1.002 - 1.259)
Blindness due to diabetes mellitus unsqueezed	Not representative	Study-level z-covariate	Prevalence	0.373 (0.193 - 0.586)	1.453 (1.213 - 1.796)
Moderate vision impairment due to uncorrected refractive error unsqueezed	Socio-demographic Index	Country covariate	Prevalence	-0.955 (-0.998 - -0.830)	0.385 (0.369 - 0.436)
Severe vision impairment due to uncorrected refractive error unsqueezed	Socio-demographic Index	Country covariate	Prevalence	-0.899 (-0.996 - -0.621)	0.407 (0.369 - 0.538)

Blindness due to uncorrected refractive error unsqueezed	Socio-demographic Index	Country covariate	Prevalence	-0.968 (-1.000 - -0.870)	0.380 (0.368 - 0.419)

2) Estimate cause-specific vision impairment

In the second step, we estimated the prevalence of vision loss due to multiple causes: refractive error, cataract, glaucoma, macular degeneration, diabetic retinopathy, retinopathy due to prematurity, trachoma, vitamin A deficiency, onchocerciasis, meningitis, and other causes not classified elsewhere. The vision loss due to retinopathy of prematurity, vitamin A deficiency, onchocerciasis, meningitis, tetanus, and neonatal conditions was modeled as part of these underlying causes. Vision loss due to trachoma is modelled as a proportion of the envelope, with separate proportion models for vision impairment and blindness. For each of cataract, glaucoma, macular degeneration, diabetic retinopathy, and other vision loss, we ran two DisMod-MR 2.1 models: one for the combined category of moderate and severe vision loss due to the cause, and one for blindness due to the cause. Moderate and severe vision loss were modelled together because input data were mostly available for the aggregate. Refractive error was modelled in three models, one for each severity. We used the following age restrictions:

Cause	Minimum age
Cataracts	20
Glaucoma	45
Macular degeneration	45
Diabetic retinopathy	20
Trachoma	15
Other vision loss	0

For the cataract model, we used known risk factors – hypertension, smoking, air pollution, and elevation.

For cataract and refractive error, we used presenting vision impairment as a covariate, as these are the main causes of vision impairment and are treatable and thus should have greater covariance with overall vision impairment than less common causes such as glaucoma or macular degeneration.

We estimated the proportions of low vision and blindness due to trachoma using custom mixed-effects models. For consistency, the two models (blindness and low vision) were parameterized identically and differ only in their input data. Our model included fixed effects on age (using cubic splines with knots at 0, 40, and 100 years of age), sex, and a covariate derived from a principal components analysis of the proportion of the population at risk for trachoma and the proportion of the population with access to sanitation. We included nested random effects on super-region, region, and country. Finally, we applied geographic and age restrictions to ensure that we estimate zero proportions in non-endemic locations and among those younger than 15 year of age (as scarring of the cornea due to trachoma takes decades to develop). The prevalence of trachoma at each severity level was calculated by multiplying the proportion of vision loss (vision impairment or blindness) due to trachoma by the corresponding best-corrected vision loss envelope.

We split the moderate plus severe vision loss estimates for each cause into moderate and severe using the ratio of best-corrected moderate and severe vision loss envelopes. As exceptions, onchocerciasis and retinopathy of prematurity were modelled for moderate and severe vision loss as part of the estimation process of these causes.

We scaled the cause-specific vision loss prevalence to the total prevalence of the best-corrected vision loss envelopes for each of the three severity levels. The final result is prevalence of vision loss due to each cause by severity.

The following changes have been implemented since GBD 2015:

- DisMod is not designed to handle wide-age data points – by age-splitting the input data we improve model fits.
- In the severity-specific vision impairment models, we use overall presenting vision impairment as a covariate, ensuring greater consistency between severities.
- In GBD 2013 vision impairment models, best-corrected vision data were crosswalked within DisMod using a single beta for all ages and locations. By crosswalking the input data, we allow the ratio between presenting and best-corrected vision impairment to vary with age and location.
- In GBD 2013, we estimated the ratio of vision impairment due to refractive error. In 2016, we are estimating the prevalence of refractive error, as it shows greater covariance with predictors such as SDI and HAQI. This allows the second step (squeezing causes to the envelopes) to include refractive error as an input.

Appendix 6

Autism

Flowchart

Autism

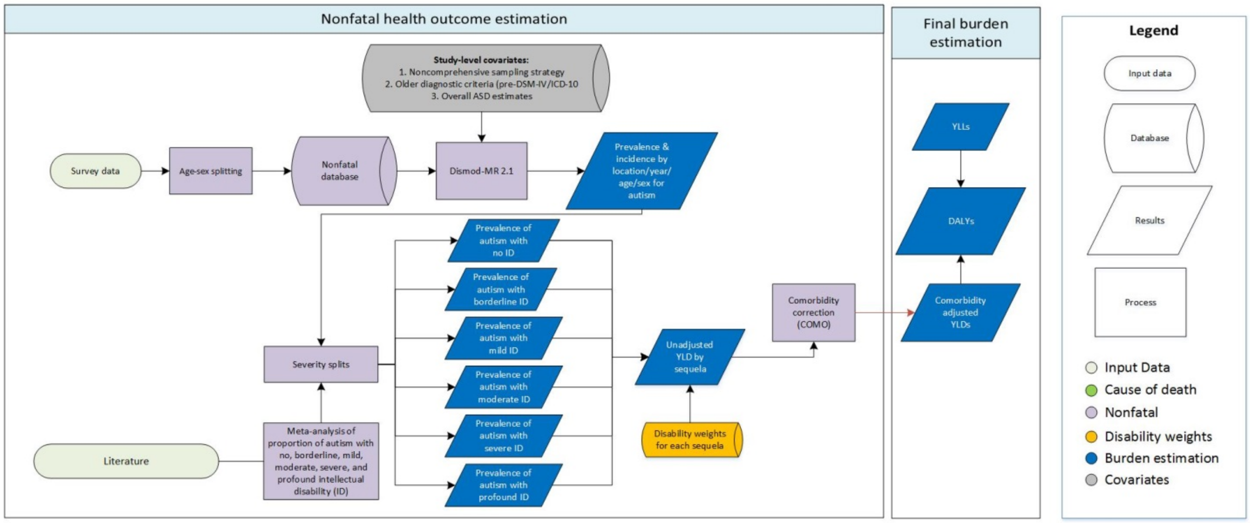

Case definition

Autism (also known as autistic disorder or childhood autism) is an autistic spectrum disorder (ASD) with onset occurring in early childhood. It is characterized by severe and pervasive impairment in several areas of development, including social interaction and communication skills, along with restricted and repetitive patterns of behaviors and/or interests. As per criteria set by the Diagnostic and Statistical Manual of Mental Disorders fourth edition, text revision (DSM-IV-TR),[1] diagnosis requires a total of six (or more) symptoms, with at least two symptoms of qualitative impairment in social interaction and at least one symptom of both qualitative impairment in communication and restricted, repetitive, stereotyped behavior. The recognized symptoms include:

Qualitative impairment in social interaction

a) marked impairment in the use of multiple nonverbal behaviors such as eye-to-eye gaze, facial expression, body postures, and gestures to regulate social interaction
b) failure to develop peer relationships appropriate to developmental level
c) a lack of spontaneous seeking to share enjoyment, interests, or achievements with other people
d) lack of social or emotional reciprocity

Qualitative impairments in communication

a) delay in, or total lack of, the development of spoken language (not accompanied by an attempt to compensate through alternative modes of communication such as gesture)
b) in individuals with adequate speech, marked impairment in the ability to initiate or sustain a conversation with others
c) stereotyped and repetitive use of language or idiosyncratic language
d) lack of varied, spontaneous make-believe play or social imitative play appropriate to developmental level

Restricted repetitive and stereotyped patterns of behavior, interests, and activities

a) encompassing preoccupation with one or more stereotyped and restricted patterns of interest that is abnormal either in intensity or focus
b) apparently inflexible adherence to specific, nonfunctional routines or rituals
c) stereotyped and repetitive motor mannerisms
d) persistent preoccupation with parts of objects

Delays or abnormal functioning with onset prior to three years of age in at social interaction, language interaction, or symbolic or imaginate play is also required. Included in GBD were cases meeting diagnostic criteria according to DSM[1] or the International Classification of Diseases (ICD).[2] These were identified by the following codes: 299.00 (DSM-IV-TR) and F84 (ICD-10). Different versions of DSM (DSM-III, DSM-III-R, DSM-IV, DSM-IV-TR, and DSM-5) and ICD (ICD-9 and ICD-10) were accepted.

Input data

Model inputs

A series of systematic literature reviews were conducted to capture studies reporting the prevalence, incidence, remission, and excess mortality of autism. The reviews incorporated searches of peer-reviewed literature via electronic databases, investigations of grey literature, and consultation with experts. In order for a study to be included, it must have been published during or after 1980, use DSM or ICD criteria to define cases, provide sufficient details on study methodology and sample characteristics to determine study quality, and be representative of the general population rather than a special population eg, prison inmates. No limitation was set on the language of publication. Detailed descriptions of this methodology have been published elsewhere.[3] This methodology was utilized in GBD 2010, GBD 2013, and GBD 2016.

Prevalence estimates were split by age and sex where possible outside of DisMod-MR 2.1. Firstly if studies reported prevalence for broad age groups by sex (eg, prevalence in 15 to 65 year old males and females separately), and also by specific age groups but for both sexes combined (eg, prevalence in 15 to 30 year olds, then in 31 to 65 year olds, for males and females combined); age-specific estimates were split by sex using the reported sex ratio and bounds of uncertainty. Secondly, where studies reported estimates across age groups spanning 20 years or more, these were split into five-year age groups using the regional prevalence age pattern estimated by DisMod-MR 2.1.

The final dataset for GBD 2016 included 174 prevalence estimates, 24 incidence estimates, 5 remission estimates, and 11 standardized mortality ratio estimates. The table below shows the number of studies for each parameter as well as the number of countries/subnationals and GBD world regions covered by the available data.

	Prevalence	Incidence	Remission	Mortality
Studies	67	3	5	3
Countries/subnationals	50	3	4	3
GBD world regions	10	3	2	2

Severity split inputs

Autism is one of the causes that contributes to the intellectual disability (ID) envelope. As such, a gradation of autism by level of severity was needed. Meta-analyses were conducted using data from six studies reporting information on the IQ level in those with autism in order to calculate the severity splits

by six sequelae: autism with 1) no ID, 2) borderline ID, 3) mild ID, 4) moderate ID, 5) severe ID, and 6) profound ID. The lay descriptions and disability weights for autism and each level of intellectual disability are shown in the table below.

Health state	Lay description	DW (95% CI)
Autism	Has severe problems interacting with others and difficulty understanding simple questions or directions. The person has great difficulty with basic daily activities and becomes distressed by any change in routine.	0.262 (0.176–0.365)
ID, borderline	Is slow in learning at school. As an adult, the person has some difficulty doing complex or unfamiliar tasks but otherwise functions independently.	0.011 (0.005–0.024)
ID, mild	Has low intelligence and is slow in learning at school. As an adult, the person can live independently, but often needs help to raise children and can only work at simple supervised jobs.	0.043 (0.028–0.067)
ID, moderate	Has low intelligence, and is slow in learning to speak and to do even simple tasks. As an adult, the person requires a lot of support to live independently and raise children. The person can only work at the simplest supervised jobs.	0.098 (0.064–0.142)
ID, severe	Has very low intelligence and cannot speak more than a few words, needs constant supervision and help with most daily activities, and can do only the simplest tasks.	0.157 (0.104–0.219)
ID, profound	Has very low intelligence, has almost no language, and does not understand even the most basic requests or instructions. The person requires constant supervision and help for all activities.	0.196 (0.126–0.272)

Modelling strategy

We assumed no incidence from 15 years of age onward. A small setting was placed on excess mortality whereby only minimal excess mortality was allowed over the lifespan. Remission was set to 0 after expert consultation revealed we would not expect remission for autism. Settings for excess mortality and remission differ from settings used in GBD 2015 where excess mortality was originally set to 0 and a small setting was placed on remission whereby only minimal remission was allowed over the lifespan. The mortality data in the autism dataset consist of standardized mortality ratio estimates from high-income locations. DisMod MR 2.1 produced good global fit for these data; however, the region fit did not follow the data, leading to high estimates of standardised mortality ratio in high-income countries and low estimates of standardised mortality ratio in low- and middle-income countries. Excess mortality estimates by age, sex, and year were therefore calculated by pulling global estimates of standardised mortality ratio from DisMod MR 2.1 and applying the following formula:

$$\text{excess mortality rate} = (\text{standardized mortality ratio} - 1) \times \text{all cause mortality rate}$$

Three study-level covariates were applied which: 1) adjusted estimates using a limited sampling strategy towards those using comprehensive sampling strategies (eg, those including private households and mainstream schools as well as healthcare and remedial therapy facilities), and 2) adjusted estimates based on older diagnostic criteria (prior to DSM-IV and ICD-10) toward estimates made using more

current criteria, and 3) adjusted estimates of ASD overall (ie, studies that did not report on the individual disorders) toward estimates representing autism. The third covariate is an addition in GBD 2016.

Study covariate	Parameter	beta	Exponentiated beta
Non-comprehensive sampling strategy	Prevalence	-0.3 (-0.58 – -0.028)	0.74 (0.56–0.97)
Identifies data classified by older criteria (ie, before DSM-IV and ICD-10)	Prevalence	-0.53 (-0.88 – -0.18)	0.59 (0.42–0.83)
Overall ASD estimates	Prevalence	1.26 (0.87–1.63)	3.54 (2.40–5.11)

References

1. American Psychiatric Association. Diagnostic and Statistical Manual of Mental Disorders (DSM-IV-TR). 4th, Text Revision ed. Washington DC: American Psychiatric Association; 2000.
2. World Health Organisation. ICD-10 Classification of Mental and Behavioural Disorders: Clinical descriptions and diagnostic guidelines. Geneva: World Health Organisation; 1992.
3. Baxter AJ, Brugha TS, Erskine HE, Scheurer RW, Vos T, Scott JG. The epidemiology and global burden of autism spectrum disorders. *Psychological Medicine* 2014; **45**(3): 601-13.

Appendix 7

Attention-deficit/hyperactivity disorder (ADHD)

Flowchart

Attention-deficit/hyperactivity disorder

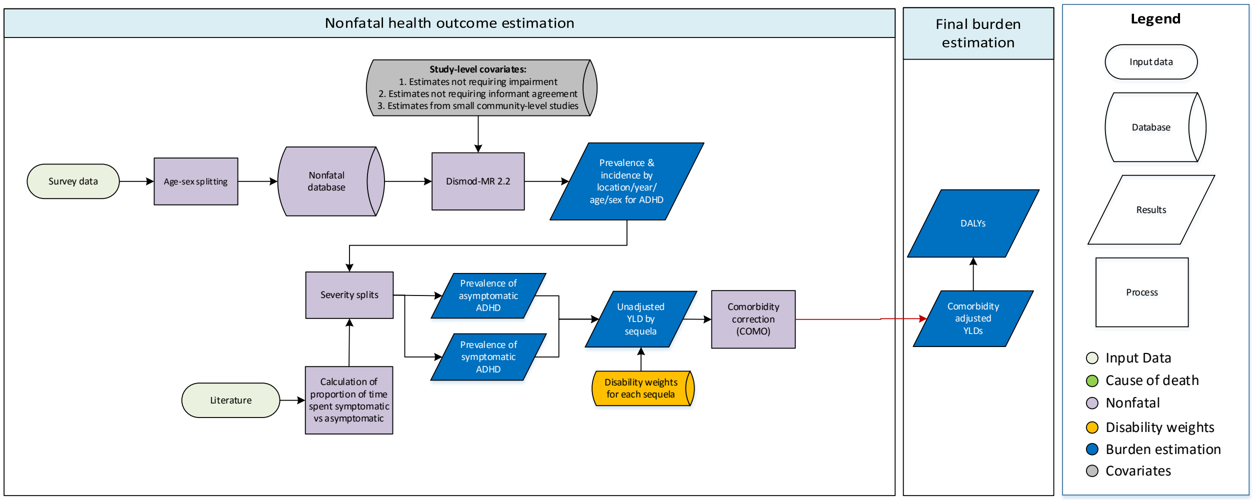

Case definition

Attention-deficit/hyperactivity disorder (ADHD) is an externalizing behavior disorder characterized by persistent inattention and/or hyperactivity-impulsivity. As per criteria set by the Diagnostic and Statistical Manual of Mental Disorders fourth edition, text revision (DSM-IV-TR)[1], diagnosis requires six or more symptoms of inattention or hyperactivity-impulsivity to have persisted for at least six months in two or more settings causing significant impairment to functioning, with at least some impairing symptoms being present prior to 7 years of age (12 years of age in DSM-5[2]). Recognized symptoms include:

Inattention:

- often fails to give close attention to details or makes careless mistakes in schoolwork, work, or other activities
- often has difficulty sustaining attention in tasks or play activities
- often does not seem to listen when spoken to directly
- often does not follow through on instructions and fails to finish school work, chores, or duties in the workplace (not due to oppositional behavior or failure to understand instructions)
- often has difficulty organizing tasks and activities
- often avoids, dislikes, or is reluctant to engage in tasks that require sustained mental effort (such as schoolwork or homework)
- often loses things necessary for tasks or activities (eg, toys, school assignments, pencils, books, or tools)
- is often easily distracted by extraneous stimuli
- is often forgetful in daily activities

Hyperactivity

- often fidgets with hands or feet or squirms in seat

- often leaves seat in classroom or in other situations in which remaining seated is expected
- often runs about or climbs excessively in situations in which it is inappropriate (in adolescents or adults, may be limited to subjective feelings of restlessness)
- often has difficulty playing or engaging in leisure activities quietly
- is often "on the go" or often acts as if "driven by a motor"
- often talks excessively

Impulsivity

- often blurts out answers before questions have been completed
- often has difficulty awaiting turn
- often interrupts or intrudes on others (eg, butts into conversations or games)

Included in GBD were cases meeting diagnostic criteria according to DSM[1] or the International Classification of Diseases (ICD)[3] (called "hyperkinetic disorder" in ICD). These were identified by the following codes: 314.0, 314.01 (DSM-IV-TR) and F90 (ICD-10). Different versions of DSM (DSM-III, DSM-III-R, DSM-IV, DSM-IV-TR, and DSM-5) and ICD (ICD-9 and ICD-10) were accepted.

Input data

Model inputs

A series of systematic literature reviews were conducted to capture studies reporting the prevalence, incidence, remission, and excess mortality of ADHD. The reviews incorporated searches of peer-reviewed literature via electronic databases, investigations of grey literature, and consultation with experts. In order for a study to be included, it must have been published during or after 1980, use DSM or ICD criteria to define cases, provide sufficient details on study methodology and sample characteristics to determine study quality, and be representative of the general population rather than a special population, eg, prison inmates. No limitation was set on the language of publication. Detailed descriptions of this methodology have been published elsewhere.[4] This methodology was utilized in GBD 2010 and GBD 2013. GBD 2015 included additional sources identified by GBD experts and microdata where available. The systematic review methodology used in GBD 2010 and 2013 was replicated to update the dataset for GBD 2016.

The final dataset for GBD 2016 included 290 prevalence estimates, five incidence estimates, 20 remission estimates, and three excess mortality estimates. The table below shows the number of studies for each parameter as well as the number of countries/subnationals and GBD world regions covered by the available data.

	Prevalence	Incidence	Remission	Mortality
Studies	130	2	14	2
Countries/subnationals	77	2	12	2
GBD world regions	17	1	3	2

Severity split inputs

A severity split for the proportion of time spent symptomatic versus asymptomatic was based on data from the Great Smoky Mountains Study which assessed the levels of disability found in children and adolescents with mental disorders.[5] Of those with ADHD, 48% reported disability while 20% of individuals with no diagnosis reported disability at the time of survey. Using these as estimates of the proportion of

time with disability in the "average case," the proportion of disability in children without a diagnosis was subtracted from the proportion with disability for ADHD, giving an adjusted proportion of 28%. Detailed descriptions of this methodology have been published elsewhere.[6] The lay description and disability weight for ADHD is shown in the table below.

Lay description	DW (95% CI)
Is hyperactive and has difficulty concentrating, remembering things, and completing tasks	0.045 (0.028–0.066)

Modelling strategy

We assumed no incidence prior to 3 years of age or onward from 12 years of age. The minimum age of onset was set in consultation with experts and based on current literature, while the upper age limit on incidence was set in line with the latest DSM-5 criteria. Remission was set to zero prior to 12 years, in line with the restriction on incidence. Excess mortality was set to zero given only three estimates were found for this parameter. Three covariates were included in the model. The first covariate was an informant covariate which adjusted estimates not requiring agreement between informants (eg, diagnosis made if either a teacher or parent indicates ADHD) toward estimates which required informant agreement. The second covariate adjusted estimates not requiring impairment (or those not specifying whether impairment was required) for diagnosis toward those which required impairment. The third covariate adjusted studies using small, community samples toward studies representative of entire regions or countries. Bounds for these covariates were calculated from the epidemiological data and applied in DisMod-MR 2.2.

Study covariate	Parameter	beta	Exponentiated beta
No informant agreement	Prevalence	0.67 (0.46–0.95)	1.96 (1.59–2.59)
No impairment	Prevalence	0.13 (0.008–0.30)	1.14 (1.01–1.34)
Small, community-level studies	Prevalence	0.62 (0.35–0.79)	1.86 (1.42–2.21)

References

1. American Psychiatric Association. Diagnostic and Statistical Manual of Mental Disorders (DSM-IV-TR). 4th, Text Revision ed. Washington DC: American Psychiatric Association; 2000.
2. American Psychiatric Association. Diagnostic and Statistical Manual of Mental Disorders, Fifth Edition. Arlington, VA: American Psychiatric Association; 2013.
3. World Health Organisation. ICD-10 Classification of Mental and Behavioural Disorders: Clinical descriptions and diagnostic guidelines. Geneva: World Health Organisation; 1992.
4. Erskine HE, Ferrari AJ, Nelson P, et al. Research Review: Epidemiological modelling of attention-deficit/hyperactivity disorder and conduct disorder for the Global Burden of Disease Study 2010. *Journal of Child Psychology and Psychiatry* 2013; **54**(12): 1263-74.
5. Ezpeleta L, Keeler G, Erkanli A, Costello EJ, Angold A. Epidemiology of Psychiatric Disability in Childhood and Adolescence. *J Child Psychol Psychiatry* 2001; **42**(7): 901-14.
6. Erskine HE, Ferrari AJ, Polanczyk GV, et al. The global burden of conduct disorder and attention-deficit/hyperactivity disorder in 2010. *J Child Psychol Psychiatry* 2014; **55**(4): 328-36.

Appendix 8. Crude Estimate of Children at risk of Sub-optimal Development in Early Childhood in LMICs

We arrived at the **crude** estimate of 350 million for LMICs by consensus as follows. Firstly, based on the reported annualised rate of decline of approximately 1.1% (Lu 2016), we estimated that the number of children exposed to stunting and extreme poverty would have reduced to about 234 million in 2016. No adjustment was made on the 80.8 million reported by McCoy et al 2016, as no significant change was anticipated between 2015 and 2016. We then combined the three crude estimates (234 + 80.8 + 50.2) million to arrive at 365 million. We made a conservative allowance of approximately 15 million for possible overlaps in the three estimates.

	**Lu et al 2016**	**McCoy et al 2016**	**Current Study**
**Global Estimate**	-	-	62.5 million
**Estimate for LMICs**	250 million	80.8 million	50.2 million
**Year**	2010	2015	2016
**Measure of developmental risk or potential**	Stunting and extreme poverty	Low cognitive and/or socioemotional development using ECD Index	Epilepsy, Intellectual Disabilities, Hearing Loss, Vision Loss, ASD & ADHD
**Age group**	Under 5 years	3 and 4 years	Under 5 years
**Assumed annualised rate of change**	-1.1%	-	-

Considering that the current study excluded prominent disabilities like idiopathic cerebral palsy, and other motor disorders, the 350 million (or 60.9% of the under-5y population of 574.3 million in LMICs) would still appear to be an extremely conservative estimate. We wish to emphasise that this is a crude estimate as there is no straightforward statistical technique for combining the estimates from the three studies.

References

1. Lu C, Black MM, Richter LM. Risk of poor development in young children in low-income and middle-income countries: an estimation and analysis at the global, regional, and country level. Lancet Glob Health 2016; 4: e916-22.
2. McCoy DC, Peet ED, Ezzati M, et al. Early Childhood Developmental Status in Low- and Middle-Income Countries: National, Regional, and Global Prevalence Estimates Using Predictive Modelling. PLoS Med 2016; 13: e1002034.
